# High-Throughput and Accurate Determination of Transgene Copy Number and Zygosity in Transgenic Maize: From DNA Extraction to Data Analysis

**DOI:** 10.3390/ijms222212487

**Published:** 2021-11-19

**Authors:** Fang Liu, Jinkui Cheng, Xuhua Liu, Xi-Qing Wang

**Affiliations:** 1 Center for Crop Functional Genomics and Molecular Breeding, China Agricultural University, Beijing 100193, China; chengjinkui@cau.edu.cn; 2 State Key Laboratory of Plant Physiology and Biochemistry, College of Biological Sciences, China Agricultural University, Beijing 100193, China; 3 Department of Mathematics, China Agricultural University, Beijing 100193, China; liuxuhua@cau.edu.cn

**Keywords:** high throughput, transgene copy number, zygosity, maize endogenous reference gene, single-nucleotide polymorphism, TaqMan assay, Southern blot, digital PCR

## Abstract

It is vital to develop high-throughput methods to determine transgene copy numbers initially and zygosity during subsequent breeding. In this study, the target sequence of the previously reported endogenous reference gene *hmg* was analyzed using 633 maize inbred lines, and two SNPs were observed. These SNPs significantly increased the PCR efficiency, while the newly developed *hmg* gene assay (hmg-taq-F2/R2) excluding these SNPs reduced the efficiency into normal ranges. The TaqMan amplification efficiency of *bar* and *hmg* with newly developed primers was calculated as 0.993 and 1.000, respectively. The inter-assay coefficient of variation (CV) values for the *bar* and *hmg* genes varied from 1.18 to 2.94%. The copy numbers of the transgene *bar* using new TaqMan assays were identical to those using dPCR. Significantly, the precision of one repetition reached 96.7% of that of three repetitions of single-copy plants analyzed by simple random sampling, and the actual accuracy reached 95.8%, confirmed by T_1_ and T_2_ progeny. With the high-throughput DNA extraction and automated data analysis procedures developed in this study, nearly 2700 samples could be analyzed within eight hours by two persons. The combined results suggested that the new *hmg* gene assay developed here could be a universal maize reference gene system, and the new assay has high throughput and high accuracy for large-scale screening of maize varieties around the world.

## 1. Introduction

Currently, the genetic transformation of plants has been widely used to generate large numbers of plants with improved agronomic traits and without undesirable traits [1]. Transgenic crops have increased by about 112 times since 1996, with an accumulated biotech area of 2.7 billion hectares, making biotechnology the fastest adopted crop technology in the world. Maize is the second most prevalent biotech crop after soybean, with a cultivation area of 60.9 million hectares, representing 31% of the global biotech area in 2019 [2]. Rapid and reliable evaluation of transgenic alleles for copy numbers and homozygosity is an indispensable step in utilizing these transformation events, so that homozygous lines with stable transgene expression can be obtained for accurate testing. Typically, single-copy events are preferred for most research and commercial applications because they are stable over several generations of subsequent breeding and the Mendelian inheritance is simple [3,4,5]. Furthermore, single-copy homozygotes are highly desirable in transgenic plant research to ensure the stable integration and inheritance of transgenes, and they have uniform phenotypes [6,7]. Traditionally, T_1_ plants are screened for zygosity by segregation analysis of their progeny at the DNA level (PCR method) or by their antibiotic resistance [8]. However, progeny segregation analysis used to determine zygosity is time-consuming and laborious, and single-copy homozygotes cannot be obtained before the T_2_ generation. In order to screen large numbers of independent transformed events or progeny quickly and reliably, a reliable and high-throughput method must be found to govern the zygosity and copy numbers of transgenes, especially for crops that require a large space for planting and a long time to grow.

Southern blot analysis, a classic molecular biology method, is traditionally used to determine transgene copy numbers. It is generally reliable but has a limit in resolution and cannot differentiate fragments of similar sizes. Thus, it cannot tell homozygotes from heterozygotes in subsequent T_1_ and/or T_2_ segregated populations. Moreover, it can produce inaccurate numbers of transgene copies when tandem integration or rearrangement of transgene copies results in a lack of relevant restriction sites [9]. In addition, it is time-consuming and laborious and requires large amounts of DNA. These disadvantages make it impractical for large-scale screening of transgenic plants in the early stages [10].

In recent years, a rapid and sensitive method has been developed for estimating transgene copy numbers or zygosity by quantitative real-time PCR (qPCR). This method requires only small amounts of plant material and is easily automated for high-throughput quantitation. qPCR usually uses an endogenous gene with a single copy or known copy number as reference to assess the copy numbers of exogenous genes, and hence can be used to determine zygosity [9,10]. To accurately distinguish between one and two transgene copies, qPCR usually requires extensive experimental optimization by selecting primers that are nearly 100% efficient and using good-quality DNA [11,12]. In theory, qPCR analysis can detect the inserted transgene regardless of where it is located within the genome. So far, qPCR has been applied to successfully and rapidly determine the zygosity/copy numbers in plants, such as wheat [13,14], maize [10,15], rice [16,17], tomato [18], and soybean and peanut [19]. In these studies, qPCR analysis was performed using TaqMan^®^-labeled target-specific probes. The advantage of the TaqMan qPCR method is its high sensitivity, and probes can have distinct sequences within the same reaction.

Digital PCR (dPCR) is a novel technology that can accurately identify transgene copy numbers, discriminate between single and low transgene copies, and do such characterization even in transgenic plants with large genomes. dPCR has several advantages, such as its precision, accuracy even at very low target concentrations, suitability for routine analytics, and lower sensitivity to PCR inhibitors [20]. However, the main reason for the interest in using this technique for copy number detection is its useful ability to perform absolute quantification independent of a reference standard/calibration curve. These properties and advantages make dPCR a reliable substitute for Southern blot for transgene copy number and zygosity measurements in transgenic plants [21,22,23,24]. However, the major limitation of dPCR at present is its higher analytical cost and lower throughput compared to qPCR [25].

For both dPCR and qPCR, accurate measurement of a transgene copy number requires the ability to detect an endogenous reference gene with a known copy number. An ideal endogenous reference gene should display species specificity, single or low copy number in the genome, and low heterogeneity among different lines [26]. In general, low heterogeneity is determined by the minimum number of single-nucleotide polymorphisms (SNPs) in the target DNA sequence and high PCR amplification performance among different lines [27,28]. SNPs within the region of primers or probes used for the qPCR assay could lead to primer or probe/template mismatching and, further, alterations in the measured total DNA copy number [29,30,31]. For instance, an SNP in the forward primer sequence of an *Adh1* assay caused a measured decrease in total DNA copy numbers [28,29].

Maize has great genetic diversity, with many SNPs in the genome among different cultivars [32,33,34,35,36,37]. Indeed, Vroh Bi et al. [36] reported that SNPs occur in maize coding genes every 73 bp. To date, reports have identified at least five taxon-specific endogenous control reference genes for maize, including alcohol dehydrogenase-1 (ADH), high-mobility group a (HMGa), invertase-1 (IVR), zein, and starch-synthase-IIb (SSII-b), along with combinations of different primer/probe systems for several of these gene systems [28,38]. Some studies show that endogenous reference genes amplify inconsistently across different maize cultivars [38]. SNPs were found in tested target sequences of *Adh1* and *zein* assays [28,29,38]. In addition, the copy number of the *zein* gene can vary among maize cultivars [38]. The nucleotide sequence of the *ivr* gene is uniform; however, PCR performance test shows that there may be more than one copy of the target sequence [28]. Several studies reported that *hmg* and z*SSIIb* showed low variation in the quantified copy number among conventional maize varieties [28,29,38]. However, we should be aware that the nucleotide stability of the reference target sequences among the varieties might not have been sufficiently assessed because the abundance of the variety was not large enough.

In this study, we developed a high-throughput screening method using the duplexed TaqMan assay for estimating transgene copy numbers and determining zygosity in transgenic maize. First, this required the identification of the most applicable reference gene and its qPCR assay that could be widely used in different maize varieties all over the world. The target sequence of the previously reported endogenous reference gene *hmg* was analyzed using 633 maize inbred lines, and a new TaqMan assay (hmg-taq-F2/R2) was designed to exclude the two SNPs observed. To provide a highly efficient and accurate method for transgene copy numbers and zygosity, this new duplexed TaqMan assay also includes the use of a multiple-target plasmid as a calibrator and the efficiency-corrected formula. The TaqMan assay developed here not only can easily identify medium- and high-copy transgene insertions, but can identify low-copy transgene insertions as accurately as dPCR and more accurately than Southern blot analysis. These improvements yield another clear benefit: the higher precision observed with the TaqMan assay developed here can reduce the number of technical repetitions to only one. In order to meet the needs of high-throughput duplexed TaqMan assays, DNA extraction was performed in four 96-well plates at one time using magnetic beads, and data analysis was automated with a developed program. Eventually, we developed high-throughput assays for the entire pipeline, from DNA extraction to data analysis.

## 2. Results

### 2.1. Discovered SNPs in Target Regions of Endogenous Reference Gene among Maize Cultivars

**Figure 1:**
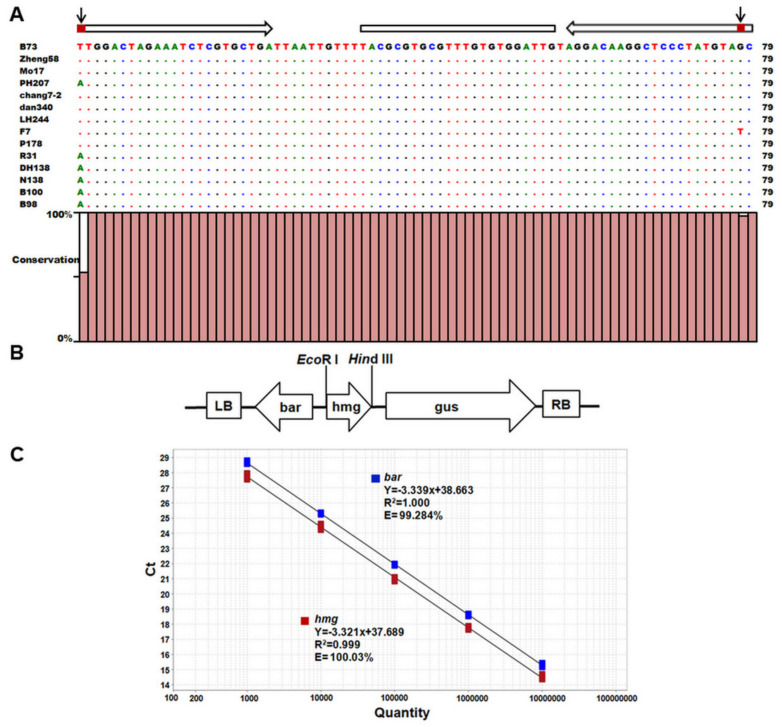
Development of universal TaqMan copy number assays in 384-well format for high-throughput screening. (**A**) Detected SNPs within amplified target DNA of *hmg* gene. Identical nucleotides are indicated by dots. White arrows and lines represent primers and probe attachment sites of *hmg* assay. Observed single-nucleotide polymorphism is shown in red boxes on white arrows. SNP of T to A was found at the first nucleotide from the 5′ end in the forward primer in maize line PH207, and another of G to T at the second nucleotide from the 5′ end in the reverse primer in maize line F7. No sequence variation was found in the probe. (**B**) Schematic diagram of pCAMBIA3301-ZmHMG. LB, left border; *bar*, phospinothricin acetyl transferase gene; *hmg*, fragment of maize endogenous gene *hmg*; *gus*, β-glucuronidase; RB, right border. Position of the *Eco*R I and *Hin*dIII restriction site is indicated above the T-DNA insert. (**C**) TaqMan assay efficiency for *hmg* and *bar* primer and probe sets. Five 10-fold serial dilutions of standard plasmid (1.0 × 10^7^ to 1.0 × 10^3^ copies/μL) were used in duplexed TaqMan copy number assays, as mentioned in Materials and Methods. Calculated Ct values were plotted against log of each concentration (copies/μL). Each sample was run in four replicates. Regression lines with their respective equations, correlation coefficient (R^2^), and amplification efficiency (E) are presented for each gene.

An ideal endogenous reference gene should be present as a consistent low copy number among different varieties; meanwhile, it should not exhibit allelic variation among varieties of the same species. Previous studies revealed that the *hmg* gene is maize-specific, with one copy number and low heterogeneity [28,29,38]. To determine whether there is any sequence variation among the target regions in the previous quantitative assays, 201 bp DNA fragments covering this region in 633 maize inbred lines were obtained by conventional PCR and sequenced. The expected DNA fragment of *hmg* gene could be amplified from all lines. After alignment analysis of the obtained DNA sequences from all lines, two SNPs (SNP719 and SNP796, related to the translational start codon) were discovered in qPCR target regions of *hmg* gene (Figure 1A, Supplementary Table S1). In SNP719, T to A at the first nucleotide from the 5′ end in the forward primer was observed in 112 maize lines (including PH207; 17.7%). In SNP796, G to T at the second nucleotide from the 5′ end in the reverse primer was observed in three maize lines (F7, DH40, and SS99; 0.47%). No sequence variation was found in the probe among all tested lines.

### 2.2. SNPs of Endogenous Reference Targets Affect qPCR Efficiency

To evaluate the effects of the discovered SNPs in *hmg* gene on qPCR assay, PCR efficiency in lines with detectable SNPs was analyzed. PCR efficiency of the cultivars containing SNPs is shown in Table 1. For sample PH207, with an SNP of T to A in the forward primer, the PCR efficiency (1.030) was higher than that of ND101 (0.994) without this SNP. For the cultivar F7 containing an SNP of G to T in the reverse primer, the PCR efficiency (1.055) was also obviously higher than that of ND101. In order to confirm that the higher PCR efficiency was, indeed, caused by the SNPs, new PCR primers (hmg-taq-F2/R2) and probe excluding the SNP sites were designed and used to test PCR efficiency. The PCR efficiency of hmg gene using the new primers and probe is shown in Table 1. Whether new primers or old primers were used, the amplification efficiency of ND101 (which had no SNP in the target region) did not change much, and all were close to 1.0 (0.994 with hmg-taq-F1/R1, 1.004 with hmg-taq-F2/R2). On the contrary, the new PCR efficiency was 0.989 in PH207 (with one SNP in the forward primer region) and 1.001 in F7 (with one SNP in the reverse primer region). The efficiency of both PCR assays was obviously decreased from that of qPCR assays using primers and probes containing mismatched SNPs (where PCR efficiency was mostly above 1.05). The results demonstrate that qPCR efficiency could be reduced to normal ranges when SNPs were eliminated from the primer/probe region, indicating the direct effect of SNPs on PCR amplified efficiency.

### 2.3. Development of Duplexed TaqMan Assay in 384-Well Format

In order to evaluate endogenous reference gene *hmg* and transgene *bar* against a uniform background, overcome the problem of suitable positive plant material, and avoid differences in DNA extraction, a standard reference molecule was constructed to include two target sequences in a single plasmid. The reference plasmid pCAMBIA3301-ZmHMG, containing *bar* and a 228 bp fragment of *hmg* gene (Figure 1B), was constructed, with sequences confirmed. After optimization of primer/probe concentrations and annealing temperatures, a highly sensitive TaqMan copy number assay for the accurate measurement of transgene *bar* copy numbers using plasmid pCAMBIA3301-ZmHMG as the reference molecule was established. Five dilutions of plasmid DNA (corresponding to 10^7^, 10^6^, 10^5^, 10^4^, and 10^3^ copies per 1 μL) were used to establish calibration curves for the exogenous *bar* and endogenous *hmg* gene. Two parameters, coefficient of determination (R^2^) and amplification efficiency (E), were employed to evaluate the standard curves of the primer pairs. The R^2^ values for *bar* and *hmg* TaqMan sets were 1.000 and 0.999, respectively (Figure 1C), indicating a high correlation between Ct value and copy numbers. The efficiency of the standard curve was 99.28% for *bar* and 100.03% for *hmg* (Figure 1C), indicating very high and similar efficiency in both reactions. These results demonstrate that the TaqMan copy number assay established in this study was adequately functional and accurate to calculate the transgene copy numbers of the unknown samples.

### 2.4. Reproducibility of TaqMan Copy Number Assay

To further validate the accuracy and stability of our quantification protocol, the standard curves were generated 10 times. Each test was performed in triplicate by a different operator on a different day and in a different machine. As shown in Table 2, the reproducibility of this TaqMan system was evaluated with five plasmid DNA dilutions (from 10^7^ to 10^3^ copies/μL). The inter-assay coefficient of variation (CV) values varied from 1.19 to 1.88%, and the standard deviation (SD) values ranged from 0.28 to 0.40 for the endogenous *hmg* (Table 2). The values for the transgene *bar* were CV = 1.18–2.94% and SD = 0.44–0.52. The CV and the SD values from these tests were relatively small, indicating that this duplexed TaqMan assay for *bar* and *hmg* worked stably and reliably in detecting templates of different concentrations.

### 2.5. Accuracy of TaqMan Copy Number Assay by Southern Blot and dPCR

In our developed TaqMan assay, the copy numbers of transgene *bar* of T_0_ transgenic plants were estimated by the Pfaffl method (Supplementary Table S2), which takes into account the amplification efficiency of the transgenic *bar* and reference gene *hmg*. A transgenic line was selected with a single copy of the transgene confirmed by Southern analysis as the positive control (calibrator). The non-transformed plant (WT) and the “no-template” control were used as negative controls, and no detectable background signals were detected in these samples. Forty-five transgenic maize samples were tested, and the results indicated that two samples had zero copies, 33 samples had one copy, six samples had two copies, and four samples had three or more copies of the *bar* gene (Supplementary Table S2). Two non-transgenic lines (12030068 and 12030084) were identified without ambiguity, indicating that the TaqMan assay developed here could clearly distinguish escapes from transformants.

**Figure 2:**
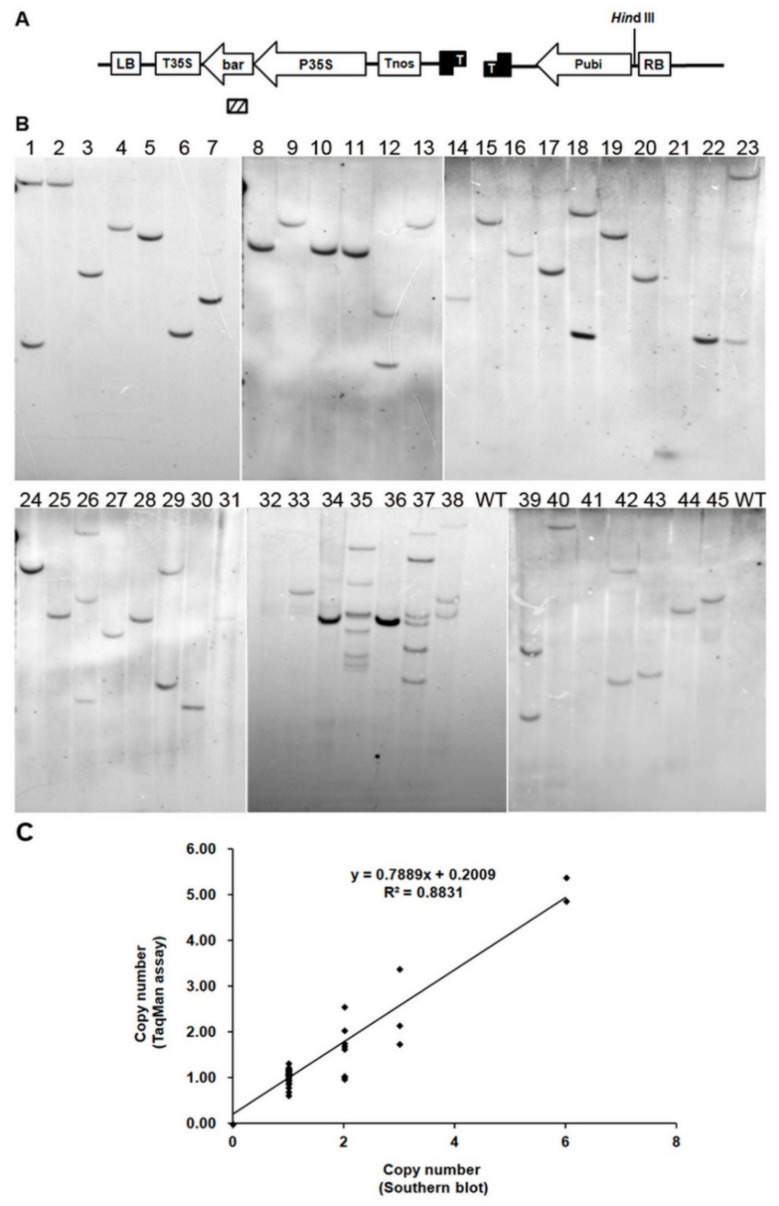
Correlation of TaqMan copy number assay data with Southern blot analysis. (**A**) Graphic representation of putative transgene insertion fragment (not to scale). pPBCXUN, binary vector construct in *Agrobacterium* strain EHA105 for *Agrobacterium*-mediated transformation; RB, right border; Pubi, ubiquitin promoter; Tnos, nopaline synthase terminator; P35S, 2 × CaMV 35S promoter; *bar*, phospinothricin acetyl transferase gene; T35S, CaMV 35S polyA; LB, left border. Position of *Hin*dIII restriction site is indicated above the T-DNA insert. Fragment used to generate *bar*-specific probes is indicated by the hatched box below *bar* gene. (**B**) Southern blot analysis of 45 independent T_0_ transgenic maize plants. DNA samples from 45 T_0_ events, as determined by TaqMan copy number assay, were digested with *Hin*dIII, fractionated by agarose gel electrophoresis, blotted to nylon, and probed with a *bar*-specific probe. Lines 1–45 correspond to 12141320, 12141321, 12151398, 12161497, 12161498, 12171579, 13181646, 13181668, 13191758, 13191764, 13191765, 13201829, 13201830, 12030065, 12050430, 12060607, 12060608, 12060612, 12060614, 12070775, 12080815, 12141331, 12161432, 12030070, 12040218, 12040220, 12050433, 12060625, 12060631, 12101014, 12111088, 12030068, 12030212, 12030213, 12030071, 12030210, 12030066, 12030067, 12010004, 12010005, 12030084, 12030085, 12030086, 12040234, and 12040236; WT represents untransformed ND101. (**C**) Correlation between copy numbers in transgenic maize T_0_ lines determined by TaqMan assay and Southern blot analysis.

The accuracy of the TaqMan assay developed here was assessed in two ways. First, Southern blot analysis was carried out to confirm the reliability of the results. All transgenic plants tested with the TaqMan assay were used for Southern blot analysis, in which maize genomic DNA was digested with *Hin*dIII and hybridized with the 484 bp DNA fragment of the *bar* gene (Figure 2A). Since *Hin*dIII did not restrict within the cassette of *bar* gene in the construct used for transformation, the number of bands observed after hybridization with *bar* probe reflects the number of copies inserted into the maize genome. We performed a correlation analysis between TaqMan and Southern blot data, and obtained a high correlation coefficient (R^2^ = 0.8831) (Figure 2C), indicating a strong relationship between the copy numbers determined by TaqMan assay and the copy/insert numbers determined by Southern blot analysis (Figure 2B). A high ratio of consistency (91%) was observed between the TaqMan and Southern blot analysis in line with one copy number (Figure 2B and Table 3). Different results were mainly found in cases with high transgene copy numbers; the five noncorrelative samples were overestimated by Southern blot, and one sample was underestimated as two bands by Southern blot but displayed three copies when analyzed by TaqMan assay (Table 3).

To further identify the level of accuracy of the TaqMan assay, eight of these lines were randomly selected for further evaluation of the transgene copy number using dPCR. The established dPCR assay successfully produced a clear distinction between positive and negative partitions in the two-dimensional analysis (data not shown), with minor modifications to the qPCR assay. The copy numbers determined by dPCR corresponded completely to the copy number estimates by TaqMan analyses (Supplementary Table S3), providing resolution of uncertainty and discrepancy in copy number estimates by Southern blot analysis (e.g., lines 13181646, 13201830, 12060612, and 12040220). TaqMan qPCR and dPCR appeared to be equally good at quantifying three or fewer copies of the transgene *bar* (Table 3).

Overall, these results demonstrate that the duplexed TaqMan qPCR analysis allows us to distinguish between lines that have one, two, or three copies of the transgene *bar*. This result also shows that this approach could be used to identify homozygous T_1_ lines, as it can accurately identify individuals that carry one or two transgene copies.

### 2.6. Feasibility of One Technical Repetition of TaqMan Assay

Given that our TaqMan results are highly reproducible and consistent with dPCR, we further evaluated the feasibility of one technical repetition of the TaqMan assay to increase detection throughput. Random sample analysis was performed on 4409 independent T_0_ events detected by TaqMan in three technical repetitions. Assuming that the three technical repetitions were 100% correct, for one-copy plants, the reliability of one and two repetitions reached 96.73 and 98.20%, respectively. Similar results were observed for two-copy plants: the reliability of one and two repetitions reached 89.32 and 94.31%, respectively (Table 4). This result indicated that the developed TaqMan assay was highly accurate in one repetition; therefore, we implemented the assay on large numbers of samples in only one repetition.

To further evaluate the validity of the copy numbers of transgene *bar* determined by the TaqMan assay performing only one technical replicate, we followed 2016 T_0_ transformants with a single one copy over two generations. After T_0_ self-mating, the copy numbers of the T_1_ progeny plants were estimated by the TaqMan assay, and these plants were expected to have a segregation pattern of 1:2:1 homozygous/heterozygous/non-transgenic. Thus, homozygous transgenic plants with two copies could be easily distinguished in the T_l_ generation. The prediction of homozygous T_1_ plants was verified by performing TaqMan assay with the T_2_ generation. If all T_2_ plants from selfing of predicted T_1_ homozygous plants had two copies, the T_1_ plants were considered homozygous. The copy number and separation ratio of T_1_ and T_2_ plants of each line were analyzed, and the results showed that 1931 plants had one copy, 74 plants had two copies, and 11 plants had three or more copies of the transgene (Table 5). Consistent with the results of random sampling analysis, the real rate reached as high as 95.8%, further showing that the single-copy number of the transgene detected by the TaqMan assay was accurate and reliable, even if only one replicate was performed.

### 2.7. Integrated High-Throughput Platform for Performing DNA Extraction, TaqMan Assay, and Data Analysis

**Figure 3:**
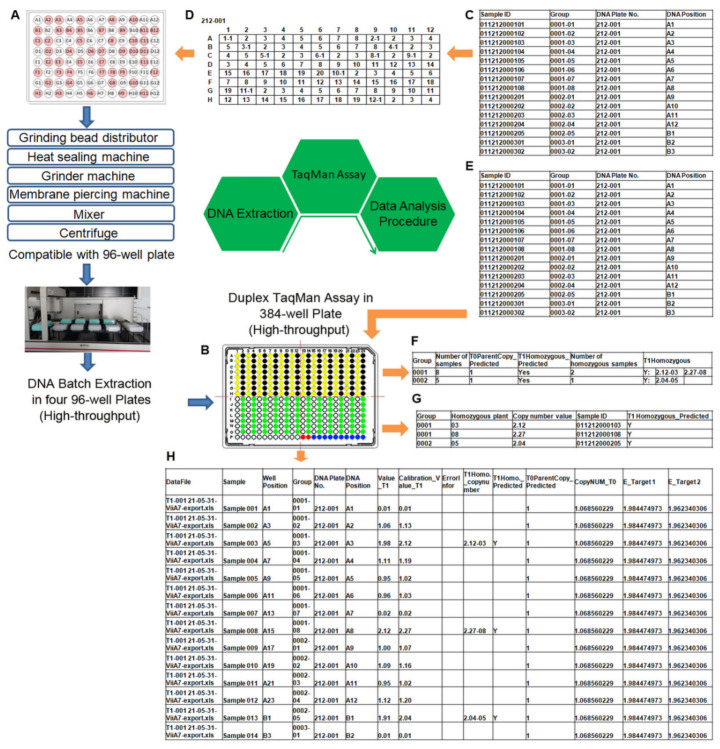
Development of TaqMan assay for high-throughput screening of transgene copy number and zygosity. (**A**) DNA extraction of samples in four 96-well plates and (**B**) TaqMan assay in 384-well plate are indicated by blue arrows. (**C**–**H**) Data analysis procedures are indicated by orange arrows: (**C**,**D**) sample corresponding to every well of 96-well plates; (**E**) sample corresponding to every well of 384-well plates; (**F**–**H**) automated data analysis results presented in three formats, (**F**) zygosity results of every line, (**G**) list of homozygous individual plants that need to be kept, and (**H**) copy number of individual T_1_ plants.

Up to this point, the duplexed TaqMan assay in a 384-well plate with one technical repeat developed here offered a fourfold increase in throughput, but a high-throughput method for isolating DNA from plants was necessary. A rapid and high-throughput method for extracting high-quality and intact DNA was developed using magnetic beads on a Beckman automated liquid handler (Figure 3A). The procedure was designed and optimized to extract four plates of 96-well DNA at the same time and allow one user to easily extract 28 plates of 96-well DNA within 8 h. To avoid cross-contamination between wells during extraction, the plates were sealed with aluminum foil, which was then pierced by a designed membrane-piercing machine instead of being peeled off.

We imagined that the developed high-throughput 384-well TaqMan assay (Figure 3B) would generate huge amounts of data. To handle any potential bottleneck, we developed a program for T_0_ copy number calculation and T_1_ zygosity determination using the Python language and the xlrd, xlwt, and numpy packages in Python (see Section 4). T_0_ and T_1_ analysis is a comprehensive data analysis tool for qPCR data of thousands of samples (Figure 3C–H). It included matching samples with 96-well plates in DNA extraction (Figure 3C,D), matching four 96-well plates with a 384-well plate in the TaqMan assay (Figure 3E), data analysis, data quality error message prompt, and data export (Figure 3F–H). For T_0_ samples, we calculated the copy number according to Formula (1) based on the PCR results. For T_1_ samples, we predicted the zygosity of T_1_ according to the copy number of every T_1_ plant. With this software, we can analyze T_0_ and T_1_ samples in batches instantly, no matter how many samples there are.

## 3. Discussion

### 3.1. Development of High-Throughput Copy Number Assay Using a “Universal” Maize Reference Gene System

#### 3.1.1. Allelic Variation of *hmg* Gene among Maize Cultivars

The use of an appropriate endogenous reference gene is indispensable to make the copy number assay more precise and reliable. An ideal endogenous reference gene should be species-specific, have a single copy, and have low heterogeneity among different cultivars. Furthermore, the endogenous reference gene used to calculate copy numbers in a sample has to amplify uniformly along a large number of commercially available varieties. For maize, at least five taxon-specific endogenous control reference genes have been developed, including alcohol dehydrogenase-1 (ADH), high-mobility group a (HMGa), invertase-1 (IVR), zein, and starch-synthase-IIb (SSII-b). We chose the maize *hmg* as the endogenous reference gene because it was previously determined to be the most suitable endogenous control gene with consistent and predictable amplification across maize cultivars [28,29,38]. Papazova et al. [28] reported that *hmg* showed low variation in quantified copy numbers (coefficient of variation) among 84 maize varieties. However, for the following reasons, variations in the *hmg* assay performance among the varieties might be expected when the assay has to be applied to analyze unknown samples. First, maize has great genetic diversity, with many SNPs in the genome among the different cultivars [32,33,34,35,36,37]. Indeed, studies on the SNPs present in 18 maize genes in 36 inbred lines have shown the high rate of nucleotide variation in maize: 1 polymorphism per 31 bp in noncoding regions and 1 per 124 bp in coding regions [34]. Second, more transgenic maize varieties from different inbred lines are in the pipeline for commercialization. To ensure the consistency of *hmg* in qPCR assays, we analyzed its target sequences using 633 diverse maize varieties, including the lines described in a previous study [38]. These lines represent an extensive collection of the most advanced publicly available maize inbred lines. Sequence alignment of target DNA from 633 maize lines in this study revealed that an identical SNP of T > A was present in 112 maize cultivars (17.7%) at the position of the first nucleotide from the 5′ end of the forward primer, and an SNP of G > T was found in three maize cultivars (F7, DH40, and SS99; 0.03%) at the position of the second nucleotide from the 5′ end of the reverse primer, but no SNP was found at the position of the probe among all tested lines.

It was previously reported that SNPs in qPCR primer/probe annealing regions could affect PCR efficiency in the quantification of genetically modified contents, viruses, and micro-organisms [29,30,31,39,40]. In our study, increased PCR efficiency was observed in *hmg* assays when lines with SNPs in the primer region were tested. As shown in Table 1, the PCR efficiency of *hmg* gene in PH207 (with one SNP in the forward primer region) and F7 (with one SNP in the reverse primer region) was 1.030 and 1.055, respectively; the PCR efficiency of *hmg* gene in ND101 (with no SNP in the primer region) was 0.994 (Table 1). The PCR efficiency of *hmg* gene in lines with SNPs was obviously higher in assays than that in lines containing no SNPs. Since the presence of SNPs in either the forward or reverse primer would increase the amplification efficiency, we assumed that the excessive amplification efficiency may result from the mismatch of primer sequences to the genome sequence in the target regions. The effects of SNPs on PCR efficiency were further confirmed by new qPCR assays employing redesigned homogeneous primers with SNPs eliminated. When the new primer/probe sets adjusted for SNPs in some lines were used, the PCR efficiency decreased (Table 1), indicating that the mismatched SNPs caused the increased qPCR efficiency. These results demonstrated that the SNPs could increase qPCR efficiency, which would result in overestimation of genetically modified contents. Similarly, the SNPs found in the primer region of maize *adh1* by qPCR assay greatly decreased the efficiency of quantitative PCR analysis [29]. Thus, considering both the absence of SNPs in the target sequence and the high PCR performance among different maize lines, we concluded that hmg-taq-F2/R2, targeting the *hmg* region, appeared to be most useful in establishing an accurate and creditable quantitative PCR assay for transgenic maize around the world.

#### 3.1.2. Development of Duplexed TaqMan Assay in 384-Well Format for High-Throughput Copy-Number Screening

We developed a high-throughput duplexed TaqMan assay, performed in a 384-well format with very small reaction volumes, to precisely and rapidly identify the copy numbers of thousands of maize transgenic lines. The R^2^ values for *bar* and *hmg* TaqMan sets are 1.000 and 0.999, respectively (Figure 1C), indicating that there was a high correlation between Ct value and copy numbers. The efficiency of the standard curve was 99.28% for *bar* and 100.03% for *hmg* (Figure 1C), indicating very high and similar efficiency in both reactions. Inter-assay reproducibility determined under optimized conditions in the 384-well plate was also satisfactory in this high-throughput assay. The coefficient of variation (CV) values for *bar* and *hmg* genes varied from 1.18 to 2.94% and the standard deviation (SD) values were between 0.28 and 0.52 (Table 2). These slight variations across the experiments indicate that the TaqMan system functioned stably and reliably.

### 3.2. High Accuracy of TaqMan Copy Number Assay without Technical Repeats, Comparable to dPCR

#### 3.2.1. High Accuracy of Copy Number Determination by TaqMan Assay Is the Same as dPCR, Higher Than Southern Blot Analysis

The number of transgene copies has traditionally been estimated by Southern blot analysis, which is costly in terms of reagents, labor, skill, and time, and also requires a considerable amount of high-quality DNA from fresh or frozen material. To confirm the transgene copy numbers derived from the TaqMan assay, 45 transgenic maize lines from 45 transformants were randomly chosen for TaqMan assay and Southern blot analysis. The agreement between qPCR and Southern blot results was approximately 88%. For five lines (13181646, 13201829, 13201830, 12040220, and 12030067), the TaqMan assay estimates were lower than the Southern blot estimates. This discrepancy may be due to incomplete insertion or partial digests. In line 12060612, three copies were estimated by the TaqMan assay, but only two copies by Southern blot analysis. There are several reasons for this underestimation by Southern blot, including the insertion of more than one *bar* copy at a single locus that was tandemly inverted and the generation of DNA fragments of very similar sizes that were not resolved on the gels. In contrast, the qPCR (TaqMan) assay should be able to detect all copies of a gene, except when rearrangements happen to disrupt a primer binding site [41].

To assess the discrepancy in copy numbers estimated by Southern blot and TaqMan assay, a duplexed dPCR was developed in this study as a comparison. dPCR is a third-generation technology based on subdividing the analytical sample into numerous partitions that are amplified individually. The main advantage of dPCR is that it provides absolute quantification of the number of the target sequences in a sample without certified reference material and a standard curve. Our study shows that the TaqMan assay and dPCR both performed equally well at quantifying low levels of the transgene *bar*. Similarly, several reports have shown that dPCR and TaqMan assay performed well at detecting one-fold differences [22,42]. These results demonstrate not only that TaqMan assay is a more reliable technique than Southern blot for estimating transgene copy numbers, but also that it can confidently identify maize lines that have one, two, or three copies of the transgene *bar*, comparable to dPCR.

In transformation projects, the goal is to rapidly select low or single copy number events on a large scale. Although highly reliable, dPCR is cost-intensive and time-consuming, making it impractical to use on a large scale for early determination of copy number and zygosity. Compared with Southern blot analysis and dPCR, TaqMan assay is amenable to being scaled up such that large numbers of events can be screened and events with high copy numbers can be discarded very early in the transformation process, thereby allowing resources to be focused only on those events with low copy numbers. Furthermore, the results generated in this study provide a strong case that the TaqMan assay had equally good accuracy in measuring three or fewer copies of the transgene *bar* compared with dPCR. Therefore, from a practical perspective, our TaqMan assay is an accurate, rapid, and low-cost analytical method to select low or single copy transformation events and to identify hemizygous and homozygous individuals in a large number of samples.

#### 3.2.2. High Accuracy of TaqMan Assay Achieved without Technical Repeats

In basic research, triplicates are commonly selected as the number of replicates. Indeed, technical replicates offer a number of benefits, such as providing an estimate of system precision, improving experimental variation, and allowing for potential outlier detection and removal. On the other hand, technical replicates add cost and reduce the throughput. From a practical perspective, the number of technical replicates should be constrained, especially when large numbers of plants need to be screened. The higher precision observed for the TaqMan assay developed here could allow the number of technical replicates to be reduced, so we performed a simple random sampling analysis to decide on an optimal number of technical replicates, balancing benefits vs. costs. Assuming that the results of three technical repetitions are 100% correct, the reliability of one replicate reached 96.7 and 89.3% for one-copy and two-copy plants, respectively, and the reliability of two replicates reached 98.2 and 94.3%, respectively. Although no technical repeats were performed, the accuracy rate of one copy number reached 96.7%. From these results, it can also be concluded that, when the number of repetitions increases, the reliability of the experiment improves, but this increase was quite small, especially for single-copy plants. For further confirmation, 2016 T_0_ transformants with a single one copy determined by TaqMan assay with one technical repetition were followed over two generations to analyze the copy numbers and separation ratios of T_1_ and T_2_ plants. For one copy, the reliability of one replicate reached as high as 95.8%. Therefore, we decided to perform only one technical repetition for the TaqMan assay, which would reduce the cost by two thirds and increase throughput by two thirds.

### 3.3. High-Throughput Analysis of Transgene Copy Number and Zygosity

To develop a successful high-throughput TaqMan assay, we made the following optimizations:

1. A suitable range for genomic DNA was 10–400 ng. The multiple time-consuming and laborious manipulation steps in the preparation of high-quality genomic DNA from thousands of samples still remains a primary limitation when thousands of samples need to be analyzed rapidly, such as in molecular plant breeding and plant transformation programs. In addition, having high-quality and intact genomic DNA is an important prerequisite for the accurate measurement of transgene copy numbers. The use of poor-quality genomic DNA produces qPCR results that are not accurate [43]. To overcome these problems, we developed a rapid and high-throughput method for the extraction of high-quality and intact DNA using magnetic beads on a Beckman automated liquid handler (Biomek FX^p^). The procedure described here was designed and optimized to extract four plates of 96-well DNA at the same time and allow one user to easily extract 28 plates of 96-well DNA within 8 h (see Section 4). Most importantly, the resulting DNA is of excellent quality and suitable for use in transgene copy number and zygosity analysis by the TaqMan assay.
2. The uniformity of the endogenous reference gene was our main concern when we were preparing to develop the TaqMan assay for transgene copy numbers. In this study, one previously reported qPCR assay on maize endogenous reference gene *hmg* was comprehensively evaluated for target gene sequence variations among 633 maize inbred lines and qPCR performance. Two SNPs were observed in the *hmg* gene, and these SNPs significantly increased the efficiency of qPCR amplification. The new TaqMan assay employs redesigned homogeneous primers and probe, eliminating SNPs, and has been validated to be most useful in establishing accurate and creditable quantitative PCR analysis of transgenic maize.
3. A standard reference plasmid was developed to include two target sequences in a single plasmid. In this way, a serial dilution of the dual-target plasmid DNA with correct concentration could be made to obtain accurate standard curves for the exogenous *bar* and endogenous *hmg* genes, which is a key prerequisite for quantitative analysis. The strengths of the developed pCAMBIA3301-ZmHMG plasmid are its complete characterization of sequence and structure, high performance, ease of preparation, stability in storage, and low cost. It was previously shown that the use of cloned plasmid GMO target sequences for calibration can produce accurate quantitative results [44,45,46,47].
4. A transgenic T_0_ plant with one copy of the transgene demonstrated by dPCR was applied as an inter-run calibrator for normalization. Inter-run calibration is required because there is a dependent relationship between quantification cycle value and relative quantity due to instrument-related variation (PCR block, lamp, filters, detectors, and so on), data analysis settings (baseline correction and threshold), reagents (polymerase, fluorophores, and so on), and the optical properties of plastics. Therefore, inter-run calibration is required to correct for possible run-to-run variation whenever all samples are not analyzed in the same run [48].
5. Duplexed reactions were used, as they are more efficient and economical, appropriate for high throughput, and less prone to technical errors than two independent reactions. They are especially valuable for quantitative experiments that require a higher degree of precision, such as copy number assays. It is important to note that the concentrations of primers and probes of the target bar and reference hmg genes should be optimized to avoid preferential amplification of one of the two, because amplification of both genes was carried out in the same reaction tube.
6. The duplexed TaqMan assay was performed in 384-well plates with a volume of 10 µL, providing an eightfold increase in throughput over the conventional uniplex 96-well format assay, and a 16-fold reduction in cost. Although there is an ultra-high-throughput fluorescent quantitative PCR system with a 1536-well block currently on the market, it has to be equipped with an automatic liquid dispensing system to pipette with ultra-micro (0.5–2 μL) volume, preventing its application in most testing and research laboratories. In our lab, with proper training, proper tools (for example, a 12-well multichannel pipette), and a good experimental protocol, any conscientious staff member can pipette reagents into 384-well plates and generate consistent high-quality data without the need for robotics or other automated liquid dispensing systems.
7. The transgene copy numbers were estimated by the efficiency-corrected method described by Pfaffl [49]. The comparative 2^-ΔΔCt^ method, which is the most widely used method for copy number determination by qPCR, has an often untrue assumption that both the transgene and reference gene have approximately equal and near 100% efficiency, a condition that is rarely met in practice [8,50]. The Pfaffl method includes the different levels of efficiency of the two genes in the equation, and, therefore, the difference in efficiency between the two genes will be accounted for in the calculation [49]. Considering that there are differences in amplification efficiency of the transgene and the reference gene in most situations, the Pfaffl method was adapted to estimate transgene copy numbers in the present study.
8. Once the above points were satisfactorily met, the accuracy of the TaqMan assay could be guaranteed, and, on this basis, only one technical repetition needed to be performed. We performed a simple random sampling analysis on the copy number results of 4409 transgenic plants, and the results showed that the accuracy of one repetition could reach 96.7% of that of three repetitions of single-copy plants. For further confirmation, the copy numbers and separation ratios of T_1_ and T_2_ plants of 2016 T_0_ transformants, which have a single copy determined with one technical repetition, were analyzed. For one copy, the reliability of one replicate reached as high as 95.8%. To the best of our knowledge, this is the first demonstration that the TaqMan copy number assay can have comparable accuracy to dPCR without technical repeats. The application of this approach would reduce the cost by two thirds and increase throughput by two thirds compared with three repetitions. In addition, this approach would make it easier to arrange four 96-well samples in a 384-well qPCR plate and to analyze large amounts of data.
9. Accurate analysis of large amounts of data was another feature required by high-throughput fluorescence qPCR technology. In addition to the software that comes with the instrument, which can analyze fluorescence quantitative PCR data, we developed data analysis software to process high-throughput data within a few minutes without being limited by the amount of data.

## 4. Materials and Methods

### 4.1. Plant Material

The natural maize population used in this study was composed of 633 temperate inbred lines, including those of well-known heterotic Reid (B73, Zheng58), Lancaster (Mo17), Iodent (PH207), Tang Sipingtou (Chang7–2), Lüda Red Cob (Dan340), and P (P178) groups.

Transgenic maize plants from the inbred ND101 line were created by the Center for Crop Functional Genomics and Molecular Breeding of China Agricultural University. *Agrobacterium tumefaciens* strain EHA105, containing the binary plasmid pBCXUN [51], was used for maize transformation. The plasmid contains the *bar* selectable marker gene driven by the CaMV 35S promoter. Independent transgenic T_0_ plants were obtained and used to assess the transgene copy number. T_1_ plants were derived from selfing of T_0_ single-copy plants and were used to assess zygosity. T_2_ plants were from selfing of T_1_ single-copy homozygous plants. T_1_ and T_2_ plants were sprayed with glufosinate to eliminate non-transgenic plants. Transgenic plants were grown in a greenhouse under a 14 h/10 h light/dark cycle.

### 4.2. High-Throughput Genomic DNA Extraction Method

For high-throughput extraction of genomic DNA for qualitative and copy number assays, an automated method was developed using the magnetic-bead-based DNA extraction kit (GeneOn Biotech, GO-GPLS-400, Changchun, China) on the Beckman automated liquid handler (Biomek FX^p^, Indiannapolis, IN, USA). The automated workflow was designed and optimized as follows: after collecting leaf samples (4 mg fresh mass, 5 mm diameter) in wells of a 2.2 mL 96-well plate (YaTong, ACDP22-SU-9, Suzhou, China), 5 mm stainless steel grinding balls were distributed in the plate within 1 s by a plate grinding bead distributor (GeneOn Biotech, GO-GBDR-005, Changchun, China), then the plates were placed into a −20 °C freezer for longer than 20 min and sealed with aluminum foil (YaTong, ACWP-SF-B, Suzhou, China) using a heat sealing machine (VITL VTS, S120499, Liverpool, UK). The sealed plates containing leaf tissue and a metal ball were immersed in liquid nitrogen for 30 s, and then pulverized in a grinder machine (Retch, MM400, Dusseldorf, German) for 12 s at a rate of 24/s. To avoid cross-contamination between wells during extraction, the plates were centrifuged briefly to consolidate tissue after shaking, and the sealing foil was then pierced by a membrane piercing machine (YaTong, ACPM-96-V, Suzhou, China) instead of being peeled off the plates.

First, 200 μL of preheated (60 °C) lysis buffer was added to each sample by a 12-well multichannel pipette, and then the plates were incubated for 10 min in a 60 °C water bath with occasional mixing. Then, 67 μL of extraction buffer was added and the plates were sealed with PCR film. The plates were mixed by a 96-well plate mixer (Qilinbeier, BE-3100, Haimen, China) for 15 s at 900 rpm, and spun in a centrifuge (Eppendorf, 5810R, Hamburg, German) equipped with rectangular buckets for 15 min at 4000 rpm (4 °C), after which 150 μL of supernatant was removed from each well by pipetting and transferred to a clean 2.2 mL 96-well plate. Next, the supernatant was processed on the Beckman automated liquid handler (Biomek, FX^p^, Indiannapolis, IN, USA). After 15 μL of magnetic beads and 165 μL of binding buffer were added to the supernatant, the plates were vortexed by a single-plate vortex oscillation module (Biomek Orbital Shaker ALP, Indiannapolis, IN, USA) and incubated for 2 min at room temperature. The plates were placed onto supermagnetic racks (Biomek, Agencourt SPRI, Indiannapolis, IN, USA) for 1 min at room temperature to separate the DNA-bound magnetic beads from the lysate. The supernatant was discarded, and 300 μL of wash buffer was added to each well; the beads were resuspended, and the plates were placed back onto the magnetic racks for 1 min. The supernatant was discarded, and the washing procedure was repeated using 80% ethanol. After ethanol was removed, the plates were air-dried. Then, 120 μL of ddH_2_O was mixed with the DNA-bound magnetic beads and the mixture was incubated for 1 min at room temperature for DNA elution. The plates were then placed on the magnetic racks and the beads were separated from the eluate. The eluate contained purified DNA. The expected yield was 10 μg of genomic DNA from normal healthy leaf tissue. The concentration of DNA was determined by using a UV spectrophotometer (NanoDrop 1000, Wilmington, DE, USA) at 260 nm, and the quality of DNA was evaluated from the 260/280 and 260/230 nm UV absorption ratios. Genomic DNA concentration was adjusted to 50 ng/μL for subsequent qPCR analysis. All DNA samples were stored at −20 °C.

### 4.3. Sequencing and Alignment of Endogenous Reference Gene Target Region

Target DNA fragments of *hmg* in 633 maize inbred lines were amplified by PCR amplification employing the sequencing primers (Supplementary Table S6) and KOD -Plus- Neo DNA polymerase (TOYOBO, KOD-401, Osaka, Japan). The PCR mixture contained the following reagents: 1 × PCR buffer for KOD -Plus- Neo, 0.2 mM dNTP, 1.5 mM Mg^2+^, 0.3 μM of each primer, 2% DMSO, 1 unit KOD -Plus- Neo DNA polymerase, and 20 ng of each DNA sample, at a final volume of 50 μL. PCR amplification was performed under touchdown conditions as follows: initial denaturation at 94 °C for 3 min, 8 cycles of denaturation at 98 °C for 20 s, annealing at 68 °C for 30 s (temperature was decreased by 1 °C per cycle), and extension at 68 °C for 40 s, followed by 26 cycles at 98 °C for 20 s, 60 °C for 30 s, and 68 °C for 40 s, and a final extension at 68 °C for 5 min. Each PCR product was analyzed using 1% agarose gel electrophoresis to verify the success of the PCR amplification and the size of amplicons. The amplified DNA fragments were sequenced by HuaDa Gene (Beijing, China). The obtained DNA sequences were aligned using the ClustalW tool of BioEdit7 to reveal SNPs within the target regions in the TaqMan assays.

### 4.4. Construction of Standard Plasmid as Reference Molecule

In the plant expression vector pCAMBIA3301, the plant selectable marker gene *bar* is driven by the 35S promoter. Therefore, pCAMBIA3301 was used as the backbone to construct pCAMBIA3301-ZmHMG. The endogenous reference control *hmg* gene fragment (228 bp) was amplified using ZmHMG-EcoR I-F/ZmHMG-Hind III-R as primers and DNA from non-transgenic maize line zheng58 as a template. The PCR amplification program was the same as above. The amplification product was inserted between the *Eco*R I and *Hin*dIII sites of pCAMBIA3301 to produce the recombinant plasmid pCAMBIA3301-ZmHMG, which was used as a standard plasmid for *bar* and *hmg*. The recombinant plasmid was transformed into *Escherichia coli* DH-5α cells and purified using a Plasmid Mini Extraction Kit (Axygen, AP-MN-P-250, Union, CA, USA). The positive plasmid was confirmed using restriction enzyme digestion and PCR, and further confirmed by sequencing at HuaDa Gene (Beijing, China).

### 4.5. Duplexed TaqMan Assay

#### 4.5.1. Primers and Probes

Primers and probes for the transgene were described in a previous report [52], designed specifically for the selectable marker *bar* gene (GenBank accession no. X17220). For the endogenous control, two sets of primers were used for qPCR analysis of *hmg* gene (GenBank accession no. AJ131373). The first set of primers was described in a previous report [53]. The second set contained redesigned primers based on the sequencing results of each amplified target DNA fragment to match the observed SNPs. Two reactions were performed with the same probe. TaqMan probes were labeled at their 5′ end with fluorescein (FAM) and VIC as reporter fluorophores for *bar* and *hmg*, respectively, and at their 3′ end with black hole quencher (BQ1) as the quencher fluorophore. All primers and probes were synthesized by Invitrogen (Guangzhou, China) and are listed in Supplementary Table S6.

#### 4.5.2. Optimization of TaqMan Primer Concentrations

All qPCR assays were carried out in a fast ViiA7 real-time PCR System (Applied Biosystems, Foster, CA, USA) in 384-well plate microtubes using a TaqMan system at a final volume of 10 μL. The concentrations of primers were optimized in preliminary experiments by testing 16 different combinations (300, 500, 700, and 900 nM for each primer). Concentrations of 500 nM *hmg* gene primers and 700 nM *bar* gene primers were found to be optimal in achieving high and comparable PCR efficiency for both genes (data not shown). For the probes, an optimal concentration of 300 nM was determined.

The optimal annealing/extension temperature was chosen as 60 °C from various annealing temperatures (55–62 °C). Optimized PCR conditions were transferred according to the following protocol.

#### 4.5.3. Duplexed TaqMan qPCR Reaction

Optimized qPCR reactions were performed at 10 µL volume in a 384-well plate. *bar* and *hmg* were amplified simultaneously using a TaqMan system in the same tube. The reaction mixture contained 4 µL of LightCycler 480 Probes Master Mix (2×; Roche, 4887301001, Indianapolis, IN, USA), 0.8 µL DNA (≈50 ng), and an optimal concentration of each transgene-specific primer and probe (700 nM bar-taq-F/R primers, 500 nM hmg-taq-F2/R2 primers, and 300 nM probe). The PCR was run in the fast ViiA7 real-time PCR system (Applied Biosystems, Foster, CA, USA) using the following program: initial denaturation at 95 °C for 10 min, 40 cycles of 10 s at 95 °C and 30 s at 60 °C, and fluorescence was collected at the 60 °C annealing extension step. To monitor potential cross-contamination during PCR setup, a no-template control (NTC) and negative control (non-transgenic plant DNA) were added to a well-containing reaction mixture as a negative control. An inter-run calibrator (single-copy sample) was used for normalization of all probes within a run and between runs. The Ct value was determined by using the instrument’s software and manually adjusted as necessary.

#### 4.5.4. Establishment of Standard Curve

To determine the PCR efficiency for the transgene (*bar*) and endogenous gene (*hmg*), pCAMBIA3301-ZmHMG plasmid DNA solution was serially diluted to final concentrations of 10^7^, 10^6^, 10^5^, 10^4^, and 10^3^ copies/μL and subjected to TaqMan assay in triplicate. Standard curves were constructed by plotting the Ct values against the logarithm of the plasmid DNA copy number. The efficiency of amplification was calculated based on the slopes of standard curves with the formula given in [54]. The hallmarks for a well-optimized qPCR assay include linear standard curve (R^2^ > 0.980), high efficiency (90–105%), and consistency across replicate reactions.

#### 4.5.5. Calculation of Copy Number by Pfaffl Method

Copy number was calculated by the Pfaffl formula [49]:

Copy number = 2 × (E_target_^ΔCt, target (calibrator- test)^)/(E_ref_ ^ΔCt, ref (calibrator–test)^) (1)

where target represents *bar* gene, ref represents endogenous *hmg* gene, and E is the amplification efficiency.

#### 4.5.6. Evaluation of the Reproducibility of the TaqMan Assay

Five 10-fold serial dilutions of a standard plasmid (1.0 × 10^7^ to 1.0 × 10^3^ copies/μL) were used to assess inter-assay reproducibility. For this process, each dilution was analyzed in 10 independent reactions on different days. The coefficient of variation (CV = SD/mean × 100) of the inter-assay was assessed to determine the reproducibility of the TaqMan assay.

### 4.6. Southern Blot Analysis

Genomic DNA for Southern blot was extracted from 2 g of fresh leaves according to the CTAB method. Fifty micrograms of genomic DNA from the transgenic event and the non-transgenic control were digested overnight with the *Hin*dIII restriction enzyme (NEB, R0104L, Ipswich, MA, USA). Digestion was followed by ethanol precipitation. Then, 25 µg of digested DNA was electrophoresed on 0.9% *w/v* agarose gel and transferred onto Hybond-N+ membranes (Roche, 11417240001, Indianapolis, IN, USA) using a Model 785 Vacuum Blotter (Bio-Rad, Hercules, CA, USA). The DNA was permanently cross-linked to the membrane by exposure to UV light in an ultraviolet cross-linker (UVP CL-1000, Uplan, CA, USA) at a setting of 120 mJ. The *bar* amplified fragments (Supplementary Table S6) were labeled by a PCR DIG Probe Synthesis Kit (Roche, 11636090910, Indianapolis, IN, USA). Membranes were prehybridized at 42 °C for 2 h in DIG Easy Hyb solution (Roche, 11603558001, Indianapolis, IN, USA) in a hybridization incubator with gentle rotation. The probes were boiled for 5 min and placed on ice for 5 min. After prehybridization, the denatured DIG-labeled probe was added and allowed to hybridize for 20 h. The membranes were washed at low stringency in 2 × SSC 0.1% (*w/v*) SDS twice at room temperature for 5 min, and then twice at higher stringency in 0.5 × SSC 0.1% (*w/v*) SDS for 15 min each. Immunological detection of the probes was performed with a DIG Luminescent Detection Kit (Roche, 11363514910, Indianapolis, IN, USA) according to the manufacturer’s instructions. After incubation with CSPD^®^, membranes were placed in an autoradiography cassette and incubated at 37 °C for 15 min to enhance the exposure, and then exposed to Kodak BioMax MR film at room temperature. Each membrane was exposed to the film for various periods of time to allow for the correct exposure to be captured.

### 4.7. Duplexed Digital PCR Analysis

For a 20 µL reaction, the following were added: 10 µL 2 × master reaction mix, 500 nM *hmg* gene primers (hmg-taq-F2/R2), 700 nM *bar* gene primers (bar-taq-F/R), 300 nM of each dual-labeled probe, and 2.8 µL undigested genomic DNA. These were mixed well and spun briefly. Then 14.5 µL of the PCR mixture was loaded onto a QuantStudio™ 3D Digital PCR 20K Chip v2 (Applied Biosystems, Foster, CA, USA), the chip was covered with immersion fluid, a lid was applied, the assembly was filled with immersion fluid, and then the loading port was sealed according to the manufacturer’s instructions. The primers and probes used for dPCR were the same as those used for TaqMan assay.

PCR was performed using the Applied Biosystems^TM^ Dual Flat Block GeneAmp PCR System 9700 (Applied Biosystems, Foster, CA, USA) with the following conditions: 96 °C for 10 min; 60 °C for 2 min, and 98 °C for 30 s, for 39 cycles; 60 °C for 2 min; and 10 °C hold. Reading of the chip was performed using the QuantStudio™ 3D Digital PCR Chip Reader (Applied Biosystems, Foster, CA, USA). Data were analyzed using QuantStudio™ 3D Analysis Suite™ Cloud Software with default settings for threshold determination to distinguish positive and negative reactions.

### 4.8. Simple Random Sampling Analysis

From among the 4409 individuals, *n* individuals were randomly selected, and then selected according to the number of repetitions (r) for each individual (1, 2, 3, etc.). Repetitions (r) were randomly extracted from the original three repetitions, and the average of these repetitions (r) was calculated. The average of three technical repetitions was assumed as the true value, and the consistency ratio between the r averages of *n* individuals and the true value was determined. This analysis was performed using R language.

### 4.9. Data Analysis Program

We developed a program for T_0_ and T_1_ copy number calculation using Python language (https://www.python.org/, accessed on 16 October 2021) and the xlrd (https://pypi.org/project/xlrd/, accessed on 16 October 2021), xlwt (https://pypi.org/project/xlwt/, accessed on 16 October 2021), and numpy (https://numpy.org/, accessed on 16 October 2021) packages in Python. All Python packages used in the program could be installed using the Python pip command.

## 5. Conclusions

In conclusion, we successfully developed and tested a high-throughput method for rapidly estimating the transgene copy number and zygosity of transgenic maize plants with the required accuracy and precision. As part of this process, we developed a 96-well plate-based high-throughput DNA extraction method to meet the need for preparation of high-quality genomic DNA from thousands of samples. Importantly, we identified suitable reference gene primers among 633 maize inbred lines according to qPCR target DNA sequence variations and qPCR performance. It is worth mentioning that, based on the high accuracy rate of one copy number with one repetition, we performed only one technical repetition for the 384-well format TaqMan assay. In addition, we developed data analysis software to process the high-throughput data within a few minutes without being limited by the amount of data. The copy number assay of 28-plate 96-well samples from DNA extraction to give the result of transgene copy numbers could be carried out within 8 h by two persons. In conclusion, the assay described in this report fulfills the necessary and desirable criteria for copy number and zygosity assay because it is simple, robust, reproducible, useful for large-scale screening, time-effective, and cost-effective.

## Figures and Tables

**Table 1:** PCR efficiency and standard curve linearity of TaqMan assays in their corresponding calibrator lines.

Primers and Probe	Maize Cultivar	Linearity (R^2^)	PCR Efficiency (E)	Standard Curve
hmg-taq-F1/R1, hmg-taq-Probe	PH207 (T > A)	0.997	1.030	y = −3.251x + 33.984
	F7 (G > T)	0.998	1.056	y = −3.196x + 33.781
	ND101 (without SNPs)	0.995	0.995	y = −3.336x + 33.105
hmg-taq-F2/R2, hmg-taq-Probe	PH207 (T > A)	0.997	0.989	y = −3.350x + 33.634
	F7 (G > T)	0.997	1.001	y = −3.319x + 33.451
	ND101 (without SNPs)	0.996	1.004	y = −3.312x + 32.605

**Table 2:** Reproducibility of TaqMan assays for the *bar* and *hmg* genes.

Gene Name	Predicted Copy Number (Copies/μL)	Mean Ct Value	SD	CV (%)
*bar* gene	10^7^	16.13	0.48	2.94
	10^6^	19.47	0.46	2.35
	10^5^	22.81	0.44	1.94
	10^4^	26.23	0.45	1.71
	10^3^	29.64	0.52	1.18
*hmg* gene	10^7^	15.02	0.28	1.88
	10^6^	18.35	0.31	1.68
	10^5^	21.69	0.30	1.36
	10^4^	25.11	0.30	1.19
	10^3^	28.44	0.40	1.40

SD, standard deviation; CV, coefficient of variation.

**Table 3:** Correlation of TaqMan with Southern blot and dPCR assay for transgene copy determination.

Copy Number	Number of Samples Determined by TaqMan (% of total)	Correlation with Southern Blot		Correlation with dPCR	
		Number of Assayed Samples	Number of Consistent Samples (%)	Number of Assayed Samples	Number of Consistent Samples (%)
0	2 (4.4%)	2	2 (100.0%)		
1	33 (73.3%)	33	30 (90.9%)	5	5 (100.0%)
2	6 (13.3%)	6	4 (66.7%)	2	2 (100.0%)
>2	4 (9.0%)	4	1 (25.0%)	1	1 (100.0%)
Total	45	45		8	

**Table 4:** Prediction accuracy of different technical repetitions using random sample analysis (*n* = 4409).

Copy Number	Accuracy of Technical Repetitions		
	r = 1	r = 2	r = 3
1	0.9673	0.9820	1.0000 (assumed)
2	0.8932	0.9431	1.0000 (assumed)

**Table 5:** Real copy numbers of 2016 T_0_ samples with one technical repetition, verified by results of T_1_ and T_2_ progeny.

Copy Number	Number of T_0_ Samples Determined by TaqMan Assay	Real Copy Number of T_0_ Samples Verified by T_1_ and T_2_ Progeny
		Number of Consistent Samples (%)
1	2016	1931 (95.8%)
2		74
>2		11
Total	2016	2016

## Supplementary Materials

The following are available online at https://www.mdpi.com/article/10.3390/ijms222212487/s1.
