# Supplementary material for: High-Throughput and Accurate Determination of Transgene Copy Number and Zygosity in Transgenic Maize: From DNA Extraction to Data Analysis
Source: Int J Mol Sci. 2021 Nov 19;22(22):12487. doi: 10.3390/ijms222212487 (PMC8619409; doi:10.3390/ijms222212487)
Supplement: Supplementary file 1 [file ijms-22-12487-s001.zip › Table S1.pdf]

Table S1. The amplified target DNAs of *hmg* gene in 633 maize inbred lines.

**Lines**	**Sequence**
shen5005	TTGGACTAGAAATCTCGTGCTGATTAATTGTTTTACGCGTGCGTTTGTGTGGATTGTAGGACAAGGCTCCCTATGTAGC
R31	ATGGACTAGAAATCTCGTGCTGATTAATTGTTTTACGCGTGCGTTTGTGTGGATTGTAGGACAAGGCTCCCTATGTAGC
mei68113	TTGGACTAGAAATCTCGTGCTGATTAATTGTTTTACGCGTGCGTTTGTGTGGATTGTAGGACAAGGCTCCCTATGTAGC
qiong51	TTGGACTAGAAATCTCGTGCTGATTAATTGTTTTACGCGTGCGTTTGTGTGGATTGTAGGACAAGGCTCCCTATGTAGC
757	TTGGACTAGAAATCTCGTGCTGATTAATTGTTTTACGCGTGCGTTTGTGTGGATTGTAGGACAAGGCTCCCTATGTAGC
DH138	ATGGACTAGAAATCTCGTGCTGATTAATTGTTTTACGCGTGCGTTTGTGTGGATTGTAGGACAAGGCTCCCTATGTAGC
R08	TTGGACTAGAAATCTCGTGCTGATTAATTGTTTTACGCGTGCGTTTGTGTGGATTGTAGGACAAGGCTCCCTATGTAGC
1145	TTGGACTAGAAATCTCGTGCTGATTAATTGTTTTACGCGTGCGTTTGTGTGGATTGTAGGACAAGGCTCCCTATGTAGC
N138	ATGGACTAGAAATCTCGTGCTGATTAATTGTTTTACGCGTGCGTTTGTGTGGATTGTAGGACAAGGCTCCCTATGTAGC
E600	TTGGACTAGAAATCTCGTGCTGATTAATTGTTTTACGCGTGCGTTTGTGTGGATTGTAGGACAAGGCTCCCTATGTAGC
L061F	TTGGACTAGAAATCTCGTGCTGATTAATTGTTTTACGCGTGCGTTTGTGTGGATTGTAGGACAAGGCTCCCTATGTAGC
F7584	TTGGACTAGAAATCTCGTGCTGATTAATTGTTTTACGCGTGCGTTTGTGTGGATTGTAGGACAAGGCTCCCTATGTAGC
DM101B	TTGGACTAGAAATCTCGTGCTGATTAATTGTTTTACGCGTGCGTTTGTGTGGATTGTAGGACAAGGCTCCCTATGTAGC
D619	TTGGACTAGAAATCTCGTGCTGATTAATTGTTTTACGCGTGCGTTTGTGTGGATTGTAGGACAAGGCTCCCTATGTAGC
D15	TTGGACTAGAAATCTCGTGCTGATTAATTGTTTTACGCGTGCGTTTGTGTGGATTGTAGGACAAGGCTCCCTATGTAGC
DM07	TTGGACTAGAAATCTCGTGCTGATTAATTGTTTTACGCGTGCGTTTGTGTGGATTGTAGGACAAGGCTCCCTATGTAGC
SC24-1	TTGGACTAGAAATCTCGTGCTGATTAATTGTTTTACGCGTGCGTTTGTGTGGATTGTAGGACAAGGCTCCCTATGTAGC
Y223	TTGGACTAGAAATCTCGTGCTGATTAATTGTTTTACGCGTGCGTTTGTGTGGATTGTAGGACAAGGCTCCCTATGTAGC
C521	TTGGACTAGAAATCTCGTGCTGATTAATTGTTTTACGCGTGCGTTTGTGTGGATTGTAGGACAAGGCTCCCTATGTAGC
LM-2	TTGGACTAGAAATCTCGTGCTGATTAATTGTTTTACGCGTGCGTTTGTGTGGATTGTAGGACAAGGCTCCCTATGTAGC
B100	ATGGACTAGAAATCTCGTGCTGATTAATTGTTTTACGCGTGCGTTTGTGTGGATTGTAGGACAAGGCTCCCTATGTAGC

Lines	Sequence
B98	ATGGACTAGAAATCTCGTGCTGATTAATTGTTTTACGCGTGCGTTTGTGTGGATTGTAGGACAAGGCTCCCTATGTAGC
B95	TTGGACTAGAAATCTCGTGCTGATTAATTGTTTTACGCGTGCGTTTGTGTGGATTGTAGGACAAGGCTCCCTATGTAGC
SC-9	TTGGACTAGAAATCTCGTGCTGATTAATTGTTTTACGCGTGCGTTTGTGTGGATTGTAGGACAAGGCTCCCTATGTAGC
4003	TTGGACTAGAAATCTCGTGCTGATTAATTGTTTTACGCGTGCGTTTGTGTGGATTGTAGGACAAGGCTCCCTATGTAGC
lian87	TTGGACTAGAAATCTCGTGCTGATTAATTGTTTTACGCGTGCGTTTGTGTGGATTGTAGGACAAGGCTCCCTATGTAGC
PN2-8	TTGGACTAGAAATCTCGTGCTGATTAATTGTTTTACGCGTGCGTTTGTGTGGATTGTAGGACAAGGCTCCCTATGTAGC
L-1	ATGGACTAGAAATCTCGTGCTGATTAATTGTTTTACGCGTGCGTTTGTGTGGATTGTAGGACAAGGCTCCCTATGTAGC
W172	TTGGACTAGAAATCTCGTGCTGATTAATTGTTTTACGCGTGCGTTTGTGTGGATTGTAGGACAAGGCTCCCTATGTAGC
SW153	TTGGACTAGAAATCTCGTGCTGATTAATTGTTTTACGCGTGCGTTTGTGTGGATTGTAGGACAAGGCTCCCTATGTAGC
W238	TTGGACTAGAAATCTCGTGCTGATTAATTGTTTTACGCGTGCGTTTGTGTGGATTGTAGGACAAGGCTCCCTATGTAGC
W968	ATGGACTAGAAATCTCGTGCTGATTAATTGTTTTACGCGTGCGTTTGTGTGGATTGTAGGACAAGGCTCCCTATGTAGC
WB8	ATGGACTAGAAATCTCGTGCTGATTAATTGTTTTACGCGTGCGTTTGTGTGGATTGTAGGACAAGGCTCCCTATGTAGC
18	TTGGACTAGAAATCTCGTGCTGATTAATTGTTTTACGCGTGCGTTTGTGTGGATTGTAGGACAAGGCTCCCTATGTAGC
1614	TTGGACTAGAAATCTCGTGCTGATTAATTGTTTTACGCGTGCGTTTGTGTGGATTGTAGGACAAGGCTCCCTATGTAGC
1610	TTGGACTAGAAATCTCGTGCTGATTAATTGTTTTACGCGTGCGTTTGTGTGGATTGTAGGACAAGGCTCCCTATGTAGC
5-Apr	TTGGACTAGAAATCTCGTGCTGATTAATTGTTTTACGCGTGCGTTTGTGTGGATTGTAGGACAAGGCTCCCTATGTAGC
chen322	TTGGACTAGAAATCTCGTGCTGATTAATTGTTTTACGCGTGCGTTTGTGTGGATTGTAGGACAAGGCTCCCTATGTAGC
E601	TTGGACTAGAAATCTCGTGCTGATTAATTGTTTTACGCGTGCGTTTGTGTGGATTGTAGGACAAGGCTCCCTATGTAGC
E588	TTGGACTAGAAATCTCGTGCTGATTAATTGTTTTACGCGTGCGTTTGTGTGGATTGTAGGACAAGGCTCCCTATGTAGC
B394	TTGGACTAGAAATCTCGTGCTGATTAATTGTTTTACGCGTGCGTTTGTGTGGATTGTAGGACAAGGCTCCCTATGTAGC
changK	TTGGACTAGAAATCTCGTGCTGATTAATTGTTTTACGCGTGCGTTTGTGTGGATTGTAGGACAAGGCTCCCTATGTAGC
huangchanga	TTGGACTAGAAATCTCGTGCTGATTAATTGTTTTACGCGTGCGTTTGTGTGGATTGTAGGACAAGGCTCCCTATGTAGC

**Lines**	**Sequence**
953	TTGGACTAGAAATCTCGTGCTGATTAATTGTTTTACGCGTGCGTTTGTGTGGATTGTAGGACAAGGCTCCCTATGTAGC
D33A	ATGGACTAGAAATCTCGTGCTGATTAATTGTTTTACGCGTGCGTTTGTGTGGATTGTAGGACAAGGCTCCCTATGTAGC
huangchangb	TTGGACTAGAAATCTCGTGCTGATTAATTGTTTTACGCGTGCGTTTGTGTGGATTGTAGGACAAGGCTCCCTATGTAGC
W499	TTGGACTAGAAATCTCGTGCTGATTAATTGTTTTACGCGTGCGTTTGTGTGGATTGTAGGACAAGGCTCCCTATGTAGC
624	TTGGACTAGAAATCTCGTGCTGATTAATTGTTTTACGCGTGCGTTTGTGTGGATTGTAGGACAAGGCTCCCTATGTAGC
SC30-1	ATGGACTAGAAATCTCGTGCTGATTAATTGTTTTACGCGTGCGTTTGTGTGGATTGTAGGACAAGGCTCCCTATGTAGC
468-3	TTGGACTAGAAATCTCGTGCTGATTAATTGTTTTACGCGTGCGTTTGTGTGGATTGTAGGACAAGGCTCCCTATGTAGC
XF223	ATGGACTAGAAATCTCGTGCTGATTAATTGTTTTACGCGTGCGTTTGTGTGGATTGTAGGACAAGGCTCCCTATGTAGC
XF134	TTGGACTAGAAATCTCGTGCTGATTAATTGTTTTACGCGTGCGTTTGTGTGGATTGTAGGACAAGGCTCCCTATGTAGC
M131-5	TTGGACTAGAAATCTCGTGCTGATTAATTGTTTTACGCGTGCGTTTGTGTGGATTGTAGGACAAGGCTCCCTATGTAGC
XOP2	TTGGACTAGAAATCTCGTGCTGATTAATTGTTTTACGCGTGCGTTTGTGTGGATTGTAGGACAAGGCTCCCTATGTAGC
R1656	TTGGACTAGAAATCTCGTGCTGATTAATTGTTTTACGCGTGCGTTTGTGTGGATTGTAGGACAAGGCTCCCTATGTAGC
jian1495b	TTGGACTAGAAATCTCGTGCTGATTAATTGTTTTACGCGTGCGTTTGTGTGGATTGTAGGACAAGGCTCCCTATGTAGC
Max	ATGGACTAGAAATCTCGTGCTGATTAATTGTTTTACGCGTGCGTTTGTGTGGATTGTAGGACAAGGCTCCCTATGTAGC
T24	ATGGACTAGAAATCTCGTGCTGATTAATTGTTTTACGCGTGCGTTTGTGTGGATTGTAGGACAAGGCTCCCTATGTAGC
Lo415	TTGGACTAGAAATCTCGTGCTGATTAATTGTTTTACGCGTGCGTTTGTGTGGATTGTAGGACAAGGCTCCCTATGTAGC
chang7-2	TTGGACTAGAAATCTCGTGCTGATTAATTGTTTTACGCGTGCGTTTGTGTGGATTGTAGGACAAGGCTCCCTATGTAGC
FC-13	TTGGACTAGAAATCTCGTGCTGATTAATTGTTTTACGCGTGCGTTTGTGTGGATTGTAGGACAAGGCTCCCTATGTAGC
L05-6	TTGGACTAGAAATCTCGTGCTGATTAATTGTTTTACGCGTGCGTTTGTGTGGATTGTAGGACAAGGCTCCCTATGTAGC
ys06	TTGGACTAGAAATCTCGTGCTGATTAATTGTTTTACGCGTGCGTTTGTGTGGATTGTAGGACAAGGCTCCCTATGTAGC
L473	TTGGACTAGAAATCTCGTGCTGATTAATTGTTTTACGCGTGCGTTTGTGTGGATTGTAGGACAAGGCTCCCTATGTAGC
equn3	ATGGACTAGAAATCTCGTGCTGATTAATTGTTTTACGCGTGCGTTTGTGTGGATTGTAGGACAAGGCTCCCTATGTAGC

Lines	Sequence
equn4	TTGGACTAGAAATCTCGTGCTGATTAATTGTTTTACGCGTGCGTTTGTGTGGATTGTAGGACAAGGCTCCCTATGTAGC
18-599	TTGGACTAGAAATCTCGTGCTGATTAATTGTTTTACGCGTGCGTTTGTGTGGATTGTAGGACAAGGCTCCCTATGTAGC
08-64	TTGGACTAGAAATCTCGTGCTGATTAATTGTTTTACGCGTGCGTTTGTGTGGATTGTAGGACAAGGCTCCCTATGTAGC
dong327	ATGGACTAGAAATCTCGTGCTGATTAATTGTTTTACGCGTGCGTTTGTGTGGATTGTAGGACAAGGCTCCCTATGTAGC
K10	TTGGACTAGAAATCTCGTGCTGATTAATTGTTTTACGCGTGCGTTTGTGTGGATTGTAGGACAAGGCTCCCTATGTAGC
ji444	TTGGACTAGAAATCTCGTGCTGATTAATTGTTTTACGCGTGCGTTTGTGTGGATTGTAGGACAAGGCTCCCTATGTAGC
ben7884	TTGGACTAGAAATCTCGTGCTGATTAATTGTTTTACGCGTGCGTTTGTGTGGATTGTAGGACAAGGCTCCCTATGTAGC
huangyesi3	ATGGACTAGAAATCTCGTGCTGATTAATTGTTTTACGCGTGCGTTTGTGTGGATTGTAGGACAAGGCTCCCTATGTAGC
ji63	TTGGACTAGAAATCTCGTGCTGATTAATTGTTTTACGCGTGCGTTTGTGTGGATTGTAGGACAAGGCTCCCTATGTAGC
ji846	TTGGACTAGAAATCTCGTGCTGATTAATTGTTTTACGCGTGCGTTTGTGTGGATTGTAGGACAAGGCTCCCTATGTAGC
H21	TTGGACTAGAAATCTCGTGCTGATTAATTGTTTTACGCGTGCGTTTGTGTGGATTGTAGGACAAGGCTCCCTATGTAGC
4F1	TTGGACTAGAAATCTCGTGCTGATTAATTGTTTTACGCGTGCGTTTGTGTGGATTGTAGGACAAGGCTCCCTATGTAGC
ji4112	TTGGACTAGAAATCTCGTGCTGATTAATTGTTTTACGCGTGCGTTTGTGTGGATTGTAGGACAAGGCTCCCTATGTAGC
xi502	TTGGACTAGAAATCTCGTGCTGATTAATTGTTTTACGCGTGCGTTTGTGTGGATTGTAGGACAAGGCTCCCTATGTAGC
wenhuang31413	TTGGACTAGAAATCTCGTGCTGATTAATTGTTTTACGCGTGCGTTTGTGTGGATTGTAGGACAAGGCTCCCTATGTAGC
S22	TTGGACTAGAAATCTCGTGCTGATTAATTGTTTTACGCGTGCGTTTGTGTGGATTGTAGGACAAGGCTCCCTATGTAGC
ye8112	TTGGACTAGAAATCTCGTGCTGATTAATTGTTTTACGCGTGCGTTTGTGTGGATTGTAGGACAAGGCTCCCTATGTAGC
zheng653	TTGGACTAGAAATCTCGTGCTGATTAATTGTTTTACGCGTGCGTTTGTGTGGATTGTAGGACAAGGCTCCCTATGTAGC
tian77	TTGGACTAGAAATCTCGTGCTGATTAATTGTTTTACGCGTGCGTTTGTGTGGATTGTAGGACAAGGCTCCCTATGTAGC
tie7922	TTGGACTAGAAATCTCGTGCTGATTAATTGTTTTACGCGTGCGTTTGTGTGGATTGTAGGACAAGGCTCCCTATGTAGC
ye488	TTGGACTAGAAATCTCGTGCTGATTAATTGTTTTACGCGTGCGTTTGTGTGGATTGTAGGACAAGGCTCCCTATGTAGC
chang72	TTGGACTAGAAATCTCGTGCTGATTAATTGTTTTACGCGTGCGTTTGTGTGGATTGTAGGACAAGGCTCCCTATGTAGC

Lines	Sequence
wu109	TTGGACTAGAAATCTCGTGCTGATTAATTGTTTTACGCGTGCGTTTGTGTGGATTGTAGGACAAGGCTCCCTATGTAGC
lv28	TTGGACTAGAAATCTCGTGCTGATTAATTGTTTTACGCGTGCGTTTGTGTGGATTGTAGGACAAGGCTCCCTATGTAGC
qi205	TTGGACTAGAAATCTCGTGCTGATTAATTGTTTTACGCGTGCGTTTGTGTGGATTGTAGGACAAGGCTCCCTATGTAGC
D854	TTGGACTAGAAATCTCGTGCTGATTAATTGTTTTACGCGTGCGTTTGTGTGGATTGTAGGACAAGGCTCCCTATGTAGC
D857	TTGGACTAGAAATCTCGTGCTGATTAATTGTTTTACGCGTGCGTTTGTGTGGATTGTAGGACAAGGCTCCCTATGTAGC
D856	ATGGACTAGAAATCTCGTGCTGATTAATTGTTTTACGCGTGCGTTTGTGTGGATTGTAGGACAAGGCTCCCTATGTAGC
D864	TTGGACTAGAAATCTCGTGCTGATTAATTGTTTTACGCGTGCGTTTGTGTGGATTGTAGGACAAGGCTCCCTATGTAGC
D869	ATGGACTAGAAATCTCGTGCTGATTAATTGTTTTACGCGTGCGTTTGTGTGGATTGTAGGACAAGGCTCCCTATGTAGC
D883	TTGGACTAGAAATCTCGTGCTGATTAATTGTTTTACGCGTGCGTTTGTGTGGATTGTAGGACAAGGCTCCCTATGTAGC
D886	TTGGACTAGAAATCTCGTGCTGATTAATTGTTTTACGCGTGCGTTTGTGTGGATTGTAGGACAAGGCTCCCTATGTAGC
D1049	TTGGACTAGAAATCTCGTGCTGATTAATTGTTTTACGCGTGCGTTTGTGTGGATTGTAGGACAAGGCTCCCTATGTAGC
D1051	TTGGACTAGAAATCTCGTGCTGATTAATTGTTTTACGCGTGCGTTTGTGTGGATTGTAGGACAAGGCTCCCTATGTAGC
dan9064	TTGGACTAGAAATCTCGTGCTGATTAATTGTTTTACGCGTGCGTTTGTGTGGATTGTAGGACAAGGCTCCCTATGTAGC
3H2	TTGGACTAGAAATCTCGTGCTGATTAATTGTTTTACGCGTGCGTTTGTGTGGATTGTAGGACAAGGCTCCCTATGTAGC
yu87-1	TTGGACTAGAAATCTCGTGCTGATTAATTGTTTTACGCGTGCGTTTGTGTGGATTGTAGGACAAGGCTCCCTATGTAGC
nan21-3	TTGGACTAGAAATCTCGTGCTGATTAATTGTTTTACGCGTGCGTTTGTGTGGATTGTAGGACAAGGCTCCCTATGTAGC
K14	TTGGACTAGAAATCTCGTGCTGATTAATTGTTTTACGCGTGCGTTTGTGTGGATTGTAGGACAAGGCTCCCTATGTAGC
cheng698-3	TTGGACTAGAAATCTCGTGCTGATTAATTGTTTTACGCGTGCGTTTGTGTGGATTGTAGGACAAGGCTCCCTATGTAGC
ye52106	TTGGACTAGAAATCTCGTGCTGATTAATTGTTTTACGCGTGCGTTTGTGTGGATTGTAGGACAAGGCTCCCTATGTAGC
ye8001	TTGGACTAGAAATCTCGTGCTGATTAATTGTTTTACGCGTGCGTTTGTGTGGATTGTAGGACAAGGCTCCCTATGTAGC
suwan1611	ATGGACTAGAAATCTCGTGCTGATTAATTGTTTTACGCGTGCGTTTGTGTGGATTGTAGGACAAGGCTCCCTATGTAGC
dan599	TTGGACTAGAAATCTCGTGCTGATTAATTGTTTTACGCGTGCGTTTGTGTGGATTGTAGGACAAGGCTCCCTATGTAGC

**Lines**	**Sequence**
D892	TTGGACTAGAAATCTCGTGCTGATTAATTGTTTTACGCGTGCGTTTGTGTGGATTGTAGGACAAGGCTCCCTATGTAGC
su75	TTGGACTAGAAATCTCGTGCTGATTAATTGTTTTACGCGTGCGTTTGTGTGGATTGTAGGACAAGGCTCCCTATGTAGC
R136	TTGGACTAGAAATCTCGTGCTGATTAATTGTTTTACGCGTGCGTTTGTGTGGATTGTAGGACAAGGCTCCCTATGTAGC
R017	TTGGACTAGAAATCTCGTGCTGATTAATTGTTTTACGCGTGCGTTTGTGTGGATTGTAGGACAAGGCTCCCTATGTAGC
3489a	TTGGACTAGAAATCTCGTGCTGATTAATTGTTTTACGCGTGCGTTTGTGTGGATTGTAGGACAAGGCTCCCTATGTAGC
P138	TTGGACTAGAAATCTCGTGCTGATTAATTGTTTTACGCGTGCGTTTGTGTGGATTGTAGGACAAGGCTCCCTATGTAGC
N68a	TTGGACTAGAAATCTCGTGCTGATTAATTGTTTTACGCGTGCGTTTGTGTGGATTGTAGGACAAGGCTCCCTATGTAGC
K12	TTGGACTAGAAATCTCGTGCTGATTAATTGTTTTACGCGTGCGTTTGTGTGGATTGTAGGACAAGGCTCCCTATGTAGC
619	TTGGACTAGAAATCTCGTGCTGATTAATTGTTTTACGCGTGCGTTTGTGTGGATTGTAGGACAAGGCTCCCTATGTAGC
shen137	TTGGACTAGAAATCTCGTGCTGATTAATTGTTTTACGCGTGCGTTTGTGTGGATTGTAGGACAAGGCTCCCTATGTAGC
3335	TTGGACTAGAAATCTCGTGCTGATTAATTGTTTTACGCGTGCGTTTGTGTGGATTGTAGGACAAGGCTCCCTATGTAGC
Beck	TTGGACTAGAAATCTCGTGCTGATTAATTGTTTTACGCGTGCGTTTGTGTGGATTGTAGGACAAGGCTCCCTATGTAGC
huangzaosi	TTGGACTAGAAATCTCGTGCTGATTAATTGTTTTACGCGTGCGTTTGTGTGGATTGTAGGACAAGGCTCCCTATGTAGC
Maxa	TTGGACTAGAAATCTCGTGCTGATTAATTGTTTTACGCGTGCGTTTGTGTGGATTGTAGGACAAGGCTCCCTATGTAGC
fangyin	TTGGACTAGAAATCTCGTGCTGATTAATTGTTTTACGCGTGCGTTTGTGTGGATTGTAGGACAAGGCTCCCTATGTAGC
5032	TTGGACTAGAAATCTCGTGCTGATTAATTGTTTTACGCGTGCGTTTGTGTGGATTGTAGGACAAGGCTCCCTATGTAGC
zheng22	TTGGACTAGAAATCTCGTGCTGATTAATTGTTTTACGCGTGCGTTTGTGTGGATTGTAGGACAAGGCTCCCTATGTAGC
7903E	TTGGACTAGAAATCTCGTGCTGATTAATTGTTTTACGCGTGCGTTTGTGTGGATTGTAGGACAAGGCTCCCTATGTAGC
dan340	TTGGACTAGAAATCTCGTGCTGATTAATTGTTTTACGCGTGCGTTTGTGTGGATTGTAGGACAAGGCTCCCTATGTAGC
99122	TTGGACTAGAAATCTCGTGCTGATTAATTGTTTTACGCGTGCGTTTGTGTGGATTGTAGGACAAGGCTCCCTATGTAGC
M1016	ATGGACTAGAAATCTCGTGCTGATTAATTGTTTTACGCGTGCGTTTGTGTGGATTGTAGGACAAGGCTCCCTATGTAGC
W9706	TTGGACTAGAAATCTCGTGCTGATTAATTGTTTTACGCGTGCGTTTGTGTGGATTGTAGGACAAGGCTCCCTATGTAGC

**Lines**	**Sequence**
R25	TTGGACTAGAAATCTCGTGCTGATTAATTGTTTTACGCGTGCGTTTGTGTGGATTGTAGGACAAGGCTCCCTATGTAGC
R150	TTGGACTAGAAATCTCGTGCTGATTAATTGTTTTACGCGTGCGTTTGTGTGGATTGTAGGACAAGGCTCCCTATGTAGC
R98	TTGGACTAGAAATCTCGTGCTGATTAATTGTTTTACGCGTGCGTTTGTGTGGATTGTAGGACAAGGCTCCCTATGTAGC
chengzi2142	TTGGACTAGAAATCTCGTGCTGATTAATTGTTTTACGCGTGCGTTTGTGTGGATTGTAGGACAAGGCTCCCTATGTAGC
xing230	TTGGACTAGAAATCTCGTGCTGATTAATTGTTTTACGCGTGCGTTTGTGTGGATTGTAGGACAAGGCTCCCTATGTAGC
20564	ATGGACTAGAAATCTCGTGCTGATTAATTGTTTTACGCGTGCGTTTGTGTGGATTGTAGGACAAGGCTCCCTATGTAGC
XF77	TTGGACTAGAAATCTCGTGCTGATTAATTGTTTTACGCGTGCGTTTGTGTGGATTGTAGGACAAGGCTCCCTATGTAGC
huotanghuang	ATGGACTAGAAATCTCGTGCTGATTAATTGTTTTACGCGTGCGTTTGTGTGGATTGTAGGACAAGGCTCCCTATGTAGC
SC11-1	ATGGACTAGAAATCTCGTGCTGATTAATTGTTTTACGCGTGCGTTTGTGTGGATTGTAGGACAAGGCTCCCTATGTAGC
811A	TTGGACTAGAAATCTCGTGCTGATTAATTGTTTTACGCGTGCGTTTGTGTGGATTGTAGGACAAGGCTCCCTATGTAGC
806A	TTGGACTAGAAATCTCGTGCTGATTAATTGTTTTACGCGTGCGTTTGTGTGGATTGTAGGACAAGGCTCCCTATGTAGC
9058	TTGGACTAGAAATCTCGTGCTGATTAATTGTTTTACGCGTGCGTTTGTGTGGATTGTAGGACAAGGCTCCCTATGTAGC
PH6WC	TTGGACTAGAAATCTCGTGCTGATTAATTGTTTTACGCGTGCGTTTGTGTGGATTGTAGGACAAGGCTCCCTATGTAGC
PH4CV	TTGGACTAGAAATCTCGTGCTGATTAATTGTTTTACGCGTGCGTTTGTGTGGATTGTAGGACAAGGCTCCCTATGTAGC
hai9-21	ATGGACTAGAAATCTCGTGCTGATTAATTGTTTTACGCGTGCGTTTGTGTGGATTGTAGGACAAGGCTCCCTATGTAGC
DH40	TTGGACTAGAAATCTCGTGCTGATTAATTGTTTTACGCGTGCGTTTGTGTGGATTGTAGGACAAGGCTCCCTATGTATC
BT1	TTGGACTAGAAATCTCGTGCTGATTAATTGTTTTACGCGTGCGTTTGTGTGGATTGTAGGACAAGGCTCCCTATGTAGC
A801	TTGGACTAGAAATCTCGTGCTGATTAATTGTTTTACGCGTGCGTTTGTGTGGATTGTAGGACAAGGCTCCCTATGTAGC
DF20	TTGGACTAGAAATCTCGTGCTGATTAATTGTTTTACGCGTGCGTTTGTGTGGATTGTAGGACAAGGCTCCCTATGTAGC
DF27	TTGGACTAGAAATCTCGTGCTGATTAATTGTTTTACGCGTGCGTTTGTGTGGATTGTAGGACAAGGCTCCCTATGTAGC
DF24	TTGGACTAGAAATCTCGTGCTGATTAATTGTTTTACGCGTGCGTTTGTGTGGATTGTAGGACAAGGCTCCCTATGTAGC
7236	TTGGACTAGAAATCTCGTGCTGATTAATTGTTTTACGCGTGCGTTTGTGTGGATTGTAGGACAAGGCTCCCTATGTAGC

Lines	Sequence
433-7	TTGGACTAGAAATCTCGTGCTGATTAATTGTTTTACGCGTGCGTTTGTGTGGATTGTAGGACAAGGCTCCCTATGTAGC
shen977	ATGGACTAGAAATCTCGTGCTGATTAATTGTTTTACGCGTGCGTTTGTGTGGATTGTAGGACAAGGCTCCCTATGTAGC
K22	TTGGACTAGAAATCTCGTGCTGATTAATTGTTTTACGCGTGCGTTTGTGTGGATTGTAGGACAAGGCTCCCTATGTAGC
niu2-1	TTGGACTAGAAATCTCGTGCTGATTAATTGTTTTACGCGTGCGTTTGTGTGGATTGTAGGACAAGGCTCCCTATGTAGC
68139	TTGGACTAGAAATCTCGTGCTGATTAATTGTTTTACGCGTGCGTTTGTGTGGATTGTAGGACAAGGCTCCCTATGTAGC
1313	ATGGACTAGAAATCTCGTGCTGATTAATTGTTTTACGCGTGCGTTTGTGTGGATTGTAGGACAAGGCTCCCTATGTAGC
X178	TTGGACTAGAAATCTCGTGCTGATTAATTGTTTTACGCGTGCGTTTGTGTGGATTGTAGGACAAGGCTCCCTATGTAGC
150-4	TTGGACTAGAAATCTCGTGCTGATTAATTGTTTTACGCGTGCGTTTGTGTGGATTGTAGGACAAGGCTCCCTATGTAGC
P007	TTGGACTAGAAATCTCGTGCTGATTAATTGTTTTACGCGTGCGTTTGTGTGGATTGTAGGACAAGGCTCCCTATGTAGC
y9961	TTGGACTAGAAATCTCGTGCTGATTAATTGTTTTACGCGTGCGTTTGTGTGGATTGTAGGACAAGGCTCCCTATGTAGC
Yd6	TTGGACTAGAAATCTCGTGCTGATTAATTGTTTTACGCGTGCGTTTGTGTGGATTGTAGGACAAGGCTCCCTATGTAGC
196	TTGGACTAGAAATCTCGTGCTGATTAATTGTTTTACGCGTGCGTTTGTGTGGATTGTAGGACAAGGCTCCCTATGTAGC
68122	ATGGACTAGAAATCTCGTGCTGATTAATTGTTTTACGCGTGCGTTTGTGTGGATTGTAGGACAAGGCTCCCTATGTAGC
shen142	TTGGACTAGAAATCTCGTGCTGATTAATTGTTTTACGCGTGCGTTTGTGTGGATTGTAGGACAAGGCTCCCTATGTAGC
M22	TTGGACTAGAAATCTCGTGCTGATTAATTGTTTTACGCGTGCGTTTGTGTGGATTGTAGGACAAGGCTCCCTATGTAGC
shan89	ATGGACTAGAAATCTCGTGCTGATTAATTGTTTTACGCGTGCGTTTGTGTGGATTGTAGGACAAGGCTCCCTATGTAGC
8701	TTGGACTAGAAATCTCGTGCTGATTAATTGTTTTACGCGTGCGTTTGTGTGGATTGTAGGACAAGGCTCCCTATGTAGC
1121	TTGGACTAGAAATCTCGTGCTGATTAATTGTTTTACGCGTGCGTTTGTGTGGATTGTAGGACAAGGCTCCCTATGTAGC
dan598	TTGGACTAGAAATCTCGTGCTGATTAATTGTTTTACGCGTGCGTTTGTGTGGATTGTAGGACAAGGCTCCCTATGTAGC
Ay420	TTGGACTAGAAATCTCGTGCTGATTAATTGTTTTACGCGTGCGTTTGTGTGGATTGTAGGACAAGGCTCCCTATGTAGC
A632	TTGGACTAGAAATCTCGTGCTGATTAATTGTTTTACGCGTGCGTTTGTGTGGATTGTAGGACAAGGCTCCCTATGTAGC
Mo24W	ATGGACTAGAAATCTCGTGCTGATTAATTGTTTTACGCGTGCGTTTGTGTGGATTGTAGGACAAGGCTCCCTATGTAGC

**Lines**	**Sequence**
A679	TTGGACTAGAAATCTCGTGCTGATTAATTGTTTTACGCGTGCGTTTGTGTGGATTGTAGGACAAGGCTCCCTATGTAGC
Sg1533	ATGGACTAGAAATCTCGTGCTGATTAATTGTTTTACGCGTGCGTTTGTGTGGATTGTAGGACAAGGCTCCCTATGTAGC
Va26	TTGGACTAGAAATCTCGTGCTGATTAATTGTTTTACGCGTGCGTTTGTGTGGATTGTAGGACAAGGCTCCCTATGTAGC
W64A	TTGGACTAGAAATCTCGTGCTGATTAATTGTTTTACGCGTGCGTTTGTGTGGATTGTAGGACAAGGCTCCCTATGTAGC
W153R	TTGGACTAGAAATCTCGTGCTGATTAATTGTTTTACGCGTGCGTTTGTGTGGATTGTAGGACAAGGCTCCCTATGTAGC
4722	TTGGACTAGAAATCTCGTGCTGATTAATTGTTTTACGCGTGCGTTTGTGTGGATTGTAGGACAAGGCTCCCTATGTAGC
W2H03	ATGGACTAGAAATCTCGTGCTGATTAATTGTTTTACGCGTGCGTTTGTGTGGATTGTAGGACAAGGCTCCCTATGTAGC
bao3040	TTGGACTAGAAATCTCGTGCTGATTAATTGTTTTACGCGTGCGTTTGTGTGGATTGTAGGACAAGGCTCCCTATGTAGC
S311	TTGGACTAGAAATCTCGTGCTGATTAATTGTTTTACGCGTGCGTTTGTGTGGATTGTAGGACAAGGCTCCCTATGTAGC
7 月 1 日	TTGGACTAGAAATCTCGTGCTGATTAATTGTTTTACGCGTGCGTTTGTGTGGATTGTAGGACAAGGCTCCCTATGTAGC
xun92-8	TTGGACTAGAAATCTCGTGCTGATTAATTGTTTTACGCGTGCGTTTGTGTGGATTGTAGGACAAGGCTCCCTATGTAGC
CT109	ATGGACTAGAAATCTCGTGCTGATTAATTGTTTTACGCGTGCGTTTGTGTGGATTGTAGGACAAGGCTCCCTATGTAGC
Lx9801	TTGGACTAGAAATCTCGTGCTGATTAATTGTTTTACGCGTGCGTTTGTGTGGATTGTAGGACAAGGCTCCCTATGTAGC
zhong128	ATGGACTAGAAATCTCGTGCTGATTAATTGTTTTACGCGTGCGTTTGTGTGGATTGTAGGACAAGGCTCCCTATGTAGC
zhonghuang64	TTGGACTAGAAATCTCGTGCTGATTAATTGTTTTACGCGTGCGTTTGTGTGGATTGTAGGACAAGGCTCCCTATGTAGC
han49	TTGGACTAGAAATCTCGTGCTGATTAATTGTTTTACGCGTGCGTTTGTGTGGATTGTAGGACAAGGCTCCCTATGTAGC
ziyu3	ATGGACTAGAAATCTCGTGCTGATTAATTGTTTTACGCGTGCGTTTGTGTGGATTGTAGGACAAGGCTCCCTATGTAGC
guangyou5	ATGGACTAGAAATCTCGTGCTGATTAATTGTTTTACGCGTGCGTTTGTGTGGATTGTAGGACAAGGCTCCCTATGTAGC
daMO	TTGGACTAGAAATCTCGTGCTGATTAATTGTTTTACGCGTGCGTTTGTGTGGATTGTAGGACAAGGCTCCCTATGTAGC
yi67	TTGGACTAGAAATCTCGTGCTGATTAATTGTTTTACGCGTGCGTTTGTGTGGATTGTAGGACAAGGCTCCCTATGTAGC
Lo1125	ATGGACTAGAAATCTCGTGCTGATTAATTGTTTTACGCGTGCGTTTGTGTGGATTGTAGGACAAGGCTCCCTATGTAGC
CN165	TTGGACTAGAAATCTCGTGCTGATTAATTGTTTTACGCGTGCGTTTGTGTGGATTGTAGGACAAGGCTCCCTATGTAGC

Lines	Sequence
ji432	TTGGACTAGAAATCTCGTGCTGATTAATTGTTTTACGCGTGCGTTTGTGTGGATTGTAGGACAAGGCTCCCTATGTAGC
cheng18	TTGGACTAGAAATCTCGTGCTGATTAATTGTTTTACGCGTGCGTTTGTGTGGATTGTAGGACAAGGCTCCCTATGTAGC
82huangzao4	TTGGACTAGAAATCTCGTGCTGATTAATTGTTTTACGCGTGCGTTTGTGTGGATTGTAGGACAAGGCTCCCTATGTAGC
91huang15	TTGGACTAGAAATCTCGTGCTGATTAATTGTTTTACGCGTGCGTTTGTGTGGATTGTAGGACAAGGCTCCCTATGTAGC
Hda-5	TTGGACTAGAAATCTCGTGCTGATTAATTGTTTTACGCGTGCGTTTGTGTGGATTGTAGGACAAGGCTCCCTATGTAGC
chaoxianbai	TTGGACTAGAAATCTCGTGCTGATTAATTGTTTTACGCGTGCGTTTGTGTGGATTGTAGGACAAGGCTCCCTATGTAGC
B12	TTGGACTAGAAATCTCGTGCTGATTAATTGTTTTACGCGTGCGTTTGTGTGGATTGTAGGACAAGGCTCCCTATGTAGC
liao2202	TTGGACTAGAAATCTCGTGCTGATTAATTGTTTTACGCGTGCGTTTGTGTGGATTGTAGGACAAGGCTCCCTATGTAGC
liao2204	TTGGACTAGAAATCTCGTGCTGATTAATTGTTTTACGCGTGCGTTTGTGTGGATTGTAGGACAAGGCTCCCTATGTAGC
liao7794	TTGGACTAGAAATCTCGTGCTGATTAATTGTTTTACGCGTGCGTTTGTGTGGATTGTAGGACAAGGCTCCCTATGTAGC
liao5110	TTGGACTAGAAATCTCGTGCTGATTAATTGTTTTACGCGTGCGTTTGTGTGGATTGTAGGACAAGGCTCCCTATGTAGC
jiu22	TTGGACTAGAAATCTCGTGCTGATTAATTGTTTTACGCGTGCGTTTGTGTGGATTGTAGGACAAGGCTCCCTATGTAGC
444	TTGGACTAGAAATCTCGTGCTGATTAATTGTTTTACGCGTGCGTTTGTGTGGATTGTAGGACAAGGCTCCCTATGTAGC
D801	TTGGACTAGAAATCTCGTGCTGATTAATTGTTTTACGCGTGCGTTTGTGTGGATTGTAGGACAAGGCTCCCTATGTAGC
96201	TTGGACTAGAAATCTCGTGCTGATTAATTGTTTTACGCGTGCGTTTGTGTGGATTGTAGGACAAGGCTCCCTATGTAGC
gan41	TTGGACTAGAAATCTCGTGCTGATTAATTGTTTTACGCGTGCGTTTGTGTGGATTGTAGGACAAGGCTCCCTATGTAGC
fu8701	TTGGACTAGAAATCTCGTGCTGATTAATTGTTTTACGCGTGCGTTTGTGTGGATTGTAGGACAAGGCTCCCTATGTAGC
lu65	TTGGACTAGAAATCTCGTGCTGATTAATTGTTTTACGCGTGCGTTTGTGTGGATTGTAGGACAAGGCTCCCTATGTAGC
yan38	TTGGACTAGAAATCTCGTGCTGATTAATTGTTTTACGCGTGCGTTTGTGTGGATTGTAGGACAAGGCTCCCTATGTAGC
yan156	ATGGACTAGAAATCTCGTGCTGATTAATTGTTTTACGCGTGCGTTTGTGTGGATTGTAGGACAAGGCTCCCTATGTAGC
yan103	TTGGACTAGAAATCTCGTGCTGATTAATTGTTTTACGCGTGCGTTTGTGTGGATTGTAGGACAAGGCTCCCTATGTAGC
qi35	TTGGACTAGAAATCTCGTGCTGATTAATTGTTTTACGCGTGCGTTTGTGTGGATTGTAGGACAAGGCTCCCTATGTAGC

**Lines**	**Sequence**
200-24-13413	ATGGACTAGAAATCTCGTGCTGATTAATTGTTTTACGCGTGCGTTTGTGTGGATTGTAGGACAAGGCTCCCTATGTAGC
guan17-1	TTGGACTAGAAATCTCGTGCTGATTAATTGTTTTACGCGTGCGTTTGTGTGGATTGTAGGACAAGGCTCCCTATGTAGC
zhengbai11	TTGGACTAGAAATCTCGTGCTGATTAATTGTTTTACGCGTGCGTTTGTGTGGATTGTAGGACAAGGCTCCCTATGTAGC
jiao05	TTGGACTAGAAATCTCGTGCTGATTAATTGTTTTACGCGTGCGTTTGTGTGGATTGTAGGACAAGGCTCCCTATGTAGC
6	TTGGACTAGAAATCTCGTGCTGATTAATTGTTTTACGCGTGCGTTTGTGTGGATTGTAGGACAAGGCTCCCTATGTAGC
baiU8112	TTGGACTAGAAATCTCGTGCTGATTAATTGTTTTACGCGTGCGTTTGTGTGGATTGTAGGACAAGGCTCCCTATGTAGC
chang7daxian1	TTGGACTAGAAATCTCGTGCTGATTAATTGTTTTACGCGTGCGTTTGTGTGGATTGTAGGACAAGGCTCCCTATGTAGC
897	TTGGACTAGAAATCTCGTGCTGATTAATTGTTTTACGCGTGCGTTTGTGTGGATTGTAGGACAAGGCTCCCTATGTAGC
cai11-8	TTGGACTAGAAATCTCGTGCTGATTAATTGTTTTACGCGTGCGTTTGTGTGGATTGTAGGACAAGGCTCCCTATGTAGC
75-14gao	TTGGACTAGAAATCTCGTGCTGATTAATTGTTTTACGCGTGCGTTTGTGTGGATTGTAGGACAAGGCTCCCTATGTAGC
030-1	TTGGACTAGAAATCTCGTGCTGATTAATTGTTTTACGCGTGCGTTTGTGTGGATTGTAGGACAAGGCTCCCTATGTAGC
707	TTGGACTAGAAATCTCGTGCTGATTAATTGTTTTACGCGTGCGTTTGTGTGGATTGTAGGACAAGGCTCCCTATGTAGC
wu312	ATGGACTAGAAATCTCGTGCTGATTAATTGTTTTACGCGTGCGTTTGTGTGGATTGTAGGACAAGGCTCCCTATGTAGC
L005	ATGGACTAGAAATCTCGTGCTGATTAATTGTTTTACGCGTGCGTTTGTGTGGATTGTAGGACAAGGCTCCCTATGTAGC
L069	ATGGACTAGAAATCTCGTGCTGATTAATTGTTTTACGCGTGCGTTTGTGTGGATTGTAGGACAAGGCTCCCTATGTAGC
ning24	ATGGACTAGAAATCTCGTGCTGATTAATTGTTTTACGCGTGCGTTTGTGTGGATTGTAGGACAAGGCTCCCTATGTAGC
zhongyin10	TTGGACTAGAAATCTCGTGCTGATTAATTGTTTTACGCGTGCGTTTGTGTGGATTGTAGGACAAGGCTCCCTATGTAGC
zhongyin15	ATGGACTAGAAATCTCGTGCTGATTAATTGTTTTACGCGTGCGTTTGTGTGGATTGTAGGACAAGGCTCCCTATGTAGC
NN14B	TTGGACTAGAAATCTCGTGCTGATTAATTGTTTTACGCGTGCGTTTGTGTGGATTGTAGGACAAGGCTCCCTATGTAGC
ZPON7	ATGGACTAGAAATCTCGTGCTGATTAATTGTTTTACGCGTGCGTTTGTGTGGATTGTAGGACAAGGCTCCCTATGTAGC
75-364	TTGGACTAGAAATCTCGTGCTGATTAATTGTTTTACGCGTGCGTTTGTGTGGATTGTAGGACAAGGCTCCCTATGTAGC
MP704	TTGGACTAGAAATCTCGTGCTGATTAATTGTTTTACGCGTGCGTTTGTGTGGATTGTAGGACAAGGCTCCCTATGTAGC

Lines	Sequence
L105	ATGGACTAGAAATCTCGTGCTGATTAATTGTTTTACGCGTGCGTTTGTGTGGATTGTAGGACAAGGCTCCCTATGTAGC
Va35	TTGGACTAGAAATCTCGTGCTGATTAATTGTTTTACGCGTGCGTTTGTGTGGATTGTAGGACAAGGCTCCCTATGTAGC
De811	TTGGACTAGAAATCTCGTGCTGATTAATTGTTTTACGCGTGCGTTTGTGTGGATTGTAGGACAAGGCTCCCTATGTAGC
DH65232(DH9)	TTGGACTAGAAATCTCGTGCTGATTAATTGTTTTACGCGTGCGTTTGTGTGGATTGTAGGACAAGGCTCCCTATGTAGC
Lo1067	TTGGACTAGAAATCTCGTGCTGATTAATTGTTTTACGCGTGCGTTTGTGTGGATTGTAGGACAAGGCTCCCTATGTAGC
han21	TTGGACTAGAAATCTCGTGCTGATTAATTGTTTTACGCGTGCGTTTGTGTGGATTGTAGGACAAGGCTCCCTATGTAGC
chang69	TTGGACTAGAAATCTCGTGCTGATTAATTGTTTTACGCGTGCGTTTGTGTGGATTGTAGGACAAGGCTCCCTATGTAGC
huobai	ATGGACTAGAAATCTCGTGCTGATTAATTGTTTTACGCGTGCGTTTGTGTGGATTGTAGGACAAGGCTCCCTATGTAGC
yuanwu05	TTGGACTAGAAATCTCGTGCTGATTAATTGTTTTACGCGTGCGTTTGTGTGGATTGTAGGACAAGGCTCCCTATGTAGC
S7913	TTGGACTAGAAATCTCGTGCTGATTAATTGTTTTACGCGTGCGTTTGTGTGGATTGTAGGACAAGGCTCCCTATGTAGC
xingK36	TTGGACTAGAAATCTCGTGCTGATTAATTGTTTTACGCGTGCGTTTGTGTGGATTGTAGGACAAGGCTCCCTATGTAGC
BS110	TTGGACTAGAAATCTCGTGCTGATTAATTGTTTTACGCGTGCGTTTGTGTGGATTGTAGGACAAGGCTCCCTATGTAGC
LH52	TTGGACTAGAAATCTCGTGCTGATTAATTGTTTTACGCGTGCGTTTGTGTGGATTGTAGGACAAGGCTCCCTATGTAGC
FR14	TTGGACTAGAAATCTCGTGCTGATTAATTGTTTTACGCGTGCGTTTGTGTGGATTGTAGGACAAGGCTCCCTATGTAGC
F42	TTGGACTAGAAATCTCGTGCTGATTAATTGTTTTACGCGTGCGTTTGTGTGGATTGTAGGACAAGGCTCCCTATGTAGC
LH93	ATGGACTAGAAATCTCGTGCTGATTAATTGTTTTACGCGTGCGTTTGTGTGGATTGTAGGACAAGGCTCCCTATGTAGC
807	TTGGACTAGAAATCTCGTGCTGATTAATTGTTTTACGCGTGCGTTTGTGTGGATTGTAGGACAAGGCTCCCTATGTAGC
PHN11	TTGGACTAGAAATCTCGTGCTGATTAATTGTTTTACGCGTGCGTTTGTGTGGATTGTAGGACAAGGCTCCCTATGTAGC
680	TTGGACTAGAAATCTCGTGCTGATTAATTGTTTTACGCGTGCGTTTGTGTGGATTGTAGGACAAGGCTCCCTATGTAGC
LH54	TTGGACTAGAAATCTCGTGCTGATTAATTGTTTTACGCGTGCGTTTGTGTGGATTGTAGGACAAGGCTCCCTATGTAGC
DJ7	TTGGACTAGAAATCTCGTGCTGATTAATTGTTTTACGCGTGCGTTTGTGTGGATTGTAGGACAAGGCTCCCTATGTAGC
PHG83	TTGGACTAGAAATCTCGTGCTGATTAATTGTTTTACGCGTGCGTTTGTGTGGATTGTAGGACAAGGCTCCCTATGTAGC

**Lines**	**Sequence**
PHG72	TTGGACTAGAAATCTCGTGCTGATTAATTGTTTTACGCGTGCGTTTGTGTGGATTGTAGGACAAGGCTCCCTATGTAGC
B09	TTGGACTAGAAATCTCGTGCTGATTAATTGTTTTACGCGTGCGTTTGTGTGGATTGTAGGACAAGGCTCCCTATGTAGC
PHW43	TTGGACTAGAAATCTCGTGCTGATTAATTGTTTTACGCGTGCGTTTGTGTGGATTGTAGGACAAGGCTCCCTATGTAGC
PHT60	ATGGACTAGAAATCTCGTGCTGATTAATTGTTTTACGCGTGCGTTTGTGTGGATTGTAGGACAAGGCTCCCTATGTAGC
PHW52	TTGGACTAGAAATCTCGTGCTGATTAATTGTTTTACGCGTGCGTTTGTGTGGATTGTAGGACAAGGCTCCCTATGTAGC
PHW79	TTGGACTAGAAATCTCGTGCTGATTAATTGTTTTACGCGTGCGTTTGTGTGGATTGTAGGACAAGGCTCCCTATGTAGC
PHJ31	ATGGACTAGAAATCTCGTGCTGATTAATTGTTTTACGCGTGCGTTTGTGTGGATTGTAGGACAAGGCTCCCTATGTAGC
PHN29	ATGGACTAGAAATCTCGTGCTGATTAATTGTTTTACGCGTGCGTTTGTGTGGATTGTAGGACAAGGCTCCCTATGTAGC
795	TTGGACTAGAAATCTCGTGCTGATTAATTGTTTTACGCGTGCGTTTGTGTGGATTGTAGGACAAGGCTCCCTATGTAGC
LH190	TTGGACTAGAAATCTCGTGCTGATTAATTGTTTTACGCGTGCGTTTGTGTGGATTGTAGGACAAGGCTCCCTATGTAGC
IBC2	ATGGACTAGAAATCTCGTGCTGATTAATTGTTTTACGCGTGCGTTTGTGTGGATTGTAGGACAAGGCTCCCTATGTAGC
PHK24	TTGGACTAGAAATCTCGTGCTGATTAATTGTTTTACGCGTGCGTTTGTGTGGATTGTAGGACAAGGCTCCCTATGTAGC
PHG71	ATGGACTAGAAATCTCGTGCTGATTAATTGTTTTACGCGTGCGTTTGTGTGGATTGTAGGACAAGGCTCCCTATGTAGC
PB80	TTGGACTAGAAATCTCGTGCTGATTAATTGTTTTACGCGTGCGTTTGTGTGGATTGTAGGACAAGGCTCCCTATGTAGC
FAPW	TTGGACTAGAAATCTCGTGCTGATTAATTGTTTTACGCGTGCGTTTGTGTGGATTGTAGGACAAGGCTCCCTATGTAGC
LH61	TTGGACTAGAAATCTCGTGCTGATTAATTGTTTTACGCGTGCGTTTGTGTGGATTGTAGGACAAGGCTCCCTATGTAGC
Q381	ATGGACTAGAAATCTCGTGCTGATTAATTGTTTTACGCGTGCGTTTGTGTGGATTGTAGGACAAGGCTCCCTATGTAGC
PHJ40	TTGGACTAGAAATCTCGTGCTGATTAATTGTTTTACGCGTGCGTTTGTGTGGATTGTAGGACAAGGCTCCCTATGTAGC
PH207	ATGGACTAGAAATCTCGTGCTGATTAATTGTTTTACGCGTGCGTTTGTGTGGATTGTAGGACAAGGCTCCCTATGTAGC
Mo17	TTGGACTAGAAATCTCGTGCTGATTAATTGTTTTACGCGTGCGTTTGTGTGGATTGTAGGACAAGGCTCCCTATGTAGC
B73	TTGGACTAGAAATCTCGTGCTGATTAATTGTTTTACGCGTGCGTTTGTGTGGATTGTAGGACAAGGCTCCCTATGTAGC
PHG39	TTGGACTAGAAATCTCGTGCTGATTAATTGTTTTACGCGTGCGTTTGTGTGGATTGTAGGACAAGGCTCCCTATGTAGC

Lines	Sequence
LH51	TTGGACTAGAAATCTCGTGCTGATTAATTGTTTTACGCGTGCGTTTGTGTGGATTGTAGGACAAGGCTCCCTATGTAGC
LH74	TTGGACTAGAAATCTCGTGCTGATTAATTGTTTTACGCGTGCGTTTGTGTGGATTGTAGGACAAGGCTCCCTATGTAGC
LH132	TTGGACTAGAAATCTCGTGCTGATTAATTGTTTTACGCGTGCGTTTGTGTGGATTGTAGGACAAGGCTCCCTATGTAGC
Seagu1117	TTGGACTAGAAATCTCGTGCTGATTAATTGTTTTACGCGTGCGTTTGTGTGGATTGTAGGACAAGGCTCCCTATGTAGC
PHT55	TTGGACTAGAAATCTCGTGCTGATTAATTGTTTTACGCGTGCGTTTGTGTGGATTGTAGGACAAGGCTCCCTATGTAGC
OH43	TTGGACTAGAAATCTCGTGCTGATTAATTGTTTTACGCGTGCGTTTGTGTGGATTGTAGGACAAGGCTCCCTATGTAGC
LH65	TTGGACTAGAAATCTCGTGCTGATTAATTGTTTTACGCGTGCGTTTGTGTGGATTGTAGGACAAGGCTCCCTATGTAGC
HBAI	TTGGACTAGAAATCTCGTGCTGATTAATTGTTTTACGCGTGCGTTTGTGTGGATTGTAGGACAAGGCTCCCTATGTAGC
oh07B	TTGGACTAGAAATCTCGTGCTGATTAATTGTTTTACGCGTGCGTTTGTGTGGATTGTAGGACAAGGCTCCCTATGTAGC
PHH93	ATGGACTAGAAATCTCGTGCTGATTAATTGTTTTACGCGTGCGTTTGTGTGGATTGTAGGACAAGGCTCCCTATGTAGC
PHP02	TTGGACTAGAAATCTCGTGCTGATTAATTGTTTTACGCGTGCGTTTGTGTGGATTGTAGGACAAGGCTCCCTATGTAGC
78551S	TTGGACTAGAAATCTCGTGCTGATTAATTGTTTTACGCGTGCGTTTGTGTGGATTGTAGGACAAGGCTCCCTATGTAGC
PHG24	TTGGACTAGAAATCTCGTGCTGATTAATTGTTTTACGCGTGCGTTTGTGTGGATTGTAGGACAAGGCTCCCTATGTAGC
FBHJ	ATGGACTAGAAATCTCGTGCTGATTAATTGTTTTACGCGTGCGTTTGTGTGGATTGTAGGACAAGGCTCCCTATGTAGC
794	TTGGACTAGAAATCTCGTGCTGATTAATTGTTTTACGCGTGCGTTTGTGTGGATTGTAGGACAAGGCTCCCTATGTAGC
743	TTGGACTAGAAATCTCGTGCTGATTAATTGTTTTACGCGTGCGTTTGTGTGGATTGTAGGACAAGGCTCCCTATGTAGC
LH149	TTGGACTAGAAATCTCGTGCTGATTAATTGTTTTACGCGTGCGTTTGTGTGGATTGTAGGACAAGGCTCCCTATGTAGC
764	TTGGACTAGAAATCTCGTGCTGATTAATTGTTTTACGCGTGCGTTTGTGTGGATTGTAGGACAAGGCTCCCTATGTAGC
PHT77	TTGGACTAGAAATCTCGTGCTGATTAATTGTTTTACGCGTGCGTTTGTGTGGATTGTAGGACAAGGCTCCCTATGTAGC
740	TTGGACTAGAAATCTCGTGCTGATTAATTGTTTTACGCGTGCGTTTGTGTGGATTGTAGGACAAGGCTCCCTATGTAGC
LH145	TTGGACTAGAAATCTCGTGCTGATTAATTGTTTTACGCGTGCGTTTGTGTGGATTGTAGGACAAGGCTCCCTATGTAGC
LH57	TTGGACTAGAAATCTCGTGCTGATTAATTGTTTTACGCGTGCGTTTGTGTGGATTGTAGGACAAGGCTCCCTATGTAGC

**Lines**	**Sequence**
LH205	TTGGACTAGAAATCTCGTGCTGATTAATTGTTTTACGCGTGCGTTTGTGTGGATTGTAGGACAAGGCTCCCTATGTAGC
LH196	TTGGACTAGAAATCTCGTGCTGATTAATTGTTTTACGCGTGCGTTTGTGTGGATTGTAGGACAAGGCTCCCTATGTAGC
LH220Ht	TTGGACTAGAAATCTCGTGCTGATTAATTGTTTTACGCGTGCGTTTGTGTGGATTGTAGGACAAGGCTCCCTATGTAGC
LH162	TTGGACTAGAAATCTCGTGCTGATTAATTGTTTTACGCGTGCGTTTGTGTGGATTGTAGGACAAGGCTCCCTATGTAGC
LH202	TTGGACTAGAAATCTCGTGCTGATTAATTGTTTTACGCGTGCGTTTGTGTGGATTGTAGGACAAGGCTCCCTATGTAGC
LH192	TTGGACTAGAAATCTCGTGCTGATTAATTGTTTTACGCGTGCGTTTGTGTGGATTGTAGGACAAGGCTCCCTATGTAGC
PHP76	ATGGACTAGAAATCTCGTGCTGATTAATTGTTTTACGCGTGCGTTTGTGTGGATTGTAGGACAAGGCTCCCTATGTAGC
PHW51	TTGGACTAGAAATCTCGTGCTGATTAATTGTTTTACGCGTGCGTTTGTGTGGATTGTAGGACAAGGCTCCCTATGTAGC
LH208	TTGGACTAGAAATCTCGTGCTGATTAATTGTTTTACGCGTGCGTTTGTGTGGATTGTAGGACAAGGCTCCCTATGTAGC
Lp215D	TTGGACTAGAAATCTCGTGCTGATTAATTGTTTTACGCGTGCGTTTGTGTGGATTGTAGGACAAGGCTCCCTATGTAGC
PHK93	TTGGACTAGAAATCTCGTGCTGATTAATTGTTTTACGCGTGCGTTTGTGTGGATTGTAGGACAAGGCTCCCTATGTAGC
PHN66	TTGGACTAGAAATCTCGTGCTGATTAATTGTTTTACGCGTGCGTTTGTGTGGATTGTAGGACAAGGCTCCCTATGTAGC
PHR58	TTGGACTAGAAATCTCGTGCTGATTAATTGTTTTACGCGTGCGTTTGTGTGGATTGTAGGACAAGGCTCCCTATGTAGC
PHW30	TTGGACTAGAAATCTCGTGCTGATTAATTGTTTTACGCGTGCGTTTGTGTGGATTGTAGGACAAGGCTCCCTATGTAGC
29MIBZ2	ATGGACTAGAAATCTCGTGCTGATTAATTGTTTTACGCGTGCGTTTGTGTGGATTGTAGGACAAGGCTCCCTATGTAGC
MBSJ	TTGGACTAGAAATCTCGTGCTGATTAATTGTTTTACGCGTGCGTTTGTGTGGATTGTAGGACAAGGCTCCCTATGTAGC
LH215	TTGGACTAGAAATCTCGTGCTGATTAATTGTTTTACGCGTGCGTTTGTGTGGATTGTAGGACAAGGCTCCCTATGTAGC
MM402A	ATGGACTAGAAATCTCGTGCTGATTAATTGTTTTACGCGTGCGTTTGTGTGGATTGTAGGACAAGGCTCCCTATGTAGC
3IBZ2	ATGGACTAGAAATCTCGTGCTGATTAATTGTTTTACGCGTGCGTTTGTGTGGATTGTAGGACAAGGCTCCCTATGTAGC
LIBC4	ATGGACTAGAAATCTCGTGCTGATTAATTGTTTTACGCGTGCGTTTGTGTGGATTGTAGGACAAGGCTCCCTATGTAGC
83IBI3	ATGGACTAGAAATCTCGTGCTGATTAATTGTTTTACGCGTGCGTTTGTGTGGATTGTAGGACAAGGCTCCCTATGTAGC
NQ508	TTGGACTAGAAATCTCGTGCTGATTAATTGTTTTACGCGTGCGTTTGTGTGGATTGTAGGACAAGGCTCCCTATGTAGC

Lines	Sequence
PHBA6	TTGGACTAGAAATCTCGTGCTGATTAATTGTTTTACGCGTGCGTTTGTGTGGATTGTAGGACAAGGCTCCCTATGTAGC
PHPR5	TTGGACTAGAAATCTCGTGCTGATTAATTGTTTTACGCGTGCGTTTGTGTGGATTGTAGGACAAGGCTCCCTATGTAGC
PHT69	TTGGACTAGAAATCTCGTGCTGATTAATTGTTTTACGCGTGCGTTTGTGTGGATTGTAGGACAAGGCTCCCTATGTAGC
PHV53	TTGGACTAGAAATCTCGTGCTGATTAATTGTTTTACGCGTGCGTTTGTGTGGATTGTAGGACAAGGCTCCCTATGTAGC
PHWG5	TTGGACTAGAAATCTCGTGCTGATTAATTGTTTTACGCGTGCGTTTGTGTGGATTGTAGGACAAGGCTCCCTATGTAGC
ML606	ATGGACTAGAAATCTCGTGCTGATTAATTGTTTTACGCGTGCGTTTGTGTGGATTGTAGGACAAGGCTCCCTATGTAGC
LH119	TTGGACTAGAAATCTCGTGCTGATTAATTGTTTTACGCGTGCGTTTGTGTGGATTGTAGGACAAGGCTCCCTATGTAGC
MDF-13D	TTGGACTAGAAATCTCGTGCTGATTAATTGTTTTACGCGTGCGTTTGTGTGGATTGTAGGACAAGGCTCCCTATGTAGC
CR1HT	TTGGACTAGAAATCTCGTGCTGATTAATTGTTTTACGCGTGCGTTTGTGTGGATTGTAGGACAAGGCTCCCTATGTAGC
IB014	ATGGACTAGAAATCTCGTGCTGATTAATTGTTTTACGCGTGCGTTTGTGTGGATTGTAGGACAAGGCTCCCTATGTAGC
5707	TTGGACTAGAAATCTCGTGCTGATTAATTGTTTTACGCGTGCGTTTGTGTGGATTGTAGGACAAGGCTCCCTATGTAGC
PHR36	TTGGACTAGAAATCTCGTGCTGATTAATTGTTTTACGCGTGCGTTTGTGTGGATTGTAGGACAAGGCTCCCTATGTAGC
PHW17	TTGGACTAGAAATCTCGTGCTGATTAATTGTTTTACGCGTGCGTTTGTGTGGATTGTAGGACAAGGCTCCCTATGTAGC
LP5	TTGGACTAGAAATCTCGTGCTGATTAATTGTTTTACGCGTGCGTTTGTGTGGATTGTAGGACAAGGCTCCCTATGTAGC
PHG86	TTGGACTAGAAATCTCGTGCTGATTAATTGTTTTACGCGTGCGTTTGTGTGGATTGTAGGACAAGGCTCCCTATGTAGC
IBB15	ATGGACTAGAAATCTCGTGCTGATTAATTGTTTTACGCGTGCGTTTGTGTGGATTGTAGGACAAGGCTCCCTATGTAGC
W8304	TTGGACTAGAAATCTCGTGCTGATTAATTGTTTTACGCGTGCGTTTGTGTGGATTGTAGGACAAGGCTCCCTATGTAGC
2369	TTGGACTAGAAATCTCGTGCTGATTAATTGTTTTACGCGTGCGTTTGTGTGGATTGTAGGACAAGGCTCCCTATGTAGC
MBST	TTGGACTAGAAATCTCGTGCTGATTAATTGTTTTACGCGTGCGTTTGTGTGGATTGTAGGACAAGGCTCCCTATGTAGC
OQ603	TTGGACTAGAAATCTCGTGCTGATTAATTGTTTTACGCGTGCGTTTGTGTGGATTGTAGGACAAGGCTCCCTATGTAGC
S8326	TTGGACTAGAAATCTCGTGCTGATTAATTGTTTTACGCGTGCGTTTGTGTGGATTGTAGGACAAGGCTCCCTATGTAGC
1538	TTGGACTAGAAATCTCGTGCTGATTAATTGTTTTACGCGTGCGTTTGTGTGGATTGTAGGACAAGGCTCCCTATGTAGC

**Lines**	**Sequence**
CR14	TTGGACTAGAAATCTCGTGCTGATTAATTGTTTTACGCGTGCGTTTGTGTGGATTGTAGGACAAGGCTCCCTATGTAGC
WIL900	TTGGACTAGAAATCTCGTGCTGATTAATTGTTTTACGCGTGCGTTTGTGTGGATTGTAGGACAAGGCTCCCTATGTAGC
WIL903	TTGGACTAGAAATCTCGTGCTGATTAATTGTTTTACGCGTGCGTTTGTGTGGATTGTAGGACAAGGCTCCCTATGTAGC
L127	ATGGACTAGAAATCTCGTGCTGATTAATTGTTTTACGCGTGCGTTTGTGTGGATTGTAGGACAAGGCTCCCTATGTAGC
L135	ATGGACTAGAAATCTCGTGCTGATTAATTGTTTTACGCGTGCGTTTGTGTGGATTGTAGGACAAGGCTCCCTATGTAGC
L139	ATGGACTAGAAATCTCGTGCTGATTAATTGTTTTACGCGTGCGTTTGTGTGGATTGTAGGACAAGGCTCCCTATGTAGC
PHK35	TTGGACTAGAAATCTCGTGCTGATTAATTGTTTTACGCGTGCGTTTGTGTGGATTGTAGGACAAGGCTCCCTATGTAGC
PHM10	ATGGACTAGAAATCTCGTGCTGATTAATTGTTTTACGCGTGCGTTTGTGTGGATTGTAGGACAAGGCTCCCTATGTAGC
PHP60	TTGGACTAGAAATCTCGTGCTGATTAATTGTTTTACGCGTGCGTTTGTGTGGATTGTAGGACAAGGCTCCCTATGTAGC
PHR63	TTGGACTAGAAATCTCGTGCTGATTAATTGTTTTACGCGTGCGTTTGTGTGGATTGTAGGACAAGGCTCCCTATGTAGC
PHV37	TTGGACTAGAAATCTCGTGCTGATTAATTGTTTTACGCGTGCGTTTGTGTGGATTGTAGGACAAGGCTCCCTATGTAGC
中 741	TTGGACTAGAAATCTCGTGCTGATTAATTGTTTTACGCGTGCGTTTGTGTGGATTGTAGGACAAGGCTCCCTATGTAGC
太 1/Lg	TTGGACTAGAAATCTCGTGCTGATTAATTGTTTTACGCGTGCGTTTGTGTGGATTGTAGGACAAGGCTCCCTATGTAGC
LH150	TTGGACTAGAAATCTCGTGCTGATTAATTGTTTTACGCGTGCGTTTGTGTGGATTGTAGGACAAGGCTCCCTATGTAGC
B64	TTGGACTAGAAATCTCGTGCTGATTAATTGTTTTACGCGTGCGTTTGTGTGGATTGTAGGACAAGGCTCCCTATGTAGC
B67	TTGGACTAGAAATCTCGTGCTGATTAATTGTTTTACGCGTGCGTTTGTGTGGATTGTAGGACAAGGCTCCCTATGTAGC
B129	TTGGACTAGAAATCTCGTGCTGATTAATTGTTTTACGCGTGCGTTTGTGTGGATTGTAGGACAAGGCTCCCTATGTAGC
B130	TTGGACTAGAAATCTCGTGCTGATTAATTGTTTTACGCGTGCGTTTGTGTGGATTGTAGGACAAGGCTCCCTATGTAGC
CI187-2	TTGGACTAGAAATCTCGTGCTGATTAATTGTTTTACGCGTGCGTTTGTGTGGATTGTAGGACAAGGCTCCCTATGTAGC
WD456	TTGGACTAGAAATCTCGTGCTGATTAATTGTTTTACGCGTGCGTTTGTGTGGATTGTAGGACAAGGCTCCCTATGTAGC
792	TTGGACTAGAAATCTCGTGCTGATTAATTGTTTTACGCGTGCGTTTGTGTGGATTGTAGGACAAGGCTCCCTATGTAGC
DNMD30(东农)	TTGGACTAGAAATCTCGTGCTGATTAATTGTTTTACGCGTGCGTTTGTGTGGATTGTAGGACAAGGCTCCCTATGTAGC

**Lines**	**Sequence**
龙单 16♀	TTGGACTAGAAATCTCGTGCTGATTAATTGTTTTACGCGTGCGTTTGTGTGGATTGTAGGACAAGGCTCCCTATGTAGC
海 268	ATGGACTAGAAATCTCGTGCTGATTAATTGTTTTACGCGTGCGTTTGTGTGGATTGTAGGACAAGGCTCCCTATGTAGC
H2417	TTGGACTAGAAATCTCGTGCTGATTAATTGTTTTACGCGTGCGTTTGTGTGGATTGTAGGACAAGGCTCCCTATGTAGC
2036	TTGGACTAGAAATCTCGTGCTGATTAATTGTTTTACGCGTGCGTTTGTGTGGATTGTAGGACAAGGCTCCCTATGTAGC
H145	ATGGACTAGAAATCTCGTGCTGATTAATTGTTTTACGCGTGCGTTTGTGTGGATTGTAGGACAAGGCTCCCTATGTAGC
H1311	TTGGACTAGAAATCTCGTGCTGATTAATTGTTTTACGCGTGCGTTTGTGTGGATTGTAGGACAAGGCTCCCTATGTAGC
H957	TTGGACTAGAAATCTCGTGCTGATTAATTGTTTTACGCGTGCGTTTGTGTGGATTGTAGGACAAGGCTCCCTATGTAGC
H954	TTGGACTAGAAATCTCGTGCTGATTAATTGTTTTACGCGTGCGTTTGTGTGGATTGTAGGACAAGGCTCCCTATGTAGC
268(9 粒)	ATGGACTAGAAATCTCGTGCTGATTAATTGTTTTACGCGTGCGTTTGTGTGGATTGTAGGACAAGGCTCCCTATGTAGC
FR1454	TTGGACTAGAAATCTCGTGCTGATTAATTGTTTTACGCGTGCGTTTGTGTGGATTGTAGGACAAGGCTCCCTATGTAGC
KP315	TTGGACTAGAAATCTCGTGCTGATTAATTGTTTTACGCGTGCGTTTGTGTGGATTGTAGGACAAGGCTCCCTATGTAGC
KM501	ATGGACTAGAAATCTCGTGCTGATTAATTGTTTTACGCGTGCGTTTGTGTGGATTGTAGGACAAGGCTCCCTATGTAGC
EP8	TTGGACTAGAAATCTCGTGCTGATTAATTGTTTTACGCGTGCGTTTGTGTGGATTGTAGGACAAGGCTCCCTATGTAGC
Zheng58	TTGGACTAGAAATCTCGTGCTGATTAATTGTTTTACGCGTGCGTTTGTGTGGATTGTAGGACAAGGCTCCCTATGTAGC
c7-2	TTGGACTAGAAATCTCGTGCTGATTAATTGTTTTACGCGTGCGTTTGTGTGGATTGTAGGACAAGGCTCCCTATGTAGC
Huangc	TTGGACTAGAAATCTCGTGCTGATTAATTGTTTTACGCGTGCGTTTGTGTGGATTGTAGGACAAGGCTCCCTATGTAGC
科禾 699-母本	TTGGACTAGAAATCTCGTGCTGATTAATTGTTTTACGCGTGCGTTTGTGTGGATTGTAGGACAAGGCTCCCTATGTAGC
中农大 788-母本	TTGGACTAGAAATCTCGTGCTGATTAATTGTTTTACGCGTGCGTTTGTGTGGATTGTAGGACAAGGCTCCCTATGTAGC
科禾 699-父本	TTGGACTAGAAATCTCGTGCTGATTAATTGTTTTACGCGTGCGTTTGTGTGGATTGTAGGACAAGGCTCCCTATGTAGC
DH149	TTGGACTAGAAATCTCGTGCTGATTAATTGTTTTACGCGTGCGTTTGTGTGGATTGTAGGACAAGGCTCCCTATGTAGC
B4	ATGGACTAGAAATCTCGTGCTGATTAATTGTTTTACGCGTGCGTTTGTGTGGATTGTAGGACAAGGCTCCCTATGTAGC
78599	ATGGACTAGAAATCTCGTGCTGATTAATTGTTTTACGCGTGCGTTTGTGTGGATTGTAGGACAAGGCTCCCTATGTAGC

**Lines**	**Sequence**
807D	TTGGACTAGAAATCTCGTGCTGATTAATTGTTTTACGCGTGCGTTTGTGTGGATTGTAGGACAAGGCTCCCTATGTAGC
E200	ATGGACTAGAAATCTCGTGCTGATTAATTGTTTTACGCGTGCGTTTGTGTGGATTGTAGGACAAGGCTCCCTATGTAGC
D88	ATGGACTAGAAATCTCGTGCTGATTAATTGTTTTACGCGTGCGTTTGTGTGGATTGTAGGACAAGGCTCCCTATGTAGC
B97	TTGGACTAGAAATCTCGTGCTGATTAATTGTTTTACGCGTGCGTTTGTGTGGATTGTAGGACAAGGCTCCCTATGTAGC
B102	TTGGACTAGAAATCTCGTGCTGATTAATTGTTTTACGCGTGCGTTTGTGTGGATTGTAGGACAAGGCTCCCTATGTAGC
zong3	TTGGACTAGAAATCTCGTGCTGATTAATTGTTTTACGCGTGCGTTTGTGTGGATTGTAGGACAAGGCTCCCTATGTAGC
W966	TTGGACTAGAAATCTCGTGCTGATTAATTGTTTTACGCGTGCGTTTGTGTGGATTGTAGGACAAGGCTCCCTATGTAGC
changD	TTGGACTAGAAATCTCGTGCTGATTAATTGTTTTACGCGTGCGTTTGTGTGGATTGTAGGACAAGGCTCCCTATGTAGC
M101	TTGGACTAGAAATCTCGTGCTGATTAATTGTTTTACGCGTGCGTTTGTGTGGATTGTAGGACAAGGCTCCCTATGTAGC
jian1495a	TTGGACTAGAAATCTCGTGCTGATTAATTGTTTTACGCGTGCGTTTGTGTGGATTGTAGGACAAGGCTCCCTATGTAGC
D20	TTGGACTAGAAATCTCGTGCTGATTAATTGTTTTACGCGTGCGTTTGTGTGGATTGTAGGACAAGGCTCCCTATGTAGC
PN2	TTGGACTAGAAATCTCGTGCTGATTAATTGTTTTACGCGTGCGTTTGTGTGGATTGTAGGACAAGGCTCCCTATGTAGC
hai014	ATGGACTAGAAATCTCGTGCTGATTAATTGTTTTACGCGTGCGTTTGTGTGGATTGTAGGACAAGGCTCCCTATGTAGC
chang3	TTGGACTAGAAATCTCGTGCTGATTAATTGTTTTACGCGTGCGTTTGTGTGGATTGTAGGACAAGGCTCCCTATGTAGC
shuang741	TTGGACTAGAAATCTCGTGCTGATTAATTGTTTTACGCGTGCGTTTGTGTGGATTGTAGGACAAGGCTCCCTATGTAGC
ji434	TTGGACTAGAAATCTCGTGCTGATTAATTGTTTTACGCGTGCGTTTGTGTGGATTGTAGGACAAGGCTCCCTATGTAGC
MO113	TTGGACTAGAAATCTCGTGCTGATTAATTGTTTTACGCGTGCGTTTGTGTGGATTGTAGGACAAGGCTCCCTATGTAGC
ye515	TTGGACTAGAAATCTCGTGCTGATTAATTGTTTTACGCGTGCGTTTGTGTGGATTGTAGGACAAGGCTCCCTATGTAGC
hu803	TTGGACTAGAAATCTCGTGCTGATTAATTGTTTTACGCGTGCGTTTGTGTGGATTGTAGGACAAGGCTCCCTATGTAGC
ji53	TTGGACTAGAAATCTCGTGCTGATTAATTGTTTTACGCGTGCGTTTGTGTGGATTGTAGGACAAGGCTCCCTATGTAGC
ye832	TTGGACTAGAAATCTCGTGCTGATTAATTGTTTTACGCGTGCGTTTGTGTGGATTGTAGGACAAGGCTCCCTATGTAGC
yu374	TTGGACTAGAAATCTCGTGCTGATTAATTGTTTTACGCGTGCGTTTGTGTGGATTGTAGGACAAGGCTCCCTATGTAGC

Lines	Sequence
D978	TTGGACTAGAAATCTCGTGCTGATTAATTGTTTTACGCGTGCGTTTGTGTGGATTGTAGGACAAGGCTCCCTATGTAGC
Q1261	TTGGACTAGAAATCTCGTGCTGATTAATTGTTTTACGCGTGCGTTTGTGTGGATTGTAGGACAAGGCTCCCTATGTAGC
huotanghuang17	ATGGACTAGAAATCTCGTGCTGATTAATTGTTTTACGCGTGCGTTTGTGTGGATTGTAGGACAAGGCTCCCTATGTAGC
1205A	TTGGACTAGAAATCTCGTGCTGATTAATTGTTTTACGCGTGCGTTTGTGTGGATTGTAGGACAAGGCTCCCTATGTAGC
200B	TTGGACTAGAAATCTCGTGCTGATTAATTGTTTTACGCGTGCGTTTGTGTGGATTGTAGGACAAGGCTCCCTATGTAGC
WN11H	TTGGACTAGAAATCTCGTGCTGATTAATTGTTTTACGCGTGCGTTTGTGTGGATTGTAGGACAAGGCTCCCTATGTAGC
98F1	TTGGACTAGAAATCTCGTGCTGATTAATTGTTTTACGCGTGCGTTTGTGTGGATTGTAGGACAAGGCTCCCTATGTAGC
GY3	TTGGACTAGAAATCTCGTGCTGATTAATTGTTTTACGCGTGCGTTTGTGTGGATTGTAGGACAAGGCTCCCTATGTAGC
4936	TTGGACTAGAAATCTCGTGCTGATTAATTGTTTTACGCGTGCGTTTGTGTGGATTGTAGGACAAGGCTCCCTATGTAGC
DF32	TTGGACTAGAAATCTCGTGCTGATTAATTGTTTTACGCGTGCGTTTGTGTGGATTGTAGGACAAGGCTCCCTATGTAGC
chong72	TTGGACTAGAAATCTCGTGCTGATTAATTGTTTTACGCGTGCGTTTGTGTGGATTGTAGGACAAGGCTCCCTATGTAGC
P25	TTGGACTAGAAATCTCGTGCTGATTAATTGTTTTACGCGTGCGTTTGTGTGGATTGTAGGACAAGGCTCCCTATGTAGC
SS99	TTGGACTAGAAATCTCGTGCTGATTAATTGTTTTACGCGTGCGTTTGTGTGGATTGTAGGACAAGGCTCCCTATGTATC
BM	TTGGACTAGAAATCTCGTGCTGATTAATTGTTTTACGCGTGCGTTTGTGTGGATTGTAGGACAAGGCTCCCTATGTAGC
d140	TTGGACTAGAAATCTCGTGCTGATTAATTGTTTTACGCGTGCGTTTGTGTGGATTGTAGGACAAGGCTCCCTATGTAGC
5003	TTGGACTAGAAATCTCGTGCTGATTAATTGTTTTACGCGTGCGTTTGTGTGGATTGTAGGACAAGGCTCCCTATGTAGC
20837	TTGGACTAGAAATCTCGTGCTGATTAATTGTTTTACGCGTGCGTTTGTGTGGATTGTAGGACAAGGCTCCCTATGTAGC
Los-6	TTGGACTAGAAATCTCGTGCTGATTAATTGTTTTACGCGTGCGTTTGTGTGGATTGTAGGACAAGGCTCCCTATGTAGC
XF27	ATGGACTAGAAATCTCGTGCTGATTAATTGTTTTACGCGTGCGTTTGTGTGGATTGTAGGACAAGGCTCCCTATGTAGC
huo17	TTGGACTAGAAATCTCGTGCTGATTAATTGTTTTACGCGTGCGTTTGTGTGGATTGTAGGACAAGGCTCCCTATGTAGC
jiutai1	TTGGACTAGAAATCTCGTGCTGATTAATTGTTTTACGCGTGCGTTTGTGTGGATTGTAGGACAAGGCTCCCTATGTAGC
SC14	TTGGACTAGAAATCTCGTGCTGATTAATTGTTTTACGCGTGCGTTTGTGTGGATTGTAGGACAAGGCTCCCTATGTAGC

**Lines**	**Sequence**
DH65232	TTGGACTAGAAATCTCGTGCTGATTAATTGTTTTACGCGTGCGTTTGTGTGGATTGTAGGACAAGGCTCCCTATGTAGC
ji533	TTGGACTAGAAATCTCGTGCTGATTAATTGTTTTACGCGTGCGTTTGTGTGGATTGTAGGACAAGGCTCCCTATGTAGC
8982	TTGGACTAGAAATCTCGTGCTGATTAATTGTTTTACGCGTGCGTTTGTGTGGATTGTAGGACAAGGCTCCCTATGTAGC
D23	TTGGACTAGAAATCTCGTGCTGATTAATTGTTTTACGCGTGCGTTTGTGTGGATTGTAGGACAAGGCTCCCTATGTAGC
1127	TTGGACTAGAAATCTCGTGCTGATTAATTGTTTTACGCGTGCGTTTGTGTGGATTGTAGGACAAGGCTCCCTATGTAGC
P167	TTGGACTAGAAATCTCGTGCTGATTAATTGTTTTACGCGTGCGTTTGTGTGGATTGTAGGACAAGGCTCCCTATGTAGC
17564	TTGGACTAGAAATCTCGTGCTGATTAATTGTTTTACGCGTGCGTTTGTGTGGATTGTAGGACAAGGCTCCCTATGTAGC
M3	TTGGACTAGAAATCTCGTGCTGATTAATTGTTTTACGCGTGCGTTTGTGTGGATTGTAGGACAAGGCTCCCTATGTAGC
9702	TTGGACTAGAAATCTCGTGCTGATTAATTGTTTTACGCGTGCGTTTGTGTGGATTGTAGGACAAGGCTCCCTATGTAGC
9711	TTGGACTAGAAATCTCGTGCTGATTAATTGTTTTACGCGTGCGTTTGTGTGGATTGTAGGACAAGGCTCCCTATGTAGC
1101	ATGGACTAGAAATCTCGTGCTGATTAATTGTTTTACGCGTGCGTTTGTGTGGATTGTAGGACAAGGCTCCCTATGTAGC
3189	TTGGACTAGAAATCTCGTGCTGATTAATTGTTTTACGCGTGCGTTTGTGTGGATTGTAGGACAAGGCTCCCTATGTAGC
LD61	TTGGACTAGAAATCTCGTGCTGATTAATTGTTTTACGCGTGCGTTTGTGTGGATTGTAGGACAAGGCTCCCTATGTAGC
wu126	TTGGACTAGAAATCTCGTGCTGATTAATTGTTTTACGCGTGCGTTTGTGTGGATTGTAGGACAAGGCTCCCTATGTAGC
W344	TTGGACTAGAAATCTCGTGCTGATTAATTGTTTTACGCGTGCGTTTGTGTGGATTGTAGGACAAGGCTCCCTATGTAGC
S53	TTGGACTAGAAATCTCGTGCTGATTAATTGTTTTACGCGTGCGTTTGTGTGGATTGTAGGACAAGGCTCCCTATGTAGC
5023	TTGGACTAGAAATCTCGTGCTGATTAATTGTTTTACGCGTGCGTTTGTGTGGATTGTAGGACAAGGCTCCCTATGTAGC
zi330	TTGGACTAGAAATCTCGTGCTGATTAATTGTTTTACGCGTGCGTTTGTGTGGATTGTAGGACAAGGCTCCCTATGTAGC
siyi	TTGGACTAGAAATCTCGTGCTGATTAATTGTTTTACGCGTGCGTTTGTGTGGATTGTAGGACAAGGCTCCCTATGTAGC
F7	TTGGACTAGAAATCTCGTGCTGATTAATTGTTTTACGCGTGCGTTTGTGTGGATTGTAGGACAAGGCTCCCTATGTATC
xun971	TTGGACTAGAAATCTCGTGCTGATTAATTGTTTTACGCGTGCGTTTGTGTGGATTGTAGGACAAGGCTCCCTATGTAGC
OH7	TTGGACTAGAAATCTCGTGCTGATTAATTGTTTTACGCGTGCGTTTGTGTGGATTGTAGGACAAGGCTCCCTATGTAGC

**Lines**	**Sequence**
ND246	ATGGACTAGAAATCTCGTGCTGATTAATTGTTTTACGCGTGCGTTTGTGTGGATTGTAGGACAAGGCTCCCTATGTAGC
CM105	TTGGACTAGAAATCTCGTGCTGATTAATTGTTTTACGCGTGCGTTTGTGTGGATTGTAGGACAAGGCTCCCTATGTAGC
B68	TTGGACTAGAAATCTCGTGCTGATTAATTGTTTTACGCGTGCGTTTGTGTGGATTGTAGGACAAGGCTCCCTATGTAGC
Co109	ATGGACTAGAAATCTCGTGCTGATTAATTGTTTTACGCGTGCGTTTGTGTGGATTGTAGGACAAGGCTCCCTATGTAGC
W182bn	TTGGACTAGAAATCTCGTGCTGATTAATTGTTTTACGCGTGCGTTTGTGTGGATTGTAGGACAAGGCTCCCTATGTAGC
W117	TTGGACTAGAAATCTCGTGCTGATTAATTGTTTTACGCGTGCGTTTGTGTGGATTGTAGGACAAGGCTCCCTATGTAGC
H84	TTGGACTAGAAATCTCGTGCTGATTAATTGTTTTACGCGTGCGTTTGTGTGGATTGTAGGACAAGGCTCCCTATGTAGC
P39	ATGGACTAGAAATCTCGTGCTGATTAATTGTTTTACGCGTGCGTTTGTGTGGATTGTAGGACAAGGCTCCCTATGTAGC
CT52C	TTGGACTAGAAATCTCGTGCTGATTAATTGTTTTACGCGTGCGTTTGTGTGGATTGTAGGACAAGGCTCCCTATGTAGC
jiao51	TTGGACTAGAAATCTCGTGCTGATTAATTGTTTTACGCGTGCGTTTGTGTGGATTGTAGGACAAGGCTCCCTATGTAGC
77	TTGGACTAGAAATCTCGTGCTGATTAATTGTTTTACGCGTGCGTTTGTGTGGATTGTAGGACAAGGCTCCCTATGTAGC
7327	TTGGACTAGAAATCTCGTGCTGATTAATTGTTTTACGCGTGCGTTTGTGTGGATTGTAGGACAAGGCTCCCTATGTAGC
daqing133	TTGGACTAGAAATCTCGTGCTGATTAATTGTTTTACGCGTGCGTTTGTGTGGATTGTAGGACAAGGCTCCCTATGTAGC
mu6	TTGGACTAGAAATCTCGTGCTGATTAATTGTTTTACGCGTGCGTTTGTGTGGATTGTAGGACAAGGCTCCCTATGTAGC
mu4	TTGGACTAGAAATCTCGTGCTGATTAATTGTTTTACGCGTGCGTTTGTGTGGATTGTAGGACAAGGCTCCCTATGTAGC
yu537A	TTGGACTAGAAATCTCGTGCTGATTAATTGTTTTACGCGTGCGTTTGTGTGGATTGTAGGACAAGGCTCCCTATGTAGC
yu82	TTGGACTAGAAATCTCGTGCTGATTAATTGTTTTACGCGTGCGTTTGTGTGGATTGTAGGACAAGGCTCCCTATGTAGC
E28	TTGGACTAGAAATCTCGTGCTGATTAATTGTTTTACGCGTGCGTTTGTGTGGATTGTAGGACAAGGCTCCCTATGTAGC
fanrong2	ATGGACTAGAAATCTCGTGCTGATTAATTGTTTTACGCGTGCGTTTGTGTGGATTGTAGGACAAGGCTCCCTATGTAGC
zhong451	TTGGACTAGAAATCTCGTGCTGATTAATTGTTTTACGCGTGCGTTTGTGTGGATTGTAGGACAAGGCTCCCTATGTAGC
M3736	TTGGACTAGAAATCTCGTGCTGATTAATTGTTTTACGCGTGCGTTTGTGTGGATTGTAGGACAAGGCTCCCTATGTAGC
jingnuo2	ATGGACTAGAAATCTCGTGCTGATTAATTGTTTTACGCGTGCGTTTGTGTGGATTGTAGGACAAGGCTCCCTATGTAGC

Lines	Sequence
song1145	TTGGACTAGAAATCTCGTGCTGATTAATTGTTTTACGCGTGCGTTTGTGTGGATTGTAGGACAAGGCTCCCTATGTAGC
zun90110	TTGGACTAGAAATCTCGTGCTGATTAATTGTTTTACGCGTGCGTTTGTGTGGATTGTAGGACAAGGCTCCCTATGTAGC
cheng435	TTGGACTAGAAATCTCGTGCTGATTAATTGTTTTACGCGTGCGTTTGTGTGGATTGTAGGACAAGGCTCCCTATGTAGC
79028	TTGGACTAGAAATCTCGTGCTGATTAATTGTTTTACGCGTGCGTTTGTGTGGATTGTAGGACAAGGCTCCCTATGTAGC
H2	ATGGACTAGAAATCTCGTGCTGATTAATTGTTTTACGCGTGCGTTTGTGTGGATTGTAGGACAAGGCTCCCTATGTAGC
tai184	TTGGACTAGAAATCTCGTGCTGATTAATTGTTTTACGCGTGCGTTTGTGTGGATTGTAGGACAAGGCTCCCTATGTAGC
ziduosui	TTGGACTAGAAATCTCGTGCTGATTAATTGTTTTACGCGTGCGTTTGTGTGGATTGTAGGACAAGGCTCCCTATGTAGC
xuan6	TTGGACTAGAAATCTCGTGCTGATTAATTGTTTTACGCGTGCGTTTGTGTGGATTGTAGGACAAGGCTCCCTATGTAGC
lan766-4-2	ATGGACTAGAAATCTCGTGCTGATTAATTGTTTTACGCGTGCGTTTGTGTGGATTGTAGGACAAGGCTCCCTATGTAGC
chi545	ATGGACTAGAAATCTCGTGCTGATTAATTGTTTTACGCGTGCGTTTGTGTGGATTGTAGGACAAGGCTCCCTATGTAGC
85bai64	TTGGACTAGAAATCTCGTGCTGATTAATTGTTTTACGCGTGCGTTTGTGTGGATTGTAGGACAAGGCTCCCTATGTAGC
91huang5	TTGGACTAGAAATCTCGTGCTGATTAATTGTTTTACGCGTGCGTTTGTGTGGATTGTAGGACAAGGCTCCCTATGTAGC
91huang10	TTGGACTAGAAATCTCGTGCTGATTAATTGTTTTACGCGTGCGTTTGTGTGGATTGTAGGACAAGGCTCCCTATGTAGC
chihuang14	TTGGACTAGAAATCTCGTGCTGATTAATTGTTTTACGCGTGCGTTTGTGTGGATTGTAGGACAAGGCTCCCTATGTAGC
chihuang32	TTGGACTAGAAATCTCGTGCTGATTAATTGTTTTACGCGTGCGTTTGTGTGGATTGTAGGACAAGGCTCCCTATGTAGC
785	TTGGACTAGAAATCTCGTGCTGATTAATTGTTTTACGCGTGCGTTTGTGTGGATTGTAGGACAAGGCTCCCTATGTAGC
liao4271	TTGGACTAGAAATCTCGTGCTGATTAATTGTTTTACGCGTGCGTTTGTGTGGATTGTAGGACAAGGCTCCCTATGTAGC
fu96	TTGGACTAGAAATCTCGTGCTGATTAATTGTTTTACGCGTGCGTTTGTGTGGATTGTAGGACAAGGCTCCCTATGTAGC
ji833	TTGGACTAGAAATCTCGTGCTGATTAATTGTTTTACGCGTGCGTTTGTGTGGATTGTAGGACAAGGCTCCCTATGTAGC
ji870	ATGGACTAGAAATCTCGTGCTGATTAATTGTTTTACGCGTGCGTTTGTGTGGATTGTAGGACAAGGCTCCCTATGTAGC
fu8521	TTGGACTAGAAATCTCGTGCTGATTAATTGTTTTACGCGTGCGTTTGTGTGGATTGTAGGACAAGGCTCCCTATGTAGC
fu8527	TTGGACTAGAAATCTCGTGCTGATTAATTGTTTTACGCGTGCGTTTGTGTGGATTGTAGGACAAGGCTCCCTATGTAGC

**Lines**	**Sequence**
fu8538	TTGGACTAGAAATCTCGTGCTGATTAATTGTTTTACGCGTGCGTTTGTGTGGATTGTAGGACAAGGCTCCCTATGTAGC
longkang1	TTGGACTAGAAATCTCGTGCTGATTAATTGTTTTACGCGTGCGTTTGTGTGGATTGTAGGACAAGGCTCCCTATGTAGC
yan172	TTGGACTAGAAATCTCGTGCTGATTAATTGTTTTACGCGTGCGTTTGTGTGGATTGTAGGACAAGGCTCCCTATGTAGC
dai6	TTGGACTAGAAATCTCGTGCTGATTAATTGTTTTACGCGTGCGTTTGTGTGGATTGTAGGACAAGGCTCCCTATGTAGC
qi410	ATGGACTAGAAATCTCGTGCTGATTAATTGTTTTACGCGTGCGTTTGTGTGGATTGTAGGACAAGGCTCCCTATGTAGC
32	TTGGACTAGAAATCTCGTGCTGATTAATTGTTTTACGCGTGCGTTTGTGTGGATTGTAGGACAAGGCTCCCTATGTAGC
yue39-4	TTGGACTAGAAATCTCGTGCTGATTAATTGTTTTACGCGTGCGTTTGTGTGGATTGTAGGACAAGGCTCCCTATGTAGC
Cwu215B	TTGGACTAGAAATCTCGTGCTGATTAATTGTTTTACGCGTGCGTTTGTGTGGATTGTAGGACAAGGCTCCCTATGTAGC
SZ3	ATGGACTAGAAATCTCGTGCTGATTAATTGTTTTACGCGTGCGTTTGTGTGGATTGTAGGACAAGGCTCCCTATGTAGC
S001	TTGGACTAGAAATCTCGTGCTGATTAATTGTTTTACGCGTGCGTTTGTGTGGATTGTAGGACAAGGCTCCCTATGTAGC
wei3322	TTGGACTAGAAATCTCGTGCTGATTAATTGTTTTACGCGTGCGTTTGTGTGGATTGTAGGACAAGGCTCCCTATGTAGC
ning45	TTGGACTAGAAATCTCGTGCTGATTAATTGTTTTACGCGTGCGTTTGTGTGGATTGTAGGACAAGGCTCCCTATGTAGC
ning55	TTGGACTAGAAATCTCGTGCTGATTAATTGTTTTACGCGTGCGTTTGTGTGGATTGTAGGACAAGGCTCCCTATGTAGC
H66/6	TTGGACTAGAAATCTCGTGCTGATTAATTGTTTTACGCGTGCGTTTGTGTGGATTGTAGGACAAGGCTCCCTATGTAGC
72-125	TTGGACTAGAAATCTCGTGCTGATTAATTGTTTTACGCGTGCGTTTGTGTGGATTGTAGGACAAGGCTCCCTATGTAGC
C103	TTGGACTAGAAATCTCGTGCTGATTAATTGTTTTACGCGTGCGTTTGTGTGGATTGTAGGACAAGGCTCCCTATGTAGC
hua160	ATGGACTAGAAATCTCGTGCTGATTAATTGTTTTACGCGTGCGTTTGTGTGGATTGTAGGACAAGGCTCCCTATGTAGC
ES40	TTGGACTAGAAATCTCGTGCTGATTAATTGTTTTACGCGTGCGTTTGTGTGGATTGTAGGACAAGGCTCCCTATGTAGC
78002A	TTGGACTAGAAATCTCGTGCTGATTAATTGTTTTACGCGTGCGTTTGTGTGGATTGTAGGACAAGGCTCCCTATGTAGC
B47	ATGGACTAGAAATCTCGTGCTGATTAATTGTTTTACGCGTGCGTTTGTGTGGATTGTAGGACAAGGCTCCCTATGTAGC
LH156	TTGGACTAGAAATCTCGTGCTGATTAATTGTTTTACGCGTGCGTTTGTGTGGATTGTAGGACAAGGCTCCCTATGTAGC
PHZ51	TTGGACTAGAAATCTCGTGCTGATTAATTGTTTTACGCGTGCGTTTGTGTGGATTGTAGGACAAGGCTCCCTATGTAGC

Lines	Sequence
LPINRHT	TTGGACTAGAAATCTCGTGCTGATTAATTGTTTTACGCGTGCGTTTGTGTGGATTGTAGGACAAGGCTCCCTATGTAGC
PHJ75	TTGGACTAGAAATCTCGTGCTGATTAATTGTTTTACGCGTGCGTTTGTGTGGATTGTAGGACAAGGCTCCCTATGTAGC
PHM57	ATGGACTAGAAATCTCGTGCTGATTAATTGTTTTACGCGTGCGTTTGTGTGGATTGTAGGACAAGGCTCCCTATGTAGC
PHW03	ATGGACTAGAAATCTCGTGCTGATTAATTGTTTTACGCGTGCGTTTGTGTGGATTGTAGGACAAGGCTCCCTATGTAGC
PHK05	ATGGACTAGAAATCTCGTGCTGATTAATTGTTTTACGCGTGCGTTTGTGTGGATTGTAGGACAAGGCTCCCTATGTAGC
PHR25	ATGGACTAGAAATCTCGTGCTGATTAATTGTTTTACGCGTGCGTTTGTGTGGATTGTAGGACAAGGCTCCCTATGTAGC
LH60	TTGGACTAGAAATCTCGTGCTGATTAATTGTTTTACGCGTGCGTTTGTGTGGATTGTAGGACAAGGCTCCCTATGTAGC
PHK76	TTGGACTAGAAATCTCGTGCTGATTAATTGTTTTACGCGTGCGTTTGTGTGGATTGTAGGACAAGGCTCCCTATGTAGC
PHG35	TTGGACTAGAAATCTCGTGCTGATTAATTGTTTTACGCGTGCGTTTGTGTGGATTGTAGGACAAGGCTCCCTATGTAGC
LH59	TTGGACTAGAAATCTCGTGCTGATTAATTGTTTTACGCGTGCGTTTGTGTGGATTGTAGGACAAGGCTCCCTATGTAGC
LH123HT	TTGGACTAGAAATCTCGTGCTGATTAATTGTTTTACGCGTGCGTTTGTGTGGATTGTAGGACAAGGCTCCCTATGTAGC
LH38	TTGGACTAGAAATCTCGTGCTGATTAATTGTTTTACGCGTGCGTTTGTGTGGATTGTAGGACAAGGCTCCCTATGTAGC
PHK42	ATGGACTAGAAATCTCGTGCTGATTAATTGTTTTACGCGTGCGTTTGTGTGGATTGTAGGACAAGGCTCCCTATGTAGC
78010	TTGGACTAGAAATCTCGTGCTGATTAATTGTTTTACGCGTGCGTTTGTGTGGATTGTAGGACAAGGCTCCCTATGTAGC
HB8229	TTGGACTAGAAATCTCGTGCTGATTAATTGTTTTACGCGTGCGTTTGTGTGGATTGTAGGACAAGGCTCCCTATGTAGC
PHR32	ATGGACTAGAAATCTCGTGCTGATTAATTGTTTTACGCGTGCGTTTGTGTGGATTGTAGGACAAGGCTCCCTATGTAGC
PHT10	TTGGACTAGAAATCTCGTGCTGATTAATTGTTTTACGCGTGCGTTTGTGTGGATTGTAGGACAAGGCTCCCTATGTAGC
LH1	TTGGACTAGAAATCTCGTGCTGATTAATTGTTTTACGCGTGCGTTTGTGTGGATTGTAGGACAAGGCTCCCTATGTAGC
790	TTGGACTAGAAATCTCGTGCTGATTAATTGTTTTACGCGTGCGTTTGTGTGGATTGTAGGACAAGGCTCCCTATGTAGC
PHG47	TTGGACTAGAAATCTCGTGCTGATTAATTGTTTTACGCGTGCGTTTGTGTGGATTGTAGGACAAGGCTCCCTATGTAGC
LH39	TTGGACTAGAAATCTCGTGCTGATTAATTGTTTTACGCGTGCGTTTGTGTGGATTGTAGGACAAGGCTCCCTATGTAGC
LH127	TTGGACTAGAAATCTCGTGCTGATTAATTGTTTTACGCGTGCGTTTGTGTGGATTGTAGGACAAGGCTCCCTATGTAGC

**Lines**	**Sequence**
LH191	TTGGACTAGAAATCTCGTGCTGATTAATTGTTTTACGCGTGCGTTTGTGTGGATTGTAGGACAAGGCTCCCTATGTAGC
LH193	TTGGACTAGAAATCTCGTGCTGATTAATTGTTTTACGCGTGCGTTTGTGTGGATTGTAGGACAAGGCTCCCTATGTAGC
RS710	TTGGACTAGAAATCTCGTGCTGATTAATTGTTTTACGCGTGCGTTTGTGTGGATTGTAGGACAAGGCTCCCTATGTAGC
BCC03	ATGGACTAGAAATCTCGTGCTGATTAATTGTTTTACGCGTGCGTTTGTGTGGATTGTAGGACAAGGCTCCCTATGTAGC
LH128	TTGGACTAGAAATCTCGTGCTGATTAATTGTTTTACGCGTGCGTTTGTGTGGATTGTAGGACAAGGCTCCCTATGTAGC
PHM81	ATGGACTAGAAATCTCGTGCTGATTAATTGTTTTACGCGTGCGTTTGTGTGGATTGTAGGACAAGGCTCCCTATGTAGC
PHR55	TTGGACTAGAAATCTCGTGCTGATTAATTGTTTTACGCGTGCGTTTGTGTGGATTGTAGGACAAGGCTCCCTATGTAGC
PHP85	TTGGACTAGAAATCTCGTGCTGATTAATTGTTTTACGCGTGCGTTTGTGTGGATTGTAGGACAAGGCTCCCTATGTAGC
6103	TTGGACTAGAAATCTCGTGCTGATTAATTGTTTTACGCGTGCGTTTGTGTGGATTGTAGGACAAGGCTCCCTATGTAGC
MBNA	TTGGACTAGAAATCTCGTGCTGATTAATTGTTTTACGCGTGCGTTTGTGTGGATTGTAGGACAAGGCTCCCTATGTAGC
4676A	ATGGACTAGAAATCTCGTGCTGATTAATTGTTTTACGCGTGCGTTTGTGTGGATTGTAGGACAAGGCTCCCTATGTAGC
IB02	TTGGACTAGAAATCTCGTGCTGATTAATTGTTTTACGCGTGCGTTTGTGTGGATTGTAGGACAAGGCTCCCTATGTAGC
787	ATGGACTAGAAATCTCGTGCTGATTAATTGTTTTACGCGTGCGTTTGTGTGGATTGTAGGACAAGGCTCCCTATGTAGC
11430	TTGGACTAGAAATCTCGTGCTGATTAATTGTTTTACGCGTGCGTTTGTGTGGATTGTAGGACAAGGCTCCCTATGTAGC
2MA22	TTGGACTAGAAATCTCGTGCTGATTAATTGTTTTACGCGTGCGTTTGTGTGGATTGTAGGACAAGGCTCCCTATGTAGC
6M502	TTGGACTAGAAATCTCGTGCTGATTAATTGTTTTACGCGTGCGTTTGTGTGGATTGTAGGACAAGGCTCCCTATGTAGC
87916W	TTGGACTAGAAATCTCGTGCTGATTAATTGTTTTACGCGTGCGTTTGTGTGGATTGTAGGACAAGGCTCCCTATGTAGC
PHN47	ATGGACTAGAAATCTCGTGCTGATTAATTGTTTTACGCGTGCGTTTGTGTGGATTGTAGGACAAGGCTCCCTATGTAGC
NS501	ATGGACTAGAAATCTCGTGCTGATTAATTGTTTTACGCGTGCGTTTGTGTGGATTGTAGGACAAGGCTCCCTATGTAGC
H8431	TTGGACTAGAAATCTCGTGCTGATTAATTGTTTTACGCGTGCGTTTGTGTGGATTGTAGGACAAGGCTCCCTATGTAGC
S8324	TTGGACTAGAAATCTCGTGCTGATTAATTGTTTTACGCGTGCGTTTGTGTGGATTGTAGGACAAGGCTCCCTATGTAGC
WIL500	TTGGACTAGAAATCTCGTGCTGATTAATTGTTTTACGCGTGCGTTTGTGTGGATTGTAGGACAAGGCTCCCTATGTAGC

Lines	Sequence
E8501	TTGGACTAGAAATCTCGTGCTGATTAATTGTTTTACGCGTGCGTTTGTGTGGATTGTAGGACAAGGCTCCCTATGTAGC
J8606	TTGGACTAGAAATCTCGTGCTGATTAATTGTTTTACGCGTGCGTTTGTGTGGATTGTAGGACAAGGCTCCCTATGTAGC
4N506	TTGGACTAGAAATCTCGTGCTGATTAATTGTTTTACGCGTGCGTTTGTGTGGATTGTAGGACAAGGCTCCCTATGTAGC
PHJ33	TTGGACTAGAAATCTCGTGCTGATTAATTGTTTTACGCGTGCGTTTGTGTGGATTGTAGGACAAGGCTCCCTATGTAGC
PHN82	TTGGACTAGAAATCTCGTGCTGATTAATTGTTTTACGCGTGCGTTTGTGTGGATTGTAGGACAAGGCTCCCTATGTAGC
PHP55	TTGGACTAGAAATCTCGTGCTGATTAATTGTTTTACGCGTGCGTTTGTGTGGATTGTAGGACAAGGCTCCCTATGTAGC
PHT22	TTGGACTAGAAATCTCGTGCTGATTAATTGTTTTACGCGTGCGTTTGTGTGGATTGTAGGACAAGGCTCCCTATGTAGC
W23	TTGGACTAGAAATCTCGTGCTGATTAATTGTTTTACGCGTGCGTTTGTGTGGATTGTAGGACAAGGCTCCCTATGTAGC
478	TTGGACTAGAAATCTCGTGCTGATTAATTGTTTTACGCGTGCGTTTGTGTGGATTGTAGGACAAGGCTCCCTATGTAGC
D340	TTGGACTAGAAATCTCGTGCTGATTAATTGTTTTACGCGTGCGTTTGTGTGGATTGTAGGACAAGGCTCCCTATGTAGC
He344	TTGGACTAGAAATCTCGTGCTGATTAATTGTTTTACGCGTGCGTTTGTGTGGATTGTAGGACAAGGCTCCCTATGTAGC
F939	TTGGACTAGAAATCTCGTGCTGATTAATTGTTTTACGCGTGCGTTTGTGTGGATTGTAGGACAAGGCTCCCTATGTAGC
Luyuan133	ATGGACTAGAAATCTCGTGCTGATTAATTGTTTTACGCGTGCGTTTGTGTGGATTGTAGGACAAGGCTCCCTATGTAGC
778	TTGGACTAGAAATCTCGTGCTGATTAATTGTTTTACGCGTGCGTTTGTGTGGATTGTAGGACAAGGCTCCCTATGTAGC
LH146HT	TTGGACTAGAAATCTCGTGCTGATTAATTGTTTTACGCGTGCGTTTGTGTGGATTGTAGGACAAGGCTCCCTATGTAGC
PHR62	TTGGACTAGAAATCTCGTGCTGATTAATTGTTTTACGCGTGCGTTTGTGTGGATTGTAGGACAAGGCTCCCTATGTAGC
B84	TTGGACTAGAAATCTCGTGCTGATTAATTGTTTTACGCGTGCGTTTGTGTGGATTGTAGGACAAGGCTCCCTATGTAGC
B87	ATGGACTAGAAATCTCGTGCTGATTAATTGTTTTACGCGTGCGTTTGTGTGGATTGTAGGACAAGGCTCCCTATGTAGC
ILL12E	TTGGACTAGAAATCTCGTGCTGATTAATTGTTTTACGCGTGCGTTTGTGTGGATTGTAGGACAAGGCTCCCTATGTAGC
黑龙江红玉米	TTGGACTAGAAATCTCGTGCTGATTAATTGTTTTACGCGTGCGTTTGTGTGGATTGTAGGACAAGGCTCCCTATGTAGC
Dian11	TTGGACTAGAAATCTCGTGCTGATTAATTGTTTTACGCGTGCGTTTGTGTGGATTGTAGGACAAGGCTCCCTATGTAGC
HR295	TTGGACTAGAAATCTCGTGCTGATTAATTGTTTTACGCGTGCGTTTGTGTGGATTGTAGGACAAGGCTCCCTATGTAGC

Lines	Sequence
2001-F22	TTGGACTAGAAATCTCGTGCTGATTAATTGTTTTACGCGTGCGTTTGTGTGGATTGTAGGACAAGGCTCCCTATGTAGC
KN3	TTGGACTAGAAATCTCGTGCTGATTAATTGTTTTACGCGTGCGTTTGTGTGGATTGTAGGACAAGGCTCCCTATGTAGC
M5911	TTGGACTAGAAATCTCGTGCTGATTAATTGTTTTACGCGTGCGTTTGTGTGGATTGTAGGACAAGGCTCCCTATGTAGC
HR78	TTGGACTAGAAATCTCGTGCTGATTAATTGTTTTACGCGTGCGTTTGTGTGGATTGTAGGACAAGGCTCCCTATGTAGC
中农大 788-父本	TTGGACTAGAAATCTCGTGCTGATTAATTGTTTTACGCGTGCGTTTGTGTGGATTGTAGGACAAGGCTCCCTATGTAGC
SC-14	TTGGACTAGAAATCTCGTGCTGATTAATTGTTTTACGCGTGCGTTTGTGTGGATTGTAGGACAAGGCTCCCTATGTAGC
XF117	ATGGACTAGAAATCTCGTGCTGATTAATTGTTTTACGCGTGCGTTTGTGTGGATTGTAGGACAAGGCTCCCTATGTAGC
XF197	TTGGACTAGAAATCTCGTGCTGATTAATTGTTTTACGCGTGCGTTTGTGTGGATTGTAGGACAAGGCTCCCTATGTAGC
C8605-2	TTGGACTAGAAATCTCGTGCTGATTAATTGTTTTACGCGTGCGTTTGTGTGGATTGTAGGACAAGGCTCCCTATGTAGC
沿 812	TTGGACTAGAAATCTCGTGCTGATTAATTGTTTTACGCGTGCGTTTGTGTGGATTGTAGGACAAGGCTCCCTATGTAGC
D881	TTGGACTAGAAATCTCGTGCTGATTAATTGTTTTACGCGTGCGTTTGTGTGGATTGTAGGACAAGGCTCCCTATGTAGC
Z31B	ATGGACTAGAAATCTCGTGCTGATTAATTGTTTTACGCGTGCGTTTGTGTGGATTGTAGGACAAGGCTCCCTATGTAGC
齐 319	TTGGACTAGAAATCTCGTGCTGATTAATTGTTTTACGCGTGCGTTTGTGTGGATTGTAGGACAAGGCTCCCTATGTAGC
441950	ATGGACTAGAAATCTCGTGCTGATTAATTGTTTTACGCGTGCGTTTGTGTGGATTGTAGGACAAGGCTCCCTATGTAGC
802	TTGGACTAGAAATCTCGTGCTGATTAATTGTTTTACGCGTGCGTTTGTGTGGATTGTAGGACAAGGCTCCCTATGTAGC
68202	TTGGACTAGAAATCTCGTGCTGATTAATTGTTTTACGCGTGCGTTTGTGTGGATTGTAGGACAAGGCTCCCTATGTAGC
黄野四	TTGGACTAGAAATCTCGTGCTGATTAATTGTTTTACGCGTGCGTTTGTGTGGATTGTAGGACAAGGCTCCCTATGTAGC
Ay3566	TTGGACTAGAAATCTCGTGCTGATTAATTGTTTTACGCGTGCGTTTGTGTGGATTGTAGGACAAGGCTCCCTATGTAGC
2005	TTGGACTAGAAATCTCGTGCTGATTAATTGTTTTACGCGTGCGTTTGTGTGGATTGTAGGACAAGGCTCCCTATGTAGC
凤可 1	TTGGACTAGAAATCTCGTGCTGATTAATTGTTTTACGCGTGCGTTTGTGTGGATTGTAGGACAAGGCTCCCTATGTAGC
宗 548-1521	TTGGACTAGAAATCTCGTGCTGATTAATTGTTTTACGCGTGCGTTTGTGTGGATTGTAGGACAAGGCTCCCTATGTAGC
Timpunia-1	TTGGACTAGAAATCTCGTGCTGATTAATTGTTTTACGCGTGCGTTTGTGTGGATTGTAGGACAAGGCTCCCTATGTAGC

Lines	Sequence
H114	TTGGACTAGAAATCTCGTGCTGATTAATTGTTTTACGCGTGCGTTTGTGTGGATTGTAGGACAAGGCTCCCTATGTAGC
PHJ70	TTGGACTAGAAATCTCGTGCTGATTAATTGTTTTACGCGTGCGTTTGTGTGGATTGTAGGACAAGGCTCCCTATGTAGC
LH160	ATGGACTAGAAATCTCGTGCTGATTAATTGTTTTACGCGTGCGTTTGTGTGGATTGTAGGACAAGGCTCCCTATGTAGC
MBPM	TTGGACTAGAAATCTCGTGCTGATTAATTGTTTTACGCGTGCGTTTGTGTGGATTGTAGGACAAGGCTCCCTATGTAGC
LH82	TTGGACTAGAAATCTCGTGCTGATTAATTGTTTTACGCGTGCGTTTGTGTGGATTGTAGGACAAGGCTCCCTATGTAGC
78371A	TTGGACTAGAAATCTCGTGCTGATTAATTGTTTTACGCGTGCGTTTGTGTGGATTGTAGGACAAGGCTCCCTATGTAGC
PHR47	TTGGACTAGAAATCTCGTGCTGATTAATTGTTTTACGCGTGCGTTTGTGTGGATTGTAGGACAAGGCTCCCTATGTAGC
LH194	TTGGACTAGAAATCTCGTGCTGATTAATTGTTTTACGCGTGCGTTTGTGTGGATTGTAGGACAAGGCTCCCTATGTAGC
MBUB	TTGGACTAGAAATCTCGTGCTGATTAATTGTTTTACGCGTGCGTTTGTGTGGATTGTAGGACAAGGCTCCCTATGTAGC
NS701	TTGGACTAGAAATCTCGTGCTGATTAATTGTTTTACGCGTGCGTTTGTGTGGATTGTAGGACAAGGCTCCCTATGTAGC
PHN37	TTGGACTAGAAATCTCGTGCTGATTAATTGTTTTACGCGTGCGTTTGTGTGGATTGTAGGACAAGGCTCCCTATGTAGC
PHW20	TTGGACTAGAAATCTCGTGCTGATTAATTGTTTTACGCGTGCGTTTGTGTGGATTGTAGGACAAGGCTCCCTATGTAGC
2FACC	TTGGACTAGAAATCTCGTGCTGATTAATTGTTTTACGCGTGCGTTTGTGTGGATTGTAGGACAAGGCTCCCTATGTAGC
B78	TTGGACTAGAAATCTCGTGCTGATTAATTGTTTTACGCGTGCGTTTGTGTGGATTGTAGGACAAGGCTCCCTATGTAGC
B127	TTGGACTAGAAATCTCGTGCTGATTAATTGTTTTACGCGTGCGTTTGTGTGGATTGTAGGACAAGGCTCCCTATGTAGC
LH244	TTGGACTAGAAATCTCGTGCTGATTAATTGTTTTACGCGTGCGTTTGTGTGGATTGTAGGACAAGGCTCCCTATGTAGC
P178	TTGGACTAGAAATCTCGTGCTGATTAATTGTTTTACGCGTGCGTTTGTGTGGATTGTAGGACAAGGCTCCCTATGTAGC
Dong237	TTGGACTAGAAATCTCGTGCTGATTAATTGTTTTACGCGTGCGTTTGTGTGGATTGTAGGACAAGGCTCCCTATGTAGC
