# Supplementary material for: High-Throughput and Accurate Determination of Transgene Copy Number and Zygosity in Transgenic Maize: From DNA Extraction to Data Analysis
Source: Int J Mol Sci. 2021 Nov 19;22(22):12487. doi: 10.3390/ijms222212487 (PMC8619409; doi:10.3390/ijms222212487)
Supplement: Supplementary file 1 [file ijms-22-12487-s001.zip › Table S2.pdf]

Table S2. Comparison of *bar* copy number estimated by TaqMan and Southern blot assay for 45 T_0 plants. For TaqMan assay, *bar* copy number was assessed with three repeats (r = 3).

Transgenic Lines	Ratio (target/ref)	Estimated Copy Number by TaqMan Assay	Estimated Copy Number by Southern Blots	Agree (+) or Disagree (-)
Non-transformed	0	0	0	+
12141320	1.64±0.07	2	2	+
12141321	0.95±0.11	1	1	+
12151398	0.95±0.05	1	1	+
12161497	1.09±0.06	1	1	+
12161498	1.13±0.11	1	1	+
12171579	1.11±0.03	1	1	+
13181646	0.98±0.07	1	2	-
13181668	1.19±0.00	1	1	+
13191758	1.08±0.04	1	1	+
13191764	0.89±0.01	1	1	+
13191765	1.17±0.08	1	1	+
13201829	1.03±0.02	1	2	-
13201830	1.04±0.02	1	2	-
12030065	1.06±0.11	1	1	+
12050430	1.09±0.04	1	1	+
12060607	0.87±0.05	1	1	+
12060608	1.04±0.08	1	1	+
12060612	2.56±0.04	3	2	-

Transgenic Lines	Ratio (target/ref)	Estimated Copy Number by TaqMan Assay	Estimated Copy Number by Southern Blots	Agree (+) or Disagree (-)
12060614	1.10±0.09	1	1	+
12070775	1.09±0.03	1	1	+
12080815	1.06±0.03	1	1	+
12141331	1.05±0.04	1	1	+
12161432	1.70±0.14	2	2	+
12030070	1.20±0.09	1	1	+
12040218	1.07±0.12	1	1	+
12040220	1.75±0.10	2	3	-
12050433	0.63±0.06	1	1	+
12060625	1.18±0.17	1	1	+
12060631	1.76±0.12	2	2	+
12101014	0.79±0.06	1	1	+
12111088	1.00±0.05	1	1	+
12030068	0	0	0	+
12030212	1.13±0.09	1	1	+
12030213	1.17±0.07	1	1	+
12030071	4.87±0.38	5	6	+
12030210	1.32±0.05	1	1	+
12030066	5.38±0.21	5	6	+
12030067	2.15±0.11	2	3	-
12010004	2.04±0.06	2	2	+
12010005	1.23±0.02	1	1	+

Transgenic Lines	Ratio (target/ref)	Estimated Copy Number by TaqMan Assay	Estimated Copy Number by Southern Blots	Agree (+) or Disagree (-)
12030084	0	0	0	+
12030085	3.39±0.18	3	3	+
12030086	1.16±0.01	1	1	+
12040234	0.71±0.02	1	1	+
12040236	1.10±0.01	1	1	+

Notes: target, transgene *bar*; ref, endogenous gene *hmg*.
