# Supplementary material for: High-Throughput and Accurate Determination of Transgene Copy Number and Zygosity in Transgenic Maize: From DNA Extraction to Data Analysis
Source: Int J Mol Sci. 2021 Nov 19;22(22):12487. doi: 10.3390/ijms222212487 (PMC8619409; doi:10.3390/ijms222212487)
Supplement: Supplementary file 1 [file ijms-22-12487-s001.zip › Table S3.pdf]

Table S3. Comparison of *bar* copy numbers estimated by TaqMan, dPCR and Southern blot assays for eight T_0 plants. For dPCR assay, the undigested DNA is analyzed and repeated once (r = 1).

**Transgenic Lines**	**Ratio (target/ref)**	**Estimated Copy Number by dPCR**	**Estimated Copy Number by TaqMan Assay**	**Estimated Copy Number by Southern Blots**
12151398	0.94	1	1	1
13181646	1.09	1	1	2
13201830	0.94	1	1	2
12060612	2.67	3	3	2
12080815	1.06	1	1	1
12040220	1.94	2	2	3
12060631	1.86	2	2	2
12040234	0.72	1	1	1

Notes: target, transgene *bar*; ref, endogenous gene *hmg*.
