# Supplementary material for: High-Throughput and Accurate Determination of Transgene Copy Number and Zygosity in Transgenic Maize: From DNA Extraction to Data Analysis
Source: Int J Mol Sci. 2021 Nov 19;22(22):12487. doi: 10.3390/ijms222212487 (PMC8619409; doi:10.3390/ijms222212487)
Supplement: Supplementary file 1 [file ijms-22-12487-s001.zip › Table S4.pdf]

Table S4. Copy numbers of 4,409 T0 samples with three technical repetitions for random sample analysis.

Sample ID	Copy Number							
	r = 1		r = 2		r = 3		Average	
	Ratio (target/ref)	Estimated Copy Number	Ratio (target/ref)	Estimated Copy Number	Ratio (target/ref)	Estimated Copy Number	Ratio (target/ref)	Estimated Copy Number
13030002	1.26	1	1.21	1	1.22	1	1.23	1
13030004	3.45	3	2.74	3	3.21	3	3.13	3
13030005	4.96	5	6.91	7	4.93	5	5.60	6
13030009	1.03	1	0.98	1	0.95	1	0.99	1
13030012	0.03	0	0.02	0	0.02	0	0.03	0
13030013	0.13	0	0.13	0	0.22	0	0.16	0
13030014	0.08	0	0.11	0	0.12	0	0.10	0
13030015	2.76	3	2.51	3	2.78	3	2.68	3
13040005	2.73	3	3.16	3	3.17	3	3.02	3
13040007	0.58	1	0.64	1	0.48	0	0.57	1
13040011	0.02	0	0.02	0	0.00	0	0.01	0
13040012	0.05	0	0.03	0	0.00	0	0.03	0
13040019	1.18	1	1.36	1	0.10	0	0.88	1
13212002	1.34	1	1.07	1	0.96	1	1.12	1
13212003	1.84	2	1.67	2	1.77	2	1.76	2
13217007	1.65	2	1.85	2	1.73	2	1.74	2
13480206	4.11	4	4.84	5	4.39	4	4.45	4

Sample ID	Copy Number							
	r = 1		r = 2		r = 3		Average	
	Ratio (target/ref)	Estimated Copy Number	Ratio (target/ref)	Estimated Copy Number	Ratio (target/ref)	Estimated Copy Number	Ratio (target/ref)	Estimated Copy Number
13480268	5.71	6	4.31	4	4.84	5	4.95	5
13480841	1.26	1	1.19	1	1.14	1	1.20	1
13490037	1.42	1	1.28	1	1.19	1	1.29	1
13490075	3.53	4	3.45	3	3.60	4	3.52	4
13490113	5.34	5	5.60	6	5.19	5	5.38	5
13490195	1.05	1	1.26	1	1.26	1	1.19	1
13500049	6.58	7	5.72	6	5.65	6	5.98	6
13500111	1.06	1	1.15	1	1.15	1	1.12	1
13500132	1.28	1	1.24	1	1.45	1	1.33	1
13500313	4.63	5	4.39	4	4.61	5	4.54	5
13500323	1.17	1	1.17	1	1.27	1	1.20	1
13500363	1.38	1	1.48	1	1.65	2	1.51	2
13500380	1.06	1	1.22	1	1.40	1	1.22	1
13500449	2.22	2	2.56	3	1.92	2	2.23	2
13500602	2.05	2	1.67	2	1.98	2	1.90	2
13500723	4.67	5	4.60	5	6.17	6	5.15	5
13500754	2.85	3	3.11	3	3.35	3	3.10	3
13500880	1.33	1	1.18	1	1.21	1	1.24	1
13501030	4.42	4	4.38	4	4.52	5	4.44	4

Sample ID	Copy Number							
	r = 1		r = 2		r = 3		Average	
	Ratio (target/ref)	Estimated Copy Number	Ratio (target/ref)	Estimated Copy Number	Ratio (target/ref)	Estimated Copy Number	Ratio (target/ref)	Estimated Copy Number
13501065	3.68	4	3.31	3	3.63	4	3.54	4
13510230	2.05	2	1.71	2	1.89	2	1.89	2
13510373	2.11	2	1.82	2	1.99	2	1.97	2
13510439	1.99	2	2.41	2	2.43	2	2.28	2
13510459	1.52	2	1.61	2	1.59	2	1.58	2
13510626	1.27	1	0.99	1	1.01	1	1.09	1
13510627	1.24	1	1.22	1	1.47	1	1.31	1
13510684	2.52	3	2.45	2	2.21	2	2.39	2
13510688	2.04	2	1.28	1	1.60	2	1.64	2
13510869	1.37	1	1.56	2	1.28	1	1.40	1
13520100	2.11	2	1.86	2	1.68	2	1.88	2
13520205	3.47	3	4.02	4	3.83	4	3.77	4
13520217	1.41	1	1.36	1	1.42	1	1.39	1
13520295	2.02	2	1.88	2	1.51	2	1.80	2
13520431	1.60	2	1.43	1	1.32	1	1.45	1
13520653	4.63	5	4.61	5	3.99	4	4.41	4
13530001	6.40	6	6.71	7	7.48	7	6.86	7
13530056	3.20	3	2.94	3	2.61	3	2.92	3
13530183	2.25	2	2.39	2	2.70	3	2.45	2

Sample ID	Copy Number							
	r = 1		r = 2		r = 3		Average	
	Ratio (target/ref)	Estimated Copy Number	Ratio (target/ref)	Estimated Copy Number	Ratio (target/ref)	Estimated Copy Number	Ratio (target/ref)	Estimated Copy Number
13530192	3.06	3	3.58	4	2.85	3	3.16	3
13530282	0.40	0	0.39	0	0.41	0	0.40	0
13530300	0.95	1	1.11	1	1.37	1	1.14	1
13530409	0.86	1	0.77	1	0.84	1	0.82	1
13530419	1.03	1	1.21	1	1.08	1	1.10	1
13530435	1.17	1	1.35	1	1.40	1	1.31	1
13530445	0.53	1	0.56	1	0.60	1	0.56	1
13530579	3.11	3	3.29	3	3.24	3	3.21	3
13530587	1.58	2	1.37	1	1.36	1	1.44	1
13540152	3.31	3	2.79	3	3.44	3	3.18	3
13570233	2.48	2	2.40	2	2.34	2	2.41	2
13580016	4.29	4	4.69	5	4.73	5	4.57	5
13580048	0.16	0	0.19	0	0.18	0	0.18	0
13580121	1.17	1	1.47	1	1.17	1	1.27	1
13580161	3.96	4	3.61	4	3.53	4	3.70	4
13580176	0.70	1	0.74	1	0.82	1	0.75	1
13580205	1.28	1	1.25	1	1.26	1	1.26	1
13580214	1.11	1	1.13	1	1.27	1	1.17	1
13590006	3.28	3	3.93	4	3.53	4	3.58	4

Sample ID	Copy Number							
	r = 1		r = 2		r = 3		Average	
	Ratio (target/ref)	Estimated Copy Number	Ratio (target/ref)	Estimated Copy Number	Ratio (target/ref)	Estimated Copy Number	Ratio (target/ref)	Estimated Copy Number
13590008	1.74	2	1.80	2	2.26	2	1.93	2
13590015	2.45	2	2.20	2	1.96	2	2.20	2
13590036	2.40	2	2.13	2	2.23	2	2.25	2
13590042	1.52	2	1.34	1	1.43	1	1.43	1
13590043	2.51	3	2.11	2	2.38	2	2.33	2
13590072	1.21	1	1.22	1	1.22	1	1.22	1
13590081	1.23	1	1.19	1	1.29	1	1.24	1
13590089	2.53	3	3.02	3	2.48	2	2.68	3
13590091	1.64	2	1.54	2	1.40	1	1.53	2
13590093	1.80	2	1.77	2	1.99	2	1.85	2
13590095	1.80	2	1.71	2	1.37	1	1.63	2
13590098	2.11	2	2.05	2	2.28	2	2.15	2
13590108	1.86	2	1.82	2	1.56	2	1.75	2
13590131	1.94	2	1.87	2	1.90	2	1.90	2
13590141	1.82	2	1.73	2	1.34	1	1.63	2
13590143	2.33	2	2.39	2	2.14	2	2.29	2
13590158	1.21	1	1.18	1	1.30	1	1.23	1
13590169	2.58	3	2.48	2	2.78	3	2.62	3
13590170	1.62	2	1.54	2	1.30	1	1.49	1

Sample ID	Copy Number							
	r = 1		r = 2		r = 3		Average	
	Ratio (target/ref)	Estimated Copy Number	Ratio (target/ref)	Estimated Copy Number	Ratio (target/ref)	Estimated Copy Number	Ratio (target/ref)	Average Copy Number
13590190	1.16	1	1.27	1	1.27	1	1.23	1
13590197	9.51	10	10.77	11	9.92	10	10.07	10
13590198	3.84	4	3.53	4	4.00	4	3.79	4
13590199	1.17	1	1.35	1	1.09	1	1.20	1
13590219	1.02	1	1.11	1	1.20	1	1.11	1
13590224	2.08	2	2.28	2	2.33	2	2.23	2
13590227	2.05	2	2.00	2	2.23	2	2.09	2
13590240	2.29	2	2.55	3	2.35	2	2.40	2
13590254	5.89	6	1.97	2	6.25	6	4.71	5
13590292	2.06	2	2.19	2	2.21	2	2.15	2
13590293	2.54	3	2.34	2	2.38	2	2.42	2
13590296	2.31	2	2.07	2	2.06	2	2.15	2
13590297	1.31	1	1.14	1	1.20	1	1.22	1
13590298	1.34	1	1.33	1	1.43	1	1.37	1
13590299	1.05	1	1.16	1	1.07	1	1.09	1
13590300	1.71	2	1.57	2	1.75	2	1.68	2
13590302	4.02	4	4.11	4	4.30	4	4.14	4
13590303	1.16	1	1.04	1	1.13	1	1.11	1
13590304	1.39	1	1.54	2	1.29	1	1.41	1

Sample ID	Copy Number							
	r = 1		r = 2		r = 3		Average	
	Ratio (target/ref)	Estimated Copy Number	Ratio (target/ref)	Estimated Copy Number	Ratio (target/ref)	Estimated Copy Number	Ratio (target/ref)	Estimated Copy Number
13590306	5.71	6	6.21	6	5.33	5	5.75	6
13590308	1.31	1	1.24	1	1.43	1	1.33	1
13590309	0.97	1	0.92	1	0.82	1	0.90	1
13590310	1.14	1	1.15	1	1.23	1	1.17	1
13590311	2.08	2	2.26	2	2.21	2	2.18	2
13590312	1.24	1	1.31	1	1.29	1	1.28	1
13590313	2.21	2	2.33	2	1.85	2	2.13	2
13590314	0.73	1	0.85	1	0.78	1	0.79	1
13590316	1.90	2	1.47	1	1.54	2	1.63	2
13590317	2.12	2	1.99	2	1.94	2	2.02	2
13590319	0.89	1	0.97	1	0.97	1	0.94	1
13590320	1.65	2	1.65	2	1.69	2	1.66	2
13590321	8.73	9	9.04	9	7.87	8	8.55	9
13590322	1.18	1	1.40	1	1.26	1	1.28	1
13590325	0.52	1	0.51	1	0.55	1	0.52	1
13590326	1.80	2	1.51	2	1.57	2	1.62	2
13590327	1.37	1	1.32	1	1.26	1	1.32	1
13590328	1.24	1	1.44	1	1.45	1	1.37	1
13590334	1.69	2	1.67	2	1.54	2	1.63	2

Sample ID	Copy Number							
	r = 1		r = 2		r = 3		Average	
	Ratio (target/ref)	Estimated Copy Number	Ratio (target/ref)	Estimated Copy Number	Ratio (target/ref)	Estimated Copy Number	Ratio (target/ref)	Estimated Copy Number
13590335	1.27	1	1.37	1	1.37	1	1.34	1
13590337	1.17	1	1.11	1	1.30	1	1.19	1
13590338	1.13	1	1.28	1	1.25	1	1.22	1
13590342	2.81	3	2.27	2	2.91	3	2.67	3
13590343	2.08	2	2.27	2	2.20	2	2.18	2
13590345	1.53	2	1.70	2	1.70	2	1.64	2
13590358	2.13	2	2.07	2	1.95	2	2.05	2
13590390	2.17	2	2.00	2	2.01	2	2.06	2
13590392	1.01	1	1.06	1	1.23	1	1.10	1
13590396	8.44	8	7.67	8	9.30	9	8.47	8
13590429	1.30	1	1.44	1	1.48	1	1.41	1
13590443	3.96	4	3.83	4	3.35	3	3.71	4
13590444	4.14	4	4.90	5	4.74	5	4.59	5
13590445	3.41	3	3.62	4	3.60	4	3.54	4
13590446	6.98	7	6.29	6	5.25	5	6.17	6
13590448	3.53	4	3.27	3	2.99	3	3.27	3
13590449	1.50	2	1.69	2	1.25	1	1.48	1
13590470	6.61	7	6.22	6	6.27	6	6.37	6
13590498	2.09	2	1.15	1	1.21	1	1.48	1

Sample ID	Copy Number							
	r = 1		r = 2		r = 3		Average	
	Ratio (target/ref)	Estimated Copy Number	Ratio (target/ref)	Estimated Copy Number	Ratio (target/ref)	Estimated Copy Number	Ratio (target/ref)	Estimated Copy Number
13590503	0.61	1	0.59	1	0.66	1	0.62	1
13590504	1.99	2	1.68	2	1.82	2	1.83	2
13590506	2.79	3	2.25	2	2.47	2	2.50	3
13590507	1.54	2	1.56	2	1.64	2	1.58	2
13590510	1.07	1	1.01	1	0.85	1	0.98	1
13590536	2.54	3	2.46	2	2.17	2	2.39	2
13590538	2.80	3	2.86	3	3.22	3	2.96	3
13590558	2.29	2	2.03	2	1.97	2	2.10	2
13590559	2.41	2	1.95	2	2.11	2	2.16	2
13590560	2.34	2	2.57	3	2.49	2	2.47	2
13590563	7.91	8	5.65	6	6.07	6	6.54	7
13590591	4.67	5	4.90	5	2.58	3	4.05	4
13590597	1.43	1	1.50	1	1.51	2	1.48	1
13590610	4.33	4	4.27	4	4.02	4	4.21	4
13590615	3.19	3	3.78	4	4.57	5	3.85	4
13590678	1.27	1	1.50	1	1.54	2	1.44	1
13590680	1.47	1	1.61	2	1.63	2	1.57	2
13590683	0.85	1	0.78	1	0.73	1	0.79	1
13590685	1.44	1	1.62	2	1.39	1	1.48	1

Sample ID	Copy Number							
	r = 1		r = 2		r = 3		Average	
	Ratio (target/ref)	Estimated Copy Number	Ratio (target/ref)	Estimated Copy Number	Ratio (target/ref)	Estimated Copy Number	Ratio (target/ref)	Estimated Copy Number
13590686	4.13	4	4.47	4	4.50	4	4.36	4
13590687	1.18	1	1.49	1	1.24	1	1.30	1
13590698	2.28	2	2.11	2	1.92	2	2.10	2
13590702	0.55	1	0.42	0	0.56	1	0.51	1
13590704	1.68	2	1.56	2	1.24	1	1.49	1
13590706	1.17	1	1.12	1	1.11	1	1.13	1
13590708	1.26	1	1.46	1	1.54	2	1.42	1
13590714	1.96	2	1.88	2	1.92	2	1.92	2
13590744	1.59	2	1.46	1	1.42	1	1.49	1
13590748	1.19	1	0.90	1	1.02	1	1.04	1
13590752	3.02	3	3.12	3	2.79	3	2.97	3
13590754	2.23	2	2.15	2	2.19	2	2.19	2
13590760	1.33	1	1.04	1	1.15	1	1.17	1
13590761	3.01	3	3.10	3	3.57	4	3.22	3
13590764	1.29	1	0.99	1	1.15	1	1.14	1
13590765	0.75	1	0.80	1	0.83	1	0.79	1
13590766	2.15	2	2.81	3	2.85	3	2.60	3
13590798	1.30	1	1.16	1	1.02	1	1.16	1
13590801	3.25	3	1.82	2	1.62	2	2.23	2

Sample ID	Copy Number							
	r = 1		r = 2		r = 3		Average	
	Ratio (target/ref)	Estimated Copy Number	Ratio (target/ref)	Estimated Copy Number	Ratio (target/ref)	Estimated Copy Number	Ratio (target/ref)	Estimated Copy Number
13590805	2.98	3	3.71	4	2.62	3	3.10	3
13590810	2.57	3	2.51	3	2.32	2	2.47	2
13590811	1.99	2	1.96	2	1.97	2	1.97	2
13590820	1.18	1	1.10	1	1.24	1	1.17	1
13590822	5.32	5	5.42	5	5.43	5	5.39	5
13590823	2.58	3	2.15	2	2.32	2	2.35	2
13590827	0.49	0	0.45	0	0.48	0	0.47	0
13600003	0.82	1	0.79	1	0.85	1	0.82	1
13600007	1.48	1	1.55	2	1.47	1	1.50	1
13600013	2.23	2	2.15	2	2.38	2	2.25	2
13600014	2.94	3	2.94	3	3.02	3	2.97	3
13600015	2.75	3	2.54	3	2.77	3	2.68	3
13600019	1.27	1	1.16	1	1.26	1	1.23	1
13600021	5.08	5	5.12	5	5.08	5	5.09	5
13600023	2.07	2	1.97	2	2.00	2	2.01	2
13600024	1.37	1	1.35	1	1.23	1	1.32	1
13600032	1.93	2	2.19	2	2.13	2	2.08	2
13600034	1.22	1	1.27	1	1.28	1	1.26	1
13600035	10.65	11	10.32	10	10.73	11	10.57	11

Sample ID	Copy Number							
	r = 1		r = 2		r = 3		Average	
	Ratio (target/ref)	Estimated Copy Number	Ratio (target/ref)	Estimated Copy Number	Ratio (target/ref)	Estimated Copy Number	Ratio (target/ref)	Estimated Copy Number
13600036	1.21	1	1.10	1	1.25	1	1.19	1
13600038	1.21	1	1.17	1	1.15	1	1.18	1
13600039	2.85	3	2.64	3	2.58	3	2.69	3
13600040	2.33	2	1.87	2	1.98	2	2.06	2
13600044	3.60	4	4.66	5	3.83	4	4.03	4
13600045	1.13	1	1.35	1	1.30	1	1.26	1
13600046	1.28	1	1.23	1	1.34	1	1.29	1
13600048	1.82	2	2.36	2	2.00	2	2.06	2
13600049	0.04	0	0.04	0	0.04	0	0.04	0
13600050	1.17	1	1.28	1	1.12	1	1.19	1
13600051	2.03	2	2.20	2	1.92	2	2.05	2
13600052	2.22	2	2.42	2	2.25	2	2.30	2
13600053	0.99	1	1.23	1	1.32	1	1.18	1
13600057	1.46	1	1.23	1	1.25	1	1.31	1
13600058	10.55	11	10.85	11	11.01	11	10.80	11
13600062	0.71	1	0.86	1	0.73	1	0.77	1
13600069	2.36	2	1.75	2	1.64	2	1.92	2
13600076	0.96	1	0.98	1	0.99	1	0.98	1
13600077	2.33	2	2.28	2	2.24	2	2.28	2

Sample ID	Copy Number							
	r = 1		r = 2		r = 3		Average	
	Ratio (target/ref)	Estimated Copy Number	Ratio (target/ref)	Estimated Copy Number	Ratio (target/ref)	Estimated Copy Number	Ratio (target/ref)	Estimated Copy Number
13600078	1.28	1	1.31	1	1.21	1	1.27	1
13600080	4.95	5	5.03	5	5.11	5	5.03	5
13600081	1.07	1	1.00	1	0.95	1	1.00	1
13600084	1.96	2	2.30	2	1.92	2	2.06	2
13600086	2.89	3	3.62	4	3.31	3	3.27	3
13600088	1.48	1	1.18	1	1.47	1	1.38	1
13600089	0.95	1	1.12	1	1.21	1	1.09	1
13600090	1.17	1	1.13	1	1.19	1	1.16	1
13600092	2.23	2	2.31	2	2.85	3	2.46	2
13600094	2.41	2	2.46	2	2.07	2	2.32	2
13600095	2.48	2	2.45	2	2.17	2	2.37	2
13600096	2.32	2	2.47	2	2.67	3	2.49	2
13600099	2.00	2	1.98	2	2.24	2	2.07	2
13600103	1.14	1	1.36	1	1.05	1	1.18	1
13600115	1.40	1	1.55	2	1.35	1	1.44	1
13600125	1.37	1	1.18	1	1.23	1	1.26	1
13600130	1.33	1	1.49	1	1.39	1	1.40	1
13600137	5.89	6	4.98	5	5.84	6	5.57	6
13600156	1.84	2	1.99	2	2.10	2	1.98	2

Sample ID	Copy Number							
	r = 1		r = 2		r = 3		Average	
	Ratio (target/ref)	Estimated Copy Number	Ratio (target/ref)	Estimated Copy Number	Ratio (target/ref)	Estimated Copy Number	Ratio (target/ref)	Estimated Copy Number
13600177	2.22	2	2.04	2	1.94	2	2.07	2
13600184	3.28	3	3.02	3	2.78	3	3.03	3
13600205	0.27	0	0.33	0	0.28	0	0.29	0
13600214	1.46	1	1.06	1	1.30	1	1.27	1
13600230	1.83	2	1.43	1	1.61	2	1.62	2
13600235	1.40	1	1.31	1	1.39	1	1.36	1
13600297	0.89	1	0.89	1	0.84	1	0.87	1
13600304	1.03	1	0.90	1	1.05	1	0.99	1
13600307	1.85	2	1.81	2	1.77	2	1.81	2
13600316	1.85	2	1.83	2	1.64	2	1.78	2
13600325	1.86	2	2.30	2	2.17	2	2.11	2
13600364	1.53	2	1.80	2	2.47	2	1.93	2
13600369	1.04	1	1.01	1	1.04	1	1.03	1
13600372	1.07	1	1.07	1	0.85	1	1.00	1
13600382	1.58	2	1.64	2	1.91	2	1.71	2
13600383	1.12	1	1.26	1	0.96	1	1.11	1
13600389	0.99	1	1.08	1	1.08	1	1.05	1
13600391	0.81	1	1.02	1	1.24	1	1.02	1
13600393	0.77	1	1.14	1	1.09	1	1.00	1

Sample ID	Copy Number							
	r = 1		r = 2		r = 3		Average	
	Ratio (target/ref)	Estimated Copy Number	Ratio (target/ref)	Estimated Copy Number	Ratio (target/ref)	Estimated Copy Number	Ratio (target/ref)	Estimated Copy Number
13600395	1.19	1	1.33	1	1.06	1	1.19	1
13600407	2.01	2	2.37	2	2.24	2	2.21	2
13600408	1.82	2	2.01	2	2.11	2	1.98	2
13610011	0.76	1	1.08	1	1.05	1	0.96	1
13610017	1.89	2	1.78	2	1.78	2	1.82	2
13610030	2.31	2	2.22	2	1.79	2	2.11	2
13610061	1.70	2	1.60	2	1.50	2	1.60	2
13610082	0.65	1	0.63	1	0.68	1	0.65	1
13610086	5.71	6	5.71	6	5.43	5	5.62	6
13610161	0.99	1	0.99	1	0.91	1	0.96	1
13610189	1.04	1	0.92	1	0.80	1	0.92	1
13610191	1.42	1	1.27	1	1.51	2	1.40	1
13610193	1.16	1	0.98	1	1.20	1	1.12	1
13610202	0.95	1	1.07	1	0.89	1	0.97	1
13610207	1.60	2	1.48	1	1.96	2	1.68	2
13610219	1.10	1	0.90	1	0.98	1	1.00	1
13610232	3.53	4	3.73	4	4.18	4	3.81	4
13610235	0.86	1	0.97	1	0.93	1	0.92	1
13610244	0.95	1	1.04	1	1.38	1	1.12	1

Sample ID	Copy Number							
	r = 1		r = 2		r = 3		Average	
	Ratio (target/ref)	Estimated Copy Number	Ratio (target/ref)	Estimated Copy Number	Ratio (target/ref)	Estimated Copy Number	Ratio (target/ref)	Estimated Copy Number
13610256	0.87	1	1.05	1	0.89	1	0.93	1
13610260	0.95	1	1.06	1	1.11	1	1.04	1
13610264	1.09	1	1.38	1	1.18	1	1.22	1
13610270	1.25	1	1.19	1	1.03	1	1.16	1
13610271	3.47	3	3.09	3	3.18	3	3.25	3
13610277	1.51	2	1.67	2	1.47	1	1.55	2
13610282	1.02	1	1.17	1	1.19	1	1.13	1
13610286	2.32	2	2.50	3	2.33	2	2.39	2
13610293	0.35	0	0.34	0	0.47	0	0.39	0
13610298	0.52	1	0.56	1	0.48	0	0.52	1
13610323	1.01	1	1.30	1	1.02	1	1.11	1
13610325	2.66	3	2.11	2	2.01	2	2.26	2
13610326	2.16	2	2.21	2	2.18	2	2.18	2
13610331	1.22	1	1.11	1	1.10	1	1.14	1
13610340	4.88	5	5.25	5	4.49	4	4.87	5
13610345	1.08	1	1.24	1	1.22	1	1.18	1
13610378	0.49	0	0.47	0	0.41	0	0.46	0
13610380	4.46	4	4.18	4	4.25	4	4.30	4
13610392	0.23	0	0.25	0	0.25	0	0.24	0

Sample ID	Copy Number							
	r = 1		r = 2		r = 3		Average	
	Ratio (target/ref)	Estimated Copy Number	Ratio (target/ref)	Estimated Copy Number	Ratio (target/ref)	Estimated Copy Number	Ratio (target/ref)	Estimated Copy Number
13610393	0.13	0	0.12	0	0.11	0	0.12	0
13610403	1.06	1	1.07	1	0.87	1	1.00	1
13610410	0.27	0	0.20	0	0.21	0	0.23	0
13610424	0.10	0	0.08	0	0.07	0	0.08	0
13610433	1.81	2	1.89	2	1.53	2	1.74	2
13610439	1.58	2	1.57	2	1.84	2	1.66	2
13610446	1.88	2	1.78	2	1.98	2	1.88	2
13610461	1.08	1	0.96	1	0.89	1	0.98	1
13610476	1.08	1	0.97	1	0.94	1	1.00	1
13610518	1.75	2	1.87	2	1.85	2	1.82	2
13610529	1.02	1	0.96	1	1.04	1	1.01	1
13610537	0.98	1	1.09	1	1.06	1	1.04	1
13610540	1.12	1	1.05	1	1.24	1	1.14	1
13610563	1.06	1	1.21	1	1.08	1	1.12	1
13610745	1.78	2	1.87	2	2.57	3	2.08	2
13610747	2.72	3	2.82	3	3.33	3	2.96	3
13610759	2.08	2	1.73	2	1.90	2	1.90	2
13610785	1.07	1	1.06	1	1.08	1	1.07	1
13610793	1.06	1	0.97	1	0.99	1	1.01	1

Sample ID	Copy Number							
	r = 1		r = 2		r = 3		Average	
	Ratio (target/ref)	Estimated Copy Number	Ratio (target/ref)	Estimated Copy Number	Ratio (target/ref)	Estimated Copy Number	Ratio (target/ref)	Estimated Copy Number
13610794	3.54	4	3.39	3	3.87	4	3.60	4
13610796	1.73	2	1.65	2	1.66	2	1.68	2
13620023	1.04	1	1.19	1	1.21	1	1.15	1
13620025	1.06	1	0.96	1	1.17	1	1.06	1
13620028	1.07	1	1.00	1	1.13	1	1.07	1
13620040	2.03	2	1.88	2	1.93	2	1.95	2
13620049	0.99	1	0.94	1	0.94	1	0.96	1
13620051	3.42	3	4.09	4	3.44	3	3.65	4
13620059	1.81	2	1.84	2	1.99	2	1.88	2
13620064	0.87	1	0.96	1	0.92	1	0.92	1
13620068	0.03	0	0.02	0	0.02	0	0.02	0
13620069	3.08	3	2.85	3	3.16	3	3.03	3
13620074	0.97	1	1.03	1	1.12	1	1.04	1
13620078	4.64	5	4.04	4	4.53	5	4.40	4
13620081	5.10	5	3.58	4	3.95	4	4.21	4
13620083	1.03	1	1.07	1	1.13	1	1.08	1
13620084	1.85	2	1.99	2	1.98	2	1.94	2
13620085	1.14	1	1.04	1	0.90	1	1.03	1
13620087	4.47	4	4.97	5	4.08	4	4.51	5

Sample ID	Copy Number							
	r = 1		r = 2		r = 3		Average	
	Ratio (target/ref)	Estimated Copy Number	Ratio (target/ref)	Estimated Copy Number	Ratio (target/ref)	Estimated Copy Number	Ratio (target/ref)	Estimated Copy Number
13620088	4.88	5	4.62	5	4.44	4	4.65	5
13620090	5.34	5	4.15	4	4.33	4	4.61	5
13620091	2.68	3	2.17	2	2.40	2	2.42	2
13620092	1.78	2	1.73	2	1.72	2	1.74	2
13620093	1.48	1	1.73	2	1.85	2	1.69	2
13620094	2.12	2	2.17	2	2.18	2	2.16	2
13620096	1.89	2	1.95	2	1.79	2	1.87	2
13620097	1.08	1	1.04	1	1.20	1	1.11	1
13620098	2.94	3	3.36	3	3.07	3	3.12	3
13620099	1.88	2	1.98	2	2.12	2	2.00	2
13620100	2.08	2	1.88	2	1.87	2	1.95	2
13620101	1.93	2	1.93	2	1.95	2	1.94	2
13620107	1.87	2	1.89	2	2.10	2	1.95	2
13620109	1.09	1	1.10	1	1.12	1	1.10	1
13620115	1.23	1	1.09	1	0.99	1	1.10	1
13620116	2.92	3	2.44	2	2.42	2	2.59	3
13620117	2.61	3	2.72	3	2.76	3	2.69	3
13620120	2.44	2	2.38	2	2.08	2	2.30	2
13620121	1.08	1	1.09	1	1.11	1	1.09	1

Sample ID	Copy Number							
	r = 1		r = 2		r = 3		Average	
	Ratio (target/ref)	Estimated Copy Number	Ratio (target/ref)	Estimated Copy Number	Ratio (target/ref)	Estimated Copy Number	Ratio (target/ref)	Estimated Copy Number
13620125	1.82	2	1.46	1	1.88	2	1.72	2
13620127	4.88	5	4.41	4	3.89	4	4.39	4
13620128	2.35	2	2.23	2	1.94	2	2.17	2
13620130	1.45	1	1.08	1	1.17	1	1.23	1
13620132	1.11	1	1.21	1	0.88	1	1.07	1
13620134	0.99	1	1.02	1	0.92	1	0.98	1
13620135	4.02	4	4.18	4	3.85	4	4.02	4
13620136	2.44	2	2.42	2	2.21	2	2.36	2
13620137	1.78	2	2.04	2	2.06	2	1.96	2
13620138	1.37	1	1.34	1	1.27	1	1.33	1
13620139	1.15	1	1.05	1	0.96	1	1.05	1
13620140	2.45	2	2.72	3	2.50	3	2.56	3
13620143	1.16	1	1.21	1	1.15	1	1.17	1
13620144	1.17	1	1.26	1	1.27	1	1.23	1
13620146	2.06	2	2.29	2	2.11	2	2.16	2
13620151	1.27	1	1.53	2	1.25	1	1.35	1
13620152	1.15	1	1.42	1	1.21	1	1.26	1
13620153	2.31	2	2.22	2	2.04	2	2.19	2
13620154	2.84	3	2.19	2	2.30	2	2.44	2

Sample ID	Copy Number							
	r = 1		r = 2		r = 3		Average	
	Ratio (target/ref)	Estimated Copy Number	Ratio (target/ref)	Estimated Copy Number	Ratio (target/ref)	Estimated Copy Number	Ratio (target/ref)	Estimated Copy Number
13620155	0.91	1	1.07	1	1.11	1	1.03	1
13620158	1.03	1	1.04	1	0.88	1	0.98	1
13620159	1.12	1	1.13	1	1.27	1	1.17	1
13620161	0.86	1	0.85	1	1.29	1	1.00	1
13620162	2.25	2	2.30	2	2.23	2	2.26	2
13620163	4.30	4	4.33	4	3.58	4	4.07	4
13620164	4.32	4	4.08	4	4.54	5	4.32	4
13620165	6.53	7	5.96	6	6.86	7	6.45	6
13620166	1.58	2	1.61	2	1.75	2	1.65	2
13620167	1.91	2	1.96	2	2.12	2	2.00	2
13620168	0.84	1	0.92	1	0.85	1	0.87	1
13620169	3.71	4	2.91	3	3.23	3	3.28	3
13620170	3.23	3	3.26	3	2.92	3	3.13	3
13620172	1.52	2	1.66	2	1.76	2	1.65	2
13620175	1.41	1	1.13	1	1.23	1	1.26	1
13620176	1.07	1	1.23	1	1.08	1	1.13	1
13620177	3.84	4	4.11	4	3.77	4	3.91	4
13620179	2.07	2	2.54	3	2.18	2	2.26	2
13620180	1.16	1	1.13	1	1.16	1	1.15	1

Sample ID	Copy Number							
	r = 1		r = 2		r = 3		Average	
	Ratio (target/ref)	Estimated Copy Number	Ratio (target/ref)	Estimated Copy Number	Ratio (target/ref)	Estimated Copy Number	Ratio (target/ref)	Estimated Copy Number
13620181	3.81	4	3.30	3	3.45	3	3.52	4
13620182	0.82	1	0.84	1	0.90	1	0.85	1
13620183	3.93	4	3.45	3	3.96	4	3.78	4
13620185	3.92	4	4.32	4	3.91	4	4.05	4
13620186	2.19	2	2.12	2	2.14	2	2.15	2
13620188	1.13	1	1.13	1	1.08	1	1.11	1
13620190	2.95	3	2.85	3	2.91	3	2.91	3
13620195	1.11	1	1.10	1	1.20	1	1.14	1
13620196	1.90	2	2.28	2	2.00	2	2.06	2
13620200	2.50	3	2.81	3	2.33	2	2.55	3
13620202	0.94	1	1.14	1	1.03	1	1.04	1
13620203	1.04	1	1.29	1	1.04	1	1.12	1
13620209	1.48	1	1.27	1	1.13	1	1.29	1
13620210	2.17	2	1.93	2	1.82	2	1.97	2
13620211	2.11	2	2.07	2	2.35	2	2.18	2
13620218	2.07	2	2.10	2	2.08	2	2.08	2
13620219	2.15	2	1.96	2	2.09	2	2.06	2
13620220	3.77	4	2.76	3	3.44	3	3.32	3
13620223	1.96	2	1.92	2	1.93	2	1.94	2

Sample ID	Copy Number							
	r = 1		r = 2		r = 3		Average	
	Ratio (target/ref)	Estimated Copy Number	Ratio (target/ref)	Estimated Copy Number	Ratio (target/ref)	Estimated Copy Number	Ratio (target/ref)	Estimated Copy Number
13620225	1.07	1	1.13	1	1.09	1	1.10	1
13620226	0.96	1	1.06	1	1.17	1	1.07	1
13620227	1.00	1	1.00	1	1.09	1	1.03	1
13620228	10.17	10	11.86	12	10.90	11	10.98	11
13620229	6.59	7	7.02	7	6.00	6	6.54	7
13620230	5.60	6	7.04	7	6.31	6	6.32	6
13620231	6.12	6	5.91	6	4.63	5	5.56	6
13620232	3.65	4	3.31	3	2.81	3	3.25	3
13620239	1.28	1	1.09	1	1.31	1	1.22	1
13620240	0.37	0	0.34	0	0.33	0	0.35	0
13620241	2.14	2	2.02	2	1.98	2	2.05	2
13620242	1.06	1	1.03	1	1.12	1	1.07	1
13620244	1.48	1	1.73	2	1.68	2	1.63	2
13620245	2.59	3	2.00	2	2.41	2	2.33	2
13620246	1.89	2	1.61	2	1.27	1	1.59	2
13620256	1.10	1	1.01	1	1.10	1	1.07	1
13620257	1.06	1	1.15	1	1.18	1	1.13	1
13620260	2.00	2	1.86	2	2.09	2	1.98	2
13620261	1.81	2	1.94	2	2.15	2	1.97	2

Sample ID	Copy Number							
	r = 1		r = 2		r = 3		Average	
	Ratio (target/ref)	Estimated Copy Number	Ratio (target/ref)	Estimated Copy Number	Ratio (target/ref)	Estimated Copy Number	Ratio (target/ref)	Estimated Copy Number
13620266	0.94	1	0.75	1	0.76	1	0.81	1
13620267	1.37	1	1.26	1	1.16	1	1.27	1
13620268	1.42	1	1.58	2	1.64	2	1.55	2
13620269	1.33	1	1.39	1	1.41	1	1.38	1
13620270	1.24	1	1.19	1	1.16	1	1.20	1
13620272	2.23	2	1.74	2	1.82	2	1.93	2
13620276	2.42	2	2.31	2	2.21	2	2.31	2
13620278	2.05	2	2.19	2	2.74	3	2.33	2
13620282	2.74	3	2.89	3	2.82	3	2.82	3
13620287	1.31	1	1.13	1	1.37	1	1.27	1
13620289	1.28	1	1.11	1	1.11	1	1.17	1
13620290	2.28	2	2.50	3	1.95	2	2.24	2
13620291	2.04	2	1.86	2	2.16	2	2.02	2
13620294	2.13	2	1.95	2	1.95	2	2.01	2
13620295	2.40	2	2.32	2	2.57	3	2.43	2
13620296	1.18	1	1.36	1	1.19	1	1.24	1
13620297	1.35	1	1.27	1	1.29	1	1.30	1
13620298	1.11	1	1.26	1	1.04	1	1.14	1
13620300	1.88	2	1.61	2	1.67	2	1.72	2

Sample ID	Copy Number							
	r = 1		r = 2		r = 3		Average	
	Ratio (target/ref)	Estimated Copy Number	Ratio (target/ref)	Estimated Copy Number	Ratio (target/ref)	Estimated Copy Number	Ratio (target/ref)	Average Copy Number
13620301	1.21	1	1.11	1	0.95	1	1.09	1
13620307	2.15	2	1.89	2	2.16	2	2.07	2
13620308	2.37	2	2.32	2	2.15	2	2.28	2
13620309	1.77	2	1.61	2	1.67	2	1.68	2
13620311	0.98	1	0.89	1	1.27	1	1.05	1
13620314	2.50	3	3.17	3	3.05	3	2.91	3
13620315	5.56	6	6.17	6	4.96	5	5.56	6
13620316	0.87	1	1.00	1	0.83	1	0.90	1
13620319	3.21	3	3.62	4	3.16	3	3.33	3
13620320	2.30	2	2.34	2	2.54	3	2.39	2
13620321	1.15	1	1.18	1	1.05	1	1.13	1
13620323	2.09	2	2.42	2	2.25	2	2.25	2
13620325	6.09	6	6.44	6	6.37	6	6.30	6
13620328	1.21	1	1.20	1	0.96	1	1.12	1
13620329	2.10	2	1.81	2	1.36	1	1.76	2
13620335	1.07	1	1.05	1	1.10	1	1.07	1
13620336	1.67	2	1.69	2	2.18	2	1.84	2
13620339	1.08	1	1.26	1	1.28	1	1.20	1
13620340	2.20	2	2.42	2	2.14	2	2.25	2

Sample ID	Copy Number							
	r = 1		r = 2		r = 3		Average	
	Ratio (target/ref)	Estimated Copy Number	Ratio (target/ref)	Estimated Copy Number	Ratio (target/ref)	Estimated Copy Number	Ratio (target/ref)	Estimated Copy Number
13620341	2.02	2	2.17	2	1.83	2	2.01	2
13620342	2.27	2	2.35	2	2.02	2	2.21	2
13620347	6.86	7	5.92	6	6.26	6	6.35	6
13620349	2.26	2	2.18	2	2.26	2	2.23	2
13620351	2.82	3	3.31	3	2.78	3	2.97	3
13620355	5.69	6	5.40	5	4.82	5	5.30	5
13620356	2.33	2	2.43	2	2.25	2	2.34	2
13620361	1.15	1	1.36	1	1.08	1	1.20	1
13620363	1.39	1	1.20	1	1.45	1	1.35	1
13620365	4.66	5	4.76	5	4.83	5	4.75	5
13620367	1.58	2	0.92	1	0.88	1	1.12	1
13620368	2.31	2	2.23	2	2.20	2	2.24	2
13620370	1.94	2	1.85	2	1.94	2	1.91	2
13620373	0.90	1	1.05	1	1.00	1	0.98	1
13620375	1.30	1	1.18	1	1.38	1	1.29	1
13620376	1.01	1	1.21	1	1.42	1	1.21	1
13620377	2.36	2	2.40	2	2.37	2	2.38	2
13620378	2.30	2	2.13	2	2.07	2	2.16	2
13620379	2.21	2	1.87	2	2.20	2	2.09	2

Sample ID	Copy Number							
	r = 1		r = 2		r = 3		Average	
	Ratio (target/ref)	Estimated Copy Number	Ratio (target/ref)	Estimated Copy Number	Ratio (target/ref)	Estimated Copy Number	Ratio (target/ref)	Estimated Copy Number
13620382	2.27	2	2.15	2	1.96	2	2.13	2
13620383	2.83	3	3.30	3	2.98	3	3.04	3
13620387	1.17	1	1.22	1	1.20	1	1.20	1
13620390	2.39	2	2.11	2	2.17	2	2.22	2
13620391	1.57	2	1.07	1	1.20	1	1.28	1
13620400	2.97	3	3.56	4	3.14	3	3.22	3
13620401	1.14	1	1.14	1	1.16	1	1.14	1
13620402	2.80	3	2.86	3	3.18	3	2.95	3
13620403	3.16	3	2.95	3	3.06	3	3.06	3
13620408	2.13	2	2.00	2	1.93	2	2.02	2
13620410	3.61	4	3.77	4	3.07	3	3.48	3
13620416	2.90	3	3.40	3	3.05	3	3.12	3
13620417	3.57	4	3.68	4	3.58	4	3.61	4
13620418	3.71	4	3.54	4	3.69	4	3.65	4
13620426	1.42	1	1.39	1	1.32	1	1.38	1
13620427	1.93	2	1.91	2	1.68	2	1.84	2
13620433	3.30	3	3.05	3	3.06	3	3.14	3
13620434	2.29	2	2.27	2	2.20	2	2.25	2
13620435	2.25	2	2.12	2	2.37	2	2.25	2

Sample ID	Copy Number							
	r = 1		r = 2		r = 3		Average	
	Ratio (target/ref)	Estimated Copy Number	Ratio (target/ref)	Estimated Copy Number	Ratio (target/ref)	Estimated Copy Number	Ratio (target/ref)	Estimated Copy Number
13620436	2.25	2	2.16	2	2.26	2	2.22	2
13620437	2.39	2	2.38	2	2.57	3	2.44	2
13620438	1.12	1	1.08	1	1.10	1	1.10	1
13620440	2.34	2	2.38	2	2.37	2	2.36	2
13620442	2.30	2	2.28	2	2.38	2	2.32	2
13620444	1.43	1	1.60	2	1.49	1	1.51	2
13620445	0.95	1	1.06	1	0.98	1	0.99	1
13620447	3.69	4	3.77	4	4.10	4	3.85	4
13620448	3.93	4	4.05	4	4.23	4	4.07	4
13620449	3.60	4	3.47	3	3.53	4	3.53	4
13620451	1.12	1	1.29	1	1.29	1	1.23	1
13620452	2.56	3	2.96	3	2.71	3	2.74	3
13620455	2.29	2	2.44	2	2.79	3	2.51	3
13620456	1.85	2	2.07	2	2.22	2	2.04	2
13620459	1.26	1	1.23	1	1.21	1	1.24	1
13620460	1.25	1	1.25	1	1.25	1	1.25	1
13620461	1.32	1	1.35	1	1.32	1	1.33	1
13620462	1.31	1	1.33	1	1.30	1	1.32	1
13620467	1.32	1	1.28	1	1.30	1	1.30	1

Sample ID	Copy Number							
	r = 1		r = 2		r = 3		Average	
	Ratio (target/ref)	Estimated Copy Number	Ratio (target/ref)	Estimated Copy Number	Ratio (target/ref)	Estimated Copy Number	Ratio (target/ref)	Estimated Copy Number
13620468	1.13	1	0.98	1	1.05	1	1.05	1
13620470	1.60	2	1.74	2	1.84	2	1.73	2
13620471	1.73	2	1.68	2	1.42	1	1.61	2
13620472	2.69	3	3.67	4	3.25	3	3.20	3
13620473	2.35	2	1.85	2	2.28	2	2.16	2
13620475	0.91	1	1.20	1	1.15	1	1.08	1
13620476	2.23	2	1.95	2	2.68	3	2.29	2
13620477	1.71	2	1.01	1	1.39	1	1.37	1
13620479	1.74	2	2.51	3	2.28	2	2.18	2
13620482	3.01	3	3.72	4	3.49	3	3.41	3
13620485	3.07	3	3.58	4	3.41	3	3.35	3
13620487	7.83	8	7.96	8	8.65	9	8.15	8
13620492	1.50	2	1.50	1	1.48	1	1.49	1
13620493	2.68	3	2.97	3	3.07	3	2.90	3
13620495	2.08	2	1.52	2	1.49	1	1.70	2
13620497	2.52	3	1.99	2	2.26	2	2.26	2
13620503	3.09	3	3.58	4	3.36	3	3.35	3
13620504	2.84	3	2.34	2	2.30	2	2.49	2
13620505	1.34	1	1.15	1	1.47	1	1.32	1

Sample ID	Copy Number							
	r = 1		r = 2		r = 3		Average	
	Ratio (target/ref)	Estimated Copy Number	Ratio (target/ref)	Estimated Copy Number	Ratio (target/ref)	Estimated Copy Number	Ratio (target/ref)	Estimated Copy Number
13620507	1.62	2	2.12	2	2.17	2	1.97	2
13620511	4.49	4	4.30	4	4.58	5	4.46	4
13620512	2.79	3	2.88	3	2.95	3	2.87	3
13620513	1.81	2	1.68	2	1.55	2	1.68	2
13620514	1.06	1	0.94	1	0.96	1	0.99	1
13620516	1.09	1	1.07	1	0.86	1	1.01	1
13620521	1.59	2	1.32	1	1.56	2	1.49	1
13620523	1.77	2	1.66	2	1.89	2	1.77	2
13620525	3.70	4	3.95	4	3.58	4	3.74	4
13620526	1.16	1	1.44	1	1.38	1	1.33	1
13620527	0.51	1	0.61	1	0.53	1	0.55	1
13620528	0.83	1	0.70	1	0.56	1	0.70	1
13620530	1.06	1	1.25	1	1.19	1	1.17	1
13620531	0.68	1	0.75	1	0.80	1	0.75	1
13620532	2.08	2	1.81	2	2.17	2	2.02	2
13620533	1.68	2	1.78	2	2.33	2	1.93	2
13620534	1.39	1	1.14	1	1.35	1	1.29	1
13620537	1.72	2	2.03	2	2.16	2	1.97	2
13620539	3.79	4	3.47	3	4.55	5	3.94	4

Sample ID	Copy Number							
	r = 1		r = 2		r = 3		Average	
	Ratio (target/ref)	Estimated Copy Number	Ratio (target/ref)	Estimated Copy Number	Ratio (target/ref)	Estimated Copy Number	Ratio (target/ref)	Estimated Copy Number
13620545	0.89	1	0.79	1	0.95	1	0.88	1
13620553	0.92	1	0.89	1	0.92	1	0.91	1
13620557	1.37	1	1.17	1	1.43	1	1.33	1
13620558	1.07	1	1.19	1	1.15	1	1.14	1
13620562	1.25	1	1.12	1	1.25	1	1.21	1
13620563	1.05	1	1.30	1	1.06	1	1.14	1
13620566	4.42	4	4.82	5	3.81	4	4.35	4
13620569	2.69	3	3.05	3	2.97	3	2.90	3
13620572	2.93	3	2.49	2	2.37	2	2.60	3
13620574	1.93	2	2.12	2	1.95	2	2.00	2
13620575	1.80	2	2.06	2	2.22	2	2.03	2
13620578	4.04	4	4.11	4	4.85	5	4.33	4
13620586	4.17	4	3.95	4	3.87	4	4.00	4
13620588	1.34	1	1.22	1	1.04	1	1.20	1
13620589	7.56	8	5.76	6	6.93	7	6.75	7
13620590	4.87	5	4.77	5	5.55	6	5.06	5
13620592	1.38	1	1.35	1	1.75	2	1.49	1
13620593	1.66	2	1.60	2	1.68	2	1.64	2
13620595	1.11	1	1.07	1	1.02	1	1.07	1

Sample ID	Copy Number							
	r = 1		r = 2		r = 3		Average	
	Ratio (target/ref)	Estimated Copy Number	Ratio (target/ref)	Estimated Copy Number	Ratio (target/ref)	Estimated Copy Number	Ratio (target/ref)	Estimated Copy Number
13620596	1.23	1	1.33	1	1.08	1	1.21	1
13620598	1.20	1	1.41	1	1.26	1	1.29	1
13620599	7.16	7	7.18	7	9.47	9	7.94	8
13620600	0.05	0	0.04	0	0.05	0	0.05	0
13620601	1.02	1	0.94	1	1.08	1	1.01	1
13620602	0.51	1	0.49	0	0.52	1	0.51	1
13620604	1.21	1	1.06	1	1.11	1	1.13	1
13620606	1.29	1	1.41	1	1.14	1	1.28	1
13620607	1.14	1	1.13	1	1.20	1	1.16	1
13620608	1.69	2	1.51	2	1.52	2	1.57	2
13620609	1.15	1	1.32	1	1.27	1	1.24	1
13620610	2.38	2	2.71	3	2.55	3	2.55	3
13620611	6.75	7	6.79	7	5.34	5	6.29	6
13620612	3.51	4	2.89	3	3.23	3	3.21	3
13620613	1.71	2	1.63	2	1.58	2	1.64	2
13620616	0.66	1	0.62	1	0.67	1	0.65	1
13620617	5.91	6	5.74	6	7.16	7	6.27	6
13620618	4.73	5	5.34	5	5.07	5	5.04	5
13620619	3.51	4	3.88	4	3.58	4	3.66	4

Sample ID	Copy Number							
	r = 1		r = 2		r = 3		Average	
	Ratio (target/ref)	Estimated Copy Number	Ratio (target/ref)	Estimated Copy Number	Ratio (target/ref)	Estimated Copy Number	Ratio (target/ref)	Estimated Copy Number
13620625	4.21	4	3.20	3	3.32	3	3.58	4
13620627	7.45	7	6.96	7	7.29	7	7.23	7
13620630	3.54	4	3.67	4	3.62	4	3.61	4
13620633	1.13	1	1.29	1	1.29	1	1.24	1
13620636	1.11	1	0.93	1	1.27	1	1.10	1
13620637	1.12	1	1.08	1	1.23	1	1.14	1
13620638	2.27	2	1.96	2	2.15	2	2.13	2
13620640	2.41	2	2.40	2	2.48	2	2.43	2
13620641	2.11	2	2.40	2	2.29	2	2.27	2
13620642	3.14	3	3.09	3	3.27	3	3.17	3
13620644	1.03	1	1.16	1	1.10	1	1.10	1
13620645	1.34	1	1.35	1	1.15	1	1.28	1
13620647	1.56	2	1.75	2	1.54	2	1.62	2
13620648	2.10	2	1.60	2	2.01	2	1.90	2
13620649	3.24	3	3.06	3	1.94	2	2.75	3
13620650	1.71	2	1.63	2	1.85	2	1.73	2
13620652	2.24	2	2.15	2	2.42	2	2.27	2
13620660	1.16	1	1.25	1	1.11	1	1.17	1
13620661	0.65	1	0.75	1	0.67	1	0.69	1

Sample ID	Copy Number							
	r = 1		r = 2		r = 3		Average	
	Ratio (target/ref)	Estimated Copy Number	Ratio (target/ref)	Estimated Copy Number	Ratio (target/ref)	Estimated Copy Number	Ratio (target/ref)	Estimated Copy Number
13620663	0.89	1	0.99	1	0.88	1	0.92	1
13620674	1.24	1	1.34	1	1.07	1	1.22	1
13620675	1.06	1	1.09	1	1.05	1	1.07	1
13620678	1.40	1	1.32	1	1.12	1	1.28	1
13620679	1.12	1	1.27	1	1.65	2	1.35	1
13620681	1.81	2	1.88	2	1.86	2	1.85	2
13620682	4.38	4	4.15	4	3.97	4	4.17	4
13620684	1.50	1	1.32	1	1.45	1	1.42	1
13620689	2.49	2	2.68	3	2.47	2	2.54	3
13620690	6.15	6	5.91	6	5.79	6	5.95	6
13620691	1.97	2	2.07	2	1.99	2	2.01	2
13620692	4.59	5	4.04	4	4.74	5	4.46	4
13620694	3.54	4	3.62	4	3.00	3	3.39	3
13620695	2.89	3	2.95	3	3.24	3	3.03	3
13620876	1.00	1	1.06	1	0.91	1	0.99	1
13620877	0.90	1	1.21	1	1.22	1	1.11	1
13620878	1.73	2	1.51	2	1.99	2	1.74	2
13620879	1.88	2	2.10	2	2.33	2	2.10	2
13620880	2.85	3	3.14	3	2.45	2	2.81	3

Sample ID	Copy Number							
	r = 1		r = 2		r = 3		Average	
	Ratio (target/ref)	Estimated Copy Number	Ratio (target/ref)	Estimated Copy Number	Ratio (target/ref)	Estimated Copy Number	Ratio (target/ref)	Estimated Copy Number
13620881	1.05	1	1.05	1	1.26	1	1.12	1
13620884	1.99	2	1.78	2	1.94	2	1.90	2
13620885	1.81	2	1.95	2	2.09	2	1.95	2
13620887	1.05	1	0.92	1	0.98	1	0.99	1
13620888	1.29	1	1.33	1	1.23	1	1.29	1
13620890	1.13	1	1.16	1	1.25	1	1.18	1
13620893	0.93	1	1.21	1	1.11	1	1.09	1
13620894	1.68	2	1.38	1	1.36	1	1.47	1
13620898	7.97	8	6.85	7	7.08	7	7.30	7
13620899	0.89	1	1.07	1	0.89	1	0.95	1
13620900	1.90	2	2.13	2	2.08	2	2.03	2
13620907	3.53	4	3.76	4	3.50	3	3.60	4
13620914	4.04	4	3.34	3	4.07	4	3.82	4
13620917	2.99	3	3.12	3	3.21	3	3.11	3
13620918	2.72	3	2.95	3	2.90	3	2.86	3
13620921	3.56	4	3.50	3	3.65	4	3.57	4
13620922	1.00	1	1.27	1	1.10	1	1.12	1
13620923	1.85	2	1.89	2	1.78	2	1.84	2
13620924	3.86	4	3.61	4	3.49	3	3.66	4

Sample ID	Copy Number							
	r = 1		r = 2		r = 3		Average	
	Ratio (target/ref)	Estimated Copy Number	Ratio (target/ref)	Estimated Copy Number	Ratio (target/ref)	Estimated Copy Number	Ratio (target/ref)	Estimated Copy Number
13620925	2.44	2	2.39	2	2.44	2	2.42	2
13620928	1.86	2	2.43	2	1.89	2	2.06	2
13620929	1.21	1	1.55	2	1.63	2	1.46	1
13620931	5.08	5	5.12	5	5.60	6	5.27	5
13620932	5.81	6	5.18	5	5.26	5	5.42	5
13620933	1.40	1	1.42	1	1.32	1	1.38	1
13620934	1.11	1	1.18	1	1.08	1	1.12	1
13620937	1.03	1	1.13	1	1.16	1	1.11	1
13620939	0.97	1	1.17	1	1.08	1	1.07	1
13620940	1.26	1	1.24	1	1.11	1	1.21	1
13620944	1.12	1	1.29	1	0.94	1	1.12	1
13620948	3.42	3	3.37	3	3.19	3	3.32	3
13620952	1.98	2	1.80	2	1.82	2	1.87	2
13620953	2.84	3	2.80	3	2.87	3	2.84	3
13620954	3.54	4	3.73	4	3.78	4	3.69	4
13620955	1.31	1	1.15	1	1.37	1	1.28	1
13620957	10.05	10	9.60	10	8.52	9	9.39	9
13620959	1.66	2	2.10	2	2.04	2	1.93	2
13620961	9.53	10	9.50	10	10.22	10	9.75	10

Sample ID	Copy Number							
	r = 1		r = 2		r = 3		Average	
	Ratio (target/ref)	Estimated Copy Number	Ratio (target/ref)	Estimated Copy Number	Ratio (target/ref)	Estimated Copy Number	Ratio (target/ref)	Estimated Copy Number
13620962	0.97	1	0.97	1	1.24	1	1.06	1
13620964	2.89	3	3.53	4	2.97	3	3.13	3
13620975	1.48	1	1.09	1	1.08	1	1.22	1
13620977	1.14	1	1.06	1	1.13	1	1.11	1
13620979	3.00	3	3.01	3	3.28	3	3.10	3
13620980	4.22	4	3.98	4	4.15	4	4.12	4
13620987	5.23	5	5.13	5	4.68	5	5.01	5
13620988	4.02	4	3.48	3	3.12	3	3.54	4
13620991	1.17	1	1.24	1	1.16	1	1.19	1
13621004	1.29	1	1.21	1	1.31	1	1.27	1
13621006	1.26	1	1.20	1	1.20	1	1.22	1
13621007	4.84	5	5.86	6	4.80	5	5.16	5
13621008	3.59	4	3.11	3	3.75	4	3.48	3
13621013	1.16	1	1.18	1	1.11	1	1.15	1
13621015	2.51	3	2.17	2	2.17	2	2.29	2
13621016	2.55	3	2.33	2	2.97	3	2.62	3
13621018	1.33	1	0.99	1	0.75	1	1.02	1
13630001	2.03	2	1.96	2	2.10	2	2.03	2
13630003	1.18	1	1.13	1	1.03	1	1.11	1

Sample ID	Copy Number							
	r = 1		r = 2		r = 3		Average	
	Ratio (target/ref)	Estimated Copy Number	Ratio (target/ref)	Estimated Copy Number	Ratio (target/ref)	Estimated Copy Number	Ratio (target/ref)	Estimated Copy Number
13630004	0.99	1	0.88	1	0.89	1	0.92	1
13630005	1.16	1	1.15	1	1.18	1	1.16	1
13630006	1.54	2	1.59	2	1.48	1	1.53	2
13630009	1.09	1	1.06	1	1.03	1	1.06	1
13630011	2.21	2	2.35	2	2.79	3	2.45	2
13630012	3.94	4	4.69	5	4.38	4	4.34	4
13630014	0.84	1	0.77	1	0.70	1	0.77	1
13630015	1.19	1	1.08	1	1.11	1	1.13	1
13630017	0.91	1	1.08	1	0.89	1	0.96	1
13630019	2.82	3	3.30	3	3.30	3	3.14	3
13630021	1.95	2	2.05	2	1.87	2	1.96	2
13630022	2.26	2	1.82	2	1.84	2	1.98	2
13630025	2.56	3	2.34	2	2.96	3	2.62	3
13630026	1.28	1	1.11	1	1.19	1	1.19	1
13630027	1.04	1	1.09	1	1.04	1	1.06	1
13630028	1.00	1	1.16	1	1.02	1	1.06	1
13630029	4.89	5	4.95	5	5.31	5	5.05	5
13630030	1.30	1	1.52	2	1.49	1	1.44	1
13630032	3.01	3	3.03	3	3.10	3	3.05	3

Sample ID	Copy Number							
	r = 1		r = 2		r = 3		Average	
	Ratio (target/ref)	Estimated Copy Number	Ratio (target/ref)	Estimated Copy Number	Ratio (target/ref)	Estimated Copy Number	Ratio (target/ref)	Estimated Copy Number
13630033	1.22	1	1.11	1	1.20	1	1.18	1
13630035	2.02	2	2.17	2	1.68	2	1.96	2
13630036	3.95	4	4.40	4	3.85	4	4.07	4
13630037	0.95	1	1.21	1	1.05	1	1.07	1
13630040	3.86	4	3.75	4	4.31	4	3.97	4
13630041	1.84	2	2.13	2	1.99	2	1.99	2
13630889	2.26	2	3.23	3	1.54	2	2.34	2
13640170	4.08	4	1.74	2	2.57	3	2.80	3
13640193	2.09	2	1.76	2	2.01	2	1.95	2
13640543	1.53	2	1.61	2	2.04	2	1.73	2
13640936	1.70	2	1.54	2	2.25	2	1.83	2
13650161	2.10	2	1.99	2	1.84	2	1.98	2
13650425	1.35	1	1.31	1	1.52	2	1.40	1
13650474	1.11	1	1.15	1	1.04	1	1.10	1
13660110	1.10	1	1.03	1	1.14	1	1.09	1
13660119	0.42	0	0.42	0	0.45	0	0.43	0
13660122	5.55	6	5.61	6	5.72	6	5.62	6
13660123	1.24	1	1.12	1	1.06	1	1.14	1
13660124	3.35	3	3.71	4	3.08	3	3.38	3

Sample ID	Copy Number							
	r = 1		r = 2		r = 3		Average	
	Ratio (target/ref)	Estimated Copy Number	Ratio (target/ref)	Estimated Copy Number	Ratio (target/ref)	Estimated Copy Number	Ratio (target/ref)	Estimated Copy Number
13660127	3.92	4	3.84	4	4.01	4	3.92	4
13660134	1.15	1	1.13	1	1.09	1	1.12	1
13660141	4.52	5	5.31	5	4.60	5	4.81	5
13660166	1.15	1	1.15	1	1.25	1	1.19	1
13660177	1.11	1	1.18	1	1.10	1	1.13	1
13660180	0.24	0	0.33	0	0.26	0	0.28	0
13660183	1.04	1	1.07	1	0.97	1	1.02	1
13660192	1.10	1	1.02	1	1.12	1	1.08	1
13660195	1.74	2	1.78	2	1.86	2	1.80	2
13660333	1.07	1	0.94	1	1.01	1	1.00	1
13660485	3.00	3	3.18	3	3.19	3	3.12	3
13660680	2.21	2	1.98	2	2.06	2	2.08	2
13660683	1.97	2	2.12	2	2.25	2	2.11	2
13660686	1.05	1	1.07	1	1.27	1	1.13	1
13660708	1.11	1	1.14	1	0.90	1	1.05	1
13660711	2.18	2	1.94	2	1.86	2	1.99	2
13660726	1.08	1	0.90	1	0.99	1	0.99	1
13660728	0.92	1	0.97	1	0.93	1	0.94	1
13660730	1.06	1	0.96	1	1.07	1	1.03	1

Sample ID	Copy Number							
	r = 1		r = 2		r = 3		Average	
	Ratio (target/ref)	Estimated Copy Number	Ratio (target/ref)	Estimated Copy Number	Ratio (target/ref)	Estimated Copy Number	Ratio (target/ref)	Estimated Copy Number
13660734	1.67	2	2.14	2	1.30	1	1.70	2
13670001	1.61	2	1.37	1	1.25	1	1.41	1
13670086	2.09	2	1.82	2	1.84	2	1.92	2
13670106	0.58	1	0.67	1	0.69	1	0.65	1
13670146	1.13	1	1.21	1	1.04	1	1.13	1
13670218	1.18	1	0.99	1	1.19	1	1.12	1
13670314	2.11	2	1.99	2	1.79	2	1.96	2
13670406	0.97	1	1.17	1	0.75	1	0.96	1
13670409	1.20	1	1.17	1	1.00	1	1.12	1
13670434	2.06	2	2.03	2	1.91	2	2.00	2
13670437	1.24	1	1.23	1	1.19	1	1.22	1
13670460	1.39	1	1.40	1	1.38	1	1.39	1
13670465	1.03	1	1.15	1	1.05	1	1.08	1
13670492	2.22	2	2.17	2	2.41	2	2.27	2
13680001	1.59	2	1.89	2	1.77	2	1.75	2
13680022	3.04	3	2.70	3	2.62	3	2.79	3
13680087	1.53	2	0.99	1	1.10	1	1.21	1
13680112	1.30	1	1.14	1	1.27	1	1.24	1
13680166	2.45	2	1.95	2	1.95	2	2.12	2

Sample ID	Copy Number							
	r = 1		r = 2		r = 3		Average	
	Ratio (target/ref)	Estimated Copy Number	Ratio (target/ref)	Estimated Copy Number	Ratio (target/ref)	Estimated Copy Number	Ratio (target/ref)	Estimated Copy Number
13680183	1.11	1	1.02	1	1.09	1	1.07	1
13680200	1.14	1	1.15	1	1.20	1	1.16	1
13680235	1.94	2	1.63	2	1.45	1	1.67	2
13680306	1.38	1	1.32	1	1.11	1	1.27	1
13680317	1.36	1	1.25	1	1.34	1	1.32	1
13680580	1.41	1	1.21	1	1.24	1	1.29	1
13680654	1.15	1	1.12	1	1.11	1	1.13	1
13690100	1.15	1	1.36	1	1.28	1	1.26	1
13690182	2.06	2	2.20	2	2.04	2	2.10	2
13690187	0.27	0	1.18	1	1.22	1	0.89	1
13690192	2.11	2	1.93	2	1.82	2	1.95	2
13690256	1.77	2	1.65	2	2.04	2	1.82	2
13690291	2.47	2	2.36	2	2.31	2	2.38	2
13700061	1.84	2	1.88	2	2.15	2	1.95	2
13700151	0.51	1	0.57	1	0.51	1	0.53	1
13700159	2.37	2	2.13	2	2.53	3	2.34	2
13700185	2.51	3	2.40	2	2.15	2	2.35	2
13700202	3.46	3	3.41	3	3.11	3	3.33	3
13710018	1.45	1	1.25	1	1.49	1	1.40	1

Sample ID	Copy Number							
	r = 1		r = 2		r = 3		Average	
	Ratio (target/ref)	Estimated Copy Number	Ratio (target/ref)	Estimated Copy Number	Ratio (target/ref)	Estimated Copy Number	Ratio (target/ref)	Estimated Copy Number
13710080	2.05	2	2.42	2	1.88	2	2.12	2
13710087	2.47	2	2.63	3	2.43	2	2.51	3
13710090	1.45	1	1.41	1	1.59	2	1.48	1
13710102	1.83	2	2.06	2	2.31	2	2.06	2
13710120	2.34	2	2.06	2	2.61	3	2.34	2
13710128	1.20	1	1.35	1	1.27	1	1.27	1
13710157	1.15	1	1.17	1	1.22	1	1.18	1
13710188	1.44	1	1.19	1	1.11	1	1.25	1
13710191	1.42	1	1.55	2	1.46	1	1.47	1
13710217	2.18	2	2.17	2	1.65	2	2.00	2
13710224	1.37	1	1.19	1	1.19	1	1.25	1
13710229	1.37	1	1.23	1	1.31	1	1.30	1
13710230	3.75	4	2.94	3	3.65	4	3.45	3
13710248	1.28	1	1.45	1	1.21	1	1.31	1
13710260	2.85	3	2.26	2	1.03	1	2.05	2
13750003	1.93	2	1.65	2	1.83	2	1.81	2
13760001	3.05	3	2.79	3	2.77	3	2.87	3
13760009	1.28	1	1.25	1	1.09	1	1.20	1
13770002	2.22	2	2.63	3	2.37	2	2.41	2

Sample ID	Copy Number							
	r = 1		r = 2		r = 3		Average	
	Ratio (target/ref)	Estimated Copy Number	Ratio (target/ref)	Estimated Copy Number	Ratio (target/ref)	Estimated Copy Number	Ratio (target/ref)	Estimated Copy Number
13770008	2.91	3	2.88	3	2.89	3	2.89	3
13770009	2.45	2	2.69	3	2.22	2	2.45	2
13780005	2.84	3	2.43	2	2.39	2	2.55	3
13780006	4.26	4	6.37	6	5.83	6	5.49	5
13800003	3.99	4	4.15	4	3.95	4	4.03	4
13800010	5.86	6	5.21	5	5.26	5	5.44	5
13800011	2.33	2	2.24	2	1.95	2	2.17	2
13800012	1.48	1	1.42	1	1.59	2	1.49	1
13800051	1.00	1	1.16	1	1.13	1	1.10	1
13810006	1.05	1	1.18	1	0.81	1	1.01	1
13810007	0.75	1	0.70	1	0.81	1	0.75	1
13810009	1.66	2	1.98	2	1.70	2	1.78	2
13820002	1.46	1	1.49	1	1.64	2	1.53	2
13820004	1.54	2	1.04	1	1.52	2	1.36	1
13820005	2.45	2	2.33	2	2.46	2	2.41	2
13820008	1.37	1	1.23	1	1.26	1	1.29	1
14010013	1.43	1	1.12	1	1.13	1	1.23	1
14010017	1.48	1	1.42	1	1.33	1	1.41	1
14010018	2.10	2	1.90	2	1.66	2	1.89	2

Sample ID	Copy Number							
	r = 1		r = 2		r = 3		Average	
	Ratio (target/ref)	Estimated Copy Number	Ratio (target/ref)	Estimated Copy Number	Ratio (target/ref)	Estimated Copy Number	Ratio (target/ref)	Estimated Copy Number
14010024	9.16	9	7.21	7	6.94	7	7.77	8
14010025	5.98	6	4.91	5	5.77	6	5.56	6
14010026	1.74	2	2.06	2	1.96	2	1.92	2
14010030	2.32	2	2.46	2	2.36	2	2.38	2
14010031	2.47	2	1.73	2	1.86	2	2.02	2
14010032	0.97	1	1.05	1	1.01	1	1.01	1
14010033	2.38	2	2.87	3	2.76	3	2.67	3
14010034	4.03	4	3.82	4	3.85	4	3.90	4
14010036	5.28	5	6.35	6	5.42	5	5.68	6
14010043	3.43	3	3.13	3	3.17	3	3.24	3
14010044	1.48	1	1.55	2	1.72	2	1.58	2
14010045	2.11	2	1.87	2	2.00	2	1.99	2
14010046	1.36	1	4.35	4	4.47	4	3.39	3
14010048	2.83	3	2.82	3	1.79	2	2.48	2
14010053	5.52	6	5.88	6	6.99	7	6.13	6
14010057	3.77	4	4.25	4	4.47	4	4.16	4
14010059	1.98	2	1.89	2	1.85	2	1.91	2
14010065	11.06	11	9.60	10	8.14	8	9.60	10
14010070	6.08	6	5.71	6	5.49	5	5.76	6

Sample ID	Copy Number							
	r = 1		r = 2		r = 3		Average	
	Ratio (target/ref)	Estimated Copy Number	Ratio (target/ref)	Estimated Copy Number	Ratio (target/ref)	Estimated Copy Number	Ratio (target/ref)	Estimated Copy Number
14010075	1.37	1	1.24	1	1.37	1	1.32	1
14010081	1.55	2	1.53	2	1.44	1	1.51	2
14010084	1.40	1	1.68	2	1.33	1	1.47	1
14010086	2.79	3	2.94	3	2.37	2	2.70	3
14010087	8.26	8	8.00	8	7.37	7	7.87	8
14010089	4.31	4	4.56	5	4.69	5	4.52	5
14010091	1.32	1	1.21	1	1.26	1	1.26	1
14010092	6.21	6	4.59	5	3.70	4	4.84	5
14010094	18.17	18	16.10	16	16.50	17	16.92	17
14010099	3.72	4	3.56	4	3.09	3	3.46	3
14010107	9.35	9	7.57	8	8.45	8	8.46	8
14010108	8.76	9	9.05	9	8.20	8	8.67	9
14010114	1.31	1	1.10	1	1.19	1	1.20	1
14010117	1.91	2	1.83	2	1.96	2	1.90	2
14010123	1.08	1	1.17	1	1.18	1	1.15	1
14010126	12.03	12	12.21	12	10.42	10	11.55	12
14010127	2.71	3	2.92	3	2.82	3	2.82	3
14010128	7.34	7	7.07	7	7.10	7	7.17	7
14010130	1.17	1	1.39	1	1.32	1	1.29	1

Sample ID	Copy Number							
	r = 1		r = 2		r = 3		Average	
	Ratio (target/ref)	Estimated Copy Number	Ratio (target/ref)	Estimated Copy Number	Ratio (target/ref)	Estimated Copy Number	Ratio (target/ref)	Estimated Copy Number
14010135	1.86	2	1.95	2	1.99	2	1.93	2
14010136	3.71	4	2.89	3	3.41	3	3.34	3
14010139	1.31	1	1.27	1	1.33	1	1.30	1
14010140	3.64	4	3.97	4	4.18	4	3.93	4
14010141	4.16	4	4.25	4	4.40	4	4.27	4
14010142	9.25	9	9.22	9	9.45	9	9.31	9
14010145	1.37	1	1.21	1	1.04	1	1.21	1
14010147	1.26	1	1.17	1	1.13	1	1.18	1
14010151	2.20	2	2.00	2	2.12	2	2.11	2
14010152	1.27	1	1.18	1	1.13	1	1.19	1
14010155	0.92	1	0.91	1	0.79	1	0.88	1
14010156	3.71	4	3.53	4	3.44	3	3.56	4
14010160	1.12	1	1.22	1	1.29	1	1.21	1
14010163	0.99	1	1.08	1	1.11	1	1.06	1
14010165	1.09	1	1.10	1	0.92	1	1.04	1
14010166	5.70	6	5.34	5	5.23	5	5.42	5
14010167	4.48	4	4.10	4	4.04	4	4.21	4
14010168	2.40	2	2.13	2	2.21	2	2.25	2
14010169	1.03	1	1.06	1	1.07	1	1.05	1

Sample ID	Copy Number							
	r = 1		r = 2		r = 3		Average	
	Ratio (target/ref)	Estimated Copy Number	Ratio (target/ref)	Estimated Copy Number	Ratio (target/ref)	Estimated Copy Number	Ratio (target/ref)	Estimated Copy Number
14010170	2.07	2	2.18	2	2.06	2	2.10	2
14010177	1.22	1	1.18	1	1.14	1	1.18	1
14010181	3.22	3	3.03	3	2.89	3	3.05	3
14010182	4.90	5	4.26	4	4.31	4	4.49	4
14010183	4.62	5	4.55	5	4.35	4	4.51	5
14010187	2.76	3	2.59	3	2.65	3	2.67	3
14010191	1.35	1	1.37	1	1.33	1	1.35	1
14010192	1.93	2	2.13	2	2.08	2	2.05	2
14010193	3.79	4	3.68	4	3.93	4	3.80	4
14010194	1.15	1	1.31	1	1.23	1	1.23	1
14010195	4.70	5	5.38	5	5.56	6	5.21	5
14010197	3.31	3	3.51	4	3.59	4	3.47	3
14010199	1.56	2	1.44	1	1.43	1	1.47	1
14010201	6.45	6	6.75	7	6.06	6	6.42	6
14010205	4.72	5	4.79	5	4.13	4	4.55	5
14010207	1.21	1	1.19	1	1.10	1	1.17	1
14010209	1.38	1	1.47	1	1.38	1	1.41	1
14010212	1.47	1	1.43	1	1.43	1	1.44	1
14010217	2.75	3	2.49	2	2.43	2	2.56	3

Sample ID	Copy Number							
	r = 1		r = 2		r = 3		Average	
	Ratio (target/ref)	Estimated Copy Number	Ratio (target/ref)	Estimated Copy Number	Ratio (target/ref)	Estimated Copy Number	Ratio (target/ref)	Estimated Copy Number
14010220	6.76	7	6.63	7	6.18	6	6.52	7
14010221	2.33	2	3.01	3	2.91	3	2.75	3
14010225	1.12	1	1.14	1	1.06	1	1.11	1
14010229	3.95	4	4.16	4	4.02	4	4.04	4
14010230	5.53	6	10.28	10	7.06	7	7.62	8
14010237	1.27	1	1.43	1	1.63	2	1.44	1
14010242	1.30	1	1.56	2	1.39	1	1.42	1
14010246	2.33	2	1.96	2	2.16	2	2.15	2
14010247	2.18	2	2.09	2	2.31	2	2.19	2
14010250	1.34	1	1.25	1	1.35	1	1.31	1
14010251	3.98	4	3.35	3	3.05	3	3.46	3
14010252	1.56	2	1.64	2	1.76	2	1.65	2
14010253	7.01	7	5.44	5	5.17	5	5.87	6
14010258	3.46	3	3.07	3	2.62	3	3.05	3
14010259	3.41	3	4.03	4	12.94	13	6.79	7
14010265	3.33	3	3.44	3	3.86	4	3.54	4
14010267	5.79	6	5.51	6	4.70	5	5.33	5
14010268	4.38	4	5.19	5	5.49	5	5.02	5
14010271	11.50	12	10.87	11	12.28	12	11.55	12

Sample ID	Copy Number							
	r = 1		r = 2		r = 3		Average	
	Ratio (target/ref)	Estimated Copy Number	Ratio (target/ref)	Estimated Copy Number	Ratio (target/ref)	Estimated Copy Number	Ratio (target/ref)	Estimated Copy Number
14010272	10.36	10	9.24	9	9.78	10	9.79	10
14010273	4.99	5	4.18	4	3.44	3	4.20	4
14010274	1.14	1	1.12	1	1.15	1	1.14	1
14010275	2.54	3	3.06	3	2.50	2	2.70	3
14010276	4.72	5	4.68	5	4.35	4	4.58	5
14010277	5.79	6	6.47	6	6.80	7	6.36	6
14010278	6.59	7	7.97	8	8.09	8	7.55	8
14010279	5.41	5	4.96	5	4.50	5	4.96	5
14010282	4.58	5	4.11	4	4.18	4	4.29	4
14010283	2.53	3	2.68	3	1.66	2	2.29	2
14010285	2.72	3	2.56	3	2.49	2	2.59	3
14010287	1.63	2	1.10	1	1.49	1	1.41	1
14010289	1.15	1	1.15	1	0.77	1	1.02	1
14010291	0.96	1	0.63	1	1.01	1	0.87	1
14010292	1.97	2	2.10	2	1.97	2	2.02	2
14010293	0.95	1	0.76	1	1.02	1	0.91	1
14010294	0.93	1	0.87	1	1.09	1	0.96	1
14010296	1.14	1	1.01	1	1.00	1	1.05	1
14010297	0.80	1	1.37	1	1.25	1	1.14	1

Sample ID	Copy Number							
	r = 1		r = 2		r = 3		Average	
	Ratio (target/ref)	Estimated Copy Number	Ratio (target/ref)	Estimated Copy Number	Ratio (target/ref)	Estimated Copy Number	Ratio (target/ref)	Estimated Copy Number
14010299	1.54	2	1.60	2	1.60	2	1.58	2
14010305	1.32	1	1.37	1	1.47	1	1.39	1
14010311	3.40	3	3.06	3	2.74	3	3.07	3
14010314	3.30	3	3.42	3	3.30	3	3.34	3
14010319	2.22	2	3.27	3	3.59	4	3.03	3
14010320	7.55	8	8.04	8	8.18	8	7.92	8
14010321	2.01	2	2.12	2	2.22	2	2.12	2
14010323	1.33	1	1.38	1	1.43	1	1.38	1
14010341	6.53	7	6.19	6	6.68	7	6.47	6
14010345	5.53	6	5.33	5	5.53	6	5.46	5
14010347	22.50	23	21.53	22	22.31	22	22.11	22
14010350	5.09	5	5.68	6	5.73	6	5.50	5
14010351	5.25	5	3.81	4	4.32	4	4.46	4
14010354	1.89	2	1.70	2	1.68	2	1.76	2
14010355	2.06	2	2.05	2	1.83	2	1.98	2
14010357	4.39	4	4.22	4	4.13	4	4.25	4
14010363	1.38	1	1.38	1	1.45	1	1.41	1
14010368	1.20	1	1.07	1	1.07	1	1.11	1
14010369	4.11	4	4.11	4	4.29	4	4.17	4

Sample ID	Copy Number							
	r = 1		r = 2		r = 3		Average	
	Ratio (target/ref)	Estimated Copy Number	Ratio (target/ref)	Estimated Copy Number	Ratio (target/ref)	Estimated Copy Number	Ratio (target/ref)	Estimated Copy Number
14010370	6.53	7	6.41	6	6.36	6	6.43	6
14010375	1.30	1	1.35	1	1.57	2	1.41	1
14010376	3.19	3	2.97	3	3.42	3	3.19	3
14010377	1.09	1	1.03	1	0.99	1	1.04	1
14010378	0.96	1	1.15	1	1.15	1	1.09	1
14010380	3.79	4	3.75	4	3.83	4	3.79	4
14010382	4.99	5	4.75	5	6.32	6	5.35	5
14010388	0.05	0	0.07	0	0.06	0	0.06	0
14010392	1.74	2	1.85	2	2.03	2	1.87	2
14010393	1.08	1	1.23	1	1.34	1	1.22	1
14010394	1.00	1	1.00	1	0.11	0	0.71	1
14010395	1.34	1	0.98	1	0.27	0	0.86	1
14010399	1.79	2	2.23	2	1.83	2	1.95	2
14010400	1.84	2	1.97	2	1.96	2	1.92	2
14010401	1.03	1	1.07	1	1.47	1	1.19	1
14010404	1.99	2	2.01	2	2.33	2	2.11	2
14010406	5.73	6	8.77	9	5.57	6	6.69	7
14010407	0.96	1	1.29	1	1.17	1	1.14	1
14010411	0.95	1	1.02	1	0.91	1	0.96	1

Sample ID	Copy Number							
	r = 1		r = 2		r = 3		Average	
	Ratio (target/ref)	Estimated Copy Number	Ratio (target/ref)	Estimated Copy Number	Ratio (target/ref)	Estimated Copy Number	Ratio (target/ref)	Estimated Copy Number
14010412	2.43	2	3.22	3	2.20	2	2.62	3
14010413	3.00	3	2.79	3	2.87	3	2.89	3
14010414	2.42	2	2.81	3	3.14	3	2.79	3
14010418	1.07	1	1.25	1	1.42	1	1.25	1
14010425	1.91	2	1.98	2	1.94	2	1.94	2
14010427	0.67	1	0.68	1	0.68	1	0.68	1
14010430	1.34	1	0.94	1	0.98	1	1.08	1
14010431	2.45	2	2.06	2	1.95	2	2.15	2
14010432	7.51	8	8.13	8	6.27	6	7.30	7
14010437	3.83	4	4.02	4	3.52	4	3.79	4
14010438	2.00	2	1.75	2	1.67	2	1.81	2
14010441	16.08	16	13.35	13	12.49	12	13.97	14
14010444	1.10	1	0.93	1	0.80	1	0.94	1
14010445	1.63	2	1.83	2	1.68	2	1.72	2
14010450	1.11	1	0.89	1	0.91	1	0.97	1
14010452	4.82	5	4.09	4	4.73	5	4.55	5
14010455	2.12	2	2.36	2	2.22	2	2.23	2
14010458	1.09	1	1.14	1	1.12	1	1.12	1
14010460	0.86	1	0.92	1	0.92	1	0.90	1

Sample ID	Copy Number							
	r = 1		r = 2		r = 3		Average	
	Ratio (target/ref)	Estimated Copy Number	Ratio (target/ref)	Estimated Copy Number	Ratio (target/ref)	Estimated Copy Number	Ratio (target/ref)	Estimated Copy Number
14010462	5.94	6	7.08	7	7.16	7	6.73	7
14010463	5.47	5	5.59	6	6.43	6	5.83	6
14010465	9.50	9	10.37	10	10.85	11	10.24	10
14010473	1.03	1	1.07	1	1.03	1	1.04	1
14010477	1.52	2	1.48	1	1.47	1	1.49	1
14010478	1.20	1	1.26	1	1.18	1	1.21	1
14010480	1.16	1	1.11	1	1.08	1	1.12	1
14010484	0.03	0	0.04	0	0.02	0	0.03	0
14010491	1.83	2	1.75	2	1.62	2	1.73	2
14010492	1.63	2	1.90	2	1.69	2	1.74	2
14010493	2.78	3	2.69	3	3.74	4	3.07	3
14010500	2.03	2	2.15	2	2.24	2	2.14	2
14010503	3.09	3	3.30	3	3.34	3	3.24	3
14010504	2.75	3	2.95	3	3.26	3	2.98	3
14010505	0.88	1	0.84	1	1.17	1	0.97	1
14010508	3.67	4	3.42	3	3.39	3	3.50	3
14010509	3.20	3	2.61	3	2.95	3	2.92	3
14010510	2.05	2	2.25	2	2.03	2	2.11	2
14010516	2.70	3	3.67	4	3.61	4	3.33	3

Sample ID	Copy Number							
	r = 1		r = 2		r = 3		Average	
	Ratio (target/ref)	Estimated Copy Number	Ratio (target/ref)	Estimated Copy Number	Ratio (target/ref)	Estimated Copy Number	Ratio (target/ref)	Estimated Copy Number
14010519	5.94	6	5.24	5	5.32	5	5.50	5
14010520	5.84	6	4.67	5	4.62	5	5.04	5
14010521	1.20	1	1.31	1	1.19	1	1.23	1
14010522	0.65	1	1.04	1	1.15	1	0.94	1
14010524	3.45	3	2.47	2	2.98	3	2.97	3
14010525	1.62	2	1.54	2	1.56	2	1.57	2
14010527	4.90	5	4.88	5	4.79	5	4.86	5
14010536	1.41	1	1.54	2	1.59	2	1.52	2
14010537	4.74	5	4.93	5	5.36	5	5.01	5
14010538	2.88	3	3.17	3	3.70	4	3.25	3
14010539	3.01	3	3.27	3	3.70	4	3.32	3
14010541	4.47	4	4.76	5	4.77	5	4.67	5
14010544	2.09	2	1.90	2	2.05	2	2.01	2
14010545	0.89	1	0.84	1	0.82	1	0.85	1
14010548	2.73	3	2.63	3	2.50	3	2.62	3
14010553	0.86	1	0.86	1	0.71	1	0.81	1
14010554	1.38	1	1.40	1	1.19	1	1.33	1
14010555	1.84	2	1.95	2	1.99	2	1.93	2
14010557	4.87	5	4.51	5	4.43	4	4.60	5

Sample ID	Copy Number							
	r = 1		r = 2		r = 3		Average	
	Ratio (target/ref)	Estimated Copy Number	Ratio (target/ref)	Estimated Copy Number	Ratio (target/ref)	Estimated Copy Number	Ratio (target/ref)	Estimated Copy Number
14010558	3.59	4	3.19	3	3.03	3	3.27	3
14010559	1.59	2	1.52	2	1.38	1	1.50	1
14010561	11.46	11	11.85	12	12.65	13	11.99	12
14010564	1.11	1	0.95	1	0.91	1	0.99	1
14010567	1.35	1	1.74	2	1.57	2	1.55	2
14010568	0.27	0	0.31	0	0.30	0	0.30	0
14010571	1.84	2	1.97	2	2.15	2	1.98	2
14010572	3.90	4	4.23	4	3.59	4	3.91	4
14010573	2.07	2	1.90	2	1.88	2	1.95	2
14010576	3.03	3	2.75	3	2.92	3	2.90	3
14010578	6.11	6	4.51	5	4.85	5	5.16	5
14010580	1.54	2	1.53	2	1.60	2	1.56	2
14010581	4.85	5	4.57	5	4.08	4	4.50	4
14010584	2.55	3	2.78	3	2.83	3	2.72	3
14010585	1.28	1	1.30	1	1.36	1	1.32	1
14010586	3.55	4	3.39	3	3.62	4	3.52	4
14010587	3.20	3	3.34	3	3.49	3	3.34	3
14010588	1.35	1	1.31	1	1.47	1	1.38	1
14010590	1.72	2	1.56	2	2.10	2	1.79	2

Sample ID	Copy Number							
	r = 1		r = 2		r = 3		Average	
	Ratio (target/ref)	Estimated Copy Number	Ratio (target/ref)	Estimated Copy Number	Ratio (target/ref)	Estimated Copy Number	Ratio (target/ref)	Estimated Copy Number
14010594	1.23	1	1.35	1	1.31	1	1.29	1
14010596	12.42	12	11.30	11	9.85	10	11.19	11
14010597	10.64	11	11.43	11	8.99	9	10.35	10
14010600	3.34	3	3.28	3	3.08	3	3.23	3
14010601	1.31	1	1.47	1	1.22	1	1.34	1
14010602	1.38	1	1.34	1	1.23	1	1.32	1
14010603	1.43	1	1.29	1	1.24	1	1.32	1
14010604	1.56	2	1.45	1	1.30	1	1.44	1
14010606	2.99	3	2.53	3	2.45	2	2.66	3
14010607	1.10	1	1.17	1	1.28	1	1.18	1
14010610	2.46	2	2.51	3	2.46	2	2.48	2
14010611	2.53	3	2.54	3	2.65	3	2.57	3
14010613	2.69	3	3.31	3	3.21	3	3.07	3
14010614	5.06	5	5.27	5	5.12	5	5.15	5
14010616	1.18	1	1.29	1	0.97	1	1.15	1
14010621	0.89	1	1.62	2	1.40	1	1.30	1
14010622	1.17	1	1.27	1	1.02	1	1.15	1
14010623	9.79	10	8.71	9	9.21	9	9.24	9
14010624	1.81	2	1.59	2	1.42	1	1.61	2

Sample ID	Copy Number							
	r = 1		r = 2		r = 3		Average	
	Ratio (target/ref)	Estimated Copy Number	Ratio (target/ref)	Estimated Copy Number	Ratio (target/ref)	Estimated Copy Number	Ratio (target/ref)	Estimated Copy Number
14010625	1.77	2	1.42	1	1.37	1	1.52	2
14010627	1.51	2	1.87	2	1.84	2	1.74	2
14010628	2.07	2	1.60	2	1.51	2	1.72	2
14010629	1.48	1	1.62	2	1.68	2	1.59	2
14010630	1.22	1	1.16	1	1.38	1	1.25	1
14010633	1.36	1	1.35	1	1.44	1	1.38	1
14010638	1.00	1	0.76	1	0.73	1	0.83	1
14010640	4.88	5	4.20	4	4.96	5	4.68	5
14010641	1.51	2	1.71	2	1.80	2	1.67	2
14010645	1.07	1	1.00	1	1.00	1	1.02	1
14010646	1.00	1	1.16	1	1.22	1	1.13	1
14010653	2.05	2	1.92	2	1.34	1	1.77	2
14010655	1.30	1	1.51	2	1.17	1	1.32	1
14010662	4.26	4	1.39	1	1.58	2	2.41	2
14010663	5.21	5	5.09	5	4.74	5	5.01	5
14010666	3.45	3	4.70	5	2.93	3	3.69	4
14010669	4.18	4	3.22	3	3.62	4	3.67	4
14010684	1.68	2	1.62	2	1.46	1	1.58	2
14010690	1.52	2	1.32	1	1.20	1	1.35	1

Sample ID	Copy Number							
	r = 1		r = 2		r = 3		Average	
	Ratio (target/ref)	Estimated Copy Number	Ratio (target/ref)	Estimated Copy Number	Ratio (target/ref)	Estimated Copy Number	Ratio (target/ref)	Estimated Copy Number
14010692	1.22	1	1.06	1	1.00	1	1.09	1
14010693	1.26	1	1.18	1	1.22	1	1.22	1
14010695	0.95	1	0.75	1	0.87	1	0.86	1
14010697	1.09	1	0.89	1	0.86	1	0.95	1
14010702	1.80	2	1.80	2	1.69	2	1.76	2
14010704	1.03	1	0.92	1	0.89	1	0.94	1
14010705	2.84	3	2.18	2	2.46	2	2.49	2
14010714	2.42	2	1.92	2	1.78	2	2.04	2
14010715	1.20	1	1.10	1	1.06	1	1.12	1
14010717	1.29	1	1.31	1	1.31	1	1.30	1
14010721	1.73	2	1.72	2	1.68	2	1.71	2
14010723	2.17	2	2.28	2	2.23	2	2.23	2
14010725	0.88	1	0.80	1	0.80	1	0.83	1
14010726	0.80	1	0.74	1	0.84	1	0.80	1
14010727	1.52	2	1.51	2	1.46	1	1.50	1
14010729	1.78	2	1.79	2	1.83	2	1.80	2
14010730	1.93	2	1.82	2	1.99	2	1.91	2
14010731	1.98	2	1.87	2	1.91	2	1.92	2
14010732	1.88	2	1.78	2	1.84	2	1.84	2

Sample ID	Copy Number							
	r = 1		r = 2		r = 3		Average	
	Ratio (target/ref)	Estimated Copy Number	Ratio (target/ref)	Estimated Copy Number	Ratio (target/ref)	Estimated Copy Number	Ratio (target/ref)	Estimated Copy Number
14010733	0.97	1	0.90	1	0.90	1	0.92	1
14010734	1.10	1	1.17	1	1.03	1	1.10	1
14010735	1.11	1	1.70	2	1.51	2	1.44	1
14010737	0.97	1	0.79	1	0.91	1	0.89	1
14010738	3.20	3	3.07	3	3.15	3	3.14	3
14010740	1.05	1	0.95	1	0.99	1	1.00	1
14010742	1.01	1	1.08	1	1.14	1	1.08	1
14010744	2.67	3	2.17	2	2.78	3	2.54	3
14010746	4.50	4	5.43	5	5.95	6	5.29	5
14010751	1.93	2	1.88	2	2.19	2	2.00	2
14010753	1.06	1	1.14	1	1.20	1	1.13	1
14010759	3.06	3	2.60	3	2.76	3	2.81	3
14010762	1.04	1	0.93	1	0.99	1	0.99	1
14010763	4.06	4	4.19	4	3.93	4	4.06	4
14010767	3.80	4	4.04	4	3.69	4	3.84	4
14010768	1.16	1	1.07	1	1.06	1	1.09	1
14010773	2.23	2	2.54	3	2.97	3	2.58	3
14010775	3.49	3	4.07	4	4.67	5	4.08	4
14010776	3.66	4	4.09	4	3.75	4	3.83	4

Sample ID	Copy Number							
	r = 1		r = 2		r = 3		Average	
	Ratio (target/ref)	Estimated Copy Number	Ratio (target/ref)	Estimated Copy Number	Ratio (target/ref)	Estimated Copy Number	Ratio (target/ref)	Estimated Copy Number
14010779	0.81	1	0.73	1	0.42	0	0.65	1
14010780	0.93	1	1.00	1	1.22	1	1.05	1
14010782	0.93	1	0.99	1	0.91	1	0.94	1
14010785	1.01	1	1.03	1	1.27	1	1.10	1
14010786	1.09	1	1.03	1	1.13	1	1.08	1
14010789	5.47	5	5.42	5	5.82	6	5.57	6
14010799	5.75	6	6.13	6	5.77	6	5.88	6
14010803	3.48	3	4.51	5	4.50	5	4.16	4
14010808	0.94	1	1.17	1	0.89	1	1.00	1
14010810	0.99	1	1.10	1	1.04	1	1.05	1
14010811	0.06	0	0.05	0	0.04	0	0.05	0
14010815	2.08	2	1.84	2	2.01	2	1.98	2
14010821	1.68	2	1.81	2	1.84	2	1.78	2
14010823	0.89	1	1.00	1	1.03	1	0.97	1
14010824	1.83	2	1.63	2	1.90	2	1.79	2
14010825	1.05	1	1.02	1	1.06	1	1.04	1
14010826	1.08	1	1.06	1	1.16	1	1.10	1
14010827	1.01	1	0.96	1	1.00	1	0.99	1
14010829	1.14	1	0.80	1	1.03	1	0.99	1

Sample ID	Copy Number							
	r = 1		r = 2		r = 3		Average	
	Ratio (target/ref)	Estimated Copy Number	Ratio (target/ref)	Estimated Copy Number	Ratio (target/ref)	Estimated Copy Number	Ratio (target/ref)	Estimated Copy Number
14010831	3.14	3	3.19	3	3.00	3	3.11	3
14010835	1.26	1	0.91	1	1.01	1	1.06	1
14010840	3.52	4	2.61	3	2.43	2	2.85	3
14010843	1.74	2	1.72	2	1.54	2	1.66	2
14010845	1.16	1	0.99	1	0.92	1	1.02	1
14010848	1.07	1	1.06	1	1.04	1	1.06	1
14010850	10.09	10	6.05	6	8.63	9	8.26	8
14010854	0.35	0	0.38	0	0.31	0	0.35	0
14010855	2.09	2	2.25	2	1.94	2	2.09	2
14010857	1.27	1	1.40	1	1.25	1	1.31	1
14010862	1.79	2	1.52	2	1.77	2	1.69	2
14010865	0.60	1	0.66	1	0.63	1	0.63	1
14010866	0.85	1	0.89	1	0.98	1	0.90	1
14010867	1.07	1	1.05	1	0.82	1	0.98	1
14010870	2.98	3	2.85	3	2.77	3	2.87	3
14010872	3.35	3	3.57	4	3.42	3	3.45	3
14010873	8.66	9	9.56	10	9.80	10	9.34	9
14010875	1.44	1	1.20	1	1.08	1	1.24	1
14010878	1.14	1	1.10	1	1.07	1	1.10	1

Sample ID	Copy Number							
	r = 1		r = 2		r = 3		Average	
	Ratio (target/ref)	Estimated Copy Number	Ratio (target/ref)	Estimated Copy Number	Ratio (target/ref)	Estimated Copy Number	Ratio (target/ref)	Estimated Copy Number
14010881	1.45	1	0.11	0	1.55	2	1.04	1
14010882	1.58	2	1.56	2	1.67	2	1.60	2
14010884	4.02	4	4.21	4	4.19	4	4.14	4
14010886	1.08	1	1.06	1	1.07	1	1.07	1
14010888	1.24	1	1.10	1	1.27	1	1.20	1
14010889	3.37	3	3.44	3	3.46	3	3.42	3
14010890	0.90	1	1.07	1	1.08	1	1.02	1
14010892	9.19	9	9.76	10	10.74	11	9.90	10
14010895	1.09	1	1.06	1	1.09	1	1.08	1
14010897	1.95	2	1.86	2	1.98	2	1.93	2
14010898	5.38	5	5.35	5	6.06	6	5.60	6
14010899	4.29	4	4.36	4	4.27	4	4.31	4
14010900	6.24	6	5.71	6	5.98	6	5.98	6
14010901	4.34	4	4.03	4	4.69	5	4.35	4
14010904	2.13	2	2.05	2	2.09	2	2.09	2
14010905	2.01	2	2.08	2	1.72	2	1.94	2
14010908	1.68	2	1.70	2	1.80	2	1.73	2
14010909	1.46	1	1.86	2	2.30	2	1.88	2
14010915	3.45	3	2.63	3	2.75	3	2.94	3

Sample ID	Copy Number							
	r = 1		r = 2		r = 3		Average	
	Ratio (target/ref)	Estimated Copy Number	Ratio (target/ref)	Estimated Copy Number	Ratio (target/ref)	Estimated Copy Number	Ratio (target/ref)	Estimated Copy Number
14010916	4.82	5	2.96	3	3.76	4	3.85	4
14010919	1.74	2	1.64	2	1.63	2	1.67	2
14010921	1.10	1	1.07	1	1.08	1	1.08	1
14010924	1.01	1	0.99	1	1.02	1	1.01	1
14010926	2.29	2	2.67	3	2.49	2	2.48	2
14010928	1.04	1	0.95	1	1.17	1	1.06	1
14010930	0.89	1	0.80	1	0.94	1	0.88	1
14010932	0.04	0	0.04	0	0.03	0	0.04	0
14010934	1.04	1	1.07	1	1.03	1	1.05	1
14010938	0.76	1	0.84	1	0.85	1	0.82	1
14010941	0.81	1	0.84	1	1.10	1	0.91	1
14010948	0.94	1	0.87	1	1.05	1	0.95	1
14010950	0.94	1	1.00	1	1.32	1	1.09	1
14010952	2.69	3	3.94	4	2.69	3	3.11	3
14010955	4.92	5	4.09	4	4.40	4	4.47	4
14010956	4.86	5	4.71	5	3.87	4	4.48	4
14010963	1.38	1	1.41	1	1.20	1	1.33	1
14010971	2.42	2	2.14	2	1.82	2	2.13	2
14010972	2.26	2	2.07	2	1.94	2	2.09	2

Sample ID	Copy Number							
	r = 1		r = 2		r = 3		Average	
	Ratio (target/ref)	Estimated Copy Number	Ratio (target/ref)	Estimated Copy Number	Ratio (target/ref)	Estimated Copy Number	Ratio (target/ref)	Estimated Copy Number
14010973	2.97	3	3.53	4	2.47	2	2.99	3
14010976	1.00	1	1.10	1	1.08	1	1.06	1
14010978	3.69	4	3.66	4	3.31	3	3.55	4
14010979	1.49	1	1.35	1	1.29	1	1.37	1
14010987	2.59	3	2.62	3	1.55	2	2.25	2
14010996	0.52	1	0.45	0	0.53	1	0.50	0
14010997	1.79	2	2.94	3	3.06	3	2.60	3
14011002	1.15	1	1.26	1	1.27	1	1.22	1
14011003	2.49	2	2.67	3	2.84	3	2.66	3
14011006	1.40	1	1.12	1	1.27	1	1.26	1
14011007	2.37	2	2.51	3	2.66	3	2.51	3
14011008	2.25	2	2.13	2	2.40	2	2.26	2
14011011	2.36	2	2.62	3	2.61	3	2.53	3
14011012	2.97	3	2.73	3	2.76	3	2.82	3
14011018	1.38	1	1.22	1	1.28	1	1.29	1
14011019	7.52	8	6.53	7	6.55	7	6.87	7
14011022	3.30	3	3.40	3	3.27	3	3.32	3
14011028	1.18	1	1.25	1	1.02	1	1.15	1
14011029	1.35	1	1.20	1	1.15	1	1.23	1

Sample ID	Copy Number							
	r = 1		r = 2		r = 3		Average	
	Ratio (target/ref)	Estimated Copy Number	Ratio (target/ref)	Estimated Copy Number	Ratio (target/ref)	Estimated Copy Number	Ratio (target/ref)	Estimated Copy Number
14011030	1.50	2	1.25	1	1.16	1	1.31	1
14011035	2.92	3	2.75	3	2.47	2	2.71	3
14011037	0.62	1	1.31	1	0.62	1	0.85	1
14011041	2.40	2	2.01	2	1.74	2	2.05	2
14011046	1.02	1	1.11	1	1.00	1	1.04	1
14011054	3.58	4	4.16	4	5.59	6	4.44	4
14011055	1.29	1	0.97	1	1.08	1	1.11	1
14011057	1.37	1	1.55	2	1.55	2	1.49	1
14011060	1.51	2	1.27	1	1.57	2	1.45	1
14011061	1.68	2	2.06	2	2.40	2	2.04	2
14011065	3.27	3	3.58	4	3.62	4	3.49	3
14011066	1.45	1	1.31	1	0.96	1	1.24	1
14011067	4.78	5	4.16	4	5.18	5	4.71	5
14011069	1.81	2	1.90	2	1.85	2	1.85	2
14011070	1.54	2	1.55	2	1.83	2	1.64	2
14011071	4.18	4	4.67	5	4.14	4	4.33	4
14011073	1.40	1	1.40	1	1.41	1	1.40	1
14011074	1.38	1	1.58	2	1.66	2	1.54	2
14011075	2.13	2	2.41	2	1.93	2	2.15	2

Sample ID	Copy Number							
	r = 1		r = 2		r = 3		Average	
	Ratio (target/ref)	Estimated Copy Number	Ratio (target/ref)	Estimated Copy Number	Ratio (target/ref)	Estimated Copy Number	Ratio (target/ref)	Estimated Copy Number
14011077	4.20	4	4.95	5	6.15	6	5.10	5
14011078	1.02	1	1.12	1	1.08	1	1.07	1
14011081	16.15	16	14.43	14	19.50	19	16.69	17
14011082	1.72	2	1.78	2	1.44	1	1.65	2
14011083	3.82	4	3.77	4	4.32	4	3.97	4
14011084	5.18	5	5.34	5	4.72	5	5.08	5
14011089	2.91	3	2.95	3	2.92	3	2.93	3
14011090	6.65	7	5.91	6	5.20	5	5.92	6
14011091	7.28	7	7.80	8	7.05	7	7.37	7
14011092	8.95	9	8.42	8	7.91	8	8.43	8
14011094	3.87	4	3.19	3	2.70	3	3.25	3
14011095	1.94	2	2.40	2	2.19	2	2.18	2
14011097	1.30	1	1.07	1	1.10	1	1.16	1
14011098	1.50	1	1.23	1	1.40	1	1.38	1
14011100	9.39	9	9.55	10	7.42	7	8.79	9
14011101	4.28	4	4.27	4	3.94	4	4.16	4
14011103	4.85	5	4.86	5	4.69	5	4.80	5
14011106	1.58	2	1.74	2	1.60	2	1.64	2
14011110	3.53	4	3.57	4	3.38	3	3.49	3

Sample ID	Copy Number							
	r = 1		r = 2		r = 3		Average	
	Ratio (target/ref)	Estimated Copy Number	Ratio (target/ref)	Estimated Copy Number	Ratio (target/ref)	Estimated Copy Number	Ratio (target/ref)	Estimated Copy Number
14020010	5.38	5	4.49	4	5.39	5	5.09	5
14020011	2.51	3	2.51	3	2.65	3	2.56	3
14020012	1.13	1	1.23	1	1.16	1	1.17	1
14020013	1.86	2	1.83	2	2.27	2	1.99	2
14020015	7.14	7	6.33	6	7.27	7	6.91	7
14020016	1.03	1	1.25	1	1.21	1	1.16	1
14020017	1.76	2	1.97	2	1.95	2	1.89	2
14020021	1.80	2	2.00	2	2.00	2	1.93	2
14020022	2.60	3	2.49	2	2.45	2	2.52	3
14020023	1.70	2	1.83	2	2.32	2	1.95	2
14020024	1.32	1	1.31	1	1.14	1	1.26	1
14020027	1.36	1	1.18	1	1.30	1	1.28	1
14020042	1.17	1	1.00	1	1.08	1	1.08	1
14020043	5.37	5	4.14	4	4.70	5	4.74	5
14020045	1.35	1	1.53	2	1.11	1	1.33	1
14020046	4.12	4	4.73	5	5.02	5	4.63	5
14020049	1.11	1	0.93	1	1.13	1	1.06	1
14020050	1.10	1	1.04	1	0.95	1	1.03	1
14020053	1.27	1	1.23	1	1.40	1	1.30	1

Sample ID	Copy Number							
	r = 1		r = 2		r = 3		Average	
	Ratio (target/ref)	Estimated Copy Number	Ratio (target/ref)	Estimated Copy Number	Ratio (target/ref)	Estimated Copy Number	Ratio (target/ref)	Estimated Copy Number
14020054	5.55	6	4.90	5	4.85	5	5.10	5
14020055	2.54	3	2.56	3	2.92	3	2.67	3
14020056	2.56	3	1.88	2	2.19	2	2.21	2
14020058	2.35	2	1.99	2	2.14	2	2.16	2
14020059	2.79	3	2.75	3	3.10	3	2.88	3
14020060	1.09	1	1.07	1	1.13	1	1.10	1
14020061	2.03	2	1.66	2	2.20	2	1.96	2
14020065	0.98	1	1.04	1	1.01	1	1.01	1
14020069	1.28	1	1.28	1	1.34	1	1.30	1
14020072	1.20	1	1.32	1	1.28	1	1.27	1
14020073	3.05	3	3.30	3	2.91	3	3.09	3
14020081	5.11	5	4.16	4	4.16	4	4.48	4
14020082	1.12	1	1.27	1	1.23	1	1.21	1
14020083	8.63	9	8.63	9	9.62	10	8.96	9
14020087	2.13	2	2.09	2	2.16	2	2.13	2
14020088	1.51	2	1.45	1	1.66	2	1.54	2
14020090	3.49	3	3.71	4	3.44	3	3.55	4
14020093	0.40	0	0.37	0	0.40	0	0.39	0
14020100	1.18	1	1.17	1	1.07	1	1.14	1

Sample ID	Copy Number							
	r = 1		r = 2		r = 3		Average	
	Ratio (target/ref)	Estimated Copy Number	Ratio (target/ref)	Estimated Copy Number	Ratio (target/ref)	Estimated Copy Number	Ratio (target/ref)	Estimated Copy Number
14020101	2.18	2	2.20	2	2.16	2	2.18	2
14020102	2.34	2	2.30	2	2.07	2	2.24	2
14020103	0.54	1	0.49	0	0.52	1	0.52	1
14020105	4.97	5	5.59	6	5.89	6	5.48	5
14020113	3.59	4	3.98	4	4.41	4	3.99	4
14020114	1.09	1	1.20	1	1.14	1	1.15	1
14020116	2.07	2	2.29	2	2.05	2	2.14	2
14020117	5.79	6	4.69	5	4.67	5	5.05	5
14020118	2.05	2	2.62	3	2.27	2	2.32	2
14020119	1.99	2	1.52	2	1.51	2	1.67	2
14020121	2.29	2	2.15	2	1.96	2	2.13	2
14020126	1.46	1	1.26	1	1.44	1	1.39	1
14020127	3.53	4	2.92	3	2.61	3	3.02	3
14020128	4.68	5	3.44	3	2.95	3	3.69	4
14020131	2.93	3	3.29	3	2.99	3	3.07	3
14020132	2.88	3	3.20	3	2.88	3	2.99	3
14020134	1.88	2	1.79	2	1.54	2	1.73	2
14020135	2.39	2	1.93	2	2.06	2	2.13	2
14020146	4.47	4	4.58	5	4.22	4	4.42	4

Sample ID	Copy Number							
	r = 1		r = 2		r = 3		Average	
	Ratio (target/ref)	Estimated Copy Number	Ratio (target/ref)	Estimated Copy Number	Ratio (target/ref)	Estimated Copy Number	Ratio (target/ref)	Estimated Copy Number
14020147	5.17	5	4.88	5	4.87	5	4.97	5
14020150	2.51	3	2.80	3	3.03	3	2.78	3
14020152	3.27	3	3.39	3	3.21	3	3.29	3
14020157	1.77	2	1.99	2	2.16	2	1.98	2
14020158	2.65	3	2.95	3	3.36	3	2.99	3
14020159	9.17	9	11.49	11	12.47	12	11.04	11
14020160	9.41	9	11.29	11	12.89	13	11.20	11
14020161	7.53	8	7.74	8	9.29	9	8.18	8
14020162	3.94	4	3.66	4	3.74	4	3.78	4
14020165	1.69	2	1.45	1	1.36	1	1.50	1
14020166	1.81	2	1.53	2	1.17	1	1.50	2
14020169	1.64	2	1.60	2	1.30	1	1.51	2
14020170	1.35	1	1.13	1	1.22	1	1.23	1
14020172	6.43	6	5.84	6	6.24	6	6.17	6
14020174	4.09	4	3.61	4	3.81	4	3.84	4
14020175	2.31	2	2.25	2	2.11	2	2.22	2
14020176	1.88	2	1.53	2	1.47	1	1.63	2
14020182	2.68	3	3.30	3	2.72	3	2.90	3
14020190	1.27	1	0.97	1	1.20	1	1.15	1

Sample ID	Copy Number							
	r = 1		r = 2		r = 3		Average	
	Ratio (target/ref)	Estimated Copy Number	Ratio (target/ref)	Estimated Copy Number	Ratio (target/ref)	Estimated Copy Number	Ratio (target/ref)	Estimated Copy Number
14020192	1.60	2	1.81	2	1.59	2	1.67	2
14020194	1.47	1	0.86	1	1.15	1	1.16	1
14020201	5.39	5	5.70	6	5.94	6	5.68	6
14020203	1.33	1	1.48	1	1.30	1	1.37	1
14020205	1.89	2	1.84	2	1.89	2	1.87	2
14020207	1.09	1	1.13	1	0.98	1	1.07	1
14020208	2.21	2	1.83	2	1.63	2	1.89	2
14020209	1.60	2	1.52	2	1.53	2	1.55	2
14020210	2.63	3	2.64	3	2.33	2	2.53	3
14020211	3.07	3	3.05	3	2.92	3	3.01	3
14020217	1.96	2	2.02	2	1.95	2	1.98	2
14020218	1.36	1	1.46	1	1.51	2	1.44	1
14020219	1.23	1	1.32	1	1.12	1	1.22	1
14020222	1.13	1	1.13	1	1.14	1	1.13	1
14020223	1.05	1	1.16	1	1.04	1	1.08	1
14020227	1.17	1	1.20	1	1.08	1	1.15	1
14020229	1.18	1	1.15	1	1.08	1	1.14	1
14020231	2.32	2	2.33	2	2.25	2	2.30	2
14020232	2.98	3	3.56	4	2.99	3	3.18	3

Sample ID	Copy Number							
	r = 1		r = 2		r = 3		Average	
	Ratio (target/ref)	Estimated Copy Number	Ratio (target/ref)	Estimated Copy Number	Ratio (target/ref)	Estimated Copy Number	Ratio (target/ref)	Estimated Copy Number
14020235	3.46	3	3.13	3	3.28	3	3.29	3
14020236	2.69	3	2.74	3	2.84	3	2.76	3
14020239	5.93	6	1.75	2	3.04	3	3.57	4
14020241	1.31	1	1.09	1	0.99	1	1.13	1
14020242	2.27	2	1.99	2	1.78	2	2.01	2
14020246	3.40	3	3.84	4	4.31	4	3.85	4
14020249	1.12	1	1.17	1	1.19	1	1.16	1
14020250	3.40	3	3.99	4	4.01	4	3.80	4
14020251	4.87	5	5.06	5	4.92	5	4.95	5
14020252	3.44	3	4.29	4	3.95	4	3.89	4
14020253	4.46	4	4.84	5	4.13	4	4.48	4
14020254	3.90	4	3.56	4	3.57	4	3.67	4
14020255	2.77	3	2.63	3	3.23	3	2.88	3
14020258	1.60	2	2.25	2	2.27	2	2.04	2
14020259	2.16	2	2.41	2	2.49	2	2.35	2
14020260	1.09	1	1.25	1	1.34	1	1.23	1
14020261	0.63	1	0.66	1	0.71	1	0.67	1
14020263	0.50	1	0.68	1	0.59	1	0.59	1
14020264	1.06	1	1.14	1	1.09	1	1.10	1

Sample ID	Copy Number							
	r = 1		r = 2		r = 3		Average	
	Ratio (target/ref)	Estimated Copy Number	Ratio (target/ref)	Estimated Copy Number	Ratio (target/ref)	Estimated Copy Number	Ratio (target/ref)	Estimated Copy Number
14020265	2.18	2	1.96	2	1.91	2	2.02	2
14020273	1.24	1	1.07	1	1.33	1	1.21	1
14020274	0.99	1	0.94	1	1.21	1	1.04	1
14020275	4.52	5	4.46	4	4.20	4	4.39	4
14020276	4.07	4	3.96	4	4.58	5	4.21	4
14020280	4.28	4	4.35	4	3.94	4	4.19	4
14020283	2.86	3	2.48	2	3.30	3	2.88	3
14020286	2.33	2	3.14	3	2.72	3	2.73	3
14020288	0.98	1	1.06	1	1.27	1	1.10	1
14020290	2.76	3	3.06	3	2.84	3	2.89	3
14020291	3.47	3	3.01	3	3.04	3	3.18	3
14020293	4.13	4	4.33	4	4.45	4	4.30	4
14020294	3.34	3	3.98	4	4.34	4	3.89	4
14020295	3.70	4	3.99	4	5.04	5	4.25	4
14020296	2.90	3	3.20	3	3.07	3	3.05	3
14020300	2.83	3	2.94	3	2.97	3	2.91	3
14020301	0.55	1	0.67	1	0.58	1	0.60	1
14020302	1.25	1	1.22	1	1.27	1	1.25	1
14020303	2.03	2	2.10	2	2.36	2	2.16	2

Sample ID	Copy Number							
	r = 1		r = 2		r = 3		Average	
	Ratio (target/ref)	Estimated Copy Number	Ratio (target/ref)	Estimated Copy Number	Ratio (target/ref)	Estimated Copy Number	Ratio (target/ref)	Estimated Copy Number
14020304	0.99	1	1.12	1	1.21	1	1.11	1
14020305	1.18	1	1.27	1	1.34	1	1.26	1
14020309	2.21	2	3.07	3	2.61	3	2.63	3
14020310	1.46	1	1.50	1	1.42	1	1.46	1
14020311	5.09	5	5.73	6	5.89	6	5.57	6
14020319	4.25	4	3.53	4	4.15	4	3.98	4
14020320	4.06	4	4.05	4	3.48	3	3.86	4
14020321	3.86	4	3.84	4	3.43	3	3.71	4
14020322	2.16	2	2.01	2	2.49	2	2.22	2
14020323	2.03	2	2.13	2	2.37	2	2.18	2
14020325	2.60	3	2.59	3	2.29	2	2.49	2
14020326	2.90	3	2.96	3	1.54	2	2.47	2
14020328	1.13	1	1.21	1	0.78	1	1.04	1
14020330	2.12	2	1.51	2	1.97	2	1.87	2
14020331	0.63	1	0.69	1	0.61	1	0.64	1
14020337	0.96	1	0.82	1	0.72	1	0.83	1
14020339	2.62	3	2.49	2	2.55	3	2.55	3
14020340	0.08	0	0.08	0	0.05	0	0.07	0
14020341	1.56	2	1.77	2	1.64	2	1.66	2

Sample ID	Copy Number							
	r = 1		r = 2		r = 3		Average	
	Ratio (target/ref)	Estimated Copy Number	Ratio (target/ref)	Estimated Copy Number	Ratio (target/ref)	Estimated Copy Number	Ratio (target/ref)	Estimated Copy Number
14020342	2.16	2	2.38	2	2.15	2	2.23	2
14020350	1.78	2	1.75	2	1.78	2	1.77	2
14020357	8.58	9	7.45	7	7.48	7	7.84	8
14020377	3.99	4	2.75	3	2.92	3	3.22	3
14020383	2.10	2	2.00	2	2.37	2	2.16	2
14020385	2.83	3	2.48	2	2.86	3	2.72	3
14020391	1.24	1	1.16	1	1.12	1	1.17	1
14020401	1.16	1	1.31	1	1.05	1	1.17	1
14020402	7.09	7	7.25	7	8.02	8	7.46	7
14020404	4.20	4	4.11	4	4.48	4	4.26	4
14020406	1.98	2	2.10	2	2.02	2	2.03	2
14020407	3.67	4	3.57	4	4.15	4	3.80	4
14020408	1.04	1	1.02	1	1.42	1	1.16	1
14020411	1.16	1	1.07	1	1.02	1	1.08	1
14020412	1.79	2	1.94	2	1.70	2	1.81	2
14020413	2.24	2	2.21	2	2.30	2	2.25	2
14020414	1.92	2	1.81	2	1.97	2	1.90	2
14020415	1.65	2	1.50	2	1.75	2	1.64	2
14020428	2.79	3	2.86	3	3.01	3	2.89	3

Sample ID	Copy Number							
	r = 1		r = 2		r = 3		Average	
	Ratio (target/ref)	Estimated Copy Number	Ratio (target/ref)	Estimated Copy Number	Ratio (target/ref)	Estimated Copy Number	Ratio (target/ref)	Estimated Copy Number
14020429	2.66	3	2.79	3	3.20	3	2.89	3
14020432	1.04	1	1.13	1	0.86	1	1.01	1
14020434	3.32	3	3.66	4	3.50	4	3.50	3
14020436	2.67	3	2.62	3	2.54	3	2.61	3
14020437	2.77	3	2.57	3	2.32	2	2.56	3
14020438	2.39	2	2.26	2	2.04	2	2.23	2
14020439	2.20	2	2.08	2	1.79	2	2.02	2
14020440	4.97	5	4.26	4	4.17	4	4.47	4
14020443	0.80	1	0.80	1	0.85	1	0.82	1
14020444	1.43	1	1.08	1	1.11	1	1.21	1
14020448	1.79	2	1.96	2	1.82	2	1.86	2
14020451	4.63	5	4.94	5	4.35	4	4.64	5
14020452	2.15	2	2.22	2	2.30	2	2.22	2
14020458	3.05	3	3.04	3	2.81	3	2.97	3
14020464	1.89	2	1.66	2	1.84	2	1.80	2
14020465	2.12	2	1.92	2	2.03	2	2.02	2
14020470	1.95	2	1.86	2	2.04	2	1.95	2
14020471	2.82	3	2.23	2	2.56	3	2.54	3
14020473	1.54	2	1.62	2	1.84	2	1.67	2

Sample ID	Copy Number							
	r = 1		r = 2		r = 3		Average	
	Ratio (target/ref)	Estimated Copy Number	Ratio (target/ref)	Estimated Copy Number	Ratio (target/ref)	Estimated Copy Number	Ratio (target/ref)	Estimated Copy Number
14020474	0.34	0	0.36	0	0.37	0	0.36	0
14020475	1.28	1	1.19	1	1.13	1	1.20	1
14020476	1.81	2	1.86	2	1.83	2	1.84	2
14020477	0.73	1	0.76	1	0.72	1	0.73	1
14020481	20.17	20	24.02	24	23.03	23	22.41	22
14020489	0.00	0	0.00	0	0.00	0	0.00	0
14020490	3.29	3	3.07	3	3.01	3	3.12	3
14020491	3.41	3	3.81	4	3.92	4	3.71	4
14020492	7.84	8	7.52	8	8.72	9	8.03	8
14020495	0.93	1	0.96	1	1.06	1	0.98	1
14020502	1.20	1	1.16	1	1.39	1	1.25	1
14020503	6.58	7	5.48	5	5.87	6	5.97	6
14020504	2.81	3	3.75	4	3.46	3	3.34	3
14020505	3.48	3	2.56	3	2.88	3	2.97	3
14020507	2.66	3	2.66	3	3.02	3	2.78	3
14020510	2.13	2	2.03	2	1.79	2	1.98	2
14020511	2.05	2	2.13	2	1.82	2	2.00	2
14020512	3.80	4	3.69	4	4.99	5	4.16	4
14020514	2.22	2	2.07	2	1.86	2	2.05	2

Sample ID	Copy Number							
	r = 1		r = 2		r = 3		Average	
	Ratio (target/ref)	Estimated Copy Number	Ratio (target/ref)	Estimated Copy Number	Ratio (target/ref)	Estimated Copy Number	Ratio (target/ref)	Estimated Copy Number
14020515	1.16	1	1.15	1	1.52	2	1.28	1
14020516	2.19	2	2.33	2	3.04	3	2.52	3
14020517	1.81	2	1.94	2	1.54	2	1.76	2
14020518	2.54	3	2.81	3	2.59	3	2.65	3
14020522	1.22	1	1.15	1	1.16	1	1.18	1
14020523	1.03	1	1.00	1	1.12	1	1.05	1
14020524	1.10	1	1.24	1	1.13	1	1.16	1
14020525	0.95	1	1.11	1	1.09	1	1.05	1
14020528	1.97	2	2.30	2	2.25	2	2.17	2
14020537	0.98	1	1.14	1	1.02	1	1.05	1
14020538	1.85	2	2.17	2	1.74	2	1.92	2
14020541	2.86	3	2.54	3	2.65	3	2.68	3
14020545	3.86	4	4.28	4	3.93	4	4.02	4
14020546	1.04	1	1.04	1	1.32	1	1.13	1
14020547	6.92	7	6.71	7	7.88	8	7.17	7
14020548	1.82	2	2.47	2	1.77	2	2.02	2
14020551	1.05	1	1.16	1	1.08	1	1.10	1
14020552	2.15	2	1.93	2	1.89	2	1.99	2
14020553	2.47	2	2.51	3	2.65	3	2.54	3

Sample ID	Copy Number							
	r = 1		r = 2		r = 3		Average	
	Ratio (target/ref)	Estimated Copy Number	Ratio (target/ref)	Estimated Copy Number	Ratio (target/ref)	Estimated Copy Number	Ratio (target/ref)	Estimated Copy Number
14020554	4.82	5	4.59	5	4.55	5	4.65	5
14020560	1.97	2	2.03	2	1.99	2	2.00	2
14020566	5.92	6	5.36	5	4.80	5	5.36	5
14020567	2.14	2	2.00	2	2.02	2	2.06	2
14020573	0.88	1	0.88	1	1.03	1	0.93	1
14020575	1.14	1	1.12	1	1.13	1	1.13	1
14020584	1.34	1	1.13	1	1.28	1	1.25	1
14020585	1.28	1	1.06	1	1.25	1	1.20	1
14020590	1.37	1	1.49	1	1.49	1	1.45	1
14020597	1.22	1	1.25	1	1.25	1	1.24	1
14020598	1.15	1	1.19	1	1.15	1	1.16	1
14020601	3.72	4	3.50	4	3.62	4	3.61	4
14020603	1.07	1	1.04	1	1.00	1	1.04	1
14020604	1.09	1	1.08	1	1.12	1	1.10	1
14020606	1.23	1	1.21	1	1.35	1	1.26	1
14020607	1.17	1	1.45	1	1.39	1	1.33	1
14020608	1.36	1	1.40	1	1.15	1	1.30	1
14020609	1.24	1	1.21	1	1.13	1	1.19	1
14020610	2.09	2	2.02	2	2.08	2	2.07	2

Sample ID	Copy Number							
	r = 1		r = 2		r = 3		Average	
	Ratio (target/ref)	Estimated Copy Number	Ratio (target/ref)	Estimated Copy Number	Ratio (target/ref)	Estimated Copy Number	Ratio (target/ref)	Estimated Copy Number
14020611	1.18	1	1.19	1	1.02	1	1.13	1
14020612	1.32	1	1.26	1	1.25	1	1.27	1
14020616	1.16	1	1.28	1	1.14	1	1.19	1
14020619	1.16	1	1.16	1	1.14	1	1.16	1
14020621	2.32	2	2.20	2	2.18	2	2.23	2
14020624	2.31	2	2.10	2	1.93	2	2.11	2
14020628	1.22	1	1.31	1	1.16	1	1.23	1
14020630	1.18	1	1.18	1	1.29	1	1.22	1
14020632	11.31	11	12.00	12	13.31	13	12.21	12
14020636	1.82	2	1.80	2	1.88	2	1.83	2
14020638	1.09	1	0.99	1	1.05	1	1.04	1
14020644	1.20	1	1.46	1	1.27	1	1.31	1
14020645	1.49	1	1.32	1	1.20	1	1.34	1
14020646	1.08	1	1.27	1	1.16	1	1.17	1
14020647	2.84	3	3.41	3	3.28	3	3.18	3
14020649	6.40	6	6.06	6	5.74	6	6.06	6
14020663	6.80	7	6.39	6	6.59	7	6.59	7
14020666	2.13	2	1.99	2	2.04	2	2.05	2
14020667	1.10	1	1.10	1	1.08	1	1.09	1

Sample ID	Copy Number							
	r = 1		r = 2		r = 3		Average	
	Ratio (target/ref)	Estimated Copy Number	Ratio (target/ref)	Estimated Copy Number	Ratio (target/ref)	Estimated Copy Number	Ratio (target/ref)	Estimated Copy Number
14020668	1.02	1	0.95	1	1.02	1	1.00	1
14020672	2.88	3	2.58	3	2.65	3	2.70	3
14020675	1.22	1	1.18	1	1.18	1	1.19	1
14020678	1.10	1	1.04	1	1.05	1	1.06	1
14020679	1.00	1	1.05	1	1.10	1	1.05	1
14020682	7.27	7	7.00	7	7.57	8	7.28	7
14020683	1.05	1	1.15	1	1.10	1	1.10	1
14020684	2.04	2	2.46	2	2.44	2	2.31	2
14020685	2.20	2	2.10	2	2.46	2	2.25	2
14020686	1.00	1	0.90	1	0.99	1	0.96	1
14020687	1.18	1	1.21	1	1.38	1	1.26	1
14020692	3.25	3	3.30	3	3.45	3	3.33	3
14020694	1.00	1	0.96	1	1.00	1	0.98	1
14020695	1.11	1	1.04	1	0.98	1	1.04	1
14020698	2.04	2	2.09	2	2.15	2	2.09	2
14020700	1.07	1	1.20	1	1.00	1	1.09	1
14020701	1.16	1	1.37	1	1.04	1	1.19	1
14020703	3.56	4	3.60	4	3.57	4	3.58	4
14020704	1.25	1	1.16	1	1.25	1	1.22	1

Sample ID	Copy Number							
	r = 1		r = 2		r = 3		Average	
	Ratio (target/ref)	Estimated Copy Number	Ratio (target/ref)	Estimated Copy Number	Ratio (target/ref)	Estimated Copy Number	Ratio (target/ref)	Estimated Copy Number
14020706	3.70	4	4.04	4	3.41	3	3.72	4
14020711	2.99	3	2.83	3	2.44	2	2.75	3
14020719	1.58	2	1.63	2	1.63	2	1.61	2
14020720	1.86	2	2.35	2	2.15	2	2.12	2
14020722	1.11	1	1.14	1	1.14	1	1.13	1
14020724	1.42	1	1.30	1	1.42	1	1.38	1
14020727	1.20	1	1.24	1	1.21	1	1.22	1
14020732	1.33	1	1.27	1	1.31	1	1.31	1
14020740	1.17	1	1.13	1	1.25	1	1.18	1
14020743	1.39	1	1.33	1	1.36	1	1.36	1
14020745	0.78	1	0.66	1	0.66	1	0.70	1
14020746	3.74	4	3.74	4	3.55	4	3.68	4
14020751	2.08	2	2.06	2	2.06	2	2.07	2
14020754	1.04	1	1.10	1	1.14	1	1.09	1
14020755	1.21	1	1.34	1	1.31	1	1.29	1
14020756	1.28	1	1.45	1	1.35	1	1.36	1
14020757	1.13	1	1.19	1	1.28	1	1.20	1
14020758	1.31	1	1.13	1	1.14	1	1.19	1
14020771	1.18	1	1.17	1	1.07	1	1.14	1

Sample ID	Copy Number							
	r = 1		r = 2		r = 3		Average	
	Ratio (target/ref)	Estimated Copy Number	Ratio (target/ref)	Estimated Copy Number	Ratio (target/ref)	Estimated Copy Number	Ratio (target/ref)	Estimated Copy Number
14020772	2.60	3	2.67	3	2.82	3	2.70	3
14020774	1.34	1	1.13	1	1.12	1	1.20	1
14020776	2.19	2	2.19	2	2.20	2	2.19	2
14020778	6.12	6	7.75	8	6.56	7	6.81	7
14020780	0.13	0	0.14	0	0.15	0	0.14	0
14020782	1.08	1	1.38	1	1.64	2	1.37	1
14020784	3.31	3	3.38	3	3.28	3	3.32	3
14020785	2.41	2	2.85	3	2.61	3	2.63	3
14020787	1.33	1	1.24	1	1.62	2	1.40	1
14020800	3.95	4	4.22	4	3.77	4	3.98	4
14020801	2.63	3	2.52	3	2.75	3	2.63	3
14020802	1.44	1	1.39	1	1.35	1	1.39	1
14020805	1.90	2	1.87	2	1.94	2	1.90	2
14020812	1.00	1	1.28	1	1.10	1	1.13	1
14020814	1.29	1	1.09	1	1.29	1	1.22	1
14020815	1.19	1	1.04	1	1.20	1	1.14	1
14020816	1.60	2	1.71	2	1.64	2	1.65	2
14020817	1.30	1	1.43	1	1.55	2	1.43	1
14020821	1.31	1	1.39	1	1.41	1	1.37	1

Sample ID	Copy Number							
	r = 1		r = 2		r = 3		Average	
	Ratio (target/ref)	Estimated Copy Number	Ratio (target/ref)	Estimated Copy Number	Ratio (target/ref)	Estimated Copy Number	Ratio (target/ref)	Average Copy Number
14020823	0.90	1	0.83	1	0.82	1	0.85	1
14020824	3.10	3	2.92	3	3.02	3	3.01	3
14020828	2.08	2	2.39	2	2.35	2	2.27	2
14020831	1.23	1	1.14	1	1.36	1	1.24	1
14020834	1.43	1	1.64	2	1.41	1	1.49	1
14020836	1.69	2	1.69	2	1.66	2	1.68	2
14020837	1.51	2	1.56	2	1.38	1	1.48	1
14020838	1.31	1	1.27	1	1.32	1	1.30	1
14020839	1.22	1	1.26	1	1.17	1	1.22	1
14020844	2.71	3	2.72	3	2.66	3	2.70	3
14020845	1.30	1	1.16	1	1.23	1	1.23	1
14020846	2.38	2	2.14	2	2.41	2	2.31	2
14020849	5.49	5	4.84	5	5.81	6	5.38	5
14020850	1.32	1	1.22	1	1.85	2	1.46	1
14020851	3.70	4	3.44	3	3.58	4	3.57	4
14020853	2.19	2	2.14	2	2.45	2	2.26	2
14020854	1.64	2	1.87	2	1.79	2	1.77	2
14020859	1.92	2	2.32	2	2.26	2	2.17	2
14020862	1.54	2	1.28	1	1.38	1	1.40	1

Sample ID	Copy Number							
	r = 1		r = 2		r = 3		Average	
	Ratio (target/ref)	Estimated Copy Number	Ratio (target/ref)	Estimated Copy Number	Ratio (target/ref)	Estimated Copy Number	Ratio (target/ref)	Estimated Copy Number
14020865	1.39	1	1.38	1	1.43	1	1.40	1
14020866	3.11	3	3.14	3	3.00	3	3.08	3
14020868	1.43	1	1.56	2	1.80	2	1.60	2
14020869	1.05	1	1.00	1	1.08	1	1.04	1
14020873	1.32	1	2.16	2	2.42	2	1.96	2
14020875	3.36	3	3.68	4	3.27	3	3.44	3
14020876	2.78	3	2.84	3	2.93	3	2.85	3
14020878	1.37	1	1.42	1	1.32	1	1.37	1
14020887	0.10	0	0.13	0	0.10	0	0.11	0
14020891	3.15	3	3.21	3	3.50	4	3.29	3
14020892	1.53	2	1.75	2	1.82	2	1.70	2
14020893	2.31	2	1.98	2	2.17	2	2.15	2
14020894	4.31	4	4.68	5	4.75	5	4.58	5
14020896	3.98	4	4.00	4	4.39	4	4.12	4
14020898	1.49	1	1.46	1	1.21	1	1.39	1
14020899	4.81	5	5.18	5	5.13	5	5.04	5
14020900	4.20	4	4.29	4	4.84	5	4.44	4
14020903	2.85	3	2.78	3	2.61	3	2.75	3
14020904	1.59	2	1.51	2	1.36	1	1.48	1

Sample ID	Copy Number							
	r = 1		r = 2		r = 3		Average	
	Ratio (target/ref)	Estimated Copy Number	Ratio (target/ref)	Estimated Copy Number	Ratio (target/ref)	Estimated Copy Number	Ratio (target/ref)	Estimated Copy Number
14020905	1.34	1	2.10	2	1.54	2	1.66	2
14020906	1.57	2	1.51	2	1.48	1	1.52	2
14020908	2.00	2	1.81	2	1.93	2	1.92	2
14020917	3.35	3	3.14	3	3.20	3	3.23	3
14020925	0.90	1	1.18	1	1.06	1	1.05	1
14020929	3.97	4	3.93	4	3.50	4	3.80	4
14020934	2.74	3	2.66	3	2.60	3	2.66	3
14020935	1.39	1	1.26	1	1.33	1	1.33	1
14020940	0.78	1	0.78	1	0.76	1	0.78	1
14020942	3.00	3	3.00	3	3.50	3	3.16	3
14020945	1.93	2	2.03	2	1.67	2	1.88	2
14020958	0.93	1	1.11	1	1.05	1	1.03	1
14020961	3.14	3	2.76	3	3.00	3	2.97	3
14020968	3.28	3	2.90	3	3.18	3	3.12	3
14020970	4.07	4	4.30	4	3.85	4	4.07	4
14020972	0.99	1	0.99	1	0.94	1	0.97	1
14020975	0.81	1	0.80	1	0.91	1	0.84	1
14020977	5.11	5	4.63	5	4.75	5	4.83	5
14020981	1.56	2	1.64	2	1.65	2	1.62	2

Sample ID	Copy Number							
	r = 1		r = 2		r = 3		Average	
	Ratio (target/ref)	Estimated Copy Number	Ratio (target/ref)	Estimated Copy Number	Ratio (target/ref)	Estimated Copy Number	Ratio (target/ref)	Estimated Copy Number
14020983	0.84	1	0.83	1	0.83	1	0.83	1
14020984	1.25	1	0.73	1	0.92	1	0.97	1
14020987	3.03	3	2.77	3	3.70	4	3.17	3
14020994	0.98	1	0.92	1	1.01	1	0.97	1
14020996	2.30	2	1.85	2	1.85	2	2.00	2
14021002	0.49	0	0.47	0	0.52	1	0.49	0
14021008	1.12	1	1.25	1	1.31	1	1.23	1
14021011	4.04	4	3.64	4	3.82	4	3.84	4
14021014	4.04	4	3.92	4	3.84	4	3.93	4
14021015	2.54	3	3.22	3	2.90	3	2.89	3
14021016	3.88	4	4.08	4	4.09	4	4.02	4
14021017	3.47	3	4.07	4	4.03	4	3.86	4
14021018	3.07	3	3.17	3	2.98	3	3.07	3
14021022	2.41	2	2.11	2	2.17	2	2.23	2
14021023	2.18	2	2.02	2	2.13	2	2.11	2
14021026	9.04	9	7.83	8	7.55	8	8.14	8
14021028	0.96	1	0.99	1	1.06	1	1.00	1
14021030	0.79	1	0.73	1	0.79	1	0.77	1
14021032	0.98	1	1.03	1	1.15	1	1.05	1

Sample ID	Copy Number							
	r = 1		r = 2		r = 3		Average	
	Ratio (target/ref)	Estimated Copy Number	Ratio (target/ref)	Estimated Copy Number	Ratio (target/ref)	Estimated Copy Number	Ratio (target/ref)	Estimated Copy Number
14021033	4.31	4	3.40	3	3.74	4	3.82	4
14021034	4.00	4	3.96	4	3.61	4	3.86	4
14021035	4.89	5	4.85	5	4.38	4	4.71	5
14021037	3.82	4	3.69	4	3.63	4	3.71	4
14021040	3.80	4	3.63	4	3.76	4	3.73	4
14021041	1.48	1	1.36	1	1.35	1	1.40	1
14021048	4.92	5	5.08	5	4.82	5	4.94	5
14021049	1.89	2	1.92	2	2.04	2	1.95	2
14021050	0.01	0	0.01	0	0.02	0	0.01	0
14021051	1.15	1	1.21	1	1.26	1	1.20	1
14021052	1.34	1	1.25	1	1.18	1	1.26	1
14021054	1.13	1	1.13	1	1.04	1	1.10	1
14021055	1.51	2	1.67	2	2.07	2	1.75	2
14021056	8.03	8	6.93	7	6.97	7	7.31	7
14021061	0.74	1	0.74	1	0.65	1	0.71	1
14021069	1.99	2	2.03	2	1.82	2	1.95	2
14021070	1.74	2	1.66	2	1.74	2	1.71	2
14021071	1.99	2	2.10	2	2.12	2	2.07	2
14021073	1.02	1	1.18	1	1.09	1	1.10	1

Sample ID	Copy Number							
	r = 1		r = 2		r = 3		Average	
	Ratio (target/ref)	Estimated Copy Number	Ratio (target/ref)	Estimated Copy Number	Ratio (target/ref)	Estimated Copy Number	Ratio (target/ref)	Estimated Copy Number
14021075	2.88	3	2.36	2	3.34	3	2.86	3
14021076	5.27	5	5.27	5	5.48	5	5.34	5
14021081	4.02	4	4.50	4	4.17	4	4.23	4
14021082	4.15	4	4.23	4	4.19	4	4.19	4
14021086	2.10	2	2.14	2	1.99	2	2.08	2
14021089	1.09	1	0.95	1	0.97	1	1.00	1
14021099	3.89	4	3.48	3	3.54	4	3.64	4
14021101	2.16	2	2.22	2	2.13	2	2.17	2
14021106	0.96	1	1.09	1	1.07	1	1.04	1
14021107	2.13	2	2.11	2	2.10	2	2.11	2
14021118	0.96	1	1.06	1	0.92	1	0.98	1
14021119	1.03	1	1.02	1	1.01	1	1.02	1
14021125	0.93	1	0.95	1	0.84	1	0.91	1
14021130	2.58	3	1.97	2	1.93	2	2.16	2
14021132	1.05	1	1.15	1	1.17	1	1.12	1
14021198	1.94	2	1.76	2	2.27	2	1.99	2
14021212	5.57	6	3.58	4	3.77	4	4.30	4
14021214	0.77	1	0.94	1	0.88	1	0.86	1
14021217	2.05	2	2.15	2	2.18	2	2.12	2

Sample ID	Copy Number							
	r = 1		r = 2		r = 3		Average	
	Ratio (target/ref)	Estimated Copy Number	Ratio (target/ref)	Estimated Copy Number	Ratio (target/ref)	Estimated Copy Number	Ratio (target/ref)	Estimated Copy Number
14021220	0.85	1	0.88	1	0.93	1	0.89	1
14021223	2.60	3	2.72	3	3.45	3	2.92	3
14021227	6.95	7	8.02	8	9.53	10	8.17	8
14021229	3.61	4	3.99	4	2.93	3	3.51	4
14021230	4.81	5	4.45	4	5.14	5	4.80	5
14021231	0.64	1	0.56	1	0.87	1	0.69	1
14021232	0.88	1	1.04	1	1.10	1	1.01	1
14021233	1.10	1	1.03	1	1.08	1	1.07	1
14021236	0.70	1	0.73	1	0.81	1	0.75	1
14021238	0.88	1	0.90	1	0.93	1	0.91	1
14021239	0.27	0	0.29	0	0.30	0	0.29	0
14021241	2.59	3	2.68	3	2.59	3	2.62	3
14021251	3.59	4	4.03	4	4.10	4	3.91	4
14021252	3.94	4	4.10	4	3.44	3	3.83	4
14021256	1.90	2	2.08	2	1.91	2	1.96	2
14021258	5.33	5	6.08	6	6.34	6	5.91	6
14021265	2.42	2	2.13	2	2.08	2	2.21	2
14021266	1.81	2	1.72	2	2.40	2	1.98	2
14021271	1.60	2	1.71	2	1.97	2	1.76	2

Sample ID	Copy Number							
	r = 1		r = 2		r = 3		Average	
	Ratio (target/ref)	Estimated Copy Number	Ratio (target/ref)	Estimated Copy Number	Ratio (target/ref)	Estimated Copy Number	Ratio (target/ref)	Estimated Copy Number
14021272	2.01	2	2.00	2	1.87	2	1.96	2
14021273	2.34	2	2.29	2	2.04	2	2.22	2
14021276	1.06	1	1.09	1	0.83	1	1.00	1
14021277	1.90	2	1.71	2	1.70	2	1.77	2
14021278	1.03	1	0.94	1	0.90	1	0.96	1
14021279	1.60	2	1.54	2	1.63	2	1.59	2
14021280	0.70	1	1.01	1	1.41	1	1.04	1
14021286	2.01	2	2.03	2	1.71	2	1.92	2
14021290	1.20	1	1.11	1	1.10	1	1.14	1
14021292	5.38	5	4.60	5	4.55	5	4.84	5
14021294	2.15	2	1.94	2	1.87	2	1.99	2
14021297	2.08	2	2.05	2	2.00	2	2.05	2
14021298	1.96	2	1.77	2	1.68	2	1.81	2
14021303	2.74	3	2.82	3	2.67	3	2.74	3
14021306	2.77	3	2.90	3	2.71	3	2.79	3
14021308	2.94	3	2.64	3	2.59	3	2.72	3
14021311	1.19	1	1.19	1	1.20	1	1.19	1
14021314	1.14	1	1.02	1	1.22	1	1.13	1
14021318	1.70	2	1.73	2	1.85	2	1.76	2

Sample ID	Copy Number							
	r = 1		r = 2		r = 3		Average	
	Ratio (target/ref)	Estimated Copy Number	Ratio (target/ref)	Estimated Copy Number	Ratio (target/ref)	Estimated Copy Number	Ratio (target/ref)	Estimated Copy Number
14021334	1.02	1	1.13	1	1.13	1	1.09	1
14021335	1.10	1	1.05	1	1.08	1	1.08	1
14021337	1.52	2	1.53	2	1.49	1	1.51	2
14021338	1.07	1	1.16	1	1.20	1	1.14	1
14021341	0.99	1	0.89	1	1.08	1	0.99	1
14021342	0.57	1	0.51	1	0.50	0	0.53	1
14021352	1.08	1	1.09	1	0.98	1	1.05	1
14021353	1.04	1	1.09	1	0.93	1	1.02	1
14021355	0.76	1	0.75	1	0.75	1	0.76	1
14021356	4.08	4	3.36	3	3.71	4	3.72	4
14021360	2.22	2	2.19	2	1.95	2	2.12	2
14021362	4.98	5	5.13	5	5.68	6	5.26	5
14021363	5.34	5	4.97	5	5.41	5	5.24	5
14021369	2.59	3	3.10	3	2.55	3	2.75	3
14021370	1.89	2	1.43	1	1.95	2	1.76	2
14021372	0.95	1	0.85	1	1.01	1	0.94	1
14021375	1.74	2	1.63	2	1.68	2	1.68	2
14021376	4.00	4	3.40	3	3.07	3	3.49	3
14021379	4.08	4	5.68	6	4.82	5	4.86	5

Sample ID	Copy Number							
	r = 1		r = 2		r = 3		Average	
	Ratio (target/ref)	Estimated Copy Number	Ratio (target/ref)	Estimated Copy Number	Ratio (target/ref)	Estimated Copy Number	Ratio (target/ref)	Estimated Copy Number
14021381	1.14	1	0.98	1	1.05	1	1.06	1
14021383	1.00	1	1.06	1	1.19	1	1.09	1
14021385	1.18	1	1.13	1	0.91	1	1.07	1
14021387	12.73	13	9.29	9	8.12	8	10.05	10
14021388	2.28	2	2.33	2	2.22	2	2.28	2
14021389	1.22	1	1.10	1	1.20	1	1.17	1
14021392	1.94	2	2.07	2	2.08	2	2.03	2
14021394	2.17	2	2.05	2	2.08	2	2.10	2
14021399	3.04	3	3.01	3	2.96	3	3.00	3
14021408	1.09	1	1.06	1	1.17	1	1.11	1
14021411	2.19	2	2.00	2	2.09	2	2.09	2
14021412	1.67	2	1.78	2	1.78	2	1.74	2
14021424	5.27	5	5.25	5	5.72	6	5.41	5
14021425	5.83	6	5.31	5	4.54	5	5.22	5
14021428	2.82	3	2.55	3	3.20	3	2.86	3
14021429	2.04	2	2.05	2	1.92	2	2.00	2
14021431	1.89	2	1.64	2	1.65	2	1.73	2
14021432	2.14	2	2.21	2	2.07	2	2.14	2
14021437	3.73	4	3.36	3	3.47	3	3.52	4

Sample ID	Copy Number							
	r = 1		r = 2		r = 3		Average	
	Ratio (target/ref)	Estimated Copy Number	Ratio (target/ref)	Estimated Copy Number	Ratio (target/ref)	Estimated Copy Number	Ratio (target/ref)	Estimated Copy Number
14021438	5.09	5	6.60	7	4.27	4	5.32	5
14021439	4.56	5	4.51	5	4.51	5	4.53	5
14021441	1.05	1	1.01	1	1.21	1	1.09	1
14021443	3.59	4	3.12	3	3.25	3	3.32	3
14021444	2.32	2	2.38	2	2.20	2	2.30	2
14021447	2.71	3	2.92	3	2.52	3	2.72	3
14021450	1.22	1	0.97	1	0.91	1	1.03	1
14021451	0.82	1	0.72	1	1.25	1	0.93	1
14021453	0.80	1	0.87	1	0.91	1	0.86	1
14021455	2.14	2	2.09	2	1.86	2	2.03	2
14021458	1.08	1	0.98	1	0.86	1	0.97	1
14021463	1.34	1	1.02	1	1.03	1	1.13	1
14021467	0.95	1	0.93	1	0.96	1	0.95	1
14021468	1.48	1	1.21	1	1.34	1	1.34	1
14021469	1.03	1	1.13	1	1.08	1	1.08	1
14021470	2.03	2	2.21	2	1.98	2	2.07	2
14021471	5.08	5	4.86	5	5.10	5	5.01	5
14021473	1.91	2	1.70	2	1.70	2	1.77	2
14021474	2.93	3	3.09	3	2.82	3	2.95	3

Sample ID	Copy Number							
	r = 1		r = 2		r = 3		Average	
	Ratio (target/ref)	Estimated Copy Number	Ratio (target/ref)	Estimated Copy Number	Ratio (target/ref)	Estimated Copy Number	Ratio (target/ref)	Estimated Copy Number
14021476	1.10	1	1.36	1	1.46	1	1.31	1
14021478	1.00	1	1.00	1	1.31	1	1.10	1
14021479	2.87	3	2.20	2	2.20	2	2.42	2
14021483	1.23	1	1.24	1	1.23	1	1.23	1
14021484	2.59	3	2.81	3	2.82	3	2.74	3
14021485	2.10	2	2.02	2	1.99	2	2.04	2
14021492	1.85	2	1.84	2	1.82	2	1.83	2
14021493	1.86	2	1.73	2	1.85	2	1.81	2
14021496	2.55	3	2.73	3	3.58	4	2.95	3
14021497	2.89	3	2.99	3	2.70	3	2.86	3
14021498	8.02	8	8.83	9	7.05	7	7.97	8
14021500	2.04	2	2.19	2	1.85	2	2.03	2
14021501	7.96	8	7.34	7	6.50	6	7.27	7
14021502	8.51	9	7.02	7	7.82	8	7.78	8
14021506	1.83	2	1.81	2	1.86	2	1.83	2
14021509	1.89	2	1.97	2	1.76	2	1.87	2
14021511	1.17	1	1.15	1	1.02	1	1.11	1
14021514	0.86	1	0.85	1	0.90	1	0.87	1
14021517	0.97	1	1.02	1	1.09	1	1.03	1

Sample ID	Copy Number							
	r = 1		r = 2		r = 3		Average	
	Ratio (target/ref)	Estimated Copy Number	Ratio (target/ref)	Estimated Copy Number	Ratio (target/ref)	Estimated Copy Number	Ratio (target/ref)	Estimated Copy Number
14021519	3.67	4	3.76	4	3.51	4	3.65	4
14021520	0.96	1	0.86	1	0.96	1	0.93	1
14021524	1.06	1	1.06	1	1.16	1	1.09	1
14021526	1.20	1	1.14	1	1.08	1	1.14	1
14021532	1.08	1	1.17	1	1.08	1	1.11	1
14021533	1.09	1	1.13	1	1.14	1	1.12	1
14021535	2.02	2	2.02	2	2.03	2	2.02	2
14021536	2.43	2	2.63	3	2.87	3	2.64	3
14021539	1.94	2	2.10	2	2.19	2	2.08	2
14021542	0.92	1	0.91	1	0.80	1	0.88	1
14021544	0.42	0	0.45	0	0.52	1	0.46	0
14021545	1.12	1	1.09	1	1.20	1	1.13	1
14021550	1.14	1	0.96	1	1.02	1	1.04	1
14021554	1.16	1	1.20	1	1.16	1	1.17	1
14021555	0.93	1	1.18	1	1.14	1	1.08	1
14021557	1.85	2	1.83	2	1.89	2	1.85	2
14021558	1.06	1	1.06	1	1.12	1	1.08	1
14021559	0.66	1	0.56	1	0.65	1	0.62	1
14021564	2.95	3	2.90	3	2.89	3	2.91	3

Sample ID	Copy Number							
	r = 1		r = 2		r = 3		Average	
	Ratio (target/ref)	Estimated Copy Number	Ratio (target/ref)	Estimated Copy Number	Ratio (target/ref)	Estimated Copy Number	Ratio (target/ref)	Estimated Copy Number
14021566	4.37	4	4.65	5	5.14	5	4.72	5
14021572	0.83	1	0.93	1	0.95	1	0.91	1
14021574	1.74	2	1.95	2	2.50	2	2.07	2
14021583	1.12	1	1.11	1	1.03	1	1.09	1
14021586	1.06	1	1.32	1	1.00	1	1.13	1
14021589	1.27	1	1.33	1	1.31	1	1.30	1
14021592	1.09	1	1.16	1	1.05	1	1.10	1
14021593	4.10	4	3.98	4	3.55	4	3.87	4
14021597	1.16	1	1.09	1	1.12	1	1.12	1
14021598	0.31	0	0.31	0	0.32	0	0.31	0
14021601	4.59	5	4.64	5	4.23	4	4.49	4
14021602	5.16	5	5.68	6	4.67	5	5.17	5
14021608	3.39	3	3.91	4	3.34	3	3.55	4
14021609	1.95	2	1.94	2	1.77	2	1.89	2
14021611	3.10	3	3.12	3	3.21	3	3.14	3
14021620	2.51	3	2.40	2	2.41	2	2.44	2
14021621	3.16	3	2.76	3	2.61	3	2.84	3
14021626	1.15	1	1.28	1	1.33	1	1.25	1
14021628	1.18	1	1.35	1	1.38	1	1.30	1

Sample ID	Copy Number							
	r = 1		r = 2		r = 3		Average	
	Ratio (target/ref)	Estimated Copy Number	Ratio (target/ref)	Estimated Copy Number	Ratio (target/ref)	Estimated Copy Number	Ratio (target/ref)	Estimated Copy Number
14021629	1.41	1	1.38	1	1.43	1	1.40	1
14021634	1.39	1	1.55	2	1.51	2	1.48	1
14021635	1.39	1	1.33	1	1.18	1	1.30	1
14021636	1.45	1	1.35	1	1.25	1	1.35	1
14021639	1.19	1	1.08	1	1.10	1	1.12	1
14021641	1.10	1	1.12	1	1.02	1	1.08	1
14021642	1.25	1	1.15	1	1.16	1	1.19	1
14021644	0.69	1	0.62	1	0.55	1	0.62	1
14021646	3.52	4	3.49	3	3.40	3	3.47	3
14021647	3.60	4	3.54	4	3.93	4	3.69	4
14021648	3.44	3	3.33	3	3.45	3	3.41	3
14021649	2.10	2	1.87	2	2.14	2	2.04	2
14021650	2.55	3	2.54	3	2.58	3	2.56	3
14021651	1.17	1	1.35	1	1.13	1	1.22	1
14021652	0.91	1	0.77	1	1.04	1	0.91	1
14021653	1.33	1	1.13	1	1.26	1	1.24	1
14021654	1.16	1	1.42	1	1.32	1	1.30	1
14021655	0.36	0	0.38	0	0.37	0	0.37	0
14021657	0.89	1	1.00	1	0.93	1	0.94	1

Sample ID	Copy Number							
	r = 1		r = 2		r = 3		Average	
	Ratio (target/ref)	Estimated Copy Number	Ratio (target/ref)	Estimated Copy Number	Ratio (target/ref)	Estimated Copy Number	Ratio (target/ref)	Estimated Copy Number
14021661	5.77	6	5.36	5	4.86	5	5.33	5
14021662	4.08	4	3.96	4	3.80	4	3.95	4
14021663	5.21	5	3.96	4	4.17	4	4.44	4
14021664	4.08	4	4.11	4	4.14	4	4.11	4
14021671	1.29	1	1.08	1	1.09	1	1.15	1
14021672	6.64	7	6.87	7	6.79	7	6.77	7
14021679	4.50	4	4.42	4	4.15	4	4.35	4
14021680	4.24	4	4.44	4	4.22	4	4.30	4
14021681	2.04	2	2.39	2	2.30	2	2.24	2
14021682	1.95	2	1.94	2	2.48	2	2.12	2
14021685	1.10	1	0.93	1	1.50	2	1.18	1
14021686	1.00	1	0.95	1	1.08	1	1.01	1
14021687	1.10	1	1.08	1	1.20	1	1.12	1
14021689	5.62	6	5.64	6	6.08	6	5.78	6
14021690	1.19	1	1.17	1	1.11	1	1.16	1
14021692	3.43	3	2.90	3	3.14	3	3.16	3
14021693	2.63	3	2.81	3	2.61	3	2.68	3
14021694	2.90	3	3.45	3	3.28	3	3.21	3
14021695	1.87	2	1.67	2	2.03	2	1.86	2

Sample ID	Copy Number							
	r = 1		r = 2		r = 3		Average	
	Ratio (target/ref)	Estimated Copy Number	Ratio (target/ref)	Estimated Copy Number	Ratio (target/ref)	Estimated Copy Number	Ratio (target/ref)	Estimated Copy Number
14021698	3.61	4	3.73	4	3.80	4	3.71	4
14021703	2.08	2	2.26	2	2.29	2	2.21	2
14021704	0.88	1	0.60	1	0.63	1	0.70	1
14021705	7.04	7	7.35	7	8.08	8	7.49	7
14021706	2.13	2	1.63	2	2.19	2	1.98	2
14021710	12.11	12	12.94	13	11.63	12	12.23	12
14021711	1.16	1	1.32	1	1.21	1	1.23	1
14021713	4.35	4	3.25	3	3.83	4	3.81	4
14021714	6.36	6	4.97	5	5.99	6	5.78	6
14021715	2.28	2	2.04	2	1.90	2	2.07	2
14021718	4.41	4	4.42	4	4.49	4	4.44	4
14021720	1.29	1	1.41	1	1.43	1	1.38	1
14021721	2.34	2	2.51	3	2.84	3	2.56	3
14021722	0.94	1	0.93	1	1.00	1	0.96	1
14021725	3.24	3	4.19	4	4.19	4	3.87	4
14021728	1.17	1	1.20	1	1.32	1	1.23	1
14021731	1.14	1	1.30	1	1.32	1	1.25	1
14021732	1.33	1	1.40	1	1.57	2	1.43	1
14021733	0.77	1	0.69	1	0.74	1	0.73	1

Sample ID	Copy Number							
	r = 1		r = 2		r = 3		Average	
	Ratio (target/ref)	Estimated Copy Number	Ratio (target/ref)	Estimated Copy Number	Ratio (target/ref)	Estimated Copy Number	Ratio (target/ref)	Estimated Copy Number
14021735	2.02	2	2.03	2	1.96	2	2.00	2
14021736	1.98	2	2.03	2	1.83	2	1.94	2
14021739	1.02	1	0.99	1	0.97	1	0.99	1
14021741	2.91	3	2.71	3	2.64	3	2.75	3
14021744	0.42	0	0.36	0	0.37	0	0.38	0
14021745	2.68	3	4.25	4	4.24	4	3.72	4
14021746	1.55	2	1.48	1	1.07	1	1.37	1
14021747	1.57	2	1.35	1	1.43	1	1.45	1
14021748	2.01	2	1.90	2	1.95	2	1.95	2
14021750	0.54	1	0.52	1	0.53	1	0.53	1
14021751	1.04	1	1.00	1	1.10	1	1.04	1
14021753	3.25	3	3.36	3	3.58	4	3.40	3
14021757	3.29	3	1.95	2	3.21	3	2.82	3
14021759	4.41	4	7.42	7	4.19	4	5.34	5
14021761	2.25	2	2.03	2	1.95	2	2.07	2
14021762	2.18	2	2.24	2	1.90	2	2.11	2
14021765	1.87	2	1.97	2	1.86	2	1.90	2
14021766	3.85	4	3.23	3	3.11	3	3.40	3
14021767	3.65	4	4.02	4	3.79	4	3.82	4

Sample ID	Copy Number							
	r = 1		r = 2		r = 3		Average	
	Ratio (target/ref)	Estimated Copy Number	Ratio (target/ref)	Estimated Copy Number	Ratio (target/ref)	Estimated Copy Number	Ratio (target/ref)	Estimated Copy Number
14021773	2.92	3	2.64	3	3.05	3	2.87	3
14021775	1.29	1	1.35	1	1.41	1	1.35	1
14021777	2.57	3	2.76	3	2.67	3	2.67	3
14021778	1.08	1	1.30	1	1.37	1	1.25	1
14021781	1.21	1	1.59	2	1.68	2	1.49	1
14021783	1.23	1	1.17	1	1.34	1	1.25	1
14021784	2.04	2	1.96	2	2.03	2	2.01	2
14021785	3.13	3	3.31	3	3.03	3	3.16	3
14021791	1.76	2	1.88	2	2.30	2	1.98	2
14021792	2.07	2	2.06	2	1.96	2	2.03	2
14021795	4.37	4	3.83	4	4.40	4	4.20	4
14021796	2.97	3	3.14	3	4.10	4	3.40	3
14021798	2.19	2	2.35	2	2.02	2	2.19	2
14021799	0.53	1	0.63	1	0.85	1	0.67	1
14021800	1.75	2	1.54	2	1.66	2	1.65	2
14030005	2.45	2	2.27	2	2.16	2	2.29	2
14030017	1.97	2	2.73	3	2.03	2	2.24	2
14030022	1.24	1	1.11	1	1.18	1	1.18	1
14030023	3.59	4	3.00	3	3.28	3	3.29	3

Sample ID	Copy Number							
	r = 1		r = 2		r = 3		Average	
	Ratio (target/ref)	Estimated Copy Number	Ratio (target/ref)	Estimated Copy Number	Ratio (target/ref)	Estimated Copy Number	Ratio (target/ref)	Estimated Copy Number
14030025	3.61	4	3.05	3	3.29	3	3.32	3
14030035	4.86	5	5.14	5	5.07	5	5.03	5
14030038	2.75	3	3.08	3	3.29	3	3.04	3
14030039	2.37	2	2.13	2	2.10	2	2.20	2
14030040	2.12	2	2.38	2	2.41	2	2.30	2
14030044	2.07	2	2.71	3	2.90	3	2.56	3
14030045	2.42	2	2.51	3	2.77	3	2.57	3
14030046	1.41	1	1.65	2	1.54	2	1.54	2
14030047	1.45	1	1.59	2	1.53	2	1.52	2
14030048	2.17	2	2.06	2	2.11	2	2.11	2
14030049	7.86	8	8.80	9	9.41	9	8.69	9
14030053	4.96	5	5.74	6	6.35	6	5.68	6
14030054	5.41	5	6.11	6	6.37	6	5.96	6
14030057	2.19	2	1.96	2	1.98	2	2.04	2
14030061	5.46	5	4.63	5	4.34	4	4.81	5
14030062	5.50	6	4.82	5	4.41	4	4.91	5
14030063	3.14	3	2.82	3	2.34	2	2.77	3
14030064	8.79	9	8.88	9	9.02	9	8.90	9
14030065	4.54	5	4.65	5	4.94	5	4.71	5

Sample ID	Copy Number							
	r = 1		r = 2		r = 3		Average	
	Ratio (target/ref)	Estimated Copy Number	Ratio (target/ref)	Estimated Copy Number	Ratio (target/ref)	Estimated Copy Number	Ratio (target/ref)	Estimated Copy Number
14030069	2.01	2	1.54	2	1.94	2	1.83	2
14030070	1.52	2	1.36	1	1.40	1	1.42	1
14030072	4.49	4	4.15	4	4.00	4	4.21	4
14030073	3.40	3	3.06	3	2.98	3	3.15	3
14030074	4.24	4	3.96	4	3.91	4	4.04	4
14030076	2.56	3	2.61	3	2.40	2	2.52	3
14030083	5.31	5	4.86	5	5.23	5	5.13	5
14030084	20.88	21	17.64	18	17.47	17	18.66	19
14030087	0.75	1	0.78	1	0.71	1	0.74	1
14030088	2.67	3	2.33	2	2.81	3	2.60	3
14030089	2.01	2	2.14	2	2.30	2	2.15	2
14030090	3.04	3	3.34	3	3.28	3	3.22	3
14030093	7.75	8	7.17	7	6.98	7	7.30	7
14030096	2.09	2	2.28	2	2.50	2	2.29	2
14030098	0.96	1	1.04	1	1.08	1	1.03	1
14030103	4.04	4	3.96	4	4.43	4	4.14	4
14030106	0.74	1	0.83	1	0.80	1	0.79	1
14030111	2.70	3	2.48	2	2.40	2	2.53	3
14030114	5.25	5	5.34	5	4.76	5	5.12	5

Sample ID	Copy Number							
	r = 1		r = 2		r = 3		Average	
	Ratio (target/ref)	Estimated Copy Number	Ratio (target/ref)	Estimated Copy Number	Ratio (target/ref)	Estimated Copy Number	Ratio (target/ref)	Estimated Copy Number
14030115	1.17	1	1.14	1	1.11	1	1.14	1
14030117	0.98	1	0.92	1	0.80	1	0.90	1
14030120	2.16	2	1.90	2	1.85	2	1.97	2
14030122	2.78	3	2.77	3	2.86	3	2.80	3
14030123	3.55	4	3.31	3	3.03	3	3.30	3
14030129	5.23	5	4.99	5	4.28	4	4.83	5
14030132	2.23	2	2.18	2	2.34	2	2.25	2
14030133	1.10	1	0.97	1	0.97	1	1.01	1
14030134	2.80	3	2.62	3	2.93	3	2.78	3
14030149	2.21	2	2.17	2	2.12	2	2.17	2
14030150	2.38	2	2.37	2	2.38	2	2.37	2
14030154	2.69	3	2.84	3	3.31	3	2.95	3
14030158	1.51	2	1.55	2	1.49	1	1.52	2
14030159	3.05	3	2.21	2	2.69	3	2.65	3
14030160	2.65	3	2.37	2	2.76	3	2.59	3
14030163	3.66	4	3.58	4	4.03	4	3.75	4
14030165	1.18	1	1.24	1	1.11	1	1.18	1
14030169	1.44	1	1.28	1	1.14	1	1.29	1
14030170	1.67	2	1.52	2	1.48	1	1.56	2

Sample ID	Copy Number							
	r = 1		r = 2		r = 3		Average	
	Ratio (target/ref)	Estimated Copy Number	Ratio (target/ref)	Estimated Copy Number	Ratio (target/ref)	Estimated Copy Number	Ratio (target/ref)	Estimated Copy Number
14030171	13.35	13	12.31	12	11.48	11	12.38	12
14030181	12.75	13	7.77	8	7.11	7	9.21	9
14030185	1.61	2	1.73	2	1.81	2	1.72	2
14030187	1.75	2	1.61	2	1.78	2	1.71	2
14030188	0.98	1	1.28	1	0.94	1	1.07	1
14030194	2.85	3	2.84	3	3.24	3	2.98	3
14030198	1.38	1	1.69	2	1.52	2	1.53	2
14030200	1.61	2	1.88	2	2.32	2	1.93	2
14030201	1.69	2	1.87	2	2.00	2	1.85	2
14030204	5.67	6	6.58	7	6.85	7	6.37	6
14030206	1.75	2	1.75	2	1.91	2	1.80	2
14030208	1.93	2	2.28	2	1.66	2	1.96	2
14030210	2.46	2	2.22	2	2.45	2	2.38	2
14030218	10.49	10	10.08	10	9.88	10	10.15	10
14030222	5.29	5	5.16	5	4.80	5	5.08	5
14030227	3.11	3	3.47	3	3.04	3	3.21	3
14030228	1.69	2	1.67	2	1.59	2	1.65	2
14030232	1.87	2	1.82	2	2.02	2	1.90	2
14030234	3.20	3	2.99	3	2.79	3	2.99	3

Sample ID	Copy Number							
	r = 1		r = 2		r = 3		Average	
	Ratio (target/ref)	Estimated Copy Number	Ratio (target/ref)	Estimated Copy Number	Ratio (target/ref)	Estimated Copy Number	Ratio (target/ref)	Estimated Copy Number
14030236	2.71	3	3.65	4	2.60	3	2.99	3
14030240	7.46	7	6.54	7	7.48	7	7.16	7
14030245	0.85	1	0.81	1	0.87	1	0.84	1
14030248	5.26	5	4.27	4	4.87	5	4.80	5
14030250	5.61	6	4.69	5	4.22	4	4.84	5
14030267	0.37	0	0.39	0	0.34	0	0.37	0
14030268	2.03	2	1.74	2	1.94	2	1.90	2
14030274	3.42	3	3.12	3	3.02	3	3.19	3
14030276	0.25	0	0.28	0	0.23	0	0.25	0
14030279	0.71	1	0.75	1	0.73	1	0.73	1
14030287	1.44	1	1.20	1	1.27	1	1.30	1
14030303	3.99	4	4.49	4	4.12	4	4.20	4
14030310	2.71	3	2.64	3	2.59	3	2.65	3
14030314	2.76	3	3.23	3	3.05	3	3.01	3
14030315	5.60	6	4.69	5	5.61	6	5.30	5
14030319	2.38	2	2.96	3	3.45	3	2.93	3
14030322	0.13	0	0.11	0	0.08	0	0.11	0
14030333	0.97	1	1.09	1	1.11	1	1.06	1
14030335	1.06	1	1.02	1	1.11	1	1.06	1

Sample ID	Copy Number							
	r = 1		r = 2		r = 3		Average	
	Ratio (target/ref)	Estimated Copy Number	Ratio (target/ref)	Estimated Copy Number	Ratio (target/ref)	Estimated Copy Number	Ratio (target/ref)	Estimated Copy Number
14030336	0.87	1	0.84	1	1.03	1	0.91	1
14030343	1.60	2	1.72	2	1.69	2	1.67	2
14030351	0.92	1	0.96	1	0.89	1	0.92	1
14030352	2.17	2	2.04	2	2.39	2	2.20	2
14030353	0.01	0	0.01	0	0.00	0	0.01	0
14030355	15.29	15	14.62	15	16.86	17	15.59	16
14030357	5.37	5	4.98	5	4.74	5	5.03	5
14030360	2.44	2	2.67	3	2.87	3	2.66	3
14030361	2.96	3	2.93	3	2.90	3	2.93	3
14030364	6.83	7	6.96	7	7.00	7	6.93	7
14030367	2.17	2	2.09	2	1.95	2	2.07	2
14030368	4.60	5	4.07	4	4.75	5	4.47	4
14030369	10.67	11	13.15	13	14.95	15	12.92	13
14030382	2.88	3	3.01	3	3.35	3	3.08	3
14030391	3.37	3	3.50	4	4.18	4	3.68	4
14030403	2.36	2	2.05	2	2.21	2	2.21	2
14030411	0.95	1	1.25	1	1.05	1	1.08	1
14030419	0.01	0	0.00	0	0.01	0	0.01	0
14030420	0.01	0	0.01	0	0.02	0	0.01	0

Sample ID	Copy Number							
	r = 1		r = 2		r = 3		Average	
	Ratio (target/ref)	Estimated Copy Number	Ratio (target/ref)	Estimated Copy Number	Ratio (target/ref)	Estimated Copy Number	Ratio (target/ref)	Estimated Copy Number
14030427	2.15	2	2.00	2	1.74	2	1.96	2
14030436	3.84	4	3.92	4	3.73	4	3.83	4
14030439	2.48	2	2.23	2	2.16	2	2.29	2
14030452	4.91	5	4.61	5	5.01	5	4.84	5
14030453	6.81	7	5.17	5	4.69	5	5.56	6
14030489	2.49	2	2.38	2	2.24	2	2.37	2
14030493	0.87	1	1.09	1	1.09	1	1.02	1
14030495	3.09	3	2.79	3	3.25	3	3.04	3
14030498	3.54	4	3.25	3	3.67	4	3.49	3
14030501	0.99	1	1.11	1	0.98	1	1.03	1
14030505	3.70	4	3.62	4	3.14	3	3.49	3
14030507	3.70	4	3.75	4	3.37	3	3.60	4
14030509	1.45	1	1.50	2	1.52	2	1.49	1
14030512	1.74	2	1.62	2	1.64	2	1.66	2
14030513	1.30	1	1.31	1	1.40	1	1.34	1
14030515	1.95	2	1.92	2	1.90	2	1.92	2
14030516	2.10	2	1.99	2	2.38	2	2.16	2
14030520	6.17	6	5.43	5	5.72	6	5.77	6
14030521	1.29	1	1.24	1	1.26	1	1.26	1

Sample ID	Copy Number							
	r = 1		r = 2		r = 3		Average	
	Ratio (target/ref)	Estimated Copy Number	Ratio (target/ref)	Estimated Copy Number	Ratio (target/ref)	Estimated Copy Number	Ratio (target/ref)	Estimated Copy Number
14030532	2.06	2	2.09	2	2.14	2	2.10	2
14030533	1.28	1	1.38	1	1.24	1	1.30	1
14030536	1.17	1	1.03	1	1.07	1	1.09	1
14030539	4.45	4	2.90	3	3.44	3	3.59	4
14030540	6.24	6	5.09	5	5.14	5	5.49	5
14030543	1.16	1	1.13	1	1.07	1	1.12	1
14030544	1.08	1	1.12	1	1.09	1	1.10	1
14030546	2.26	2	2.11	2	1.88	2	2.09	2
14030547	3.30	3	3.20	3	3.27	3	3.26	3
14030548	9.88	10	8.89	9	9.10	9	9.29	9
14030557	1.30	1	1.37	1	1.45	1	1.37	1
14030558	1.17	1	1.09	1	1.23	1	1.16	1
14030559	0.71	1	1.16	1	1.18	1	1.02	1
14030560	2.15	2	1.67	2	1.85	2	1.89	2
14030561	1.32	1	1.37	1	1.61	2	1.43	1
14030562	1.82	2	1.78	2	1.61	2	1.74	2
14030564	1.55	2	1.47	1	1.49	1	1.51	2
14030567	2.61	3	2.62	3	2.58	3	2.61	3
14030569	2.98	3	2.94	3	3.07	3	3.00	3

Sample ID	Copy Number							
	r = 1		r = 2		r = 3		Average	
	Ratio (target/ref)	Estimated Copy Number	Ratio (target/ref)	Estimated Copy Number	Ratio (target/ref)	Estimated Copy Number	Ratio (target/ref)	Estimated Copy Number
14030570	2.08	2	2.05	2	2.14	2	2.09	2
14030571	1.10	1	1.48	1	1.63	2	1.41	1
14030573	3.42	3	3.25	3	3.69	4	3.45	3
14030582	1.99	2	2.07	2	1.87	2	1.98	2
14030583	2.22	2	2.52	3	2.92	3	2.55	3
14030584	1.38	1	1.46	1	1.38	1	1.41	1
14030585	1.02	1	0.91	1	0.93	1	0.95	1
14030586	2.35	2	2.21	2	1.94	2	2.17	2
14030588	3.32	3	3.58	4	3.43	3	3.44	3
14030589	1.14	1	1.33	1	1.54	2	1.34	1
14030591	2.40	2	2.52	3	2.78	3	2.57	3
14030593	1.89	2	1.93	2	1.94	2	1.92	2
14030595	1.96	2	1.94	2	1.88	2	1.93	2
14030597	3.19	3	2.80	3	2.40	2	2.79	3
14030601	1.19	1	1.18	1	1.13	1	1.17	1
14030607	5.58	6	5.28	5	4.45	4	5.10	5
14030608	1.87	2	1.78	2	1.65	2	1.77	2
14030611	2.85	3	2.95	3	2.71	3	2.84	3
14030612	1.38	1	1.43	1	1.23	1	1.35	1

Sample ID	Copy Number							
	r = 1		r = 2		r = 3		Average	
	Ratio (target/ref)	Estimated Copy Number	Ratio (target/ref)	Estimated Copy Number	Ratio (target/ref)	Estimated Copy Number	Ratio (target/ref)	Estimated Copy Number
14030618	4.72	5	4.35	4	4.47	4	4.51	5
14030619	2.27	2	2.44	2	2.04	2	2.25	2
14030620	2.41	2	1.88	2	1.91	2	2.07	2
14030621	2.84	3	2.52	3	2.32	2	2.56	3
14030623	3.04	3	2.79	3	2.45	2	2.76	3
14030624	2.01	2	1.79	2	1.63	2	1.81	2
14030625	1.11	1	1.33	1	1.22	1	1.22	1
14030626	2.93	3	3.06	3	3.09	3	3.03	3
14030653	2.90	3	2.47	2	2.76	3	2.71	3
14030654	2.23	2	1.92	2	2.20	2	2.11	2
14030655	1.14	1	1.22	1	1.05	1	1.14	1
14030658	4.72	5	6.37	6	4.63	5	5.24	5
14030660	6.83	7	6.06	6	4.92	5	5.94	6
14030664	2.36	2	2.50	2	2.49	2	2.45	2
14030665	2.48	2	2.48	2	2.69	3	2.55	3
14030666	6.07	6	7.99	8	9.29	9	7.79	8
14030667	1.16	1	1.16	1	1.24	1	1.19	1
14030668	1.19	1	1.23	1	1.20	1	1.20	1
14030670	6.04	6	3.84	4	4.64	5	4.84	5

Sample ID	Copy Number							
	r = 1		r = 2		r = 3		Average	
	Ratio (target/ref)	Estimated Copy Number	Ratio (target/ref)	Estimated Copy Number	Ratio (target/ref)	Estimated Copy Number	Ratio (target/ref)	Estimated Copy Number
14030671	0.69	1	0.74	1	0.77	1	0.74	1
14030674	1.69	2	1.49	1	1.55	2	1.58	2
14030675	1.45	1	1.27	1	1.32	1	1.35	1
14030677	5.99	6	4.84	5	5.38	5	5.40	5
14030682	1.26	1	1.18	1	1.24	1	1.22	1
14030683	1.22	1	1.18	1	1.09	1	1.16	1
14030684	2.92	3	2.50	3	2.54	3	2.65	3
14030686	1.06	1	1.29	1	1.17	1	1.17	1
14030689	4.72	5	5.02	5	5.17	5	4.97	5
14030694	2.70	3	2.75	3	2.41	2	2.62	3
14030695	2.53	3	2.68	3	3.52	4	2.91	3
14030700	21.51	22	21.59	22	21.02	21	21.37	21
14030711	2.54	3	2.46	2	2.30	2	2.43	2
14030714	2.13	2	2.40	2	2.20	2	2.25	2
14030715	5.62	6	6.21	6	5.28	5	5.70	6
14030720	1.22	1	1.17	1	1.13	1	1.18	1
14030721	3.13	3	2.97	3	3.20	3	3.10	3
14030722	1.55	2	1.25	1	1.42	1	1.40	1
14030723	1.57	2	1.11	1	1.18	1	1.29	1

Sample ID	Copy Number							
	r = 1		r = 2		r = 3		Average	
	Ratio (target/ref)	Estimated Copy Number	Ratio (target/ref)	Estimated Copy Number	Ratio (target/ref)	Estimated Copy Number	Ratio (target/ref)	Estimated Copy Number
14030724	3.87	4	3.44	3	3.41	3	3.57	4
14030725	2.73	3	2.57	3	2.91	3	2.74	3
14030726	2.13	2	2.17	2	2.18	2	2.16	2
14030727	2.01	2	2.35	2	2.00	2	2.12	2
14030728	1.66	2	1.86	2	1.97	2	1.83	2
14030729	2.86	3	3.15	3	4.16	4	3.39	3
14030730	4.66	5	4.64	5	4.47	4	4.59	5
14030731	2.08	2	2.24	2	2.18	2	2.16	2
14030732	9.78	10	9.51	10	9.84	10	9.71	10
14030733	2.04	2	1.81	2	1.68	2	1.84	2
14030734	4.58	5	4.21	4	4.75	5	4.51	5
14030735	3.81	4	3.97	4	3.88	4	3.89	4
14030736	1.88	2	2.03	2	2.03	2	1.98	2
14030737	0.85	1	0.78	1	0.91	1	0.85	1
14030738	1.15	1	1.14	1	1.10	1	1.13	1
14030739	1.45	1	1.48	1	1.56	2	1.50	1
14030740	2.01	2	2.31	2	2.42	2	2.25	2
14030741	4.17	4	4.31	4	4.34	4	4.27	4
14030742	0.50	1	0.53	1	0.44	0	0.49	0

Sample ID	Copy Number							
	r = 1		r = 2		r = 3		Average	
	Ratio (target/ref)	Estimated Copy Number	Ratio (target/ref)	Estimated Copy Number	Ratio (target/ref)	Estimated Copy Number	Ratio (target/ref)	Estimated Copy Number
14030743	1.00	1	1.03	1	1.00	1	1.01	1
14030744	1.65	2	1.20	1	1.61	2	1.49	1
14030745	0.99	1	0.90	1	0.94	1	0.94	1
14030746	1.86	2	2.02	2	1.99	2	1.96	2
14030747	2.76	3	3.04	3	2.94	3	2.91	3
14030748	0.59	1	0.54	1	0.62	1	0.58	1
14030749	2.81	3	2.90	3	3.37	3	3.03	3
14030750	1.65	2	1.99	2	2.05	2	1.90	2
14030751	0.99	1	0.99	1	0.88	1	0.95	1
14030752	1.04	1	0.82	1	0.86	1	0.91	1
14030753	0.53	1	0.45	0	0.55	1	0.51	1
14030754	1.07	1	0.98	1	1.26	1	1.10	1
14030755	1.16	1	1.03	1	1.11	1	1.10	1
14030756	1.17	1	1.19	1	1.02	1	1.12	1
14030757	1.55	2	1.69	2	1.60	2	1.61	2
14030758	2.56	3	2.56	3	2.62	3	2.58	3
14030759	0.31	0	0.22	0	0.24	0	0.26	0
14030760	1.88	2	2.05	2	1.85	2	1.93	2
14030761	1.58	2	1.72	2	1.87	2	1.72	2

Sample ID	Copy Number							
	r = 1		r = 2		r = 3		Average	
	Ratio (target/ref)	Estimated Copy Number	Ratio (target/ref)	Estimated Copy Number	Ratio (target/ref)	Estimated Copy Number	Ratio (target/ref)	Estimated Copy Number
14030762	2.15	2	2.09	2	2.02	2	2.09	2
14030763	5.09	5	5.00	5	5.05	5	5.05	5
14030764	1.29	1	1.04	1	1.31	1	1.21	1
14030765	1.15	1	1.40	1	1.63	2	1.39	1
14030766	0.97	1	1.03	1	0.98	1	0.99	1
14030767	1.06	1	0.99	1	1.06	1	1.04	1
14030768	2.81	3	3.23	3	3.11	3	3.05	3
14030769	4.08	4	3.99	4	4.17	4	4.08	4
14030770	3.76	4	4.25	4	4.65	5	4.22	4
14030771	0.48	0	0.51	1	0.58	1	0.53	1
14030772	0.81	1	1.02	1	1.10	1	0.98	1
14030773	7.53	8	6.94	7	6.69	7	7.05	7
14030774	7.04	7	7.32	7	6.67	7	7.01	7
14030775	8.38	8	7.04	7	6.32	6	7.24	7
14030776	1.64	2	5.62	6	5.86	6	4.38	4
14030777	4.77	5	5.03	5	4.59	5	4.80	5
14030778	4.50	5	4.73	5	4.49	4	4.58	5
14030779	3.23	3	3.47	3	2.99	3	3.23	3
14030780	0.99	1	0.94	1	1.05	1	1.00	1

Sample ID	Copy Number							
	r = 1		r = 2		r = 3		Average	
	Ratio (target/ref)	Estimated Copy Number	Ratio (target/ref)	Estimated Copy Number	Ratio (target/ref)	Estimated Copy Number	Ratio (target/ref)	Estimated Copy Number
14030781	2.25	2	2.26	2	2.14	2	2.21	2
14030782	1.14	1	1.15	1	1.10	1	1.13	1
14030783	5.15	5	3.75	4	4.41	4	4.44	4
14030784	1.18	1	0.96	1	1.12	1	1.08	1
14030785	2.26	2	2.30	2	2.21	2	2.26	2
14030786	1.31	1	1.32	1	1.28	1	1.30	1
14030787	1.18	1	1.03	1	1.09	1	1.10	1
14030788	2.36	2	2.42	2	2.20	2	2.32	2
14030789	2.65	3	2.65	3	2.72	3	2.67	3
14030790	1.05	1	1.12	1	1.06	1	1.08	1
14030791	2.18	2	2.41	2	1.49	1	2.03	2
14030792	1.77	2	1.27	1	1.36	1	1.47	1
14030793	5.04	5	4.70	5	4.56	5	4.77	5
14030794	2.50	2	2.08	2	2.15	2	2.24	2
14030795	2.93	3	2.94	3	2.94	3	2.94	3
14030796	1.96	2	2.02	2	1.87	2	1.95	2
14030797	1.08	1	1.06	1	1.44	1	1.19	1
14030798	1.30	1	1.54	2	1.33	1	1.39	1
14030799	2.26	2	2.31	2	2.22	2	2.26	2

Sample ID	Copy Number							
	r = 1		r = 2		r = 3		Average	
	Ratio (target/ref)	Estimated Copy Number	Ratio (target/ref)	Estimated Copy Number	Ratio (target/ref)	Estimated Copy Number	Ratio (target/ref)	Estimated Copy Number
14030800	2.38	2	1.50	2	1.91	2	1.93	2
14030801	2.03	2	1.90	2	2.06	2	2.00	2
14030802	5.03	5	4.57	5	4.83	5	4.81	5
14030803	5.55	6	5.33	5	5.37	5	5.42	5
14030804	5.11	5	5.57	6	4.77	5	5.15	5
14030805	3.48	3	2.97	3	3.12	3	3.19	3
14030806	5.31	5	4.31	4	4.41	4	4.68	5
14030807	1.03	1	0.95	1	0.97	1	0.98	1
14030808	11.61	12	11.82	12	12.57	13	12.00	12
14030809	11.63	12	11.70	12	10.05	10	11.13	11
14030810	12.39	12	11.20	11	10.88	11	11.49	11
14030811	2.59	3	3.32	3	3.45	3	3.12	3
14030812	15.16	15	17.24	17	16.10	16	16.16	16
14030813	2.94	3	2.79	3	3.01	3	2.91	3
14030814	2.80	3	2.86	3	2.96	3	2.87	3
14030815	2.27	2	2.25	2	2.00	2	2.17	2
14030816	2.13	2	1.86	2	2.01	2	2.00	2
14030817	1.61	2	1.68	2	1.74	2	1.68	2
14030818	0.12	0	0.10	0	0.10	0	0.11	0

Sample ID	Copy Number							
	r = 1		r = 2		r = 3		Average	
	Ratio (target/ref)	Estimated Copy Number	Ratio (target/ref)	Estimated Copy Number	Ratio (target/ref)	Estimated Copy Number	Ratio (target/ref)	Estimated Copy Number
14030819	2.88	3	2.74	3	2.76	3	2.79	3
14030820	1.57	2	1.62	2	1.40	1	1.53	2
14030821	2.36	2	2.16	2	1.98	2	2.17	2
14030822	2.28	2	2.08	2	2.50	2	2.29	2
14030823	2.52	3	2.61	3	2.46	2	2.53	3
14030824	1.02	1	1.17	1	1.23	1	1.14	1
14030825	1.05	1	1.01	1	1.07	1	1.04	1
14030826	1.19	1	0.94	1	1.03	1	1.05	1
14030827	3.20	3	2.68	3	2.74	3	2.87	3
14030828	3.67	4	3.04	3	3.09	3	3.27	3
14030829	1.62	2	2.62	3	2.04	2	2.10	2
14030830	3.19	3	3.17	3	2.31	2	2.89	3
14030831	3.84	4	3.81	4	4.38	4	4.01	4
14030832	1.81	2	1.97	2	2.20	2	1.99	2
14030833	5.39	5	5.02	5	5.38	5	5.27	5
14030834	1.06	1	0.93	1	0.96	1	0.98	1
14030835	3.16	3	2.56	3	2.73	3	2.81	3
14030836	1.05	1	1.17	1	1.05	1	1.09	1
14030837	1.85	2	1.86	2	2.23	2	1.98	2

Sample ID	Copy Number							
	r = 1		r = 2		r = 3		Average	
	Ratio (target/ref)	Estimated Copy Number	Ratio (target/ref)	Estimated Copy Number	Ratio (target/ref)	Estimated Copy Number	Ratio (target/ref)	Estimated Copy Number
14030838	3.42	3	3.59	4	3.63	4	3.55	4
14030839	1.18	1	1.26	1	1.34	1	1.26	1
14030840	2.82	3	3.44	3	3.49	3	3.25	3
14030841	1.23	1	1.48	1	1.18	1	1.29	1
14030842	1.00	1	1.05	1	1.08	1	1.05	1
14030843	3.12	3	3.09	3	2.99	3	3.07	3
14030844	2.91	3	3.10	3	3.23	3	3.08	3
14030845	2.29	2	2.12	2	2.41	2	2.28	2
14030846	3.90	4	3.59	4	3.80	4	3.77	4
14030847	1.79	2	1.81	2	1.59	2	1.73	2
14030848	1.05	1	1.10	1	1.13	1	1.09	1
14030849	2.20	2	2.35	2	2.30	2	2.28	2
14030850	3.12	3	3.04	3	2.84	3	3.00	3
14030851	4.15	4	4.02	4	3.64	4	3.93	4
14030852	1.64	2	1.45	1	1.33	1	1.47	1
14030853	2.15	2	2.15	2	2.01	2	2.10	2
14030854	1.22	1	1.22	1	1.19	1	1.21	1
14030855	2.12	2	2.48	2	2.22	2	2.27	2
14030856	5.17	5	5.83	6	6.10	6	5.70	6

Sample ID	Copy Number							
	r = 1		r = 2		r = 3		Average	
	Ratio (target/ref)	Estimated Copy Number	Ratio (target/ref)	Estimated Copy Number	Ratio (target/ref)	Estimated Copy Number	Ratio (target/ref)	Estimated Copy Number
14030857	1.44	1	1.38	1	1.19	1	1.34	1
14030858	3.21	3	2.78	3	2.63	3	2.87	3
14030859	1.22	1	1.17	1	1.33	1	1.24	1
14030860	3.99	4	4.68	5	4.18	4	4.28	4
14030861	4.21	4	4.10	4	4.58	5	4.30	4
14030862	1.71	2	1.75	2	1.88	2	1.78	2
14030863	1.07	1	1.02	1	1.12	1	1.07	1
14030864	1.73	2	1.70	2	1.69	2	1.71	2
14030865	1.09	1	1.11	1	0.93	1	1.04	1
14030866	3.01	3	2.86	3	2.61	3	2.83	3
14030867	2.57	3	3.06	3	3.11	3	2.91	3
14030868	1.44	1	1.21	1	1.31	1	1.32	1
14030869	1.13	1	1.10	1	1.08	1	1.10	1
14030870	1.74	2	1.66	2	1.34	1	1.58	2
14030871	11.72	12	11.01	11	11.74	12	11.49	11
14030872	0.79	1	0.71	1	0.77	1	0.76	1
14030873	1.19	1	1.14	1	1.16	1	1.16	1
14030874	2.13	2	2.07	2	2.09	2	2.10	2
14030875	1.97	2	1.94	2	1.80	2	1.90	2

Sample ID	Copy Number							
	r = 1		r = 2		r = 3		Average	
	Ratio (target/ref)	Estimated Copy Number	Ratio (target/ref)	Estimated Copy Number	Ratio (target/ref)	Estimated Copy Number	Ratio (target/ref)	Estimated Copy Number
14030876	1.10	1	1.13	1	0.98	1	1.07	1
14030877	1.12	1	1.11	1	1.10	1	1.11	1
14030878	1.17	1	1.22	1	1.34	1	1.24	1
14030879	1.14	1	1.14	1	1.12	1	1.13	1
14030880	1.11	1	1.21	1	1.08	1	1.14	1
14030881	5.67	6	5.95	6	5.79	6	5.80	6
14030882	1.23	1	1.16	1	1.07	1	1.15	1
14030883	3.40	3	2.81	3	2.98	3	3.06	3
14030884	1.15	1	1.19	1	1.13	1	1.15	1
14030885	0.99	1	1.15	1	1.14	1	1.09	1
14030886	3.54	4	2.79	3	2.72	3	3.02	3
14030887	2.92	3	2.89	3	3.37	3	3.06	3
14030888	1.32	1	1.34	1	1.14	1	1.27	1
14030889	3.17	3	3.15	3	3.13	3	3.15	3
14030890	2.11	2	2.20	2	2.31	2	2.21	2
14030891	3.53	4	3.44	3	3.64	4	3.54	4
14030892	1.27	1	1.18	1	1.11	1	1.18	1
14030893	1.01	1	0.99	1	1.00	1	1.00	1
14030894	1.73	2	1.59	2	1.91	2	1.74	2

Sample ID	Copy Number							
	r = 1		r = 2		r = 3		Average	
	Ratio (target/ref)	Estimated Copy Number	Ratio (target/ref)	Estimated Copy Number	Ratio (target/ref)	Estimated Copy Number	Ratio (target/ref)	Estimated Copy Number
14030895	3.71	4	3.49	3	3.33	3	3.51	4
14030896	1.56	2	1.64	2	1.54	2	1.58	2
14030897	1.20	1	1.06	1	0.98	1	1.08	1
14030898	2.03	2	2.18	2	1.99	2	2.07	2
14030899	1.02	1	1.03	1	1.16	1	1.07	1
14030900	1.81	2	1.91	2	1.91	2	1.88	2
14030901	0.99	1	1.11	1	1.10	1	1.07	1
14030902	4.02	4	3.66	4	3.91	4	3.87	4
14030903	3.04	3	2.92	3	3.01	3	2.99	3
14030904	2.00	2	2.03	2	2.50	2	2.18	2
14030905	1.14	1	1.04	1	1.04	1	1.07	1
14030906	1.06	1	1.07	1	1.08	1	1.07	1
14030907	0.95	1	1.02	1	0.82	1	0.93	1
14030908	1.02	1	1.04	1	0.97	1	1.01	1
14030909	1.04	1	0.97	1	1.07	1	1.03	1
14030910	0.99	1	0.97	1	1.04	1	1.00	1
14030911	2.77	3	2.68	3	2.59	3	2.68	3
14030912	0.93	1	1.02	1	0.96	1	0.97	1
14030913	1.05	1	1.13	1	1.12	1	1.10	1

Sample ID	Copy Number							
	r = 1		r = 2		r = 3		Average	
	Ratio (target/ref)	Estimated Copy Number	Ratio (target/ref)	Estimated Copy Number	Ratio (target/ref)	Estimated Copy Number	Ratio (target/ref)	Estimated Copy Number
14030914	3.16	3	3.21	3	3.57	4	3.31	3
14030915	4.47	4	4.06	4	3.90	4	4.14	4
14030916	2.18	2	2.03	2	2.12	2	2.11	2
14030917	1.08	1	1.11	1	1.05	1	1.08	1
14030918	3.04	3	3.15	3	3.45	3	3.21	3
14030919	2.94	3	2.98	3	2.65	3	2.86	3
14030920	11.40	11	11.89	12	11.42	11	11.57	12
14030921	66.41	66	67.77	68	70.07	70	68.08	68
14030922	7.33	7	6.89	7	6.26	6	6.83	7
14030923	2.86	3	2.81	3	2.68	3	2.78	3
14030924	3.72	4	3.72	4	3.23	3	3.56	4
14030925	3.00	3	3.33	3	2.97	3	3.10	3
14030926	3.18	3	3.18	3	3.04	3	3.13	3
14030927	2.64	3	2.94	3	2.80	3	2.80	3
14030928	61.18	61	2.90	3	2.60	3	22.23	22
14030929	7.72	8	7.53	8	7.56	8	7.60	8
14030930	12.98	13	12.79	13	12.73	13	12.83	13
14030931	5.05	5	4.81	5	5.06	5	4.97	5
14030932	1.44	1	1.20	1	1.18	1	1.27	1

Sample ID	Copy Number							
	r = 1		r = 2		r = 3		Average	
	Ratio (target/ref)	Estimated Copy Number	Ratio (target/ref)	Estimated Copy Number	Ratio (target/ref)	Estimated Copy Number	Ratio (target/ref)	Estimated Copy Number
14030933	2.16	2	1.75	2	1.88	2	1.93	2
14030934	4.90	5	4.83	5	4.76	5	4.83	5
14030935	5.16	5	5.08	5	4.92	5	5.05	5
14030936	2.14	2	2.00	2	1.91	2	2.02	2
14030937	6.26	6	5.67	6	5.47	5	5.80	6
14030938	1.94	2	1.99	2	1.90	2	1.94	2
14030939	2.53	3	2.84	3	2.83	3	2.73	3
14030940	1.96	2	2.00	2	1.88	2	1.95	2
14030941	0.65	1	0.66	1	0.68	1	0.66	1
14030942	1.14	1	1.13	1	1.09	1	1.12	1
14030943	1.14	1	1.15	1	1.16	1	1.15	1
14030944	0.86	1	1.16	1	1.22	1	1.08	1
14030945	1.03	1	0.89	1	1.86	2	1.26	1
14030946	3.52	4	2.96	3	4.25	4	3.58	4
14030947	1.77	2	1.95	2	2.52	3	2.08	2
14030948	2.09	2	1.99	2	2.03	2	2.04	2
14030949	2.02	2	2.23	2	1.84	2	2.03	2
14030950	2.51	3	2.56	3	2.79	3	2.62	3
14030951	2.09	2	2.04	2	2.21	2	2.12	2

Sample ID	Copy Number							
	r = 1		r = 2		r = 3		Average	
	Ratio (target/ref)	Estimated Copy Number	Ratio (target/ref)	Estimated Copy Number	Ratio (target/ref)	Estimated Copy Number	Ratio (target/ref)	Estimated Copy Number
14030952	2.86	3	2.67	3	3.38	3	2.97	3
14030953	3.81	4	3.01	3	4.76	5	3.86	4
14030954	3.04	3	2.95	3	2.94	3	2.98	3
14030955	2.53	3	2.50	3	2.59	3	2.54	3
14030956	1.88	2	2.04	2	2.07	2	2.00	2
14030957	1.22	1	1.65	2	2.02	2	1.63	2
14030958	1.52	2	1.47	1	1.43	1	1.47	1
14030959	1.21	1	1.08	1	1.14	1	1.14	1
14030960	1.12	1	1.07	1	1.13	1	1.11	1
14030961	1.19	1	1.25	1	1.22	1	1.22	1
14030962	0.97	1	0.94	1	1.05	1	0.99	1
14030963	1.92	2	2.12	2	2.05	2	2.03	2
14030964	2.19	2	2.08	2	2.09	2	2.12	2
14030965	2.10	2	2.15	2	2.06	2	2.10	2
14030966	2.08	2	3.27	3	2.45	2	2.60	3
14030967	1.26	1	1.31	1	1.39	1	1.32	1
14030968	1.15	1	1.07	1	1.17	1	1.13	1
14030969	6.62	7	6.53	7	7.39	7	6.85	7
14030970	1.10	1	1.94	2	1.36	1	1.47	1

Sample ID	Copy Number							
	r = 1		r = 2		r = 3		Average	
	Ratio (target/ref)	Estimated Copy Number	Ratio (target/ref)	Estimated Copy Number	Ratio (target/ref)	Estimated Copy Number	Ratio (target/ref)	Estimated Copy Number
14030971	14.65	15	13.85	14	13.58	14	14.03	14
14030972	2.01	2	2.04	2	2.14	2	2.06	2
14030973	1.21	1	1.02	1	0.94	1	1.06	1
14030974	1.02	1	1.15	1	1.07	1	1.08	1
14030975	6.43	6	6.87	7	8.19	8	7.17	7
14030976	5.79	6	6.41	6	5.20	5	5.80	6
14030977	3.36	3	3.25	3	3.39	3	3.34	3
14030978	1.15	1	1.16	1	1.23	1	1.18	1
14030979	9.82	10	11.36	11	10.98	11	10.72	11
14030980	1.33	1	1.39	1	1.27	1	1.33	1
14030981	1.74	2	1.41	1	1.33	1	1.49	1
14030982	1.29	1	1.32	1	1.19	1	1.27	1
14030983	1.15	1	1.15	1	1.21	1	1.17	1
14030984	1.36	1	1.33	1	1.37	1	1.35	1
14030985	2.72	3	2.57	3	2.78	3	2.69	3
14030986	1.21	1	1.10	1	1.07	1	1.12	1
14030987	2.48	2	2.17	2	2.17	2	2.27	2
14030988	1.32	1	1.19	1	1.04	1	1.18	1
14030989	4.50	5	4.37	4	4.22	4	4.37	4

Sample ID	Copy Number							
	r = 1		r = 2		r = 3		Average	
	Ratio (target/ref)	Estimated Copy Number	Ratio (target/ref)	Estimated Copy Number	Ratio (target/ref)	Estimated Copy Number	Ratio (target/ref)	Estimated Copy Number
14030990	4.92	5	4.41	4	3.79	4	4.37	4
14030991	1.01	1	1.00	1	1.05	1	1.02	1
14030992	2.01	2	1.99	2	2.04	2	2.01	2
14030993	0.59	1	0.70	1	0.59	1	0.63	1
14030994	23.04	23	22.01	22	22.29	22	22.45	22
14030995	1.20	1	1.05	1	1.27	1	1.17	1
14030996	1.19	1	1.21	1	1.17	1	1.19	1
14030997	1.78	2	1.82	2	1.66	2	1.75	2
14030998	1.02	1	0.90	1	1.00	1	0.97	1
14030999	2.92	3	2.61	3	2.84	3	2.79	3
14031000	0.99	1	0.92	1	1.00	1	0.97	1
14031001	1.00	1	1.06	1	0.99	1	1.02	1
14031002	1.12	1	1.13	1	1.07	1	1.11	1
14031003	1.10	1	1.07	1	1.11	1	1.09	1
14031004	4.52	5	3.97	4	4.22	4	4.23	4
14031005	1.18	1	1.20	1	0.93	1	1.11	1
14031006	1.29	1	1.24	1	1.25	1	1.26	1
14031007	4.51	5	4.85	5	4.58	5	4.65	5
14031008	1.97	2	1.86	2	1.79	2	1.87	2

Sample ID	Copy Number							
	r = 1		r = 2		r = 3		Average	
	Ratio (target/ref)	Estimated Copy Number	Ratio (target/ref)	Estimated Copy Number	Ratio (target/ref)	Estimated Copy Number	Ratio (target/ref)	Average Copy Number
14031009	1.05	1	1.20	1	1.02	1	1.09	1
14031010	1.69	2	1.69	2	2.06	2	1.81	2
14031011	2.19	2	2.16	2	2.06	2	2.13	2
14031012	5.32	5	5.25	5	4.66	5	5.08	5
14031013	1.18	1	1.25	1	1.18	1	1.21	1
14031014	2.37	2	2.37	2	2.30	2	2.35	2
14031015	1.53	2	1.92	2	1.83	2	1.76	2
14031016	1.14	1	1.10	1	1.03	1	1.09	1
14031017	1.04	1	1.15	1	1.04	1	1.08	1
14031018	2.29	2	2.27	2	2.26	2	2.27	2
14031019	17.24	17	16.70	17	16.17	16	16.70	17
14031020	2.33	2	2.39	2	2.25	2	2.32	2
14031021	5.26	5	5.09	5	5.14	5	5.16	5
14031022	8.84	9	8.02	8	7.96	8	8.27	8
14031023	3.26	3	3.36	3	3.23	3	3.28	3
14031024	2.09	2	1.97	2	2.00	2	2.02	2
14031025	6.07	6	6.07	6	5.10	5	5.75	6
14031026	1.85	2	2.07	2	2.32	2	2.08	2
14031027	2.14	2	2.03	2	2.16	2	2.11	2

Sample ID	Copy Number							
	r = 1		r = 2		r = 3		Average	
	Ratio (target/ref)	Estimated Copy Number	Ratio (target/ref)	Estimated Copy Number	Ratio (target/ref)	Estimated Copy Number	Ratio (target/ref)	Estimated Copy Number
14031028	1.22	1	1.29	1	1.17	1	1.23	1
14031029	1.12	1	1.01	1	1.02	1	1.05	1
14031030	2.15	2	1.98	2	2.03	2	2.06	2
14031031	1.13	1	1.03	1	1.29	1	1.15	1
14031032	10.55	11	10.11	10	8.85	9	9.83	10
14031033	2.94	3	2.63	3	2.70	3	2.76	3
14031034	2.97	3	2.99	3	2.68	3	2.88	3
14031035	0.90	1	0.89	1	0.88	1	0.89	1
14031036	4.68	5	4.18	4	4.55	5	4.47	4
14031037	3.65	4	6.60	7	3.57	4	4.61	5
14031038	2.88	3	3.17	3	3.67	4	3.24	3
14031039	3.46	3	3.42	3	2.83	3	3.24	3
14031040	1.11	1	1.03	1	1.02	1	1.06	1
14031041	3.07	3	2.69	3	2.79	3	2.85	3
14031042	1.19	1	1.17	1	1.13	1	1.17	1
14031043	0.65	1	0.69	1	0.59	1	0.64	1
14031044	2.07	2	2.04	2	1.99	2	2.03	2
14031045	9.18	9	9.17	9	9.84	10	9.40	9
14031046	0.82	1	0.92	1	1.18	1	0.98	1

Sample ID	Copy Number							
	r = 1		r = 2		r = 3		Average	
	Ratio (target/ref)	Estimated Copy Number	Ratio (target/ref)	Estimated Copy Number	Ratio (target/ref)	Estimated Copy Number	Ratio (target/ref)	Estimated Copy Number
14031047	1.00	1	1.17	1	1.13	1	1.10	1
14031048	1.05	1	0.95	1	0.98	1	0.99	1
14031049	0.26	0	0.22	0	0.27	0	0.25	0
14031050	2.62	3	2.66	3	2.42	2	2.57	3
14031051	2.18	2	2.00	2	2.11	2	2.10	2
14031052	1.27	1	1.20	1	1.11	1	1.19	1
14031053	3.20	3	3.07	3	3.04	3	3.10	3
14031054	3.35	3	3.20	3	3.43	3	3.33	3
14031055	5.40	5	5.82	6	6.35	6	5.86	6
14031056	2.77	3	2.87	3	2.75	3	2.80	3
14031057	2.79	3	2.74	3	2.50	3	2.68	3
14031058	3.09	3	2.90	3	2.90	3	2.96	3
14031059	1.67	2	1.74	2	1.68	2	1.70	2
14031060	1.80	2	1.72	2	1.73	2	1.75	2
14031061	0.81	1	0.85	1	0.81	1	0.83	1
14031062	3.25	3	3.57	4	3.45	3	3.42	3
14031063	1.20	1	1.06	1	0.96	1	1.07	1
14031064	1.17	1	1.09	1	0.87	1	1.04	1
14031065	1.07	1	1.07	1	1.15	1	1.09	1

Sample ID	Copy Number							
	r = 1		r = 2		r = 3		Average	
	Ratio (target/ref)	Estimated Copy Number	Ratio (target/ref)	Estimated Copy Number	Ratio (target/ref)	Estimated Copy Number	Ratio (target/ref)	Estimated Copy Number
14031066	2.35	2	2.03	2	2.32	2	2.23	2
14031067	2.87	3	3.18	3	3.16	3	3.07	3
14031068	1.45	1	1.44	1	1.36	1	1.42	1
14031069	1.18	1	1.26	1	0.93	1	1.12	1
14031070	3.85	4	3.65	4	2.38	2	3.29	3
14031071	5.55	6	4.83	5	5.15	5	5.18	5
14031072	2.23	2	2.36	2	2.38	2	2.32	2
14031073	4.85	5	5.41	5	6.16	6	5.47	5
14031074	2.43	2	2.33	2	2.16	2	2.30	2
14031075	5.33	5	5.46	5	5.52	6	5.44	5
14031076	3.34	3	3.78	4	3.83	4	3.65	4
14031077	1.20	1	1.22	1	1.21	1	1.21	1
14031078	1.25	1	1.26	1	1.11	1	1.21	1
14031079	1.20	1	1.28	1	1.13	1	1.20	1
14031080	1.11	1	1.04	1	1.04	1	1.06	1
14031081	1.03	1	0.94	1	1.06	1	1.01	1
14031082	3.08	3	3.14	3	2.97	3	3.06	3
14031083	1.27	1	1.16	1	1.23	1	1.22	1
14031084	1.13	1	1.09	1	1.14	1	1.12	1

Sample ID	Copy Number							
	r = 1		r = 2		r = 3		Average	
	Ratio (target/ref)	Estimated Copy Number	Ratio (target/ref)	Estimated Copy Number	Ratio (target/ref)	Estimated Copy Number	Ratio (target/ref)	Estimated Copy Number
14031085	2.19	2	2.43	2	2.34	2	2.32	2
14031086	1.41	1	1.39	1	1.55	2	1.45	1
14031087	3.03	3	3.14	3	3.28	3	3.15	3
14031088	2.04	2	2.05	2	2.11	2	2.07	2
14031089	1.25	1	1.26	1	1.21	1	1.24	1
14031090	1.22	1	1.15	1	1.17	1	1.18	1
14031091	1.06	1	1.07	1	1.02	1	1.05	1
14031092	1.15	1	1.00	1	1.14	1	1.10	1
14031093	1.25	1	1.21	1	1.25	1	1.24	1
14031094	1.97	2	2.20	2	1.93	2	2.03	2
14031095	1.35	1	1.48	1	1.30	1	1.37	1
14031096	3.62	4	3.61	4	3.69	4	3.64	4
14031097	3.55	4	3.52	4	4.20	4	3.76	4
14031098	1.18	1	1.05	1	1.14	1	1.12	1
14031099	2.10	2	2.23	2	2.29	2	2.20	2
14031100	3.09	3	3.04	3	3.25	3	3.13	3
14031101	3.15	3	2.90	3	2.66	3	2.90	3
14031102	4.04	4	4.04	4	3.67	4	3.92	4
14031103	1.22	1	1.17	1	1.18	1	1.19	1

Sample ID	Copy Number							
	r = 1		r = 2		r = 3		Average	
	Ratio (target/ref)	Estimated Copy Number	Ratio (target/ref)	Estimated Copy Number	Ratio (target/ref)	Estimated Copy Number	Ratio (target/ref)	Estimated Copy Number
14031104	1.23	1	1.26	1	1.17	1	1.22	1
14031105	3.30	3	3.12	3	3.50	3	3.30	3
14031106	3.96	4	3.67	4	3.67	4	3.76	4
14031107	2.10	2	1.83	2	2.13	2	2.02	2
14031108	1.20	1	1.41	1	1.19	1	1.26	1
14031109	5.96	6	6.01	6	5.46	5	5.81	6
14031110	1.40	1	1.49	1	1.67	2	1.52	2
14031111	3.18	3	3.23	3	3.31	3	3.24	3
14031112	8.60	9	8.87	9	8.43	8	8.63	9
14031113	1.26	1	1.30	1	1.25	1	1.27	1
14031114	1.25	1	1.32	1	1.19	1	1.25	1
14031115	4.55	5	4.01	4	4.26	4	4.27	4
14031116	2.84	3	2.82	3	2.94	3	2.87	3
14031117	2.38	2	2.30	2	2.36	2	2.35	2
14031118	1.16	1	1.15	1	1.08	1	1.13	1
14031119	1.28	1	1.58	2	1.31	1	1.39	1
14031120	1.18	1	0.90	1	1.00	1	1.03	1
14031121	1.33	1	1.25	1	1.36	1	1.31	1
14031122	2.37	2	2.21	2	2.37	2	2.32	2

Sample ID	Copy Number							
	r = 1		r = 2		r = 3		Average	
	Ratio (target/ref)	Estimated Copy Number	Ratio (target/ref)	Estimated Copy Number	Ratio (target/ref)	Estimated Copy Number	Ratio (target/ref)	Average Copy Number
14031123	2.37	2	2.03	2	2.34	2	2.25	2
14031124	3.58	4	3.01	3	3.17	3	3.25	3
14031125	2.72	3	2.40	2	2.46	2	2.53	3
14031126	2.00	2	3.06	3	2.63	3	2.56	3
14031127	2.56	3	2.55	3	2.24	2	2.45	2
14031128	1.45	1	1.71	2	1.52	2	1.56	2
14031129	1.46	1	1.39	1	1.38	1	1.41	1
14031130	1.31	1	1.04	1	1.09	1	1.15	1
14031131	9.14	9	8.42	8	9.19	9	8.92	9
14031132	2.19	2	2.21	2	2.08	2	2.16	2
14031133	4.49	4	6.62	7	4.37	4	5.16	5
14031134	4.87	5	4.84	5	4.70	5	4.80	5
14031135	4.93	5	5.13	5	4.61	5	4.89	5
14031136	3.40	3	3.00	3	3.19	3	3.20	3
14031137	5.86	6	5.28	5	5.57	6	5.57	6
14031138	1.39	1	1.23	1	1.14	1	1.25	1
14031139	8.78	9	8.91	9	8.27	8	8.65	9
14031140	1.49	1	1.28	1	1.24	1	1.33	1
14031141	4.80	5	5.16	5	4.44	4	4.80	5

Sample ID	Copy Number							
	r = 1		r = 2		r = 3		Average	
	Ratio (target/ref)	Estimated Copy Number	Ratio (target/ref)	Estimated Copy Number	Ratio (target/ref)	Estimated Copy Number	Ratio (target/ref)	Estimated Copy Number
14031142	1.40	1	1.45	1	1.49	1	1.44	1
14031143	5.02	5	4.37	4	4.62	5	4.67	5
14031144	5.92	6	6.10	6	6.19	6	6.07	6
14031145	2.61	3	2.64	3	2.44	2	2.56	3
14031146	2.20	2	2.16	2	2.25	2	2.20	2
14031147	1.28	1	1.29	1	1.37	1	1.31	1
14031148	1.26	1	1.49	1	1.40	1	1.38	1
14031149	4.45	4	4.92	5	4.75	5	4.71	5
14031150	1.83	2	2.17	2	2.29	2	2.10	2
14031151	1.18	1	1.42	1	1.49	1	1.36	1
14031152	1.40	1	1.48	1	1.51	2	1.46	1
14031153	4.30	4	4.86	5	4.96	5	4.71	5
14031154	4.02	4	3.69	4	4.14	4	3.95	4
14031155	2.31	2	2.54	3	2.44	2	2.43	2
14031156	3.09	3	3.33	3	3.43	3	3.28	3
14031157	2.35	2	2.62	3	2.89	3	2.62	3
14031158	3.41	3	3.76	4	4.07	4	3.75	4
14031159	1.66	2	1.59	2	1.41	1	1.55	2
14031160	5.77	6	5.32	5	5.29	5	5.46	5

Sample ID	Copy Number							
	r = 1		r = 2		r = 3		Average	
	Ratio (target/ref)	Estimated Copy Number	Ratio (target/ref)	Estimated Copy Number	Ratio (target/ref)	Estimated Copy Number	Ratio (target/ref)	Estimated Copy Number
14031161	1.48	1	1.53	2	1.46	1	1.49	1
14031162	1.34	1	1.22	1	1.25	1	1.27	1
14031163	3.37	3	3.59	4	3.17	3	3.38	3
14031164	2.29	2	2.57	3	2.16	2	2.34	2
14031165	1.57	2	1.63	2	1.37	1	1.52	2
14031166	4.61	5	4.27	4	4.15	4	4.34	4
14031167	2.79	3	2.68	3	3.38	3	2.95	3
14031168	2.46	2	2.20	2	2.09	2	2.25	2
14031169	9.61	10	8.86	9	8.70	9	9.06	9
14031170	4.31	4	4.17	4	3.40	3	3.96	4
14031171	3.47	3	3.12	3	3.13	3	3.24	3
14031172	1.25	1	1.21	1	1.15	1	1.20	1
14031173	2.60	3	2.43	2	2.32	2	2.45	2
14031174	2.40	2	2.20	2	1.84	2	2.15	2
14031175	5.42	5	5.09	5	4.70	5	5.07	5
14031176	4.15	4	3.84	4	3.57	4	3.86	4
14031177	3.58	4	3.70	4	3.61	4	3.63	4
14031178	2.31	2	2.50	3	2.39	2	2.40	2
14031179	1.62	2	1.42	1	1.34	1	1.46	1

Sample ID	Copy Number							
	r = 1		r = 2		r = 3		Average	
	Ratio (target/ref)	Estimated Copy Number	Ratio (target/ref)	Estimated Copy Number	Ratio (target/ref)	Estimated Copy Number	Ratio (target/ref)	Estimated Copy Number
14031180	1.21	1	1.22	1	1.56	2	1.33	1
14031181	1.47	1	1.37	1	1.40	1	1.42	1
14031182	5.61	6	5.75	6	6.37	6	5.91	6
14031183	3.29	3	2.74	3	3.11	3	3.05	3
14031184	2.48	2	2.98	3	2.86	3	2.77	3
14031185	1.29	1	1.39	1	1.31	1	1.33	1
14031186	1.16	1	1.14	1	1.13	1	1.14	1
14031187	2.21	2	2.13	2	2.28	2	2.21	2
14031188	9.78	10	9.14	9	10.90	11	9.94	10
14031189	1.25	1	1.28	1	1.16	1	1.23	1
14031190	1.15	1	1.26	1	1.23	1	1.21	1
14031191	1.13	1	1.08	1	1.09	1	1.10	1
14031192	4.14	4	3.99	4	3.89	4	4.00	4
14031193	4.08	4	4.36	4	4.21	4	4.21	4
14031194	1.68	2	1.58	2	1.47	1	1.58	2
14031195	6.08	6	5.85	6	5.73	6	5.89	6
14031196	3.27	3	3.44	3	3.24	3	3.32	3
14031197	3.48	3	3.27	3	3.10	3	3.28	3
14031198	1.16	1	1.11	1	1.14	1	1.13	1

Sample ID	Copy Number							
	r = 1		r = 2		r = 3		Average	
	Ratio (target/ref)	Estimated Copy Number	Ratio (target/ref)	Estimated Copy Number	Ratio (target/ref)	Estimated Copy Number	Ratio (target/ref)	Estimated Copy Number
14031199	1.25	1	1.16	1	1.26	1	1.22	1
14031200	3.84	4	3.91	4	4.01	4	3.92	4
14031201	22.11	22	21.55	22	23.33	23	22.33	22
14031202	3.30	3	2.98	3	3.37	3	3.22	3
14031203	2.25	2	2.19	2	2.21	2	2.22	2
14031204	2.54	3	3.00	3	3.04	3	2.86	3
14031205	6.53	7	6.49	6	6.21	6	6.41	6
14031206	1.55	2	1.66	2	2.29	2	1.84	2
14031207	1.52	2	1.54	2	1.67	2	1.58	2
14031208	1.22	1	1.25	1	1.14	1	1.20	1
14031209	1.12	1	1.11	1	1.15	1	1.13	1
14031210	1.31	1	1.28	1	1.51	2	1.37	1
14031211	1.15	1	1.22	1	1.20	1	1.19	1
14031212	1.13	1	1.17	1	1.10	1	1.13	1
14031213	1.14	1	1.21	1	1.42	1	1.26	1
14031214	1.24	1	1.36	1	1.39	1	1.33	1
14031215	2.03	2	2.07	2	1.97	2	2.02	2
14031216	2.20	2	2.14	2	2.43	2	2.26	2
14031217	2.27	2	2.20	2	2.10	2	2.19	2

Sample ID	Copy Number							
	r = 1		r = 2		r = 3		Average	
	Ratio (target/ref)	Estimated Copy Number	Ratio (target/ref)	Estimated Copy Number	Ratio (target/ref)	Estimated Copy Number	Ratio (target/ref)	Estimated Copy Number
14031218	2.20	2	2.31	2	2.18	2	2.23	2
14031219	1.24	1	1.46	1	1.47	1	1.39	1
14031220	3.18	3	3.05	3	3.29	3	3.18	3
14031221	0.90	1	0.91	1	0.97	1	0.93	1
14031222	6.33	6	6.46	6	5.40	5	6.06	6
14031223	1.43	1	1.26	1	1.39	1	1.36	1
14031224	1.65	2	1.56	2	1.51	2	1.57	2
14031225	2.78	3	2.81	3	2.94	3	2.84	3
14031226	2.52	3	2.68	3	2.85	3	2.68	3
14031227	3.92	4	3.60	4	3.55	4	3.69	4
14031228	2.92	3	3.23	3	3.57	4	3.24	3
14031229	1.07	1	0.98	1	1.14	1	1.06	1
14031230	1.21	1	1.12	1	1.18	1	1.17	1
14031231	1.08	1	1.10	1	1.05	1	1.08	1
14031232	1.29	1	1.35	1	1.24	1	1.29	1
14031233	1.55	2	1.52	2	1.39	1	1.49	1
14031234	2.27	2	2.20	2	2.03	2	2.16	2
14031235	3.70	4	3.36	3	3.85	4	3.64	4
14031236	1.37	1	1.45	1	1.54	2	1.45	1

Sample ID	Copy Number							
	r = 1		r = 2		r = 3		Average	
	Ratio (target/ref)	Estimated Copy Number	Ratio (target/ref)	Estimated Copy Number	Ratio (target/ref)	Estimated Copy Number	Ratio (target/ref)	Estimated Copy Number
14031237	3.39	3	3.26	3	3.70	4	3.45	3
14031238	2.68	3	2.51	3	2.70	3	2.63	3
14031239	3.97	4	3.94	4	3.51	4	3.81	4
14031240	2.38	2	2.41	2	2.31	2	2.37	2
14031241	1.25	1	1.30	1	1.21	1	1.25	1
14031242	2.53	3	2.46	2	2.50	3	2.50	2
14031243	3.53	4	2.94	3	3.20	3	3.23	3
14031244	5.54	6	5.31	5	5.18	5	5.34	5
14031245	1.90	2	1.81	2	1.72	2	1.81	2
14031246	2.44	2	2.30	2	2.38	2	2.37	2
14031247	2.50	2	2.41	2	2.32	2	2.41	2
14031248	2.91	3	3.16	3	3.15	3	3.07	3
14031249	2.50	3	2.59	3	2.70	3	2.60	3
14031250	3.13	3	3.07	3	2.25	2	2.82	3
14031251	2.83	3	2.88	3	2.81	3	2.84	3
14031252	1.13	1	0.80	1	1.08	1	1.01	1
14031253	1.28	1	1.22	1	1.27	1	1.26	1
14031254	1.24	1	1.18	1	1.21	1	1.21	1
14031255	1.02	1	0.90	1	0.95	1	0.96	1

Sample ID	Copy Number							
	r = 1		r = 2		r = 3		Average	
	Ratio (target/ref)	Estimated Copy Number	Ratio (target/ref)	Estimated Copy Number	Ratio (target/ref)	Estimated Copy Number	Ratio (target/ref)	Estimated Copy Number
14031256	3.65	4	3.03	3	3.07	3	3.25	3
14031257	1.97	2	1.71	2	1.96	2	1.88	2
14031258	1.90	2	1.64	2	1.84	2	1.80	2
14031259	1.97	2	2.08	2	1.91	2	1.99	2
14031260	1.12	1	0.96	1	1.17	1	1.08	1
14031261	1.10	1	1.08	1	1.20	1	1.13	1
14031262	4.78	5	4.64	5	4.25	4	4.56	5
14031263	7.82	8	7.67	8	7.17	7	7.55	8
14031264	9.00	9	7.23	7	8.34	8	8.19	8
14031265	9.33	9	9.12	9	9.52	10	9.32	9
14031266	2.04	2	2.15	2	1.95	2	2.05	2
14031267	1.14	1	1.16	1	1.13	1	1.14	1
14031268	2.27	2	1.88	2	2.37	2	2.17	2
14031269	1.04	1	0.95	1	0.88	1	0.96	1
14031270	1.53	2	1.35	1	1.10	1	1.33	1
14031271	1.07	1	1.04	1	0.90	1	1.00	1
14031272	1.11	1	1.01	1	1.00	1	1.04	1
14031273	0.83	1	0.94	1	0.96	1	0.91	1
14031274	2.29	2	2.16	2	2.23	2	2.23	2

Sample ID	Copy Number							
	r = 1		r = 2		r = 3		Average	
	Ratio (target/ref)	Estimated Copy Number	Ratio (target/ref)	Estimated Copy Number	Ratio (target/ref)	Estimated Copy Number	Ratio (target/ref)	Estimated Copy Number
14031275	1.89	2	2.03	2	2.21	2	2.04	2
14031276	2.74	3	3.26	3	2.48	2	2.82	3
14031277	2.85	3	3.19	3	3.22	3	3.09	3
14031278	2.46	2	2.51	3	2.92	3	2.63	3
14031279	1.29	1	1.44	1	1.39	1	1.37	1
14031280	6.21	6	6.12	6	5.39	5	5.91	6
14031281	2.19	2	2.12	2	1.91	2	2.07	2
14031282	2.32	2	2.41	2	2.03	2	2.25	2
14031283	4.60	5	4.00	4	4.27	4	4.29	4
14031284	3.77	4	4.17	4	3.59	4	3.84	4
14031285	1.89	2	1.96	2	1.95	2	1.93	2
14031286	2.59	3	3.29	3	2.33	2	2.73	3
14031287	1.63	2	1.58	2	1.71	2	1.64	2
14031288	1.54	2	1.70	2	1.73	2	1.66	2
14031289	2.03	2	2.01	2	2.02	2	2.02	2
14031290	3.12	3	3.00	3	2.63	3	2.92	3
14031291	2.30	2	2.05	2	1.94	2	2.10	2
14031292	2.13	2	2.30	2	2.29	2	2.24	2
14031293	4.83	5	4.24	4	4.32	4	4.47	4

Sample ID	Copy Number							
	r = 1		r = 2		r = 3		Average	
	Ratio (target/ref)	Estimated Copy Number	Ratio (target/ref)	Estimated Copy Number	Ratio (target/ref)	Estimated Copy Number	Ratio (target/ref)	Estimated Copy Number
14031294	2.14	2	2.07	2	2.16	2	2.13	2
14031295	2.55	3	1.99	2	2.27	2	2.27	2
14031296	3.00	3	2.89	3	3.00	3	2.96	3
14031297	3.35	3	2.87	3	2.70	3	2.98	3
14031298	2.22	2	2.11	2	2.01	2	2.11	2
14031299	2.54	3	2.23	2	2.02	2	2.26	2
14031300	1.94	2	2.08	2	2.12	2	2.05	2
14031301	2.07	2	2.18	2	2.22	2	2.16	2
14031302	7.04	7	7.48	7	7.29	7	7.27	7
14031303	6.66	7	5.66	6	6.46	6	6.26	6
14031304	1.12	1	1.06	1	1.09	1	1.09	1
14031305	1.24	1	1.14	1	1.13	1	1.17	1
14031306	1.15	1	1.15	1	1.15	1	1.15	1
14031307	1.42	1	1.23	1	1.24	1	1.30	1
14031308	1.08	1	1.06	1	1.09	1	1.08	1
14031309	4.56	5	4.08	4	4.39	4	4.34	4
14031310	0.38	0	0.33	0	0.46	0	0.39	0
14031311	0.85	1	0.84	1	0.86	1	0.85	1
14031312	2.48	2	2.56	3	2.98	3	2.67	3

Sample ID	Copy Number							
	r = 1		r = 2		r = 3		Average	
	Ratio (target/ref)	Estimated Copy Number	Ratio (target/ref)	Estimated Copy Number	Ratio (target/ref)	Estimated Copy Number	Ratio (target/ref)	Estimated Copy Number
14031313	0.27	0	0.25	0	0.30	0	0.27	0
14031314	1.31	1	1.24	1	1.28	1	1.28	1
14031315	4.43	4	4.05	4	4.33	4	4.27	4
14031316	2.00	2	2.08	2	2.04	2	2.04	2
14031317	2.19	2	1.99	2	2.32	2	2.17	2
14031318	1.17	1	1.14	1	1.11	1	1.14	1
14031319	1.14	1	1.23	1	1.12	1	1.16	1
14031320	0.37	0	0.42	0	0.44	0	0.41	0
14031321	4.57	5	4.36	4	5.08	5	4.67	5
14031322	0.99	1	1.03	1	1.01	1	1.01	1
14031323	1.08	1	1.04	1	1.11	1	1.08	1
14031324	1.07	1	1.17	1	1.01	1	1.09	1
14031325	1.18	1	1.27	1	1.12	1	1.19	1
14031326	1.03	1	1.15	1	1.20	1	1.13	1
14031327	0.95	1	1.07	1	1.09	1	1.03	1
14031328	0.90	1	0.83	1	0.88	1	0.87	1
14031329	1.18	1	1.19	1	1.24	1	1.21	1
14031330	1.91	2	2.07	2	1.98	2	1.99	2
14031331	1.17	1	1.20	1	1.26	1	1.21	1

Sample ID	Copy Number							
	r = 1		r = 2		r = 3		Average	
	Ratio (target/ref)	Estimated Copy Number	Ratio (target/ref)	Estimated Copy Number	Ratio (target/ref)	Estimated Copy Number	Ratio (target/ref)	Estimated Copy Number
14031332	1.15	1	1.11	1	1.00	1	1.09	1
14031333	2.46	2	2.35	2	2.20	2	2.34	2
14031334	2.84	3	3.01	3	2.94	3	2.93	3
14031335	0.68	1	0.70	1	0.79	1	0.72	1
14031336	2.43	2	2.25	2	2.30	2	2.33	2
14031337	1.34	1	1.38	1	1.39	1	1.37	1
14031338	1.68	2	1.68	2	1.66	2	1.67	2
14031339	3.34	3	3.19	3	3.08	3	3.20	3
14031340	21.61	22	21.94	22	20.19	20	21.25	21
14031341	22.05	22	20.14	20	19.57	20	20.59	21
14031342	2.07	2	2.20	2	2.24	2	2.17	2
14031343	3.41	3	3.08	3	2.84	3	3.11	3
14031344	1.39	1	1.57	2	1.55	2	1.50	2
14031345	2.43	2	2.10	2	2.19	2	2.24	2
14031346	5.21	5	6.88	7	5.61	6	5.90	6
14031347	4.03	4	3.34	3	3.57	4	3.65	4
14031348	5.39	5	4.79	5	5.11	5	5.10	5
14031349	1.47	1	1.35	1	1.38	1	1.40	1
14031350	1.29	1	1.29	1	1.26	1	1.28	1

Sample ID	Copy Number							
	r = 1		r = 2		r = 3		Average	
	Ratio (target/ref)	Estimated Copy Number	Ratio (target/ref)	Estimated Copy Number	Ratio (target/ref)	Estimated Copy Number	Ratio (target/ref)	Estimated Copy Number
14031351	11.35	11	11.71	12	11.69	12	11.58	12
14031352	2.35	2	2.36	2	2.30	2	2.34	2
14031353	1.09	1	1.10	1	1.05	1	1.08	1
14031354	1.61	2	1.66	2	1.82	2	1.69	2
14031355	2.03	2	2.23	2	2.22	2	2.16	2
14031356	2.37	2	2.29	2	2.41	2	2.36	2
14031357	2.11	2	2.07	2	2.35	2	2.18	2
14031358	2.41	2	2.22	2	2.16	2	2.26	2
14031359	3.36	3	3.17	3	3.37	3	3.30	3
14031360	1.18	1	1.19	1	1.36	1	1.25	1
14031361	1.31	1	1.33	1	1.36	1	1.33	1
14031362	5.81	6	6.34	6	6.42	6	6.19	6
14031363	4.09	4	3.69	4	3.97	4	3.92	4
14031364	1.15	1	1.20	1	1.18	1	1.18	1
14031365	3.84	4	3.82	4	3.46	3	3.71	4
14031366	1.24	1	1.43	1	1.27	1	1.32	1
14031367	3.32	3	3.15	3	3.36	3	3.27	3
14031368	3.06	3	2.80	3	3.06	3	2.97	3
14031369	10.75	11	11.95	12	13.44	13	12.04	12

Sample ID	Copy Number							
	r = 1		r = 2		r = 3		Average	
	Ratio (target/ref)	Estimated Copy Number	Ratio (target/ref)	Estimated Copy Number	Ratio (target/ref)	Estimated Copy Number	Ratio (target/ref)	Estimated Copy Number
14031370	4.81	5	4.72	5	5.28	5	4.94	5
14031371	1.26	1	1.28	1	1.22	1	1.25	1
14031372	1.79	2	1.83	2	1.51	2	1.71	2
14031373	1.34	1	1.24	1	1.27	1	1.28	1
14031374	0.14	0	0.13	0	0.14	0	0.14	0
14031375	2.78	3	2.81	3	2.87	3	2.82	3
14031376	1.36	1	1.10	1	1.47	1	1.31	1
14031377	1.37	1	1.49	1	1.31	1	1.39	1
14031378	1.29	1	1.12	1	1.28	1	1.23	1
14031379	3.88	4	4.53	5	3.32	3	3.91	4
14031380	1.30	1	1.40	1	1.33	1	1.34	1
14031381	3.32	3	3.27	3	2.93	3	3.17	3
14031382	3.38	3	3.98	4	3.85	4	3.74	4
14031383	2.36	2	1.47	1	1.31	1	1.72	2
14031384	3.10	3	2.65	3	2.87	3	2.88	3
14031385	2.69	3	2.61	3	2.27	2	2.52	3
14031386	2.44	2	2.18	2	2.34	2	2.32	2
14031387	1.42	1	1.46	1	1.53	2	1.47	1
14031388	2.54	3	2.74	3	2.82	3	2.70	3

Sample ID	Copy Number							
	r = 1		r = 2		r = 3		Average	
	Ratio (target/ref)	Estimated Copy Number	Ratio (target/ref)	Estimated Copy Number	Ratio (target/ref)	Estimated Copy Number	Ratio (target/ref)	Estimated Copy Number
14031389	3.37	3	3.59	4	3.73	4	3.56	4
14031390	8.09	8	8.89	9	8.49	8	8.49	8
14031391	21.24	21	21.62	22	18.80	19	20.55	21
14031392	9.58	10	10.14	10	9.23	9	9.65	10
14031393	1.66	2	1.37	1	1.44	1	1.49	1
14031394	2.89	3	2.59	3	2.56	3	2.68	3
14031395	2.53	3	2.65	3	2.22	2	2.47	2
14031396	5.19	5	4.84	5	4.36	4	4.80	5
14031397	3.75	4	4.12	4	4.00	4	3.96	4
14031398	2.50	2	2.70	3	2.22	2	2.48	2
14031399	3.35	3	2.58	3	2.67	3	2.87	3
14031400	2.72	3	2.26	2	2.57	3	2.51	3
14031401	1.37	1	1.23	1	0.75	1	1.12	1
14031402	2.71	3	2.85	3	1.99	2	2.52	3
14031403	2.24	2	1.90	2	1.86	2	2.00	2
14031404	4.75	5	4.75	5	4.73	5	4.74	5
14031405	3.28	3	3.47	3	3.36	3	3.37	3
14031406	1.28	1	1.01	1	1.24	1	1.17	1
14031407	2.43	2	1.82	2	2.54	3	2.26	2

Sample ID	Copy Number							
	r = 1		r = 2		r = 3		Average	
	Ratio (target/ref)	Estimated Copy Number	Ratio (target/ref)	Estimated Copy Number	Ratio (target/ref)	Estimated Copy Number	Ratio (target/ref)	Average Copy Number
14031408	3.88	4	3.43	3	3.41	3	3.58	4
14031409	4.57	5	4.10	4	4.31	4	4.33	4
14031410	1.29	1	1.24	1	1.32	1	1.28	1
14031411	1.47	1	1.20	1	1.45	1	1.38	1
14031412	2.92	3	2.93	3	2.69	3	2.85	3
14031413	1.42	1	1.46	1	1.61	2	1.50	1
14031414	1.57	2	1.73	2	1.84	2	1.71	2
14031415	3.52	4	3.59	4	3.53	4	3.54	4
14031416	2.59	3	2.84	3	2.78	3	2.74	3
14031417	2.53	3	2.95	3	3.32	3	2.93	3
14031418	2.74	3	3.29	3	3.33	3	3.12	3
14031419	17.90	18	17.63	18	17.88	18	17.80	18
14031420	2.53	3	2.55	3	2.43	2	2.50	3
14031421	1.32	1	1.32	1	1.26	1	1.30	1
14031422	4.27	4	4.40	4	4.62	5	4.43	4
14031423	1.29	1	1.17	1	1.16	1	1.21	1
14031424	3.56	4	3.62	4	3.63	4	3.60	4
14031425	3.29	3	2.91	3	3.33	3	3.18	3
14031426	3.55	4	3.60	4	3.22	3	3.46	3

Sample ID	Copy Number							
	r = 1		r = 2		r = 3		Average	
	Ratio (target/ref)	Estimated Copy Number	Ratio (target/ref)	Estimated Copy Number	Ratio (target/ref)	Estimated Copy Number	Ratio (target/ref)	Estimated Copy Number
14031427	4.25	4	3.68	4	3.77	4	3.90	4
14031428	9.49	9	9.40	9	8.82	9	9.24	9
14031429	3.15	3	3.03	3	3.11	3	3.10	3
14031430	4.39	4	4.40	4	4.41	4	4.40	4
14031431	1.18	1	1.08	1	1.13	1	1.13	1
14031432	2.68	3	2.59	3	2.53	3	2.60	3
14031433	1.45	1	1.38	1	1.22	1	1.35	1
14031434	1.53	2	1.35	1	1.34	1	1.40	1
14031435	7.09	7	6.34	6	6.18	6	6.54	7
14031436	1.26	1	1.24	1	1.12	1	1.21	1
14031437	1.16	1	1.17	1	1.05	1	1.13	1
14031438	2.23	2	2.41	2	2.35	2	2.33	2
14031439	5.09	5	5.47	5	4.81	5	5.12	5
14031440	1.20	1	1.05	1	0.99	1	1.08	1
14031441	1.24	1	1.19	1	1.18	1	1.21	1
14031442	2.43	2	2.02	2	2.23	2	2.23	2
14031443	5.96	6	6.27	6	6.36	6	6.19	6
14031444	6.46	6	7.02	7	6.85	7	6.78	7
14031445	2.27	2	2.15	2	1.99	2	2.14	2

Sample ID	Copy Number							
	r = 1		r = 2		r = 3		Average	
	Ratio (target/ref)	Estimated Copy Number	Ratio (target/ref)	Estimated Copy Number	Ratio (target/ref)	Estimated Copy Number	Ratio (target/ref)	Estimated Copy Number
14031446	2.36	2	2.11	2	1.97	2	2.15	2
14031447	1.38	1	1.21	1	1.20	1	1.27	1
14031448	1.19	1	1.16	1	1.21	1	1.18	1
14031449	1.10	1	1.02	1	1.14	1	1.09	1
14031450	0.90	1	0.83	1	0.83	1	0.85	1
14031451	4.31	4	4.59	5	4.76	5	4.55	5
14031452	5.46	5	5.41	5	5.78	6	5.55	6
14031453	5.86	6	5.98	6	5.63	6	5.82	6
14031454	1.50	2	1.54	2	1.53	2	1.52	2
14031455	1.22	1	1.06	1	1.30	1	1.19	1
14031456	1.25	1	1.33	1	1.25	1	1.28	1
14031457	1.05	1	1.07	1	1.09	1	1.07	1
14031458	1.64	2	1.30	1	1.32	1	1.42	1
14031459	1.13	1	1.04	1	1.25	1	1.14	1
14031460	0.41	0	0.45	0	0.44	0	0.43	0
14031461	1.38	1	1.37	1	1.44	1	1.40	1
14031462	3.97	4	3.84	4	4.13	4	3.98	4
14031463	0.51	1	0.44	0	0.54	1	0.49	0
14031464	11.36	11	10.24	10	10.66	11	10.75	11

Sample ID	Copy Number							
	r = 1		r = 2		r = 3		Average	
	Ratio (target/ref)	Estimated Copy Number	Ratio (target/ref)	Estimated Copy Number	Ratio (target/ref)	Estimated Copy Number	Ratio (target/ref)	Estimated Copy Number
14031465	1.88	2	1.88	2	1.83	2	1.86	2
14031466	4.67	5	5.23	5	4.87	5	4.92	5
14031467	1.92	2	1.99	2	2.24	2	2.05	2
14031468	2.19	2	2.37	2	2.34	2	2.30	2
14031469	2.03	2	2.13	2	1.92	2	2.03	2
14031470	1.18	1	1.08	1	1.15	1	1.14	1
14031471	1.17	1	1.20	1	1.11	1	1.16	1
14031472	1.14	1	1.18	1	1.22	1	1.18	1
14031473	1.79	2	1.66	2	1.88	2	1.78	2
14031474	3.52	4	3.59	4	3.35	3	3.49	3
14031475	2.99	3	2.83	3	3.14	3	2.99	3
14031476	2.86	3	3.05	3	2.91	3	2.94	3
14031477	1.09	1	1.14	1	1.08	1	1.10	1
14031478	0.97	1	0.94	1	1.08	1	1.00	1
14031479	1.10	1	1.35	1	1.00	1	1.15	1
14031480	1.61	2	1.40	1	1.60	2	1.54	2
14031481	8.58	9	8.60	9	9.94	10	9.04	9
14031482	1.32	1	1.28	1	1.20	1	1.26	1
14031483	1.06	1	1.01	1	1.11	1	1.06	1

Sample ID	Copy Number							
	r = 1		r = 2		r = 3		Average	
	Ratio (target/ref)	Estimated Copy Number	Ratio (target/ref)	Estimated Copy Number	Ratio (target/ref)	Estimated Copy Number	Ratio (target/ref)	Estimated Copy Number
14031484	1.04	1	1.14	1	1.21	1	1.13	1
14031485	1.26	1	1.16	1	1.39	1	1.27	1
14031486	3.69	4	3.67	4	3.77	4	3.71	4
14031487	5.81	6	5.45	5	5.72	6	5.66	6
14031488	1.78	2	1.85	2	1.87	2	1.83	2
14031489	1.46	1	1.58	2	1.49	1	1.51	2
14031490	1.75	2	1.78	2	1.75	2	1.76	2
14031491	1.16	1	1.26	1	1.10	1	1.17	1
14031492	2.19	2	2.22	2	2.19	2	2.20	2
14031493	1.08	1	0.95	1	1.04	1	1.03	1
14031494	1.29	1	1.23	1	1.15	1	1.22	1
14031495	1.26	1	1.11	1	1.13	1	1.17	1
14040963	1.20	1	1.17	1	1.16	1	1.18	1
14040964	49.74	50	41.99	42	36.19	36	42.64	43
14040967	1.81	2	1.49	1	1.65	2	1.65	2
14040971	1.08	1	0.96	1	0.96	1	1.00	1
14040972	2.12	2	2.24	2	2.21	2	2.19	2
14040974	2.19	2	2.35	2	2.63	3	2.39	2
14040976	3.06	3	2.79	3	2.99	3	2.95	3

Sample ID	Copy Number							
	r = 1		r = 2		r = 3		Average	
	Ratio (target/ref)	Estimated Copy Number	Ratio (target/ref)	Estimated Copy Number	Ratio (target/ref)	Estimated Copy Number	Ratio (target/ref)	Estimated Copy Number
14040977	2.91	3	2.64	3	2.22	2	2.59	3
14040978	5.63	6	5.89	6	6.17	6	5.90	6
14040979	2.28	2	2.05	2	2.24	2	2.19	2
14040980	1.84	2	1.97	2	1.83	2	1.88	2
14040982	1.89	2	1.76	2	1.12	1	1.59	2
14040983	1.69	2	1.92	2	1.84	2	1.82	2
14040985	1.66	2	1.80	2	1.84	2	1.77	2
14040986	1.62	2	1.60	2	1.82	2	1.68	2
14040987	1.91	2	2.22	2	2.43	2	2.19	2
14040988	1.19	1	1.10	1	1.10	1	1.13	1
14040989	1.03	1	1.06	1	1.37	1	1.15	1
14040990	1.24	1	1.20	1	1.25	1	1.23	1
14040991	1.14	1	1.14	1	1.16	1	1.15	1
14040992	1.10	1	1.18	1	1.23	1	1.17	1
14040993	1.02	1	1.09	1	0.94	1	1.02	1
14040994	0.88	1	1.00	1	1.07	1	0.98	1
14040995	1.06	1	1.15	1	1.07	1	1.09	1
14040999	1.85	2	2.16	2	1.71	2	1.90	2
14041000	2.34	2	2.42	2	2.56	3	2.44	2

Sample ID	Copy Number							
	r = 1		r = 2		r = 3		Average	
	Ratio (target/ref)	Estimated Copy Number	Ratio (target/ref)	Estimated Copy Number	Ratio (target/ref)	Estimated Copy Number	Ratio (target/ref)	Estimated Copy Number
14041001	1.18	1	1.12	1	1.04	1	1.11	1
14041002	1.03	1	1.02	1	1.05	1	1.04	1
14041003	4.03	4	4.21	4	3.57	4	3.94	4
14041006	1.12	1	1.03	1	1.13	1	1.09	1
14041007	1.16	1	1.03	1	1.13	1	1.11	1
14041009	2.45	2	2.39	2	2.79	3	2.54	3
14041010	1.11	1	1.05	1	1.07	1	1.07	1
14041011	4.58	5	4.60	5	3.98	4	4.39	4
14041012	3.23	3	3.27	3	3.12	3	3.21	3
14041013	3.17	3	3.18	3	3.47	3	3.27	3
14041014	4.93	5	5.86	6	5.51	6	5.43	5
14041015	3.03	3	3.02	3	2.88	3	2.98	3
14041016	6.07	6	5.73	6	5.80	6	5.87	6
14041017	1.29	1	1.04	1	1.09	1	1.14	1
14041018	1.09	1	1.15	1	1.08	1	1.11	1
14041019	0.49	0	0.49	0	0.53	1	0.50	1
14041020	3.22	3	3.11	3	3.26	3	3.19	3
14041021	1.96	2	2.14	2	1.78	2	1.96	2
14041023	0.44	0	0.48	0	0.49	0	0.47	0

Sample ID	Copy Number							
	r = 1		r = 2		r = 3		Average	
	Ratio (target/ref)	Estimated Copy Number	Ratio (target/ref)	Estimated Copy Number	Ratio (target/ref)	Estimated Copy Number	Ratio (target/ref)	Estimated Copy Number
14041025	1.74	2	1.61	2	1.40	1	1.58	2
14041030	1.03	1	1.08	1	1.02	1	1.04	1
14041032	1.01	1	0.99	1	1.06	1	1.02	1
14041033	0.99	1	1.01	1	1.00	1	1.00	1
14041035	1.44	1	1.41	1	1.52	2	1.45	1
14041036	0.88	1	0.86	1	0.95	1	0.90	1
14041037	0.11	0	0.09	0	0.08	0	0.09	0
14041038	3.40	3	2.79	3	2.99	3	3.06	3
14041040	2.01	2	1.90	2	2.05	2	1.99	2
14041041	2.22	2	2.12	2	2.10	2	2.15	2
14041044	2.21	2	2.09	2	1.98	2	2.09	2
14041045	1.14	1	1.13	1	1.05	1	1.11	1
14041046	1.09	1	1.12	1	1.18	1	1.13	1
14041047	1.17	1	1.08	1	1.18	1	1.14	1
14041048	1.11	1	1.06	1	1.04	1	1.07	1
14041049	6.05	6	6.34	6	6.16	6	6.18	6
14041050	5.51	6	6.25	6	6.92	7	6.23	6
14041053	1.96	2	1.93	2	1.92	2	1.94	2
14041054	1.87	2	1.90	2	2.07	2	1.95	2

Sample ID	Copy Number							
	r = 1		r = 2		r = 3		Average	
	Ratio (target/ref)	Estimated Copy Number	Ratio (target/ref)	Estimated Copy Number	Ratio (target/ref)	Estimated Copy Number	Ratio (target/ref)	Estimated Copy Number
14041055	2.11	2	1.94	2	1.78	2	1.94	2
14041056	2.10	2	2.10	2	1.90	2	2.03	2
14041059	1.49	1	1.63	2	1.56	2	1.56	2
14041060	0.61	1	0.54	1	0.60	1	0.58	1
14041063	1.33	1	1.18	1	1.26	1	1.26	1
14041064	1.14	1	1.16	1	1.18	1	1.16	1
14041066	1.05	1	1.12	1	1.09	1	1.09	1
14041068	3.20	3	2.83	3	3.04	3	3.02	3
14041069	13.34	13	12.92	13	15.63	16	13.96	14
14041073	3.29	3	3.08	3	3.00	3	3.12	3
14041077	2.59	3	2.36	2	2.21	2	2.39	2
14041082	2.84	3	2.58	3	2.39	2	2.60	3
14041083	3.22	3	3.27	3	3.14	3	3.21	3
14041085	4.46	4	4.50	5	4.28	4	4.41	4
14041087	1.07	1	1.19	1	1.12	1	1.13	1
14041088	7.54	8	7.53	8	7.23	7	7.43	7
14041089	5.91	6	6.01	6	5.99	6	5.97	6
14041092	2.37	2	2.13	2	2.31	2	2.27	2
14041093	1.74	2	1.89	2	2.09	2	1.90	2

Sample ID	Copy Number							
	r = 1		r = 2		r = 3		Average	
	Ratio (target/ref)	Estimated Copy Number	Ratio (target/ref)	Estimated Copy Number	Ratio (target/ref)	Estimated Copy Number	Ratio (target/ref)	Estimated Copy Number
14041094	5.73	6	5.92	6	5.55	6	5.73	6
14041095	5.48	5	5.25	5	6.44	6	5.72	6
14041097	1.24	1	1.30	1	1.13	1	1.22	1
14041098	3.38	3	3.22	3	3.71	4	3.44	3
14041099	4.99	5	5.08	5	4.88	5	4.98	5
14041100	1.44	1	1.45	1	1.45	1	1.45	1
14041104	4.59	5	4.35	4	4.16	4	4.36	4
14041110	1.86	2	2.26	2	2.08	2	2.07	2
14041111	4.59	5	4.59	5	4.84	5	4.67	5
14041112	2.57	3	2.87	3	2.60	3	2.68	3
14041114	1.01	1	1.13	1	1.10	1	1.08	1
14041115	1.03	1	1.04	1	1.05	1	1.04	1
14041116	2.73	3	2.68	3	2.60	3	2.67	3
14041117	1.22	1	1.06	1	1.07	1	1.12	1
14041119	1.17	1	1.12	1	0.99	1	1.09	1
14041120	2.26	2	1.91	2	1.95	2	2.04	2
14041121	13.46	13	13.80	14	9.77	10	12.34	12
14041124	2.23	2	1.96	2	2.32	2	2.17	2
14041125	2.18	2	2.22	2	2.17	2	2.19	2

Sample ID	Copy Number							
	r = 1		r = 2		r = 3		Average	
	Ratio (target/ref)	Estimated Copy Number	Ratio (target/ref)	Estimated Copy Number	Ratio (target/ref)	Estimated Copy Number	Ratio (target/ref)	Estimated Copy Number
14041129	4.89	5	5.04	5	4.59	5	4.84	5
14041130	1.37	1	1.35	1	1.25	1	1.32	1
14041131	1.33	1	1.24	1	1.13	1	1.23	1
14041138	2.21	2	1.93	2	1.96	2	2.03	2
14041139	2.55	3	2.21	2	2.18	2	2.31	2
14041140	3.53	4	3.79	4	3.72	4	3.68	4
14041141	4.01	4	3.40	3	3.70	4	3.70	4
14041142	4.03	4	4.55	5	4.45	4	4.35	4
14041143	3.64	4	3.74	4	3.55	4	3.64	4
14041145	1.01	1	1.03	1	1.05	1	1.03	1
14041146	0.98	1	0.97	1	1.21	1	1.05	1
14041147	1.07	1	1.02	1	1.07	1	1.05	1
14041149	0.92	1	0.93	1	1.11	1	0.99	1
14041150	1.79	2	1.76	2	1.79	2	1.78	2
14041151	5.18	5	5.13	5	5.31	5	5.21	5
14041152	3.32	3	3.29	3	3.72	4	3.44	3
14041164	1.36	1	1.38	1	1.26	1	1.34	1
14041165	1.48	1	1.59	2	1.73	2	1.60	2
14041166	16.54	17	17.59	18	17.71	18	17.28	17

Sample ID	Copy Number							
	r = 1		r = 2		r = 3		Average	
	Ratio (target/ref)	Estimated Copy Number	Ratio (target/ref)	Estimated Copy Number	Ratio (target/ref)	Estimated Copy Number	Ratio (target/ref)	Estimated Copy Number
14041167	30.31	30	32.55	33	33.28	33	32.05	32
14041168	0.95	1	1.04	1	1.05	1	1.01	1
14041177	1.81	2	1.99	2	2.17	2	1.99	2
14041179	1.02	1	0.99	1	1.08	1	1.03	1
14041182	2.84	3	3.02	3	3.12	3	3.00	3
14041183	2.72	3	2.89	3	2.64	3	2.75	3
14041185	1.92	2	2.20	2	2.08	2	2.07	2
14041187	2.15	2	1.98	2	1.92	2	2.02	2
14041191	0.94	1	0.94	1	0.92	1	0.93	1
14041193	1.07	1	0.94	1	0.94	1	0.98	1
14041195	2.63	3	2.71	3	2.88	3	2.74	3
14041197	1.47	1	1.40	1	1.58	2	1.48	1
14041201	1.11	1	0.90	1	1.03	1	1.01	1
14041206	10.11	10	9.64	10	9.22	9	9.66	10
14041209	1.14	1	1.02	1	1.06	1	1.08	1
14041210	1.04	1	1.08	1	0.94	1	1.02	1
14041217	1.15	1	1.01	1	1.05	1	1.07	1
14041219	2.02	2	1.97	2	1.84	2	1.94	2
14041221	3.34	3	3.65	4	3.58	4	3.52	4

Sample ID	Copy Number							
	r = 1		r = 2		r = 3		Average	
	Ratio (target/ref)	Estimated Copy Number	Ratio (target/ref)	Estimated Copy Number	Ratio (target/ref)	Estimated Copy Number	Ratio (target/ref)	Estimated Copy Number
14041222	0.35	0	0.39	0	0.55	1	0.43	0
14041226	0.99	1	1.06	1	0.77	1	0.94	1
14041227	5.32	5	6.32	6	7.65	8	6.43	6
14041228	5.34	5	7.62	8	6.65	7	6.53	7
14041235	0.94	1	1.04	1	1.16	1	1.05	1
14041236	2.55	3	3.33	3	4.01	4	3.30	3
14041237	2.77	3	2.59	3	2.71	3	2.69	3
14041238	2.50	2	2.38	2	2.49	2	2.46	2
14041244	2.82	3	3.27	3	3.35	3	3.15	3
14041253	2.54	3	2.16	2	2.68	3	2.46	2
14041260	5.04	5	4.73	5	5.29	5	5.02	5
14041261	6.94	7	6.69	7	7.11	7	6.91	7
14041267	2.88	3	2.69	3	2.87	3	2.81	3
14041271	5.79	6	6.32	6	7.18	7	6.43	6
14041272	0.90	1	0.89	1	1.07	1	0.95	1
14041275	0.95	1	0.99	1	0.94	1	0.96	1
14041276	4.28	4	5.07	5	4.51	5	4.62	5
14041277	0.94	1	0.87	1	0.98	1	0.93	1
14041280	2.79	3	3.03	3	2.90	3	2.91	3

Sample ID	Copy Number							
	r = 1		r = 2		r = 3		Average	
	Ratio (target/ref)	Estimated Copy Number	Ratio (target/ref)	Estimated Copy Number	Ratio (target/ref)	Estimated Copy Number	Ratio (target/ref)	Estimated Copy Number
14041282	3.32	3	4.56	5	3.42	3	3.77	4
14041286	3.84	4	3.28	3	3.14	3	3.42	3
14041287	5.21	5	4.92	5	4.71	5	4.95	5
14041288	2.01	2	1.97	2	1.96	2	1.98	2
14041289	2.76	3	2.50	3	2.69	3	2.65	3
14041294	1.92	2	2.88	3	2.03	2	2.28	2
14041295	2.93	3	2.41	2	2.74	3	2.69	3
14041297	2.06	2	1.79	2	1.85	2	1.90	2
14041300	7.01	7	7.20	7	7.02	7	7.08	7
14041302	1.95	2	1.98	2	2.07	2	2.00	2
14041303	9.30	9	9.04	9	8.93	9	9.09	9
14041313	1.87	2	2.11	2	2.08	2	2.02	2
14041317	2.06	2	2.07	2	2.30	2	2.14	2
14041321	3.68	4	4.09	4	3.99	4	3.92	4
14041322	2.27	2	2.25	2	2.42	2	2.32	2
14041323	3.51	4	4.77	5	3.86	4	4.05	4
14041329	2.11	2	2.11	2	2.30	2	2.17	2
14041330	1.29	1	1.99	2	2.15	2	1.81	2
14041331	1.64	2	1.48	1	1.47	1	1.53	2

Sample ID	Copy Number							
	r = 1		r = 2		r = 3		Average	
	Ratio (target/ref)	Estimated Copy Number	Ratio (target/ref)	Estimated Copy Number	Ratio (target/ref)	Estimated Copy Number	Ratio (target/ref)	Estimated Copy Number
14041333	2.60	3	2.61	3	2.77	3	2.66	3
14041334	3.99	4	3.83	4	3.88	4	3.90	4
14041335	0.67	1	0.69	1	0.66	1	0.67	1
14041336	1.86	2	1.50	2	1.37	1	1.58	2
14041337	1.52	2	1.39	1	1.21	1	1.37	1
14041339	1.21	1	1.01	1	0.90	1	1.04	1
14041340	1.91	2	1.55	2	1.37	1	1.61	2
14041341	1.63	2	1.38	1	1.36	1	1.46	1
14041342	0.62	1	0.62	1	0.55	1	0.59	1
14041343	1.76	2	1.74	2	1.89	2	1.80	2
14041344	1.51	2	1.52	2	1.42	1	1.48	1
14041345	1.46	1	1.47	1	1.46	1	1.46	1
14041346	3.97	4	3.92	4	4.00	4	3.97	4
14041347	1.31	1	1.46	1	1.40	1	1.39	1
14041356	1.16	1	1.11	1	1.12	1	1.13	1
14041357	1.39	1	1.33	1	1.34	1	1.35	1
14041361	6.61	7	6.77	7	6.39	6	6.59	7
14041362	1.44	1	1.32	1	1.23	1	1.33	1
14041364	4.44	4	3.82	4	3.81	4	4.02	4

Sample ID	Copy Number							
	r = 1		r = 2		r = 3		Average	
	Ratio (target/ref)	Estimated Copy Number	Ratio (target/ref)	Estimated Copy Number	Ratio (target/ref)	Estimated Copy Number	Ratio (target/ref)	Estimated Copy Number
14041365	4.39	4	4.12	4	4.71	5	4.41	4
14041368	2.47	2	2.91	3	2.45	2	2.61	3
14041371	2.50	2	2.35	2	2.63	3	2.49	2
14041372	1.26	1	1.29	1	1.11	1	1.22	1
14041374	3.63	4	3.22	3	3.33	3	3.39	3
14041376	2.10	2	2.17	2	2.08	2	2.11	2
14041378	5.46	5	5.26	5	4.38	4	5.03	5
14041379	3.07	3	3.17	3	3.35	3	3.20	3
14041381	4.35	4	4.45	4	4.86	5	4.55	5
14041383	3.95	4	3.97	4	3.86	4	3.93	4
14041384	4.18	4	3.57	4	4.68	5	4.14	4
14041387	3.43	3	2.96	3	2.87	3	3.08	3
14041391	8.60	9	8.31	8	6.89	7	7.94	8
14041392	1.40	1	1.20	1	1.26	1	1.29	1
14041394	1.25	1	1.30	1	1.37	1	1.31	1
14041396	1.12	1	1.26	1	1.07	1	1.15	1
14041399	2.21	2	2.08	2	2.00	2	2.10	2
14041401	3.57	4	3.71	4	3.36	3	3.55	4
14041403	1.24	1	1.24	1	1.24	1	1.24	1

Sample ID	Copy Number							
	r = 1		r = 2		r = 3		Average	
	Ratio (target/ref)	Estimated Copy Number	Ratio (target/ref)	Estimated Copy Number	Ratio (target/ref)	Estimated Copy Number	Ratio (target/ref)	Estimated Copy Number
14041406	2.62	3	2.39	2	2.58	3	2.53	3
14041407	2.26	2	2.70	3	2.18	2	2.38	2
14041408	3.80	4	2.83	3	3.58	4	3.40	3
14041409	2.65	3	2.04	2	1.95	2	2.21	2
14041412	4.13	4	4.08	4	3.88	4	4.03	4
14041415	2.33	2	2.25	2	2.16	2	2.25	2
14041418	2.44	2	2.44	2	2.39	2	2.42	2
14041419	4.40	4	4.24	4	4.39	4	4.35	4
14041421	2.33	2	2.39	2	2.27	2	2.33	2
14041424	1.35	1	1.48	1	1.29	1	1.37	1
14041427	2.97	3	3.04	3	3.69	4	3.23	3
14041430	2.33	2	1.90	2	2.03	2	2.09	2
14041431	3.61	4	4.06	4	3.62	4	3.77	4
14041433	2.16	2	2.32	2	2.68	3	2.39	2
14041435	3.43	3	3.21	3	3.47	3	3.37	3
14041436	3.05	3	3.32	3	3.04	3	3.14	3
14041437	11.75	12	15.60	16	16.34	16	14.56	15
14041438	16.85	17	19.26	19	21.01	21	19.04	19
14041445	4.62	5	4.64	5	4.03	4	4.43	4

Sample ID	Copy Number							
	r = 1		r = 2		r = 3		Average	
	Ratio (target/ref)	Estimated Copy Number	Ratio (target/ref)	Estimated Copy Number	Ratio (target/ref)	Estimated Copy Number	Ratio (target/ref)	Estimated Copy Number
14041446	1.84	2	1.87	2	1.95	2	1.89	2
14041449	2.47	2	2.82	3	3.09	3	2.79	3
14041450	1.74	2	1.99	2	2.07	2	1.93	2
14041455	1.43	1	0.61	1	1.51	2	1.19	1
14041460	4.41	4	4.51	5	4.48	4	4.47	4
14041464	8.86	9	7.86	8	7.97	8	8.23	8
14041466	1.49	1	1.23	1	1.31	1	1.34	1
14041467	1.57	2	1.33	1	1.27	1	1.39	1
14041468	1.56	2	1.41	1	1.45	1	1.47	1
14041470	1.23	1	1.24	1	1.20	1	1.22	1
14041478	4.11	4	4.54	5	4.85	5	4.50	4
14041479	2.41	2	2.42	2	2.39	2	2.41	2
14041481	2.27	2	2.33	2	2.13	2	2.24	2
14041483	2.14	2	2.13	2	2.25	2	2.17	2
14041485	1.27	1	1.25	1	1.14	1	1.22	1
14041494	2.36	2	2.88	3	2.21	2	2.48	2
14041495	1.97	2	2.05	2	2.08	2	2.03	2
14041496	1.44	1	1.53	2	1.31	1	1.43	1
14041502	2.15	2	2.09	2	2.32	2	2.19	2

Sample ID	Copy Number							
	r = 1		r = 2		r = 3		Average	
	Ratio (target/ref)	Estimated Copy Number	Ratio (target/ref)	Estimated Copy Number	Ratio (target/ref)	Estimated Copy Number	Ratio (target/ref)	Estimated Copy Number
14041507	2.94	3	3.20	3	3.27	3	3.14	3
14041512	1.22	1	1.22	1	1.26	1	1.23	1
14041514	2.66	3	2.35	2	2.15	2	2.39	2
14041515	2.50	2	2.27	2	2.44	2	2.40	2
14041518	2.27	2	2.44	2	2.52	3	2.41	2
14041524	3.46	3	3.43	3	3.63	4	3.51	4
14041528	1.36	1	1.51	2	1.53	2	1.47	1
14041529	1.13	1	1.24	1	1.07	1	1.15	1
14041531	0.36	0	0.33	0	0.31	0	0.33	0
14041540	3.17	3	3.26	3	3.25	3	3.23	3
14041541	4.82	5	4.51	5	4.44	4	4.59	5
14041542	3.34	3	3.07	3	3.36	3	3.26	3
14041543	3.41	3	3.14	3	3.58	4	3.38	3
14041550	1.96	2	2.56	3	2.23	2	2.25	2
14041551	2.28	2	2.48	2	2.31	2	2.36	2
14041552	2.59	3	2.55	3	2.46	2	2.53	3
14041555	1.28	1	1.34	1	1.29	1	1.30	1
14041561	1.24	1	1.08	1	1.14	1	1.15	1
14041563	3.07	3	3.07	3	3.40	3	3.18	3

Sample ID	Copy Number							
	r = 1		r = 2		r = 3		Average	
	Ratio (target/ref)	Estimated Copy Number	Ratio (target/ref)	Estimated Copy Number	Ratio (target/ref)	Estimated Copy Number	Ratio (target/ref)	Average Copy Number
14041567	1.69	2	1.54	2	1.59	2	1.61	2
14041568	3.19	3	3.14	3	2.59	3	2.97	3
14041577	2.20	2	2.20	2	2.02	2	2.14	2
14041580	1.42	1	1.45	1	1.44	1	1.43	1
14041581	1.21	1	1.23	1	1.29	1	1.24	1
14041582	1.39	1	1.37	1	1.42	1	1.39	1
14041587	3.46	3	3.40	3	3.46	3	3.44	3
14041588	2.46	2	2.50	3	2.32	2	2.43	2
14041593	1.27	1	1.40	1	1.16	1	1.28	1
14041594	3.19	3	3.16	3	3.01	3	3.12	3
14041596	2.37	2	2.50	3	2.29	2	2.39	2
14041611	3.19	3	2.82	3	3.28	3	3.10	3
14041630	1.23	1	1.17	1	1.30	1	1.23	1
14041643	1.44	1	1.54	2	1.56	2	1.51	2
14041653	4.07	4	4.17	4	3.85	4	4.03	4
14041659	3.11	3	2.88	3	2.96	3	2.98	3
14041660	2.52	3	2.40	2	2.08	2	2.33	2
14041663	3.68	4	3.45	3	3.33	3	3.49	3
14041664	1.02	1	1.04	1	0.99	1	1.02	1

Sample ID	Copy Number							
	r = 1		r = 2		r = 3		Average	
	Ratio (target/ref)	Estimated Copy Number	Ratio (target/ref)	Estimated Copy Number	Ratio (target/ref)	Estimated Copy Number	Ratio (target/ref)	Estimated Copy Number
14041676	3.27	3	2.88	3	3.23	3	3.13	3
14041679	7.04	7	5.30	5	6.56	7	6.30	6
14041688	2.36	2	2.19	2	2.20	2	2.25	2
14041690	3.15	3	3.08	3	2.88	3	3.04	3
14041691	9.20	9	8.34	8	7.44	7	8.33	8
14041692	9.72	10	8.68	9	8.21	8	8.87	9
14041693	9.95	10	9.37	9	9.82	10	9.71	10
14041694	1.16	1	1.09	1	1.18	1	1.14	1
14041698	2.55	3	2.45	2	2.50	3	2.50	2
14041699	0.68	1	0.63	1	0.66	1	0.66	1
14041701	1.18	1	1.12	1	1.16	1	1.16	1
14041703	1.06	1	1.13	1	1.08	1	1.09	1
14041704	2.11	2	2.20	2	2.03	2	2.11	2
14041705	2.98	3	3.02	3	2.87	3	2.96	3
14041706	2.69	3	2.57	3	2.10	2	2.45	2
14041711	1.34	1	1.30	1	1.30	1	1.31	1
14041712	2.33	2	2.16	2	2.15	2	2.22	2
14041715	8.97	9	8.73	9	8.48	8	8.73	9
14041716	9.71	10	9.75	10	9.99	10	9.82	10

Sample ID	Copy Number							
	r = 1		r = 2		r = 3		Average	
	Ratio (target/ref)	Estimated Copy Number	Ratio (target/ref)	Estimated Copy Number	Ratio (target/ref)	Estimated Copy Number	Ratio (target/ref)	Estimated Copy Number
14041721	3.66	4	4.66	5	4.74	5	4.35	4
14041722	4.50	5	4.27	4	4.26	4	4.34	4
14041726	5.85	6	6.85	7	6.28	6	6.32	6
14041727	5.73	6	5.44	5	4.48	4	5.22	5
14041731	0.73	1	0.77	1	0.83	1	0.78	1
14041734	2.39	2	1.96	2	2.07	2	2.14	2
14041740	1.20	1	1.17	1	1.27	1	1.22	1
14041741	2.15	2	2.44	2	2.37	2	2.32	2
14041746	9.46	9	8.84	9	9.85	10	9.38	9
14041747	10.80	11	10.90	11	10.49	10	10.73	11
14041754	1.25	1	1.19	1	1.20	1	1.21	1
14041760	1.35	1	1.30	1	0.90	1	1.18	1
14041762	1.27	1	1.21	1	1.33	1	1.27	1
14041763	2.33	2	2.61	3	2.48	2	2.47	2
14041764	1.42	1	1.45	1	1.33	1	1.40	1
14041766	8.53	9	10.26	10	8.61	9	9.13	9
14041769	2.20	2	2.07	2	1.92	2	2.06	2
14041770	1.26	1	1.28	1	1.21	1	1.25	1
14041772	2.71	3	2.49	2	2.50	2	2.57	3

Sample ID	Copy Number							
	r = 1		r = 2		r = 3		Average	
	Ratio (target/ref)	Estimated Copy Number	Ratio (target/ref)	Estimated Copy Number	Ratio (target/ref)	Estimated Copy Number	Ratio (target/ref)	Estimated Copy Number
14041773	2.36	2	2.40	2	2.53	3	2.43	2
14041777	1.63	2	1.77	2	1.57	2	1.66	2
14041779	1.20	1	1.26	1	1.25	1	1.23	1
14041781	4.09	4	4.05	4	3.56	4	3.90	4
14041785	1.48	1	1.15	1	1.56	2	1.40	1
14041789	1.29	1	1.33	1	1.18	1	1.27	1
14041790	0.95	1	1.37	1	1.24	1	1.19	1
14041792	1.35	1	1.30	1	1.32	1	1.32	1
14041795	1.87	2	2.42	2	2.25	2	2.18	2
14041799	1.16	1	1.40	1	2.11	2	1.56	2
14041800	1.21	1	1.29	1	1.46	1	1.32	1
14041801	2.14	2	0.52	1	2.16	2	1.60	2
14041803	1.19	1	1.15	1	1.15	1	1.16	1
14041804	1.27	1	1.46	1	1.50	1	1.41	1
14041805	1.38	1	1.34	1	1.30	1	1.34	1
14041810	8.90	9	10.78	11	6.74	7	8.81	9
14041815	6.03	6	6.92	7	8.31	8	7.09	7
14041819	1.35	1	1.26	1	0.95	1	1.19	1
14041821	0.92	1	1.91	2	1.68	2	1.50	2

Sample ID	Copy Number							
	r = 1		r = 2		r = 3		Average	
	Ratio (target/ref)	Estimated Copy Number	Ratio (target/ref)	Estimated Copy Number	Ratio (target/ref)	Estimated Copy Number	Ratio (target/ref)	Estimated Copy Number
14041823	1.21	1	1.31	1	1.20	1	1.24	1
14041824	1.41	1	1.18	1	1.22	1	1.27	1
14041826	2.07	2	2.31	2	1.90	2	2.10	2
14041827	1.12	1	1.28	1	1.28	1	1.23	1
14041828	1.23	1	1.39	1	1.54	2	1.39	1
14041834	10.73	11	11.74	12	12.01	12	11.49	11
14041835	6.64	7	6.73	7	7.09	7	6.82	7
14041836	6.06	6	5.69	6	5.41	5	5.72	6
14041837	1.95	2	2.01	2	1.93	2	1.96	2
14041838	3.42	3	3.42	3	3.27	3	3.37	3
14041839	5.43	5	4.86	5	4.66	5	4.98	5
14041841	6.52	7	6.34	6	6.37	6	6.41	6
14041842	4.11	4	4.08	4	3.86	4	4.01	4
14041845	1.21	1	1.34	1	1.22	1	1.25	1
14041850	1.27	1	1.30	1	1.16	1	1.24	1
14041851	5.09	5	4.97	5	4.69	5	4.92	5
14041852	1.32	1	1.15	1	1.20	1	1.22	1
14041854	3.98	4	4.27	4	4.57	5	4.28	4
14041855	4.80	5	4.58	5	4.45	4	4.61	5

Sample ID	Copy Number							
	r = 1		r = 2		r = 3		Average	
	Ratio (target/ref)	Estimated Copy Number	Ratio (target/ref)	Estimated Copy Number	Ratio (target/ref)	Estimated Copy Number	Ratio (target/ref)	Estimated Copy Number
14041856	4.68	5	4.53	5	4.55	5	4.59	5
14041865	1.29	1	1.48	1	1.44	1	1.40	1
14041867	2.65	3	2.62	3	2.63	3	2.63	3
14041868	2.42	2	2.42	2	2.39	2	2.41	2
14041869	1.57	2	1.52	2	1.66	2	1.58	2
14041872	2.91	3	3.32	3	3.65	4	3.30	3
14041873	2.42	2	2.46	2	2.61	3	2.50	2
14041881	1.20	1	1.26	1	1.40	1	1.29	1
14041883	2.19	2	2.32	2	2.44	2	2.32	2
14041886	5.90	6	5.56	6	5.71	6	5.72	6
14041889	1.52	2	1.36	1	1.25	1	1.38	1
14041891	2.26	2	2.29	2	2.17	2	2.24	2
14041894	1.29	1	1.29	1	1.14	1	1.24	1
14041895	10.08	10	9.10	9	9.21	9	9.47	9
14041899	1.22	1	1.34	1	1.19	1	1.25	1
14041905	1.24	1	1.18	1	1.22	1	1.21	1
14041907	2.73	3	2.67	3	2.73	3	2.71	3
14041915	1.26	1	1.23	1	1.25	1	1.25	1
14041916	1.33	1	1.18	1	1.25	1	1.25	1

Sample ID	Copy Number							
	r = 1		r = 2		r = 3		Average	
	Ratio (target/ref)	Estimated Copy Number	Ratio (target/ref)	Estimated Copy Number	Ratio (target/ref)	Estimated Copy Number	Ratio (target/ref)	Estimated Copy Number
14041917	2.69	3	2.60	3	2.49	2	2.59	3
14041918	2.17	2	1.96	2	2.12	2	2.08	2
14041930	4.43	4	4.39	4	4.27	4	4.36	4
14041933	0.68	1	0.67	1	0.68	1	0.68	1
14041938	3.45	3	3.60	4	3.57	4	3.54	4
14041941	11.90	12	13.41	13	12.45	12	12.59	13
14041942	2.26	2	1.74	2	2.59	3	2.19	2
14041943	1.99	2	1.97	2	2.03	2	2.00	2
14041949	1.13	1	1.13	1	1.14	1	1.13	1
14041950	1.51	2	1.30	1	1.31	1	1.37	1
14041953	2.76	3	3.01	3	2.63	3	2.80	3
14041963	3.11	3	3.00	3	3.02	3	3.04	3
14041965	1.19	1	1.35	1	1.22	1	1.26	1
14041968	7.31	7	7.84	8	7.41	7	7.52	8
14041969	1.28	1	1.16	1	1.23	1	1.22	1
14041970	1.04	1	1.14	1	1.27	1	1.15	1
14041972	1.23	1	1.23	1	1.20	1	1.22	1
14041973	1.41	1	1.48	1	1.63	2	1.51	2
14041974	1.34	1	1.56	2	1.54	2	1.48	1

Sample ID	Copy Number							
	r = 1		r = 2		r = 3		Average	
	Ratio (target/ref)	Estimated Copy Number	Ratio (target/ref)	Estimated Copy Number	Ratio (target/ref)	Estimated Copy Number	Ratio (target/ref)	Estimated Copy Number
14041978	3.16	3	3.20	3	3.37	3	3.24	3
14041980	2.05	2	2.23	2	2.34	2	2.21	2
14041981	2.06	2	2.17	2	2.45	2	2.23	2
14041982	2.25	2	1.89	2	2.45	2	2.20	2
14041983	2.13	2	2.30	2	2.12	2	2.18	2
14041985	1.17	1	1.17	1	1.10	1	1.15	1
14041987	1.41	1	1.32	1	1.26	1	1.33	1
14041989	1.29	1	1.29	1	1.40	1	1.32	1
14041993	1.15	1	1.14	1	1.11	1	1.13	1
14041994	1.25	1	1.15	1	1.20	1	1.20	1
14041995	1.25	1	1.49	1	1.50	1	1.41	1
14041996	1.36	1	1.39	1	1.43	1	1.39	1
14041997	1.27	1	1.31	1	1.41	1	1.33	1
14041998	11.06	11	10.71	11	11.05	11	10.94	11
14042003	4.45	4	4.19	4	4.13	4	4.26	4
14042004	1.30	1	1.32	1	1.21	1	1.28	1
14042005	5.06	5	3.74	4	4.16	4	4.32	4
14042007	3.10	3	2.79	3	2.75	3	2.88	3
14042008	2.96	3	2.95	3	3.04	3	2.98	3

Sample ID	Copy Number							
	r = 1		r = 2		r = 3		Average	
	Ratio (target/ref)	Estimated Copy Number	Ratio (target/ref)	Estimated Copy Number	Ratio (target/ref)	Estimated Copy Number	Ratio (target/ref)	Estimated Copy Number
14042010	4.02	4	4.29	4	4.60	5	4.31	4
14042013	1.34	1	1.30	1	1.19	1	1.28	1
14042014	2.67	3	2.85	3	2.80	3	2.77	3
14042016	3.01	3	2.94	3	2.70	3	2.88	3
14042018	11.90	12	10.19	10	10.56	11	10.88	11
14042019	1.36	1	1.24	1	1.23	1	1.28	1
14042020	1.41	1	1.40	1	1.20	1	1.34	1
14042027	8.06	8	7.73	8	8.20	8	8.00	8
14042028	3.95	4	4.57	5	5.01	5	4.51	5
14042029	0.75	1	0.76	1	0.81	1	0.77	1
14042032	1.22	1	1.19	1	1.16	1	1.19	1
14042034	1.21	1	1.30	1	1.33	1	1.28	1
14042039	1.23	1	1.25	1	1.31	1	1.26	1
14042040	1.42	1	1.38	1	1.53	2	1.44	1
14042046	1.14	1	1.17	1	1.02	1	1.11	1
14042048	1.04	1	1.22	1	1.32	1	1.20	1
14042049	1.29	1	1.33	1	1.13	1	1.25	1
14042050	1.27	1	1.20	1	1.20	1	1.22	1
14042061	5.32	5	5.80	6	5.48	5	5.54	6

Sample ID	Copy Number							
	r = 1		r = 2		r = 3		Average	
	Ratio (target/ref)	Estimated Copy Number	Ratio (target/ref)	Estimated Copy Number	Ratio (target/ref)	Estimated Copy Number	Ratio (target/ref)	Estimated Copy Number
14042062	1.11	1	1.08	1	1.03	1	1.08	1
14042063	14.08	14	15.05	15	14.55	15	14.56	15
14042067	2.27	2	2.19	2	2.35	2	2.27	2
14042068	4.03	4	4.11	4	4.72	5	4.29	4
14042070	0.22	0	0.19	0	0.17	0	0.19	0
14042071	2.33	2	2.05	2	2.11	2	2.16	2
14042073	1.22	1	1.11	1	1.31	1	1.22	1
14042074	1.44	1	1.44	1	1.44	1	1.44	1
14042077	9.07	9	8.87	9	9.44	9	9.13	9
14042078	2.16	2	2.17	2	1.94	2	2.09	2
14042079	2.20	2	1.96	2	2.23	2	2.13	2
14042082	1.20	1	1.25	1	1.10	1	1.18	1
14042083	4.05	4	4.03	4	4.05	4	4.04	4
14042086	1.17	1	1.05	1	1.06	1	1.09	1
14042087	1.09	1	1.07	1	0.78	1	0.98	1
14042088	1.17	1	1.20	1	1.17	1	1.18	1
14042090	1.17	1	1.13	1	1.23	1	1.18	1
14042091	2.23	2	2.24	2	2.29	2	2.25	2
14042092	2.30	2	2.37	2	2.42	2	2.36	2

Sample ID	Copy Number							
	r = 1		r = 2		r = 3		Average	
	Ratio (target/ref)	Estimated Copy Number	Ratio (target/ref)	Estimated Copy Number	Ratio (target/ref)	Estimated Copy Number	Ratio (target/ref)	Estimated Copy Number
14042093	13.39	13	12.96	13	11.65	12	12.67	13
14042094	1.07	1	1.03	1	0.90	1	1.00	1
14042095	4.09	4	3.78	4	3.52	4	3.80	4
14042096	5.55	6	5.51	6	5.52	6	5.52	6
14042097	5.40	5	5.40	5	5.88	6	5.56	6
14042098	3.33	3	3.25	3	3.30	3	3.30	3
14042099	6.70	7	6.15	6	5.77	6	6.20	6
14042100	7.57	8	6.65	7	7.24	7	7.15	7
14042105	1.23	1	1.08	1	1.11	1	1.14	1
14042106	1.26	1	1.22	1	1.24	1	1.24	1
14050001	1.55	2	1.29	1	1.30	1	1.38	1
14050002	1.40	1	1.00	1	1.27	1	1.22	1
14050003	1.46	1	1.34	1	1.37	1	1.39	1
14050006	2.01	2	2.22	2	1.83	2	2.02	2
14050008	1.12	1	1.28	1	1.39	1	1.26	1
14050009	1.22	1	1.17	1	1.29	1	1.23	1
14050010	1.36	1	1.37	1	1.37	1	1.37	1
14050014	5.43	5	5.18	5	5.53	6	5.38	5
14050015	3.70	4	3.91	4	3.60	4	3.74	4

Sample ID	Copy Number							
	r = 1		r = 2		r = 3		Average	
	Ratio (target/ref)	Estimated Copy Number	Ratio (target/ref)	Estimated Copy Number	Ratio (target/ref)	Estimated Copy Number	Ratio (target/ref)	Estimated Copy Number
14050021	1.37	1	0.96	1	1.24	1	1.19	1
14050028	2.20	2	2.65	3	2.30	2	2.38	2
14050029	1.33	1	1.13	1	1.22	1	1.23	1
14050030	1.25	1	1.34	1	0.98	1	1.19	1
14050031	1.30	1	1.06	1	1.30	1	1.22	1
14050032	1.71	2	1.68	2	1.74	2	1.71	2
14050033	14.61	15	11.38	11	13.08	13	13.02	13
14050034	1.48	1	1.18	1	1.36	1	1.34	1
14050035	1.40	1	1.34	1	1.50	2	1.41	1
14050041	1.63	2	1.25	1	1.83	2	1.57	2
14050044	4.86	5	4.60	5	4.64	5	4.70	5
14050046	1.20	1	1.48	1	1.49	1	1.39	1
14050049	2.88	3	2.76	3	2.93	3	2.86	3
14050051	3.35	3	2.99	3	3.01	3	3.12	3
14050058	1.56	2	1.17	1	1.31	1	1.35	1
14050059	2.33	2	2.37	2	2.49	2	2.40	2
14050061	3.96	4	3.87	4	4.37	4	4.07	4
14050064	3.56	4	3.23	3	3.49	3	3.43	3
14050066	13.21	13	12.07	12	10.78	11	12.02	12

Sample ID	Copy Number							
	r = 1		r = 2		r = 3		Average	
	Ratio (target/ref)	Estimated Copy Number	Ratio (target/ref)	Estimated Copy Number	Ratio (target/ref)	Estimated Copy Number	Ratio (target/ref)	Estimated Copy Number
14050068	1.48	1	1.25	1	1.40	1	1.38	1
14050070	1.26	1	1.30	1	0.91	1	1.15	1
14050074	4.95	5	5.56	6	4.08	4	4.86	5
14050075	5.05	5	4.58	5	4.71	5	4.78	5
14050077	3.59	4	3.44	3	3.55	4	3.52	4
14050079	1.32	1	1.28	1	1.22	1	1.27	1
14050081	2.01	2	1.79	2	1.88	2	1.89	2
14050082	3.03	3	2.51	3	2.47	2	2.67	3
14050088	1.70	2	1.39	1	1.49	1	1.53	2
14050092	6.61	7	5.60	6	8.37	8	6.86	7
14050093	9.72	10	10.71	11	8.95	9	9.79	10
14050096	6.42	6	6.49	6	4.97	5	5.96	6
14050100	2.61	3	2.13	2	2.33	2	2.36	2
14050104	2.79	3	2.99	3	2.96	3	2.91	3
14050105	3.26	3	2.83	3	2.65	3	2.92	3
14050110	1.09	1	1.17	1	1.01	1	1.09	1
14050112	1.28	1	1.20	1	1.29	1	1.26	1
14050113	1.27	1	1.27	1	1.36	1	1.30	1
14050114	1.39	1	1.42	1	1.37	1	1.39	1

Sample ID	Copy Number							
	r = 1		r = 2		r = 3		Average	
	Ratio (target/ref)	Estimated Copy Number	Ratio (target/ref)	Estimated Copy Number	Ratio (target/ref)	Estimated Copy Number	Ratio (target/ref)	Estimated Copy Number
14050118	2.81	3	3.13	3	2.51	3	2.82	3
14050119	4.10	4	3.56	4	4.32	4	3.99	4
14050120	3.65	4	3.27	3	3.40	3	3.44	3
14050121	2.28	2	2.40	2	2.24	2	2.30	2
14050125	1.85	2	2.20	2	2.09	2	2.05	2
14050128	4.76	5	4.85	5	3.85	4	4.49	4
14050129	2.94	3	3.30	3	3.39	3	3.21	3
14050130	3.30	3	3.35	3	3.62	4	3.42	3
14050135	3.87	4	4.23	4	4.10	4	4.06	4
14050137	2.74	3	2.78	3	2.91	3	2.81	3
14050140	3.38	3	3.24	3	3.59	4	3.40	3
14050141	2.80	3	2.82	3	2.89	3	2.83	3
14050142	6.01	6	6.54	7	6.05	6	6.20	6
14050144	2.20	2	2.46	2	2.13	2	2.27	2
14050145	4.72	5	4.44	4	5.08	5	4.75	5
14050147	2.28	2	2.37	2	2.30	2	2.32	2
14050150	2.71	3	2.89	3	2.67	3	2.76	3
14050152	1.30	1	1.43	1	1.24	1	1.32	1
14050155	1.30	1	1.34	1	1.38	1	1.34	1

Sample ID	Copy Number							
	r = 1		r = 2		r = 3		Average	
	Ratio (target/ref)	Estimated Copy Number	Ratio (target/ref)	Estimated Copy Number	Ratio (target/ref)	Estimated Copy Number	Ratio (target/ref)	Estimated Copy Number
14050157	1.33	1	1.23	1	1.44	1	1.33	1
14050158	1.40	1	1.29	1	1.20	1	1.30	1
14050160	1.48	1	1.45	1	1.50	2	1.48	1
14050163	1.37	1	1.42	1	1.65	2	1.48	1
14050165	1.04	1	1.10	1	1.11	1	1.08	1
14050167	4.65	5	4.82	5	4.99	5	4.82	5
14050174	5.25	5	4.81	5	4.80	5	4.95	5
14050175	4.83	5	5.13	5	4.54	5	4.84	5
14050183	1.32	1	1.31	1	1.28	1	1.30	1
14050189	2.28	2	2.09	2	2.16	2	2.18	2
14050190	2.72	3	2.49	2	2.45	2	2.55	3
14050191	2.47	2	2.59	3	2.67	3	2.58	3
14050193	5.27	5	5.35	5	5.50	5	5.37	5
14050194	5.47	5	5.67	6	5.72	6	5.62	6
14050199	2.32	2	2.41	2	2.43	2	2.39	2
14050200	2.18	2	2.15	2	2.47	2	2.27	2
14050201	2.23	2	2.28	2	2.35	2	2.29	2
14050203	1.35	1	1.34	1	1.54	2	1.41	1
14050204	4.55	5	4.74	5	4.89	5	4.73	5

Sample ID	Copy Number							
	r = 1		r = 2		r = 3		Average	
	Ratio (target/ref)	Estimated Copy Number	Ratio (target/ref)	Estimated Copy Number	Ratio (target/ref)	Estimated Copy Number	Ratio (target/ref)	Estimated Copy Number
14050209	15.12	15	14.49	14	15.18	15	14.93	15
14050210	14.06	14	14.31	14	14.76	15	14.37	14
14050211	3.41	3	3.15	3	3.06	3	3.21	3
14050215	3.20	3	3.23	3	3.32	3	3.25	3
14050216	4.59	5	4.60	5	5.16	5	4.78	5
14050217	3.95	4	3.36	3	3.26	3	3.52	4
14050218	4.30	4	4.25	4	3.94	4	4.16	4
14050219	2.62	3	2.73	3	2.47	2	2.61	3
14050223	1.53	2	1.51	2	1.48	1	1.51	2
14050225	2.67	3	2.36	2	2.56	3	2.53	3
14050226	2.15	2	2.14	2	2.21	2	2.16	2
14050240	2.79	3	2.82	3	2.80	3	2.80	3
14050244	0.53	1	0.58	1	0.15	0	0.42	0
14050245	2.58	3	2.61	3	2.74	3	2.64	3
14050246	1.90	2	1.77	2	1.84	2	1.84	2
14050248	0.57	1	0.56	1	0.62	1	0.58	1
14050269	1.51	2	1.54	2	1.38	1	1.48	1
14050270	1.46	1	1.52	2	1.35	1	1.44	1
14050276	1.78	2	1.33	1	1.49	1	1.54	2

Sample ID	Copy Number							
	r = 1		r = 2		r = 3		Average	
	Ratio (target/ref)	Estimated Copy Number	Ratio (target/ref)	Estimated Copy Number	Ratio (target/ref)	Estimated Copy Number	Ratio (target/ref)	Estimated Copy Number
14050277	1.34	1	1.30	1	1.18	1	1.27	1
14050278	1.48	1	0.22	0	1.50	1	1.06	1
14050279	1.45	1	1.51	2	1.06	1	1.34	1
14050283	3.91	4	1.85	2	4.44	4	3.40	3
14050286	1.45	1	1.45	1	1.46	1	1.46	1
14050290	1.50	1	1.29	1	1.44	1	1.41	1
14050291	1.44	1	1.31	1	1.33	1	1.36	1
14050293	4.11	4	4.19	4	4.10	4	4.13	4
14050296	2.67	3	2.35	2	2.41	2	2.48	2
14050298	3.33	3	3.43	3	7.05	7	4.61	5
14050299	1.44	1	1.27	1	1.52	2	1.41	1
14050301	2.20	2	2.60	3	3.45	3	2.75	3
14050304	4.55	5	5.76	6	5.93	6	5.41	5
14050306	1.51	2	1.49	1	1.52	2	1.51	2
14050309	1.90	2	2.14	2	1.90	2	1.98	2
14050310	1.17	1	1.39	1	1.19	1	1.25	1
14050311	1.37	1	1.26	1	1.37	1	1.33	1
14050312	6.54	7	10.20	10	9.91	10	8.89	9
14050313	8.18	8	8.15	8	8.58	9	8.30	8

Sample ID	Copy Number							
	r = 1		r = 2		r = 3		Average	
	Ratio (target/ref)	Estimated Copy Number	Ratio (target/ref)	Estimated Copy Number	Ratio (target/ref)	Estimated Copy Number	Ratio (target/ref)	Estimated Copy Number
14050315	2.41	2	2.10	2	1.93	2	2.15	2
14050316	1.69	2	2.58	3	2.21	2	2.16	2
14050319	1.90	2	2.13	2	1.99	2	2.01	2
14050321	1.32	1	1.32	1	1.38	1	1.34	1
14050328	3.62	4	3.60	4	3.51	4	3.58	4
14050329	7.78	8	7.81	8	7.77	8	7.79	8
14050330	3.26	3	3.43	3	4.30	4	3.66	4
14050331	2.64	3	1.92	2	2.14	2	2.23	2
14050332	2.34	2	2.19	2	2.18	2	2.24	2
14050337	1.35	1	1.32	1	1.25	1	1.31	1
14050343	2.37	2	2.47	2	2.09	2	2.31	2
14050345	2.98	3	3.33	3	3.19	3	3.17	3
14050346	1.31	1	1.28	1	1.42	1	1.34	1
14050347	4.08	4	3.90	4	4.16	4	4.05	4
14050353	2.14	2	2.14	2	2.27	2	2.18	2
14050355	1.33	1	1.36	1	1.20	1	1.29	1
14050359	3.90	4	3.69	4	3.91	4	3.84	4
14050360	3.84	4	4.29	4	4.50	4	4.21	4
14050363	3.48	3	3.28	3	3.24	3	3.33	3

Sample ID	Copy Number							
	r = 1		r = 2		r = 3		Average	
	Ratio (target/ref)	Estimated Copy Number	Ratio (target/ref)	Estimated Copy Number	Ratio (target/ref)	Estimated Copy Number	Ratio (target/ref)	Estimated Copy Number
14050367	7.39	7	6.52	7	7.01	7	6.97	7
14050368	7.34	7	7.21	7	7.17	7	7.24	7
14050377	4.58	5	5.20	5	4.03	4	4.60	5
14050378	4.32	4	4.30	4	4.80	5	4.47	4
14050380	2.12	2	2.15	2	2.19	2	2.15	2
14050382	6.01	6	6.34	6	7.71	8	6.68	7
14050383	1.26	1	1.52	2	1.37	1	1.38	1
14050385	1.37	1	1.57	2	1.38	1	1.44	1
14050386	1.58	2	1.34	1	1.43	1	1.45	1
14050387	1.25	1	1.31	1	1.30	1	1.29	1
14050388	1.23	1	1.40	1	1.31	1	1.31	1
14050392	1.00	1	0.96	1	1.08	1	1.01	1
14050393	1.48	1	1.36	1	1.44	1	1.43	1
14050395	1.40	1	1.33	1	1.39	1	1.37	1
14050398	1.67	2	1.55	2	1.47	1	1.57	2
14050402	1.59	2	1.39	1	1.34	1	1.44	1
14050404	1.24	1	1.28	1	1.27	1	1.26	1
14050406	1.42	1	1.38	1	1.53	2	1.44	1
14050407	1.17	1	1.14	1	1.22	1	1.18	1

Sample ID	Copy Number							
	r = 1		r = 2		r = 3		Average	
	Ratio (target/ref)	Estimated Copy Number	Ratio (target/ref)	Estimated Copy Number	Ratio (target/ref)	Estimated Copy Number	Ratio (target/ref)	Estimated Copy Number
14050408	2.47	2	2.37	2	2.45	2	2.43	2
14050410	1.48	1	1.44	1	1.38	1	1.43	1
14050411	3.25	3	3.26	3	3.06	3	3.19	3
14050412	4.86	5	4.92	5	5.17	5	4.98	5
14050414	2.61	3	2.69	3	2.08	2	2.46	2
14050415	4.78	5	4.81	5	4.86	5	4.82	5
14050419	2.14	2	2.21	2	2.05	2	2.13	2
14050420	2.09	2	2.38	2	2.17	2	2.21	2
14050421	2.54	3	2.48	2	2.56	3	2.53	3
14050422	1.14	1	1.29	1	1.39	1	1.27	1
14050424	1.35	1	1.22	1	1.33	1	1.30	1
14050425	5.92	6	6.41	6	6.25	6	6.19	6
14050428	1.20	1	1.10	1	1.28	1	1.19	1
14050429	3.20	3	2.74	3	3.01	3	2.98	3
14050431	2.37	2	2.16	2	2.31	2	2.28	2
14050432	2.29	2	2.09	2	2.31	2	2.23	2
14050433	2.08	2	1.96	2	1.62	2	1.89	2
14050434	1.51	2	1.24	1	1.42	1	1.39	1
14050440	3.33	3	2.85	3	3.07	3	3.09	3

Sample ID	Copy Number							
	r = 1		r = 2		r = 3		Average	
	Ratio (target/ref)	Estimated Copy Number	Ratio (target/ref)	Estimated Copy Number	Ratio (target/ref)	Estimated Copy Number	Ratio (target/ref)	Estimated Copy Number
14050441	3.18	3	3.15	3	3.35	3	3.23	3
14050446	6.79	7	6.45	6	6.95	7	6.73	7
14050449	1.33	1	1.44	1	1.50	1	1.42	1
14050452	2.12	2	2.41	2	1.83	2	2.12	2
14050453	1.88	2	1.41	1	1.21	1	1.50	1
14050457	3.12	3	2.98	3	3.45	3	3.19	3
14050463	2.92	3	3.64	4	2.77	3	3.11	3
14050464	2.42	2	2.13	2	2.43	2	2.33	2
14050465	3.88	4	3.39	3	3.36	3	3.54	4
14050466	1.42	1	1.79	2	1.57	2	1.59	2
14050469	1.20	1	1.26	1	0.95	1	1.13	1
14050470	1.37	1	1.34	1	1.36	1	1.36	1
14050471	1.30	1	1.42	1	1.10	1	1.27	1
14050472	1.37	1	1.22	1	1.43	1	1.34	1
14050473	1.14	1	1.26	1	1.39	1	1.26	1
14050474	1.49	1	1.49	1	1.47	1	1.48	1
14050476	0.74	1	1.55	2	1.65	2	1.32	1
14050477	1.25	1	1.10	1	1.16	1	1.17	1
14050478	1.26	1	1.34	1	1.25	1	1.28	1

Sample ID	Copy Number							
	r = 1		r = 2		r = 3		Average	
	Ratio (target/ref)	Estimated Copy Number	Ratio (target/ref)	Estimated Copy Number	Ratio (target/ref)	Estimated Copy Number	Ratio (target/ref)	Estimated Copy Number
14050485	1.95	2	2.10	2	2.43	2	2.16	2
14050486	1.15	1	1.21	1	1.36	1	1.24	1
14050490	1.35	1	1.46	1	1.30	1	1.37	1
14050491	1.28	1	1.38	1	1.25	1	1.30	1
14050496	2.78	3	2.67	3	2.91	3	2.78	3
14050497	2.17	2	2.17	2	1.78	2	2.04	2
14050498	4.38	4	3.86	4	4.38	4	4.21	4
14050499	3.96	4	3.75	4	4.29	4	4.00	4
14050500	3.24	3	3.23	3	2.89	3	3.12	3
14050501	3.15	3	2.97	3	3.12	3	3.08	3
14050502	7.52	8	7.34	7	7.92	8	7.60	8
14050503	1.38	1	1.30	1	1.26	1	1.32	1
14050504	1.55	2	1.30	1	1.19	1	1.35	1
14050505	3.66	4	3.54	4	3.67	4	3.62	4
14050506	5.71	6	5.45	5	5.37	5	5.51	6
14050507	2.00	2	1.91	2	2.31	2	2.07	2
14050508	1.32	1	1.26	1	1.19	1	1.26	1
14050522	2.96	3	3.19	3	2.73	3	2.96	3
14050528	23.54	24	21.78	22	22.69	23	22.67	23

Sample ID	Copy Number							
	r = 1		r = 2		r = 3		Average	
	Ratio (target/ref)	Estimated Copy Number	Ratio (target/ref)	Estimated Copy Number	Ratio (target/ref)	Estimated Copy Number	Ratio (target/ref)	Estimated Copy Number
14050531	2.21	2	2.08	2	2.11	2	2.13	2
14050532	4.80	5	5.18	5	4.54	5	4.84	5
14050535	1.70	2	1.85	2	1.66	2	1.74	2
14050541	1.17	1	1.09	1	1.22	1	1.16	1
14050542	2.05	2	2.15	2	2.22	2	2.14	2
14050543	1.26	1	1.26	1	1.17	1	1.23	1
14050545	4.18	4	4.44	4	4.04	4	4.22	4
14050546	4.88	5	4.49	4	5.11	5	4.83	5
14050547	1.16	1	1.15	1	1.23	1	1.18	1
14050548	3.34	3	3.66	4	3.39	3	3.47	3
14050551	1.05	1	1.16	1	1.27	1	1.16	1
14050552	1.87	2	1.83	2	1.98	2	1.89	2
14050553	1.97	2	2.03	2	2.23	2	2.08	2
14050554	2.04	2	1.93	2	1.85	2	1.94	2
14050555	1.99	2	1.87	2	1.93	2	1.93	2
14050556	1.98	2	1.99	2	2.09	2	2.02	2
14050559	1.91	2	1.96	2	2.26	2	2.04	2
14050560	1.88	2	1.87	2	2.16	2	1.97	2
14050563	1.92	2	2.04	2	2.11	2	2.02	2

Sample ID	Copy Number							
	r = 1		r = 2		r = 3		Average	
	Ratio (target/ref)	Estimated Copy Number	Ratio (target/ref)	Estimated Copy Number	Ratio (target/ref)	Estimated Copy Number	Ratio (target/ref)	Estimated Copy Number
14050564	3.21	3	3.34	3	3.23	3	3.26	3
14050575	3.44	3	3.28	3	3.41	3	3.38	3
14050576	3.38	3	3.11	3	3.15	3	3.21	3
14050577	3.55	4	3.01	3	3.31	3	3.29	3
14050578	3.45	3	3.47	3	3.31	3	3.41	3
14050579	2.43	2	2.49	2	2.43	2	2.45	2
14050585	2.23	2	2.20	2	2.18	2	2.20	2
14050587	4.32	4	3.90	4	4.04	4	4.09	4
14050592	2.51	3	2.42	2	2.66	3	2.53	3
14050594	9.24	9	11.64	12	10.25	10	10.37	10
14050598	2.07	2	2.02	2	2.00	2	2.03	2
14050599	5.28	5	5.59	6	5.70	6	5.52	6
14050600	6.69	7	5.76	6	5.94	6	6.13	6
14050604	1.23	1	1.18	1	1.28	1	1.23	1
14050609	1.26	1	1.29	1	1.38	1	1.31	1
14050610	1.23	1	1.25	1	1.20	1	1.23	1
14050613	2.80	3	2.74	3	2.79	3	2.78	3
14050616	0.99	1	1.20	1	1.15	1	1.11	1
14050617	1.08	1	1.12	1	1.05	1	1.08	1

Sample ID	Copy Number							
	r = 1		r = 2		r = 3		Average	
	Ratio (target/ref)	Estimated Copy Number	Ratio (target/ref)	Estimated Copy Number	Ratio (target/ref)	Estimated Copy Number	Ratio (target/ref)	Estimated Copy Number
14050618	1.23	1	1.22	1	1.15	1	1.20	1
14050619	3.72	4	3.75	4	3.15	3	3.54	4
14050620	3.72	4	3.09	3	2.72	3	3.18	3
14050623	0.98	1	1.05	1	0.99	1	1.01	1
14050625	1.09	1	1.14	1	1.18	1	1.14	1
14050630	1.86	2	1.94	2	1.88	2	1.90	2
14050631	1.85	2	1.93	2	1.97	2	1.92	2
14050632	2.35	2	2.48	2	2.47	2	2.43	2
14050634	3.07	3	3.45	3	4.25	4	3.59	4
14050635	1.99	2	1.76	2	1.82	2	1.86	2
14050640	2.70	3	2.82	3	2.84	3	2.79	3
14050642	1.14	1	1.14	1	1.23	1	1.17	1
14050643	1.22	1	1.28	1	1.44	1	1.31	1
14050644	1.16	1	1.27	1	1.22	1	1.22	1
14050649	1.12	1	1.20	1	1.35	1	1.22	1
14050655	2.97	3	3.10	3	3.27	3	3.11	3
14050657	7.33	7	8.65	9	9.05	9	8.35	8
14050662	1.85	2	2.04	2	1.78	2	1.89	2
14050664	3.72	4	3.68	4	0.00	0	2.47	2

Sample ID	Copy Number							
	r = 1		r = 2		r = 3		Average	
	Ratio (target/ref)	Estimated Copy Number	Ratio (target/ref)	Estimated Copy Number	Ratio (target/ref)	Estimated Copy Number	Ratio (target/ref)	Estimated Copy Number
14050665	3.61	4	3.87	4	3.85	4	3.78	4
14050673	4.06	4	3.59	4	4.30	4	3.98	4
14050683	1.91	2	1.89	2	1.76	2	1.86	2
14050684	1.98	2	2.01	2	1.84	2	1.95	2
14050691	3.41	3	3.51	4	3.40	3	3.44	3
14050692	4.01	4	3.98	4	3.93	4	3.97	4
14050693	1.27	1	1.15	1	1.24	1	1.22	1
14050698	3.94	4	3.77	4	4.55	5	4.09	4
14050699	1.13	1	1.29	1	1.17	1	1.20	1
14050710	4.65	5	5.28	5	5.42	5	5.11	5
14050711	7.60	8	6.96	7	6.81	7	7.13	7
14050718	3.98	4	4.13	4	3.85	4	3.99	4
14050737	4.83	5	4.42	4	4.84	5	4.70	5
14050745	1.60	2	1.83	2	1.75	2	1.72	2
14050763	1.29	1	1.51	2	1.41	1	1.40	1
14050786	1.19	1	1.34	1	1.20	1	1.24	1
14050787	1.33	1	1.23	1	1.30	1	1.28	1
14050797	1.17	1	1.18	1	1.17	1	1.18	1
14050801	2.72	3	2.41	2	2.48	2	2.54	3

Sample ID	Copy Number							
	r = 1		r = 2		r = 3		Average	
	Ratio (target/ref)	Estimated Copy Number	Ratio (target/ref)	Estimated Copy Number	Ratio (target/ref)	Estimated Copy Number	Ratio (target/ref)	Estimated Copy Number
14050802	15.69	16	14.79	15	15.60	16	15.36	15
14050804	3.58	4	3.48	3	3.61	4	3.56	4
14050805	1.34	1	1.35	1	1.41	1	1.37	1
14050806	1.14	1	1.23	1	1.38	1	1.25	1
14050810	1.21	1	1.25	1	1.32	1	1.26	1
14050814	20.12	20	21.61	22	23.50	23	21.74	22
14050815	2.41	2	2.51	3	2.60	3	2.51	3
14050817	2.95	3	2.84	3	2.69	3	2.83	3
14050824	4.24	4	4.08	4	3.67	4	4.00	4
14050825	3.98	4	3.86	4	3.62	4	3.82	4
14050826	3.81	4	3.69	4	3.52	4	3.67	4
14050830	3.77	4	3.70	4	3.32	3	3.60	4
14050831	3.65	4	3.47	3	3.23	3	3.45	3
14050833	2.29	2	2.12	2	2.05	2	2.15	2
14050835	8.00	8	7.96	8	7.29	7	7.75	8
14050836	3.83	4	3.79	4	3.83	4	3.82	4
14050840	1.93	2	1.92	2	1.87	2	1.91	2
14050841	1.69	2	1.70	2	1.75	2	1.71	2
14050845	8.74	9	7.35	7	7.04	7	7.71	8

Sample ID	Copy Number							
	r = 1		r = 2		r = 3		Average	
	Ratio (target/ref)	Estimated Copy Number	Ratio (target/ref)	Estimated Copy Number	Ratio (target/ref)	Estimated Copy Number	Ratio (target/ref)	Estimated Copy Number
14050857	1.87	2	1.70	2	1.84	2	1.80	2
14050858	1.22	1	1.14	1	1.32	1	1.23	1
14050863	2.81	3	2.85	3	2.92	3	2.86	3
14050865	1.20	1	1.30	1	1.36	1	1.28	1
14050866	1.20	1	1.14	1	1.31	1	1.22	1
14050868	2.15	2	2.13	2	1.96	2	2.08	2
14050882	1.18	1	0.98	1	1.03	1	1.06	1
14050892	1.28	1	1.14	1	1.10	1	1.17	1
14050895	3.02	3	2.70	3	2.87	3	2.86	3
14050904	0.03	0	0.04	0	0.03	0	0.04	0
14050907	1.75	2	1.50	1	1.54	2	1.60	2
14050910	2.76	3	2.81	3	2.92	3	2.83	3
14050911	2.60	3	2.36	2	3.00	3	2.65	3
14050912	2.70	3	2.65	3	2.43	2	2.59	3
14050913	2.26	2	2.51	3	2.25	2	2.34	2
14050916	1.17	1	1.01	1	1.24	1	1.14	1
14050917	2.25	2	2.12	2	1.89	2	2.09	2
14050919	6.02	6	5.66	6	5.80	6	5.83	6
14050925	2.25	2	2.57	3	2.47	2	2.43	2

Sample ID	Copy Number							
	r = 1		r = 2		r = 3		Average	
	Ratio (target/ref)	Estimated Copy Number	Ratio (target/ref)	Estimated Copy Number	Ratio (target/ref)	Estimated Copy Number	Ratio (target/ref)	Estimated Copy Number
14050930	1.39	1	1.46	1	1.39	1	1.41	1
14050932	1.99	2	1.84	2	1.75	2	1.86	2
14050934	1.44	1	1.39	1	1.34	1	1.39	1
14050942	1.32	1	1.36	1	1.00	1	1.23	1
14050943	3.21	3	3.25	3	2.88	3	3.11	3
14050948	1.20	1	1.20	1	1.19	1	1.20	1
14050950	1.18	1	1.22	1	1.10	1	1.17	1
14050959	1.20	1	1.16	1	1.24	1	1.20	1
14050970	4.20	4	4.08	4	4.38	4	4.22	4
14050973	1.36	1	1.30	1	1.43	1	1.36	1
14050974	1.38	1	1.66	2	1.49	1	1.51	2
14050977	1.17	1	1.21	1	1.25	1	1.21	1
14050981	1.14	1	1.26	1	1.12	1	1.17	1
14050982	1.14	1	1.11	1	1.04	1	1.09	1
14050993	1.15	1	1.09	1	1.02	1	1.08	1
14050994	1.87	2	1.84	2	1.92	2	1.88	2
14050997	0.54	1	0.57	1	0.55	1	0.56	1
14050999	1.25	1	1.31	1	1.23	1	1.26	1
14051000	1.44	1	1.43	1	1.26	1	1.38	1

Sample ID	Copy Number							
	r = 1		r = 2		r = 3		Average	
	Ratio (target/ref)	Estimated Copy Number	Ratio (target/ref)	Estimated Copy Number	Ratio (target/ref)	Estimated Copy Number	Ratio (target/ref)	Estimated Copy Number
14051001	2.25	2	2.30	2	2.31	2	2.29	2
14051007	1.19	1	1.17	1	1.12	1	1.16	1
14051011	35.90	36	35.30	35	33.28	33	34.83	35
14051013	3.59	4	3.49	3	3.72	4	3.60	4
14051014	4.05	4	4.15	4	4.25	4	4.15	4
14051016	3.11	3	2.95	3	3.17	3	3.08	3
14051017	4.70	5	4.99	5	4.47	4	4.72	5
14051019	2.85	3	2.71	3	2.97	3	2.85	3
14051023	1.91	2	2.19	2	2.19	2	2.09	2
14051028	1.28	1	1.49	1	1.40	1	1.39	1
14051029	1.05	1	1.10	1	1.30	1	1.15	1
14051030	0.78	1	0.80	1	0.66	1	0.75	1
14051040	1.22	1	1.21	1	1.33	1	1.25	1
14051045	2.13	2	2.14	2	2.01	2	2.09	2
14051047	6.64	7	7.38	7	6.43	6	6.82	7
14051049	1.60	2	1.52	2	1.67	2	1.60	2
14051051	1.42	1	1.47	1	1.39	1	1.42	1
14051054	4.84	5	4.34	4	3.73	4	4.30	4
14051055	4.05	4	3.99	4	3.71	4	3.92	4

Sample ID	Copy Number							
	r = 1		r = 2		r = 3		Average	
	Ratio (target/ref)	Estimated Copy Number	Ratio (target/ref)	Estimated Copy Number	Ratio (target/ref)	Estimated Copy Number	Ratio (target/ref)	Estimated Copy Number
14051056	3.86	4	4.33	4	4.41	4	4.20	4
14051058	1.11	1	1.21	1	1.30	1	1.21	1
14051062	2.88	3	2.72	3	2.76	3	2.79	3
14051063	2.74	3	2.85	3	2.51	3	2.70	3
14051065	2.53	3	2.28	2	2.37	2	2.39	2
14051068	2.49	2	2.17	2	2.41	2	2.36	2
14051070	1.47	1	1.38	1	1.43	1	1.43	1
14051074	5.17	5	6.05	6	5.99	6	5.73	6
14051077	1.17	1	1.28	1	1.56	2	1.34	1
14051080	3.38	3	3.64	4	3.89	4	3.64	4
14051081	1.31	1	1.48	1	1.60	2	1.46	1
14051084	1.38	1	1.52	2	1.36	1	1.42	1
14051085	3.42	3	3.50	3	3.43	3	3.45	3
14051090	2.56	3	2.52	3	2.37	2	2.48	2
14051092	2.36	2	2.89	3	2.53	3	2.59	3
14051098	6.03	6	6.12	6	6.40	6	6.19	6
14051104	1.49	1	1.39	1	1.38	1	1.42	1
14051105	1.45	1	1.31	1	1.35	1	1.37	1
14051106	4.47	4	3.93	4	3.60	4	4.00	4

Sample ID	Copy Number							
	r = 1		r = 2		r = 3		Average	
	Ratio (target/ref)	Estimated Copy Number	Ratio (target/ref)	Estimated Copy Number	Ratio (target/ref)	Estimated Copy Number	Ratio (target/ref)	Estimated Copy Number
14051110	2.45	2	2.39	2	2.36	2	2.40	2
14051114	3.48	3	3.30	3	2.57	3	3.11	3
14051115	2.91	3	3.39	3	3.26	3	3.19	3
14051116	3.24	3	2.85	3	2.81	3	2.97	3
14051117	5.17	5	4.55	5	3.91	4	4.54	5
14051118	4.42	4	4.90	5	3.64	4	4.32	4
14051121	4.34	4	3.98	4	3.79	4	4.04	4
14051124	1.41	1	1.37	1	1.27	1	1.35	1
14051127	18.79	19	20.82	21	17.46	17	19.02	19
14051129	2.45	2	2.54	3	2.24	2	2.41	2
14051132	4.53	5	4.67	5	4.40	4	4.53	5
14051133	1.73	2	1.52	2	1.36	1	1.54	2
14051134	1.20	1	1.26	1	1.15	1	1.20	1
14051137	1.28	1	1.26	1	1.35	1	1.30	1
14051139	2.21	2	2.20	2	2.18	2	2.20	2
14051142	4.24	4	4.30	4	4.38	4	4.31	4
14051143	4.71	5	4.50	5	4.84	5	4.69	5
14051149	1.64	2	1.69	2	1.64	2	1.66	2
14051155	2.07	2	2.15	2	1.98	2	2.07	2

Sample ID	Copy Number							
	r = 1		r = 2		r = 3		Average	
	Ratio (target/ref)	Estimated Copy Number	Ratio (target/ref)	Estimated Copy Number	Ratio (target/ref)	Estimated Copy Number	Ratio (target/ref)	Estimated Copy Number
14051157	10.27	10	10.81	11	10.24	10	10.44	10
14051160	1.10	1	1.24	1	1.25	1	1.19	1
14051161	1.18	1	1.22	1	1.23	1	1.21	1
14051165	3.47	3	4.64	5	4.54	5	4.22	4
14051175	2.84	3	2.94	3	3.61	4	3.13	3
14051177	1.36	1	1.25	1	1.21	1	1.27	1
14051178	2.00	2	2.05	2	1.73	2	1.92	2
14051188	1.76	2	2.06	2	1.97	2	1.93	2
14051191	4.09	4	4.37	4	4.24	4	4.24	4
14051192	4.45	4	4.44	4	5.07	5	4.66	5
14051198	1.90	2	2.07	2	1.85	2	1.94	2
14051199	1.92	2	2.08	2	1.97	2	1.99	2
14051202	12.58	13	11.60	12	8.70	9	10.96	11
14051203	3.15	3	3.12	3	3.17	3	3.15	3
14051207	1.23	1	1.29	1	1.42	1	1.31	1
14051215	3.55	4	4.36	4	4.09	4	4.00	4
14051217	1.28	1	1.28	1	1.39	1	1.32	1
14051218	2.36	2	2.70	3	2.19	2	2.42	2
14051222	1.13	1	1.34	1	1.14	1	1.20	1

Sample ID	Copy Number							
	r = 1		r = 2		r = 3		Average	
	Ratio (target/ref)	Estimated Copy Number	Ratio (target/ref)	Estimated Copy Number	Ratio (target/ref)	Estimated Copy Number	Ratio (target/ref)	Estimated Copy Number
14051230	1.46	1	1.25	1	1.15	1	1.29	1
14051234	1.39	1	1.30	1	1.31	1	1.33	1
14051242	2.43	2	2.25	2	2.10	2	2.26	2
14051243	2.50	2	2.49	2	2.52	3	2.50	3
14051247	1.58	2	1.29	1	1.36	1	1.41	1
14051250	1.67	2	1.64	2	1.45	1	1.58	2
14051251	4.43	4	4.46	4	4.39	4	4.43	4
14051252	5.38	5	4.84	5	4.47	4	4.90	5
14051253	0.97	1	1.23	1	1.33	1	1.18	1
14051256	1.11	1	1.13	1	1.26	1	1.17	1
14051259	1.22	1	1.32	1	1.08	1	1.20	1
14051260	1.32	1	1.34	1	1.30	1	1.32	1
14051267	10.72	11	9.82	10	10.00	10	10.18	10
14051269	2.21	2	2.39	2	2.55	3	2.38	2
14051270	2.31	2	2.39	2	2.25	2	2.32	2
14051272	2.47	2	3.00	3	3.16	3	2.87	3
14051274	3.51	4	3.42	3	3.71	4	3.54	4
14051275	4.52	5	4.83	5	5.03	5	4.79	5
14051276	1.65	2	1.72	2	1.72	2	1.70	2

Sample ID	Copy Number							
	r = 1		r = 2		r = 3		Average	
	Ratio (target/ref)	Estimated Copy Number	Ratio (target/ref)	Estimated Copy Number	Ratio (target/ref)	Estimated Copy Number	Ratio (target/ref)	Estimated Copy Number
14051279	2.55	3	2.13	2	2.32	2	2.34	2
14051280	1.54	2	1.19	1	1.05	1	1.26	1
14051282	1.13	1	1.22	1	1.15	1	1.17	1
14051283	1.11	1	1.11	1	1.32	1	1.18	1
14051286	3.12	3	3.60	4	3.87	4	3.53	4
14051288	2.16	2	2.07	2	2.13	2	2.12	2
14051293	2.25	2	2.27	2	2.27	2	2.27	2
14051295	1.24	1	1.18	1	1.13	1	1.18	1
14051300	2.27	2	2.25	2	1.89	2	2.14	2
14051301	2.01	2	2.25	2	1.99	2	2.08	2
14051302	1.22	1	1.23	1	1.13	1	1.19	1
14051310	1.46	1	1.37	1	1.24	1	1.35	1
14051327	1.39	1	1.33	1	1.42	1	1.38	1
14051331	3.64	4	3.73	4	3.81	4	3.73	4
14051333	6.88	7	7.79	8	8.08	8	7.58	8
14051338	0.48	0	0.53	1	0.44	0	0.48	0
14051350	4.98	5	5.02	5	4.88	5	4.96	5
14051352	1.34	1	1.21	1	1.37	1	1.30	1
14051353	2.26	2	2.11	2	2.01	2	2.13	2

Sample ID	Copy Number							
	r = 1		r = 2		r = 3		Average	
	Ratio (target/ref)	Estimated Copy Number	Ratio (target/ref)	Estimated Copy Number	Ratio (target/ref)	Estimated Copy Number	Ratio (target/ref)	Estimated Copy Number
14051356	1.43	1	1.42	1	1.45	1	1.43	1
14051382	3.11	3	2.75	3	3.55	4	3.14	3
14051385	1.33	1	1.45	1	1.43	1	1.40	1
14051386	1.32	1	1.26	1	1.53	2	1.37	1
14051390	2.02	2	1.96	2	2.24	2	2.07	2
14051392	1.88	2	2.18	2	2.44	2	2.17	2
14051394	0.98	1	1.17	1	0.91	1	1.02	1
14051396	1.94	2	1.93	2	1.72	2	1.86	2
14051402	1.14	1	1.16	1	1.07	1	1.12	1
14051406	2.67	3	2.54	3	2.10	2	2.44	2
14051430	9.87	10	8.81	9	8.62	9	9.10	9
14051437	4.15	4	3.85	4	4.26	4	4.08	4
14051455	1.41	1	1.14	1	1.31	1	1.28	1
14051456	1.40	1	1.40	1	1.29	1	1.36	1
14051458	3.52	4	3.03	3	3.34	3	3.30	3
14051477	2.88	3	2.56	3	2.28	2	2.58	3
14051480	1.23	1	1.23	1	1.19	1	1.22	1
14051483	2.35	2	2.28	2	2.09	2	2.24	2
14051484	3.68	4	3.68	4	3.47	3	3.61	4

Sample ID	Copy Number							
	r = 1		r = 2		r = 3		Average	
	Ratio (target/ref)	Estimated Copy Number	Ratio (target/ref)	Estimated Copy Number	Ratio (target/ref)	Estimated Copy Number	Ratio (target/ref)	Estimated Copy Number
14051486	2.70	3	2.30	2	2.47	2	2.49	2
14051489	17.87	18	15.89	16	14.52	15	16.09	16
14051491	1.28	1	1.24	1	1.20	1	1.24	1
14051492	2.54	3	2.51	3	2.31	2	2.45	2
14051493	1.57	2	1.88	2	1.81	2	1.75	2
14051495	4.60	5	5.30	5	5.22	5	5.04	5
14051496	5.47	5	5.54	6	5.91	6	5.64	6
14150001	5.36	5	5.08	5	4.62	5	5.02	5
14180006	1.71	2	1.87	2	1.58	2	1.72	2
14180007	1.85	2	1.70	2	1.77	2	1.77	2
14180008	1.99	2	1.46	1	1.66	2	1.70	2
14180009	1.82	2	1.80	2	1.79	2	1.80	2
14180011	1.91	2	1.61	2	1.95	2	1.82	2
14180012	2.15	2	2.44	2	2.24	2	2.28	2
14720170	3.57	4	3.47	3	3.50	3	3.51	4
14720329	1.33	1	1.36	1	1.11	1	1.27	1
14730039	3.57	4	2.90	3	2.65	3	3.04	3
14730119	0.29	0	0.25	0	0.23	0	0.25	0
14730167	1.41	1	1.21	1	1.43	1	1.35	1

Sample ID	Copy Number							
	r = 1		r = 2		r = 3		Average	
	Ratio (target/ref)	Estimated Copy Number	Ratio (target/ref)	Estimated Copy Number	Ratio (target/ref)	Estimated Copy Number	Ratio (target/ref)	Estimated Copy Number
14730169	0.02	0	0.03	0	0.04	0	0.03	0
14730175	2.33	2	2.37	2	2.16	2	2.29	2
14730177	2.24	2	2.45	2	2.39	2	2.36	2
14730181	1.54	2	1.59	2	1.19	1	1.44	1
14730183	1.43	1	1.34	1	1.27	1	1.35	1
14740011	1.37	1	1.34	1	1.14	1	1.28	1
14740038	1.26	1	1.38	1	1.21	1	1.28	1
14740055	2.52	3	2.56	3	3.07	3	2.72	3
14740074	1.31	1	1.37	1	1.24	1	1.30	1
14740077	1.37	1	1.45	1	1.44	1	1.42	1
14740085	1.22	1	1.32	1	1.30	1	1.28	1
14740092	1.48	1	1.44	1	1.26	1	1.40	1
14740099	1.27	1	1.42	1	1.50	1	1.40	1
14740167	1.13	1	1.06	1	1.17	1	1.12	1
14740189	2.99	3	2.98	3	2.74	3	2.90	3
14740229	1.49	1	1.63	2	1.46	1	1.53	2
14750031	2.25	2	1.94	2	2.19	2	2.13	2
14750037	1.92	2	1.86	2	1.67	2	1.82	2
14750045	2.59	3	2.76	3	2.92	3	2.76	3

Sample ID	Copy Number							
	r = 1		r = 2		r = 3		Average	
	Ratio (target/ref)	Estimated Copy Number	Ratio (target/ref)	Estimated Copy Number	Ratio (target/ref)	Estimated Copy Number	Ratio (target/ref)	Estimated Copy Number
14750083	1.44	1	2.03	2	1.70	2	1.72	2
14750093	2.42	2	1.98	2	2.23	2	2.21	2
14750121	0.28	0	0.28	0	0.32	0	0.30	0
14750127	1.48	1	1.35	1	1.12	1	1.31	1
14750141	1.69	2	1.84	2	1.95	2	1.83	2
14750164	1.52	2	1.36	1	1.26	1	1.38	1
14750190	1.12	1	1.24	1	1.13	1	1.16	1
14760025	2.27	2	2.30	2	1.86	2	2.14	2
14760031	1.66	2	1.60	2	1.57	2	1.61	2
14760044	1.61	2	2.03	2	1.74	2	1.79	2
14760054	5.41	5	5.71	6	5.04	5	5.39	5
14760058	0.50	0	0.54	1	0.61	1	0.55	1
14760061	2.83	3	2.27	2	2.50	2	2.53	3
14760064	1.85	2	1.97	2	2.11	2	1.97	2
14760065	1.12	1	1.29	1	1.23	1	1.22	1
14760077	2.08	2	1.94	2	2.17	2	2.06	2
14760079	0.94	1	0.95	1	0.88	1	0.92	1
14760085	1.94	2	2.29	2	1.94	2	2.06	2
14760090	3.25	3	2.80	3	3.30	3	3.11	3

Sample ID	Copy Number							
	r = 1		r = 2		r = 3		Average	
	Ratio (target/ref)	Estimated Copy Number	Ratio (target/ref)	Estimated Copy Number	Ratio (target/ref)	Estimated Copy Number	Ratio (target/ref)	Estimated Copy Number
14760091	1.17	1	1.34	1	1.26	1	1.26	1
14760092	2.06	2	2.08	2	2.05	2	2.06	2
14760096	2.49	2	1.96	2	2.00	2	2.15	2
14760097	4.16	4	3.72	4	4.56	5	4.15	4
14760099	1.29	1	1.22	1	1.28	1	1.26	1
14770002	2.88	3	3.23	3	3.42	3	3.18	3
14770015	1.80	2	2.13	2	1.89	2	1.94	2
14770018	3.64	4	2.47	2	2.87	3	2.99	3
14770039	1.06	1	1.10	1	0.94	1	1.04	1
14770048	3.17	3	2.82	3	2.93	3	2.98	3
14770056	2.54	3	0.16	0	2.18	2	1.63	2
14770057	1.03	1	1.13	1	0.96	1	1.04	1
14770062	3.49	3	3.12	3	2.69	3	3.10	3
14770070	0.89	1	0.92	1	0.96	1	0.92	1
14770075	3.80	4	2.46	2	3.14	3	3.13	3
14770087	4.24	4	2.40	2	2.26	2	2.96	3
14770098	2.71	3	2.93	3	1.71	2	2.45	2
14770103	2.92	3	3.02	3	2.73	3	2.89	3
14770114	3.44	3	3.13	3	3.28	3	3.28	3

Sample ID	Copy Number							
	r = 1		r = 2		r = 3		Average	
	Ratio (target/ref)	Estimated Copy Number	Ratio (target/ref)	Estimated Copy Number	Ratio (target/ref)	Estimated Copy Number	Ratio (target/ref)	Estimated Copy Number
14770127	2.56	3	2.57	3	2.59	3	2.57	3
14770169	3.90	4	3.35	3	3.52	4	3.59	4
14770195	2.34	2	2.25	2	2.23	2	2.27	2
14770204	1.11	1	1.04	1	0.83	1	0.99	1
14770215	2.09	2	2.20	2	1.93	2	2.07	2
14770222	1.16	1	1.22	1	1.19	1	1.19	1
14770238	2.56	3	2.50	2	2.45	2	2.50	3
14770252	2.45	2	2.21	2	2.16	2	2.27	2
14770255	1.97	2	2.10	2	2.44	2	2.17	2
14770270	1.73	2	2.02	2	1.93	2	1.90	2
14770301	2.09	2	2.14	2	1.67	2	1.96	2
14770306	1.38	1	1.22	1	1.24	1	1.28	1
14770311	1.41	1	1.43	1	1.71	2	1.52	2
14770320	1.33	1	1.52	2	1.48	1	1.44	1
14770329	1.71	2	1.25	1	1.86	2	1.61	2
14770331	2.40	2	2.01	2	2.75	3	2.39	2
14770343	1.07	1	0.98	1	0.97	1	1.01	1
14770350	1.83	2	1.83	2	2.34	2	2.00	2
14770390	3.29	3	4.19	4	2.91	3	3.46	3

Sample ID	Copy Number							
	r = 1		r = 2		r = 3		Average	
	Ratio (target/ref)	Estimated Copy Number	Ratio (target/ref)	Estimated Copy Number	Ratio (target/ref)	Estimated Copy Number	Ratio (target/ref)	Estimated Copy Number
14770395	1.69	2	1.60	2	2.03	2	1.77	2
14770405	1.08	1	1.04	1	1.20	1	1.11	1
14770408	1.15	1	1.11	1	1.23	1	1.16	1
14770409	2.08	2	2.26	2	1.58	2	1.97	2
14770429	1.30	1	1.32	1	1.53	2	1.38	1
14770439	1.74	2	2.35	2	2.28	2	2.12	2
14770440	0.96	1	1.20	1	1.37	1	1.18	1
14770443	1.10	1	1.15	1	1.43	1	1.22	1
14780012	1.89	2	2.04	2	2.27	2	2.07	2
14780013	1.81	2	1.92	2	2.36	2	2.03	2
14780021	8.60	9	12.12	12	7.82	8	9.51	10
14780036	3.26	3	3.01	3	3.12	3	3.13	3
14780039	2.90	3	3.72	4	4.19	4	3.60	4
14780040	1.22	1	1.43	1	1.34	1	1.33	1
14780050	1.51	2	1.97	2	1.68	2	1.72	2
14780053	1.19	1	0.75	1	1.20	1	1.05	1
14780056	1.63	2	1.64	2	1.59	2	1.62	2
14780058	1.00	1	1.13	1	1.17	1	1.10	1
14780066	2.84	3	3.07	3	3.68	4	3.20	3

Sample ID	Copy Number							
	r = 1		r = 2		r = 3		Average	
	Ratio (target/ref)	Estimated Copy Number	Ratio (target/ref)	Estimated Copy Number	Ratio (target/ref)	Estimated Copy Number	Ratio (target/ref)	Estimated Copy Number
14780072	1.09	1	1.36	1	1.08	1	1.18	1
14780075	2.72	3	2.42	2	2.68	3	2.61	3
14780076	1.49	1	1.62	2	1.45	1	1.52	2
14780080	1.21	1	1.33	1	1.35	1	1.30	1
14780089	1.90	2	3.90	4	2.06	2	2.62	3
14780090	1.11	1	1.21	1	1.32	1	1.21	1
14780091	1.07	1	1.47	1	1.19	1	1.25	1
14780096	1.09	1	1.14	1	1.29	1	1.17	1
14780107	0.98	1	0.89	1	1.37	1	1.08	1
14780110	0.81	1	0.74	1	2.06	2	1.20	1
14780126	1.57	2	1.06	1	1.29	1	1.31	1
14780137	2.41	2	2.43	2	2.18	2	2.34	2
14780145	3.95	4	3.40	3	3.61	4	3.65	4
14780150	0.43	0	2.36	2	2.63	3	1.81	2
14780159	1.17	1	1.24	1	1.21	1	1.20	1
14780160	1.56	2	1.42	1	7.25	7	3.41	3
14780163	1.82	2	1.85	2	2.07	2	1.91	2
14780164	1.31	1	0.95	1	1.13	1	1.13	1
14780171	1.13	1	1.14	1	1.36	1	1.21	1

Sample ID	Copy Number							
	r = 1		r = 2		r = 3		Average	
	Ratio (target/ref)	Estimated Copy Number	Ratio (target/ref)	Estimated Copy Number	Ratio (target/ref)	Estimated Copy Number	Ratio (target/ref)	Estimated Copy Number
14780195	1.20	1	1.13	1	1.14	1	1.16	1
14780203	1.33	1	1.36	1	1.41	1	1.37	1
14780219	0.36	0	0.34	0	0.36	0	0.36	0
14780221	2.80	3	2.78	3	2.69	3	2.76	3
14780224	2.97	3	2.42	2	1.20	1	2.20	2
14790014	1.44	1	1.39	1	1.39	1	1.41	1
14790022	1.94	2	1.71	2	1.57	2	1.74	2
14790028	1.94	2	1.80	2	1.90	2	1.88	2
14790034	1.34	1	1.04	1	1.07	1	1.15	1
14790068	1.49	1	1.46	1	1.40	1	1.45	1
14790082	1.02	1	1.11	1	0.97	1	1.03	1
14790103	1.20	1	1.10	1	1.28	1	1.19	1
14790104	62.19	62	58.54	59	37.63	38	52.78	53
14790110	1.01	1	1.14	1	1.13	1	1.09	1
14790138	2.28	2	2.13	2	2.22	2	2.21	2
14790143	0.88	1	1.13	1	0.83	1	0.95	1
14790144	2.32	2	2.10	2	2.13	2	2.18	2
14790156	1.09	1	1.09	1	1.07	1	1.08	1
14790177	1.33	1	1.80	2	1.26	1	1.46	1

Sample ID	Copy Number							
	r = 1		r = 2		r = 3		Average	
	Ratio (target/ref)	Estimated Copy Number	Ratio (target/ref)	Estimated Copy Number	Ratio (target/ref)	Estimated Copy Number	Ratio (target/ref)	Estimated Copy Number
14790220	1.18	1	1.08	1	1.36	1	1.20	1
14790221	1.15	1	1.07	1	1.02	1	1.08	1
14790222	1.12	1	1.15	1	1.16	1	1.14	1
14790224	2.99	3	3.10	3	2.87	3	2.98	3
14790226	1.92	2	1.95	2	1.78	2	1.88	2
14790235	2.89	3	2.92	3	2.77	3	2.86	3
14790245	2.57	3	2.64	3	2.59	3	2.60	3
14790290	1.06	1	1.15	1	0.99	1	1.06	1
14790292	1.98	2	2.04	2	2.30	2	2.10	2
14790302	1.60	2	1.56	2	1.34	1	1.50	1
14790313	2.66	3	2.60	3	2.31	2	2.52	3
14790324	1.33	1	1.36	1	1.26	1	1.32	1
14790331	1.37	1	1.42	1	1.40	1	1.39	1
14790336	2.12	2	1.84	2	1.81	2	1.92	2
14790342	1.79	2	2.12	2	2.19	2	2.03	2
14790343	1.26	1	1.22	1	0.83	1	1.10	1
14790348	2.26	2	2.23	2	2.17	2	2.22	2
14790349	2.34	2	2.14	2	2.20	2	2.22	2
14790359	2.58	3	2.60	3	2.65	3	2.61	3

Sample ID	Copy Number							
	r = 1		r = 2		r = 3		Average	
	Ratio (target/ref)	Estimated Copy Number	Ratio (target/ref)	Estimated Copy Number	Ratio (target/ref)	Estimated Copy Number	Ratio (target/ref)	Estimated Copy Number
14790364	1.13	1	1.11	1	0.97	1	1.07	1
14790365	1.16	1	1.23	1	1.20	1	1.20	1
14790368	0.18	0	0.21	0	0.23	0	0.21	0
14790369	2.72	3	2.69	3	3.74	4	3.05	3
14790372	1.16	1	1.13	1	1.09	1	1.13	1
14790373	1.21	1	1.09	1	0.98	1	1.09	1
14790375	1.38	1	1.39	1	1.35	1	1.37	1
14790378	1.66	2	1.82	2	1.81	2	1.77	2
14790381	1.05	1	1.11	1	1.07	1	1.08	1
14790382	1.01	1	0.98	1	1.05	1	1.01	1
14790388	1.29	1	1.41	1	1.53	2	1.41	1
14790390	1.55	2	1.24	1	1.43	1	1.40	1
14790393	0.34	0	1.77	2	1.22	1	1.11	1
14790394	1.71	2	1.17	1	1.60	2	1.49	1
14790395	2.19	2	2.16	2	1.50	2	1.95	2
14790396	1.47	1	1.37	1	1.43	1	1.42	1
14790398	1.30	1	1.26	1	1.21	1	1.26	1
14790404	1.35	1	1.30	1	1.28	1	1.31	1
14790406	1.60	2	1.47	1	1.40	1	1.49	1

Sample ID	Copy Number							
	r = 1		r = 2		r = 3		Average	
	Ratio (target/ref)	Estimated Copy Number	Ratio (target/ref)	Estimated Copy Number	Ratio (target/ref)	Estimated Copy Number	Ratio (target/ref)	Estimated Copy Number
14790408	1.45	1	1.69	2	1.56	2	1.57	2
14790410	3.24	3	3.29	3	3.30	3	3.28	3
14790411	2.75	3	3.11	3	3.20	3	3.02	3
14790412	2.09	2	2.17	2	2.41	2	2.22	2
14790413	2.20	2	2.37	2	2.33	2	2.30	2
14790414	2.19	2	2.06	2	1.93	2	2.06	2
14790416	1.31	1	1.41	1	1.48	1	1.40	1
14790422	2.89	3	2.82	3	2.76	3	2.83	3
14790423	2.06	2	2.21	2	2.13	2	2.13	2
14790444	1.04	1	1.14	1	1.06	1	1.08	1
14790445	2.32	2	2.27	2	2.09	2	2.23	2
14790460	10.55	11	11.62	12	9.84	10	10.67	11
14790463	1.16	1	1.10	1	1.27	1	1.18	1
14790466	0.94	1	1.02	1	0.95	1	0.97	1
14790483	1.28	1	1.23	1	1.22	1	1.24	1
14790495	1.68	2	1.42	1	1.17	1	1.43	1
14790496	2.58	3	2.44	2	2.14	2	2.39	2
14790497	1.92	2	2.18	2	1.85	2	1.98	2
14790498	1.09	1	1.31	1	1.13	1	1.17	1

Sample ID	Copy Number							
	r = 1		r = 2		r = 3		Average	
	Ratio (target/ref)	Estimated Copy Number	Ratio (target/ref)	Estimated Copy Number	Ratio (target/ref)	Estimated Copy Number	Ratio (target/ref)	Estimated Copy Number
14790500	1.03	1	1.35	1	1.04	1	1.14	1
14790502	5.97	6	5.21	5	4.88	5	5.36	5
14790503	0.88	1	1.10	1	1.28	1	1.09	1
14790504	0.94	1	1.14	1	1.04	1	1.04	1
14790505	0.48	0	0.53	1	0.47	0	0.49	0
14790506	1.89	2	1.99	2	1.67	2	1.85	2
14790508	2.78	3	2.93	3	2.46	2	2.72	3
14790511	1.40	1	1.27	1	1.23	1	1.30	1
14790512	2.12	2	2.21	2	2.06	2	2.13	2
14790513	2.07	2	1.65	2	1.68	2	1.80	2
14790515	2.99	3	2.88	3	2.98	3	2.95	3
14790517	2.24	2	2.06	2	2.12	2	2.14	2
14790518	1.92	2	1.96	2	1.88	2	1.92	2
14790519	1.05	1	0.83	1	0.97	1	0.95	1
14790520	1.54	2	1.90	2	1.91	2	1.79	2
14790521	0.91	1	1.11	1	1.27	1	1.10	1
14790523	1.63	2	1.84	2	1.58	2	1.69	2
14790528	1.22	1	1.00	1	1.18	1	1.13	1
14790529	1.52	2	1.62	2	1.58	2	1.58	2

Sample ID	Copy Number							
	r = 1		r = 2		r = 3		Average	
	Ratio (target/ref)	Estimated Copy Number	Ratio (target/ref)	Estimated Copy Number	Ratio (target/ref)	Estimated Copy Number	Ratio (target/ref)	Estimated Copy Number
14790530	2.00	2	1.66	2	2.05	2	1.91	2
14790534	1.92	2	2.17	2	1.91	2	2.00	2
14790535	0.96	1	1.14	1	0.91	1	1.00	1
14790543	4.29	4	4.61	5	4.66	5	4.52	5
14790554	0.95	1	0.91	1	0.95	1	0.94	1
14790556	1.08	1	1.30	1	0.65	1	1.01	1
14790561	3.83	4	4.35	4	5.33	5	4.50	5
14790574	0.92	1	0.96	1	1.29	1	1.06	1
14790575	1.29	1	1.13	1	1.00	1	1.14	1
14790577	8.01	8	9.57	10	8.48	8	8.69	9
14790578	7.46	7	9.47	9	8.86	9	8.60	9
14790580	1.45	1	1.43	1	1.41	1	1.43	1
14790581	2.16	2	2.04	2	2.09	2	2.10	2
14790583	1.82	2	2.67	3	1.97	2	2.15	2
14790590	2.70	3	2.80	3	2.96	3	2.82	3
14790591	2.35	2	2.28	2	2.26	2	2.30	2
14790594	1.57	2	1.42	1	1.98	2	1.66	2
14790595	0.93	1	1.15	1	1.01	1	1.03	1
14790597	1.98	2	2.45	2	1.98	2	2.14	2

Sample ID	Copy Number							
	r = 1		r = 2		r = 3		Average	
	Ratio (target/ref)	Estimated Copy Number	Ratio (target/ref)	Estimated Copy Number	Ratio (target/ref)	Estimated Copy Number	Ratio (target/ref)	Estimated Copy Number
14790601	1.23	1	1.20	1	1.28	1	1.24	1
14790605	1.24	1	1.04	1	1.32	1	1.20	1
14790612	0.38	0	0.46	0	0.46	0	0.43	0
14790615	0.95	1	1.09	1	1.31	1	1.12	1
14790619	0.70	1	0.76	1	0.75	1	0.74	1
14790629	0.52	1	0.52	1	0.49	0	0.51	1
14790630	1.95	2	2.09	2	2.03	2	2.03	2
14790632	0.94	1	0.99	1	1.07	1	1.00	1
14790633	1.28	1	1.10	1	1.00	1	1.13	1
14790634	1.41	1	1.58	2	1.55	2	1.52	2
14790635	1.77	2	1.76	2	1.80	2	1.78	2
14790637	1.61	2	1.41	1	1.48	1	1.50	2
14790638	2.11	2	2.49	2	2.25	2	2.29	2
14790640	1.57	2	1.66	2	1.71	2	1.65	2
14790641	1.75	2	1.96	2	1.97	2	1.89	2
14790642	2.24	2	2.62	3	2.63	3	2.50	2
14790643	8.87	9	8.75	9	10.47	10	9.36	9
14790645	1.51	2	1.48	1	1.38	1	1.45	1
14790646	1.50	2	1.41	1	1.37	1	1.43	1

Sample ID	Copy Number							
	r = 1		r = 2		r = 3		Average	
	Ratio (target/ref)	Estimated Copy Number	Ratio (target/ref)	Estimated Copy Number	Ratio (target/ref)	Estimated Copy Number	Ratio (target/ref)	Estimated Copy Number
14790647	3.11	3	3.05	3	3.11	3	3.09	3
14790648	1.56	2	1.38	1	1.54	2	1.49	1
14790649	3.34	3	3.45	3	3.42	3	3.40	3
14790650	6.85	7	6.53	7	5.99	6	6.46	6
14790655	1.37	1	1.00	1	1.35	1	1.24	1
14790658	3.45	3	3.14	3	3.20	3	3.26	3
14790661	1.23	1	1.08	1	1.12	1	1.14	1
14790662	2.13	2	1.57	2	1.28	1	1.66	2
14790664	0.95	1	1.30	1	1.21	1	1.15	1
14790667	6.39	6	5.64	6	5.44	5	5.82	6
14790670	2.74	3	2.00	2	2.45	2	2.40	2
14790672	1.33	1	1.55	2	1.55	2	1.48	1
14790673	2.15	2	2.53	3	2.47	2	2.39	2
14790675	2.13	2	2.10	2	2.08	2	2.10	2
14790676	19.90	20	18.06	18	16.93	17	18.30	18
14790677	2.65	3	2.47	2	2.30	2	2.47	2
14790678	5.13	5	4.81	5	5.78	6	5.24	5
14790679	2.61	3	3.54	4	2.98	3	3.04	3
14790680	1.77	2	1.77	2	1.90	2	1.81	2

Sample ID	Copy Number							
	r = 1		r = 2		r = 3		Average	
	Ratio (target/ref)	Estimated Copy Number	Ratio (target/ref)	Estimated Copy Number	Ratio (target/ref)	Estimated Copy Number	Ratio (target/ref)	Estimated Copy Number
14790682	1.56	2	1.53	2	1.32	1	1.47	1
14790684	2.52	3	2.09	2	2.00	2	2.20	2
14790685	4.22	4	3.83	4	3.83	4	3.96	4
14790686	1.15	1	0.07	0	1.13	1	0.78	1
14790687	1.12	1	1.17	1	0.97	1	1.08	1
14790689	2.28	2	2.35	2	2.09	2	2.24	2
14790691	1.94	2	2.09	2	2.48	2	2.17	2
14790692	4.22	4	3.69	4	5.29	5	4.40	4
14790696	2.02	2	1.87	2	2.01	2	1.97	2
14790698	1.73	2	1.10	1	1.70	2	1.51	2
14790699	1.49	1	1.63	2	1.41	1	1.51	2
14790700	1.33	1	1.30	1	1.27	1	1.30	1
14790701	0.66	1	0.63	1	0.57	1	0.62	1
14790702	0.79	1	0.84	1	0.71	1	0.78	1
14790704	1.14	1	1.09	1	1.04	1	1.09	1
14790705	1.88	2	1.92	2	1.83	2	1.88	2
14790712	3.02	3	2.70	3	2.74	3	2.82	3
14790715	0.41	0	0.40	0	0.48	0	0.43	0
14790716	2.55	3	2.64	3	2.68	3	2.62	3

Sample ID	Copy Number							
	r = 1		r = 2		r = 3		Average	
	Ratio (target/ref)	Estimated Copy Number	Ratio (target/ref)	Estimated Copy Number	Ratio (target/ref)	Estimated Copy Number	Ratio (target/ref)	Estimated Copy Number
14790717	1.99	2	2.24	2	1.91	2	2.05	2
14790718	2.44	2	2.44	2	2.18	2	2.36	2
14790719	2.71	3	2.66	3	2.61	3	2.66	3
14790720	2.82	3	2.65	3	2.64	3	2.70	3
14790721	2.90	3	2.93	3	2.87	3	2.90	3
14790722	2.72	3	2.86	3	2.76	3	2.78	3
14790723	1.11	1	1.12	1	1.10	1	1.11	1
14790730	1.04	1	1.00	1	1.07	1	1.04	1
14790736	2.18	2	2.32	2	2.32	2	2.27	2
14790738	2.36	2	2.20	2	2.15	2	2.24	2
14790740	4.42	4	5.20	5	5.73	6	5.12	5
14790741	5.32	5	5.59	6	6.60	7	5.84	6
14790743	1.17	1	1.18	1	1.06	1	1.14	1
14790744	1.07	1	1.29	1	1.07	1	1.14	1
14790745	1.13	1	1.06	1	1.03	1	1.07	1
14790746	1.89	2	1.79	2	1.65	2	1.77	2
14790747	2.03	2	2.32	2	2.11	2	2.15	2
14790748	3.15	3	3.07	3	3.12	3	3.11	3
14790749	2.61	3	2.69	3	2.66	3	2.65	3

Sample ID	Copy Number							
	r = 1		r = 2		r = 3		Average	
	Ratio (target/ref)	Estimated Copy Number	Ratio (target/ref)	Estimated Copy Number	Ratio (target/ref)	Estimated Copy Number	Ratio (target/ref)	Estimated Copy Number
14790750	2.67	3	2.60	3	2.75	3	2.67	3
14790751	3.82	4	4.04	4	4.72	5	4.19	4
14790754	1.48	1	2.00	2	1.68	2	1.72	2
14790755	3.64	4	3.96	4	3.41	3	3.67	4
14790757	1.79	2	1.49	1	1.58	2	1.62	2
14790758	1.07	1	1.55	2	1.44	1	1.36	1
14790759	3.65	4	3.94	4	3.78	4	3.79	4
14790760	3.18	3	3.70	4	3.13	3	3.33	3
14790761	1.93	2	2.68	3	1.97	2	2.19	2
14790762	5.18	5	5.54	6	5.33	5	5.35	5
14790763	3.41	3	3.83	4	3.77	4	3.67	4
14790764	3.23	3	3.25	3	3.49	3	3.32	3
14790765	3.12	3	3.24	3	3.58	4	3.31	3
14790766	1.47	1	1.37	1	1.51	2	1.45	1
14790767	1.46	1	1.36	1	1.63	2	1.49	1
14790768	1.38	1	0.05	0	1.84	2	1.09	1
14790769	1.80	2	1.86	2	1.43	1	1.70	2
14790770	3.75	4	3.51	4	4.45	4	3.90	4
14790771	2.79	3	3.13	3	2.79	3	2.90	3

Sample ID	Copy Number							
	r = 1		r = 2		r = 3		Average	
	Ratio (target/ref)	Estimated Copy Number	Ratio (target/ref)	Estimated Copy Number	Ratio (target/ref)	Estimated Copy Number	Ratio (target/ref)	Estimated Copy Number
14790773	1.78	2	1.93	2	1.85	2	1.85	2
14790774	4.80	5	4.83	5	6.03	6	5.22	5
14790775	5.08	5	5.97	6	6.62	7	5.89	6
14790779	4.99	5	4.91	5	4.44	4	4.78	5
14790780	2.51	3	2.40	2	1.84	2	2.25	2
14790781	4.12	4	3.58	4	3.72	4	3.81	4
14790785	1.59	2	1.83	2	1.67	2	1.70	2
14790790	2.63	3	2.32	2	2.53	3	2.49	2
14790792	2.95	3	2.61	3	2.42	2	2.66	3
14790794	3.93	4	3.69	4	4.60	5	4.07	4
14790797	7.34	7	3.64	4	3.00	3	4.66	5
14790800	3.64	4	3.10	3	3.38	3	3.37	3
14790803	3.11	3	2.48	2	2.74	3	2.77	3
14790804	2.16	2	3.70	4	2.49	2	2.78	3
14790805	2.86	3	1.77	2	3.27	3	2.64	3
14790808	1.54	2	1.86	2	1.40	1	1.60	2
14790809	1.26	1	1.22	1	1.47	1	1.32	1
14790810	2.48	2	2.59	3	1.57	2	2.21	2
14790812	0.38	0	0.45	0	0.50	1	0.45	0

Sample ID	Copy Number							
	r = 1		r = 2		r = 3		Average	
	Ratio (target/ref)	Estimated Copy Number	Ratio (target/ref)	Estimated Copy Number	Ratio (target/ref)	Estimated Copy Number	Ratio (target/ref)	Estimated Copy Number
14790814	3.24	3	2.92	3	3.04	3	3.06	3
14790815	2.02	2	1.96	2	1.98	2	1.99	2
14790817	0.52	1	0.58	1	0.55	1	0.55	1
14790819	2.70	3	2.18	2	2.17	2	2.35	2
14790821	1.49	1	0.86	1	1.30	1	1.22	1
14790824	1.26	1	1.19	1	1.22	1	1.22	1
14790825	2.07	2	2.10	2	1.88	2	2.02	2
14790826	1.35	1	1.04	1	1.01	1	1.13	1
14790829	7.47	7	4.71	5	6.24	6	6.14	6
14790830	13.03	13	8.93	9	9.86	10	10.61	11
14790833	1.43	1	1.44	1	1.25	1	1.37	1
14790834	0.87	1	0.94	1	0.76	1	0.86	1
14790835	1.36	1	1.39	1	1.38	1	1.38	1
14790836	1.16	1	1.14	1	1.21	1	1.17	1
14790838	1.12	1	1.34	1	0.82	1	1.09	1
14790840	3.11	3	3.22	3	2.73	3	3.02	3
14790841	3.20	3	3.32	3	3.41	3	3.31	3
14790843	2.59	3	2.51	3	2.47	2	2.53	3
14790844	1.05	1	0.70	1	0.93	1	0.89	1

Sample ID	Copy Number							
	r = 1		r = 2		r = 3		Average	
	Ratio (target/ref)	Estimated Copy Number	Ratio (target/ref)	Estimated Copy Number	Ratio (target/ref)	Estimated Copy Number	Ratio (target/ref)	Estimated Copy Number
14790845	3.25	3	3.34	3	3.02	3	3.20	3
14790847	9.01	9	10.46	10	10.66	11	10.04	10
14790848	1.12	1	0.97	1	0.96	1	1.01	1
14790849	6.49	6	6.62	7	6.58	7	6.56	7
14790851	3.36	3	3.43	3	3.37	3	3.39	3
14790854	3.37	3	3.56	4	3.25	3	3.39	3
14790855	1.61	2	2.10	2	1.70	2	1.80	2
14790857	2.39	2	2.34	2	3.12	3	2.62	3
14790859	1.25	1	1.28	1	1.22	1	1.25	1
14790862	1.39	1	1.52	2	1.86	2	1.59	2
14790863	3.25	3	5.34	5	5.70	6	4.76	5
14790864	1.25	1	1.13	1	1.16	1	1.18	1
14790867	3.03	3	3.32	3	3.10	3	3.15	3
14790869	4.77	5	5.56	6	6.00	6	5.44	5
14790871	1.45	1	1.46	1	2.00	2	1.63	2
14790872	2.73	3	2.69	3	2.49	2	2.64	3
14790873	2.21	2	2.40	2	2.59	3	2.40	2
14790874	2.69	3	2.86	3	2.30	2	2.62	3
14790879	2.07	2	2.03	2	1.91	2	2.00	2

Sample ID	Copy Number							
	r = 1		r = 2		r = 3		Average	
	Ratio (target/ref)	Estimated Copy Number	Ratio (target/ref)	Estimated Copy Number	Ratio (target/ref)	Estimated Copy Number	Ratio (target/ref)	Estimated Copy Number
14790880	1.72	2	1.95	2	2.20	2	1.96	2
14790883	1.23	1	1.24	1	1.33	1	1.27	1
14790884	1.47	1	1.83	2	0.34	0	1.21	1
14790885	1.00	1	1.09	1	0.27	0	0.79	1
14790886	0.92	1	1.08	1	1.36	1	1.12	1
14790887	0.91	1	1.00	1	1.02	1	0.97	1
14790890	1.62	2	1.84	2	1.78	2	1.75	2
14790897	4.44	4	4.01	4	5.16	5	4.54	5
14790898	2.03	2	1.89	2	1.90	2	1.94	2
14790899	2.59	3	3.79	4	3.92	4	3.43	3
14790901	1.00	1	1.23	1	1.49	1	1.24	1
14790902	0.85	1	0.40	0	0.96	1	0.74	1
14790903	1.07	1	1.04	1	0.83	1	0.98	1
14790905	1.67	2	1.80	2	1.60	2	1.69	2
14790906	1.60	2	1.79	2	2.01	2	1.80	2
14790908	2.67	3	2.20	2	2.64	3	2.51	3
14790910	0.54	1	0.68	1	0.58	1	0.60	1
14790911	2.73	3	2.56	3	3.08	3	2.79	3
14790912	2.29	2	1.73	2	2.05	2	2.02	2

Sample ID	Copy Number							
	r = 1		r = 2		r = 3		Average	
	Ratio (target/ref)	Estimated Copy Number	Ratio (target/ref)	Estimated Copy Number	Ratio (target/ref)	Estimated Copy Number	Ratio (target/ref)	Estimated Copy Number
14790915	1.34	1	0.99	1	1.12	1	1.15	1
14790916	4.19	4	3.45	3	4.07	4	3.90	4
14790917	3.19	3	3.16	3	3.48	3	3.28	3
14790918	1.18	1	1.09	1	1.11	1	1.13	1
14790919	2.59	3	2.69	3	2.86	3	2.71	3
14790922	0.97	1	0.94	1	0.96	1	0.96	1
14790923	4.29	4	4.61	5	4.14	4	4.35	4
14790925	1.03	1	0.98	1	0.96	1	0.99	1
14790926	1.49	1	1.62	2	1.48	1	1.53	2
14790931	2.07	2	2.14	2	2.06	2	2.09	2
14790933	1.76	2	1.86	2	1.80	2	1.81	2
14790936	2.36	2	2.67	3	1.83	2	2.29	2
14790937	1.10	1	1.13	1	1.07	1	1.10	1
14790940	1.71	2	1.86	2	1.85	2	1.80	2
14790944	3.39	3	4.05	4	4.13	4	3.86	4
14790946	1.69	2	1.81	2	1.85	2	1.78	2
14790948	2.09	2	2.10	2	2.15	2	2.12	2
14790949	0.90	1	0.97	1	0.89	1	0.92	1
14790958	1.01	1	1.08	1	0.92	1	1.00	1

Sample ID	Copy Number							
	r = 1		r = 2		r = 3		Average	
	Ratio (target/ref)	Estimated Copy Number	Ratio (target/ref)	Estimated Copy Number	Ratio (target/ref)	Estimated Copy Number	Ratio (target/ref)	Estimated Average Copy Number
14790959	2.10	2	1.66	2	1.59	2	1.78	2
14790965	2.07	2	2.05	2	1.81	2	1.98	2
14790966	2.14	2	2.30	2	2.37	2	2.27	2
14790967	3.31	3	3.39	3	3.36	3	3.35	3
14790968	4.78	5	4.67	5	5.07	5	4.84	5
14790969	1.03	1	1.02	1	1.08	1	1.04	1
14790972	2.15	2	2.50	3	2.39	2	2.35	2
14790973	2.09	2	2.60	3	2.65	3	2.45	2
14790981	0.81	1	0.88	1	0.87	1	0.85	1
14790982	1.13	1	1.09	1	0.97	1	1.06	1
14790985	1.39	1	1.28	1	1.39	1	1.35	1
14790986	2.41	2	2.29	2	2.41	2	2.37	2
14790987	3.17	3	2.61	3	3.24	3	3.01	3
14790988	1.44	1	1.87	2	1.72	2	1.68	2
14791040	1.39	1	1.26	1	1.37	1	1.34	1
14791066	3.37	3	3.39	3	4.48	4	3.75	4
14791067	2.80	3	2.74	3	2.56	3	2.70	3
14791068	3.56	4	3.28	3	3.68	4	3.50	4
14791070	10.97	11	9.83	10	9.45	9	10.08	10

Sample ID	Copy Number							
	r = 1		r = 2		r = 3		Average	
	Ratio (target/ref)	Estimated Copy Number	Ratio (target/ref)	Estimated Copy Number	Ratio (target/ref)	Estimated Copy Number	Ratio (target/ref)	Estimated Copy Number
14791071	1.48	1	1.37	1	1.48	1	1.44	1
14791072	1.30	1	1.56	2	1.17	1	1.34	1
14791073	4.94	5	2.10	2	4.27	4	3.77	4
14791075	3.52	4	3.48	3	3.53	4	3.51	4
14791076	1.40	1	1.70	2	1.66	2	1.59	2
14791077	1.45	1	1.58	2	1.65	2	1.56	2
14791079	2.35	2	2.55	3	2.66	3	2.52	3
14791080	1.30	1	1.43	1	1.51	2	1.41	1
14791082	2.39	2	1.77	2	1.81	2	1.99	2
14791083	2.25	2	5.63	6	2.58	3	3.49	3
14791085	1.15	1	1.56	2	1.59	2	1.44	1
14791088	1.20	1	1.23	1	1.31	1	1.25	1
14791089	1.92	2	1.75	2	1.84	2	1.84	2
14791090	5.96	6	5.09	5	5.02	5	5.36	5
14791091	3.65	4	3.35	3	3.36	3	3.45	3
14791093	2.79	3	2.60	3	2.42	2	2.60	3
14791095	2.80	3	2.71	3	2.45	2	2.65	3
14791097	4.89	5	4.91	5	4.71	5	4.84	5
14791098	2.73	3	2.74	3	2.53	3	2.67	3

Sample ID	Copy Number							
	r = 1		r = 2		r = 3		Average	
	Ratio (target/ref)	Estimated Copy Number	Ratio (target/ref)	Estimated Copy Number	Ratio (target/ref)	Estimated Copy Number	Ratio (target/ref)	Estimated Copy Number
14791101	5.94	6	5.66	6	6.14	6	5.91	6
14791102	6.73	7	6.37	6	6.93	7	6.68	7
14791106	1.87	2	2.02	2	2.05	2	1.98	2
14791107	2.00	2	1.97	2	2.11	2	2.03	2
14791109	1.32	1	1.41	1	1.38	1	1.37	1
14791110	2.36	2	2.36	2	2.26	2	2.33	2
14791111	11.11	11	10.08	10	10.32	10	10.50	11
14791112	1.19	1	1.24	1	1.27	1	1.23	1
14791113	1.18	1	1.33	1	1.34	1	1.28	1
14791114	1.07	1	1.08	1	1.11	1	1.09	1
14791115	1.42	1	1.19	1	1.18	1	1.27	1
14791116	2.28	2	2.77	3	2.36	2	2.47	2
14791118	1.29	1	1.38	1	1.37	1	1.35	1
14791119	1.28	1	1.32	1	1.30	1	1.30	1
14791120	1.69	2	1.53	2	1.53	2	1.58	2
14791122	1.26	1	0.97	1	1.06	1	1.10	1
14791123	1.13	1	1.25	1	1.27	1	1.22	1
14791124	2.10	2	2.37	2	2.37	2	2.28	2
14791127	2.09	2	2.04	2	2.59	3	2.24	2

Sample ID	Copy Number							
	r = 1		r = 2		r = 3		Average	
	Ratio (target/ref)	Estimated Copy Number	Ratio (target/ref)	Estimated Copy Number	Ratio (target/ref)	Estimated Copy Number	Ratio (target/ref)	Estimated Copy Number
14791128	1.40	1	1.63	2	1.47	1	1.50	2
14791129	1.38	1	1.15	1	1.22	1	1.25	1
14791130	1.10	1	1.16	1	1.04	1	1.10	1
14791132	3.31	3	3.49	3	3.19	3	3.33	3
14791136	2.02	2	2.08	2	1.85	2	1.98	2
14791137	0.99	1	1.06	1	0.95	1	1.00	1
14791140	1.32	1	1.29	1	1.22	1	1.27	1
14791141	1.13	1	1.02	1	1.06	1	1.07	1
14791142	1.20	1	1.06	1	1.11	1	1.12	1
14791143	1.01	1	1.02	1	0.98	1	1.00	1
14791146	1.12	1	1.04	1	1.22	1	1.12	1
14791147	1.16	1	1.14	1	1.23	1	1.17	1
14791149	2.88	3	2.88	3	3.38	3	3.05	3
14791152	5.72	6	5.05	5	4.70	5	5.15	5
14791153	0.97	1	1.13	1	1.05	1	1.05	1
14791157	1.99	2	2.63	3	2.15	2	2.26	2
14791159	1.88	2	1.99	2	2.13	2	2.00	2
14791160	1.18	1	1.31	1	1.25	1	1.25	1
14791161	1.14	1	1.30	1	1.27	1	1.24	1

Sample ID	Copy Number							
	r = 1		r = 2		r = 3		Average	
	Ratio (target/ref)	Estimated Copy Number	Ratio (target/ref)	Estimated Copy Number	Ratio (target/ref)	Estimated Copy Number	Ratio (target/ref)	Average Copy Number
14791164	2.21	2	2.19	2	4.17	4	2.86	3
14791165	2.45	2	2.62	3	2.94	3	2.67	3
14791166	5.34	5	5.16	5	5.25	5	5.25	5

Notes: target, transgene *bar*; ref, endogenous gene *hmg*.
