# Supplementary material for: High-Throughput and Accurate Determination of Transgene Copy Number and Zygosity in Transgenic Maize: From DNA Extraction to Data Analysis
Source: Int J Mol Sci. 2021 Nov 19;22(22):12487. doi: 10.3390/ijms222212487 (PMC8619409; doi:10.3390/ijms222212487)
Supplement: Supplementary file 1 [file ijms-22-12487-s001.zip › Table S5.pdf]

Table S5. Copy numbers of 2,016 T_0 samples and successive progeny (T_1 and T_2) with one technical repetition.

Transgenic Line	T_0		T_1			T_2
	Ratio (target/ref)	Estimated Copy Number	Homozygote No.	Ratio (target/ref)	Estimated Copy Number of T_0 Plant	Verification of T_1 Homozygote
14210420	0.94	1	39	4.28	2	39: two-copy homozygote
14310319	1.05	1	3	2.31	1	3: single-copy homozygote
14401378	1.04	1	4	2.45	1	4: single-copy homozygote
14320052	1.17	1	8	2.56	1	8: single-copy homozygote
14200239	0.88	1	41	3.85	2	41: two-copy homozygote
14220324	1.18	1	44	4.11	2	44: two-copy homozygote
14220339	0.78	1	7	2.02	1	7: single-copy homozygote
14230292	1.02	1	7	2.00	1	7: single-copy homozygote
14360847	1.28	1	11	1.60	1	11: single-copy homozygote
14350538	1.18	1	6	1.73	1	6: single-copy homozygote
14350542	1.08	1	4	2.58	1	4: single-copy homozygote
14350544	1.01	1	9	1.67	1	9: single-copy homozygote
14370045	1.08	1	5	2.02	1	5: single-copy homozygote
14370428	1.39	1	1	4.42	2	1: two-copy homozygote
14400520	1.36	1	1	2.50	1	1: single-copy homozygote
14350563	1.09	1	5	1.84	1	5: single-copy homozygote
14350564	0.91	1	1	2.37	1	1: single-copy homozygote
14340421	0.96	1	1	2.26	1	1: single-copy homozygote
14360957	1.24	1	1	2.33	1	1: single-copy homozygote

Transgenic Line	T_0		T_1			T_2
	**Ratio (target/ref)**	**Estimated Copy Number**	**Homozygote No.**	**Ratio (target/ref)**	**Estimated Copy Number of T_0 Plant**	**Verification of T_1 Homozygote**
14350703	1.07	1	32	2.41	1	32: single-copy homozygote
14361414	1.30	1	4	1.93	1	4: single-copy homozygote
14340143	1.18	1	12	2.55	1	12: single-copy homozygote
14390872	1.35	1	2	2.00	1	2: single-copy homozygote
14160881	1.13	1	42	2.00	1	42: single-copy homozygote
14170715	0.92	1	19	1.81	1	19: single-copy homozygote
14200479	1.14	1	2	2.48	1	2: single-copy homozygote
14350623	1.18	1	3	2.35	1	3: single-copy homozygote
14370950	1.11	1	5	2.04	1	5: single-copy homozygote
14210292	1.10	1	6	2.50	1	6: single-copy homozygote
14350454	1.04	1	5	2.08	1	5: single-copy homozygote
14350456	1.07	1	1	2.07	1	1: single-copy homozygote
14361197	1.28	1	35	2.44	1	35: single-copy homozygote
14370621	1.12	1	2	2.53	1	2: single-copy homozygote
14380998	1.13	1	4	2.16	1	4: single-copy homozygote
14361428	1.28	1	2	1.97	1	2: single-copy homozygote
14371237	0.84	1	7	2.18	1	7: single-copy homozygote
14340538	1.26	1	5	2.26	1	5: single-copy homozygote
14340658	1.12	1	13	1.64	1	13: single-copy homozygote
14340660	0.99	1	10	1.79	1	10: single-copy homozygote

Transgenic Line	T_0		T_1			T_2
	Ratio (target/ref)	Estimated Copy Number	Homozygote No.	Ratio (target/ref)	Estimated Copy Number of T_0 Plant	Verification of T_1 Homozygote
14350950	1.09	1	3	1.97	1	3: single-copy homozygote
14361454	1.14	1	9	5.98	⩾3	9: multi-copy
14390255	1.36	1	12	1.98	1	12: single-copy homozygote
14170848	0.64	1	12	2.12	1	12: single-copy homozygote
14170855	0.98	1	3	2.01	1	3: single-copy homozygote
14190548	0.58	1	36	2.38	1	36: single-copy homozygote
14190565	1.04	1	38	2.14	1	38: single-copy homozygote
14160932	1.04	1	2	2.13	1	2: single-copy homozygote
14170762	1.12	1	1	2.06	1	1: single-copy homozygote
14200036	0.99	1	5	1.96	1	5: single-copy homozygote
14380299	1.29	1	2	2.09	1	2: single-copy homozygote
14380300	1.05	1	1	2.13	1	1: single-copy homozygote
14370366	0.84	1	5	2.05	1	5: single-copy homozygote
14350656	1.09	1	31	2.21	1	31: single-copy homozygote
14371295	0.92	1	1	2.03	1	1: single-copy homozygote
14390342	1.28	1	2	2.12	1	2: single-copy homozygote
14390343	0.88	1	40	2.00	1	40: single-copy homozygote
14390355	1.15	1	1	1.89	1	1: single-copy homozygote
14361370	1.06	1	2	2.16	1	2: single-copy homozygote
14391004	1.10	1	9	2.37	1	9: single-copy homozygote

Transgenic Line	T_0		T_1			T_2
	Ratio (target/ref)	Estimated Copy Number	Homozygote No.	Ratio (target/ref)	Estimated Copy Number of T_0 Plant	Verification of T_1 Homozygote
14390487	1.28	1	4	2.19	1	4: single-copy homozygote
14350842	1.25	1	2	2.52	1	2: single-copy homozygote
14190432	1.16	1	1	2.26	1	1: single-copy homozygote
14170577	1.16	1	32	2.37	1	32: single-copy homozygote
14220112	0.95	1	4	2.44	1	4: single-copy homozygote
14170885	1.08	1	3	2.00	1	3: single-copy homozygote
14190532	1.17	1	6	2.43	1	6: single-copy homozygote
14190621	1.15	1	31	2.23	1	31: single-copy homozygote
14200602	1.18	1	5	2.00	1	5: single-copy homozygote
14220207	0.53	1	5	2.45	1	5: single-copy homozygote
14200179	1.07	1	1	2.37	1	1: single-copy homozygote
14220306	0.99	1	6	2.19	1	6: single-copy homozygote
14230234	1.03	1	8	2.14	1	8: single-copy homozygote
14230249	0.99	1	9	1.87	1	9: single-copy homozygote
14200310	1.18	1	6	2.28	1	6: single-copy homozygote
14210308	1.01	1	38	2.37	1	38: single-copy homozygote
14160715	1.20	1	3	2.04	1	3: single-copy homozygote
14220039	0.99	1	5	2.04	1	5: single-copy homozygote
14320365	1.38	1	35	1.96	1	35: single-copy homozygote
14380001	1.35	1	3	1.81	1	3: single-copy homozygote

Transgenic Line	T_0		T_1			T_2
	Ratio (target/ref)	Estimated Copy Number	Homozygote No.	Ratio (target/ref)	Estimated Copy Number of T_0 Plant	Verification of T_1 Homozygote
14180147	1.25	1	5	1.88	1	5: single-copy homozygote
14210052	1.34	1	2	2.16	1	2: single-copy homozygote
14210061	1.18	1	4	4.82	2	4: two-copy homozygote
14160622	1.11	1	31	2.36	1	31: single-copy homozygote
14170424	1.29	1	1	2.12	1	1: single-copy homozygote
14160759	1.27	1	5	2.46	1	5: single-copy homozygote
14160573	0.94	1	4	2.12	1	4: single-copy homozygote
14170654	1.13	1	2	2.39	1	2: single-copy homozygote
14170120	1.09	1	34	2.24	1	34: single-copy homozygote
14170123	1.03	1	4	2.26	1	4: single-copy homozygote
14381608	1.23	1	1	2.38	1	1: single-copy homozygote
14400968	1.29	1	1	2.43	1	1: single-copy homozygote
14430123	1.09	1	1	2.24	1	1: single-copy homozygote
14310352	1.04	1	1	1.64	1	1: single-copy homozygote
14340607	1.01	1	1	1.99	1	1: single-copy homozygote
14430640	1.15	1	33	2.55	1	33: single-copy homozygote
14410426	1.07	1	7	1.93	1	7: single-copy homozygote
14160854	1.11	1	33	2.66	1	33: single-copy homozygote
14170695	1.23	1	34	2.48	1	34: single-copy homozygote
14170706	0.89	1	32	2.21	1	32: single-copy homozygote

Transgenic Line	T_0		T_1			T_2
	Ratio (target/ref)	Estimated Copy Number	Homozygote No.	Ratio (target/ref)	Estimated Copy Number of T_0 Plant	Verification of T_1 Homozygote
14430584	1.09	1	3	1.97	1	3: single-copy homozygote
14380608	1.02	1	1	2.13	1	1: single-copy homozygote
14391260	1.06	1	1	2.23	1	1: single-copy homozygote
14390080	1.05	1	2	2.06	1	2: single-copy homozygote
14380117	1.30	1	6	2.05	1	6: single-copy homozygote
14370446	0.98	1	2	1.99	1	2: single-copy homozygote
14380422	1.23	1	4	1.96	1	4: single-copy homozygote
14400549	1.17	1	2	1.92	1	2: single-copy homozygote
14400822	1.28	1	2	2.19	1	2: single-copy homozygote
14380196	1.10	1	5	2.07	1	5: single-copy homozygote
14340247	0.93	1	1	3.93	2	1: two-copy homozygote
14380220	1.18	1	2	1.92	1	2: single-copy homozygote
14400385	1.08	1	4	2.14	1	4: single-copy homozygote
14391033	0.97	1	1	2.29	1	1: single-copy homozygote
14391049	1.21	1	1	2.30	1	1: single-copy homozygote
14400914	1.22	1	1	2.07	1	1: single-copy homozygote
14401000	1.14	1	1	2.33	1	1: single-copy homozygote
14410439	1.15	1	1	2.10	1	1: single-copy homozygote
14310085	1.21	1	2	2.47	1	2: single-copy homozygote
14390690	1.21	1	4	2.11	1	4: single-copy homozygote

Transgenic Line	T_0		T_1			T_2
	Ratio (target/ref)	Estimated Copy Number	Homozygote No.	Ratio (target/ref)	Estimated Copy Number of T_0 Plant	Verification of T_1 Homozygote
14360503	1.18	1	1	2.27	1	1: single-copy homozygote
14420290	1.14	1	3	2.31	1	3: single-copy homozygote
14190326	1.20	1	5	1.81	1	5: single-copy homozygote
14170366	1.02	1	2	2.49	1	2: single-copy homozygote
15100520	1.08	1	9	2.24	1	9: single-copy homozygote
15081919	1.23	1	5	2.14	1	5: single-copy homozygote
15081920	1.02	1	5	2.09	1	5: single-copy homozygote
15110690	1.29	1	19	5.84	⩾3	19: multi-copy
15110698	1.29	1	7	5.63	⩾3	7: multi-copy
15110634	1.10	1	12	2.34	1	12: single-copy homozygote
15120299	0.93	1	9	2.30	1	9: single-copy homozygote
15120302	0.97	1	1	2.23	1	1: single-copy homozygote
15150169	0.92	1	4	1.97	1	4: single-copy homozygote
15170320	1.06	1	4	2.00	1	4: single-copy homozygote
15141228	0.98	1	11	2.24	1	11: single-copy homozygote
15161041	1.14	1	14	2.23	1	14: single-copy homozygote
15111280	1.07	1	10	2.21	1	10: single-copy homozygote
15120631	1.02	1	4	2.63	1	4: single-copy homozygote
15121828	1.10	1	2	2.22	1	2: single-copy homozygote
15121839	1.06	1	8	4.38	2	8: two-copy homozygote

Transgenic Line	T_0		T_1			T_2
	Ratio (target/ref)	Estimated Copy Number	Homozygote No.	Ratio (target/ref)	Estimated Copy Number of T_0 Plant	Verification of T_1 Homozygote
15150230	1.06	1	11	2.42	1	11: single-copy homozygote
15150233	1.06	1	4	2.35	1	4: single-copy homozygote
15150201	1.31	1	3	2.25	1	3: single-copy homozygote
15060872	1.03	1	11	1.95	1	11: single-copy homozygote
15071092	0.96	1	8	2.03	1	8: single-copy homozygote
14300181	1.34	1	17	2.08	1	17: single-copy homozygote
15100599	1.13	1	1	2.02	1	1: single-copy homozygote
15120826	1.19	1	3	2.24	1	3: single-copy homozygote
15080779	1.23	1	13	2.32	1	13: single-copy homozygote
15141257	1.15	1	3	2.47	1	3: single-copy homozygote
15081176	1.37	1	8	4.51	2	8: two-copy homozygote
15081189	1.13	1	9	1.88	1	9: single-copy homozygote
15160210	0.98	1	12	2.35	1	12: single-copy homozygote
15111121	0.98	1	9	2.14	1	9: single-copy homozygote
15081552	0.99	1	1	2.24	1	1: single-copy homozygote
15131112	1.37	1	14	4.09	2	14: two-copy homozygote
15140420	1.16	1	4	2.05	1	4: single-copy homozygote
15120568	1.27	1	13	2.00	1	13: single-copy homozygote
15210583	1.33	1	14	4.12	2	14: two-copy homozygote
15070777	1.01	1	3	2.44	1	3: single-copy homozygote

Transgenic Line	T_0		T_1			T_2
	Ratio (target/ref)	Estimated Copy Number	Homozygote No.	Ratio (target/ref)	Estimated Copy Number of T_0 Plant	Verification of T_1 Homozygote
15121160	0.83	1	4	2.31	1	4: single-copy homozygote
15140592	1.17	1	9	5.08	2	9: two-copy homozygote
15140297	1.18	1	15	2.12	1	15: single-copy homozygote
15170950	1.00	1	6	2.26	1	6: single-copy homozygote
15180322	1.19	1	7	2.19	1	7: single-copy homozygote
15170804	1.38	1	7	5.10	⩾3	7: multi-copy
15140118	1.05	1	2	2.42	1	2: single-copy homozygote
15141556	0.97	1	13	1.83	1	13: single-copy homozygote
15160191	1.12	1	16	2.16	1	16: single-copy homozygote
15180338	1.14	1	4	4.45	2	4: two-copy homozygote
15160335	1.07	1	2	1.94	1	2: single-copy homozygote
15160377	1.03	1	21	2.18	1	21: single-copy homozygote
15180611	1.11	1	1	2.24	1	1: single-copy homozygote
15170392	1.13	1	3	2.02	1	3: single-copy homozygote
15140842	0.57	1	2	4.67	2	2: two-copy homozygote
15170557	0.57	1	12	4.33	2	12: two-copy homozygote
15070712	1.14	1	9	2.01	1	9: single-copy homozygote
15081520	0.96	1	5	2.58	1	5: single-copy homozygote
15110963	0.96	1	21	2.00	1	21: single-copy homozygote
15200377	1.15	1	6	2.12	1	6: single-copy homozygote

Transgenic Line	T_0		T_1			T_2
	**Ratio (target/ref)**	**Estimated Copy Number**	**Homozygote No.**	**Ratio (target/ref)**	**Estimated Copy Number of T_0 Plant**	**Verification of T_1 Homozygote**
15120927	1.23	1	1	2.21	1	1: single-copy homozygote
15171068	1.34	1	8	2.44	1	8: single-copy homozygote
15040107	1.32	1	9	3.55	2	9: two-copy homozygote
15040111	0.91	1	11	3.67	2	11: two-copy homozygote
15160953	1.26	1	4	4.85	⩾3	4: multi-copy
15030172	1.06	1	7	2.17	1	7: single-copy homozygote
15040132	0.82	1	6	2.12	1	6: single-copy homozygote
15060400	1.14	1	4	2.04	1	4: single-copy homozygote
15100625	1.08	1	5	2.08	1	5: single-copy homozygote
15071011	1.09	1	1	2.20	1	1: single-copy homozygote
15060132	1.19	1	2	2.28	1	2: single-copy homozygote
15070531	0.94	1	7	1.90	1	7: single-copy homozygote
15070533	1.12	1	6	2.11	1	6: single-copy homozygote
15070543	1.08	1	13	2.30	1	13: single-copy homozygote
15070548	1.10	1	14	2.28	1	14: single-copy homozygote
15080260	0.92	1	11	1.67	1	11: single-copy homozygote
15090241	0.65	1	10	2.01	1	10: single-copy homozygote
15111564	1.13	1	6	2.05	1	6: single-copy homozygote
15111737	1.13	1	1	2.04	1	1: single-copy homozygote
15111738	1.01	1	1	2.11	1	1: single-copy homozygote

Transgenic Line	T_0		T_1			T_2
	Ratio (target/ref)	Estimated Copy Number	Homozygote No.	Ratio (target/ref)	Estimated Copy Number of T_0 Plant	Verification of T_1 Homozygote
15131113	0.86	1	4	2.39	1	4: single-copy homozygote
15110418	1.06	1	2	2.08	1	2: single-copy homozygote
15080665	1.02	1	4	1.80	1	4: single-copy homozygote
15060417	1.11	1	7	1.99	1	7: single-copy homozygote
15070005	0.81	1	6	2.00	1	6: single-copy homozygote
15081723	0.95	1	5	1.71	1	5: single-copy homozygote
15100673	1.08	1	2	2.12	1	2: single-copy homozygote
15060384	1.03	1	2	2.07	1	2: single-copy homozygote
15200043	1.15	1	1	2.38	1	1: single-copy homozygote
15110319	1.19	1	2	2.38	1	2: single-copy homozygote
15110428	1.27	1	9	3.84	2	9: two-copy homozygote
14260065	1.08	1	5	2.03	1	5: single-copy homozygote
15100368	0.98	1	4	2.53	1	4: single-copy homozygote
15100451	1.04	1	10	2.28	1	10: single-copy homozygote
15090229	1.12	1	2	2.16	1	2: single-copy homozygote
15111559	1.08	1	7	2.16	1	7: single-copy homozygote
15141526	1.10	1	8	2.13	1	8: single-copy homozygote
15200665	1.18	1	6	4.15	2	6: two-copy homozygote
15190645	1.08	1	13	2.34	1	13: single-copy homozygote
15050055	1.06	1	2	4.54	2	2: two-copy homozygote

Transgenic Line	T_0		T_1			T_2
	Ratio (target/ref)	Estimated Copy Number	Homozygote No.	Ratio (target/ref)	Estimated Copy Number of T_0 Plant	Verification of T_1 Homozygote
15060569	1.06	1	2	2.38	1	2: single-copy homozygote
15081546	1.09	1	2	2.20	1	2: single-copy homozygote
15081651	1.08	1	3	2.48	1	3: single-copy homozygote
15030052	1.23	1	2	2.20	1	2: single-copy homozygote
15030115	1.07	1	1	2.12	1	1: single-copy homozygote
15070813	0.98	1	12	2.08	1	12: single-copy homozygote
15081293	1.09	1	6	2.07	1	6: single-copy homozygote
15130052	1.39	1	15	2.12	1	15: single-copy homozygote
15141024	1.11	1	2	2.24	1	2: single-copy homozygote
15140990	1.25	1	1	2.15	1	1: single-copy homozygote
15121204	1.12	1	7	2.16	1	7: single-copy homozygote
15131195	1.07	1	3	2.40	1	3: single-copy homozygote
15150228	1.25	1	3	4.07	⩾3	3: multi-copy
15131064	1.10	1	1	2.18	1	1: single-copy homozygote
15111164	1.05	1	1	2.20	1	1: single-copy homozygote
15060292	1.04	1	3	2.22	1	3: single-copy homozygote
15110528	0.99	1	1	2.25	1	1: single-copy homozygote
15150151	1.13	1	5	2.00	1	5: single-copy homozygote
15081212	1.14	1	4	2.12	1	4: single-copy homozygote
15140104	1.14	1	11	1.99	1	11: single-copy homozygote

Transgenic Line	T_0		T_1			T_2
	**Ratio (target/ref)**	**Estimated Copy Number**	**Homozygote No.**	**Ratio (target/ref)**	**Estimated Copy Number of T_0 Plant**	**Verification of T_1 Homozygote**
15180363	1.22	1	3	2.08	1	3: single-copy homozygote
15190698	1.05	1	19	2.40	1	19: single-copy homozygote
15120113	1.28	1	10	4.40	2	10: two-copy homozygote
15170033	0.53	1	1	5.52	⩾3	1: multi-copy
15060915	1.10	1	8	2.54	1	8: single-copy homozygote
15090480	1.12	1	4	2.42	1	4: single-copy homozygote
15120511	0.97	1	10	1.92	1	10: single-copy homozygote
15080059	0.96	1	9	2.18	1	9: single-copy homozygote
15150012	1.22	1	7	2.33	1	7: single-copy homozygote
15160960	1.15	1	1	2.20	1	1: single-copy homozygote
15170045	1.12	1	11	1.91	1	11: single-copy homozygote
15121881	1.13	1	9	1.62	1	9: single-copy homozygote
15180507	1.18	1	11	2.26	1	11: single-copy homozygote
15180554	1.06	1	2	1.89	1	2: single-copy homozygote
14371258	0.75	1	5	2.19	1	5: single-copy homozygote
16131430	1.30	1	9	1.92	1	9: single-copy homozygote
16141216	1.31	1	21	4.96	2	21: two-copy homozygote
16141272	1.17	1	13	1.94	1	13: single-copy homozygote
16131061	1.27	1	10	2.22	1	10: single-copy homozygote
16140761	1.21	1	1	2.00	1	1: single-copy homozygote

Transgenic Line	T_0		T_1			T_2
	**Ratio (target/ref)**	**Estimated Copy Number**	**Homozygote No.**	**Ratio (target/ref)**	**Estimated Copy Number of T_0 Plant**	**Verification of T_1 Homozygote**
16170629	1.15	1	7	1.99	1	7: single-copy homozygote
16130411	1.10	1	1	1.99	1	1: single-copy homozygote
16141062	1.28	1	4	2.00	1	4: single-copy homozygote
16170018	1.12	1	3	2.02	1	3: single-copy homozygote
16170236	1.14	1	3	2.08	1	3: single-copy homozygote
16131980	1.32	1	1	2.32	1	1: single-copy homozygote
16132036	1.39	1	11	2.06	1	11: single-copy homozygote
16170335	1.14	1	4	2.03	1	4: single-copy homozygote
15451784	0.83	1	5	1.97	1	5: single-copy homozygote
15420478	1.26	1	16	2.12	1	16: single-copy homozygote
15450976	1.22	1	14	1.95	1	14: single-copy homozygote
15460982	0.99	1	4	1.97	1	4: single-copy homozygote
15452191	1.18	1	16	3.57	2	16: two-copy homozygote
16131937	1.21	1	17	2.02	1	17: single-copy homozygote
16141199	1.34	1	8	1.90	1	8: single-copy homozygote
16141269	1.07	1	13	1.97	1	13: single-copy homozygote
15130289	0.60	1	8	3.84	2	8: two-copy homozygote
16080137	0.96	1	5	3.94	2	5: two-copy homozygote
15480840	0.56	1	6	2.09	1	6: single-copy homozygote
15481096	0.98	1	13	1.90	1	13: single-copy homozygote

Transgenic Line	T_0		T_1			T_2
	**Ratio (target/ref)**	**Estimated Copy Number**	**Homozygote No.**	**Ratio (target/ref)**	**Estimated Copy Number of T_0 Plant**	**Verification of T_1 Homozygote**
15471273	1.29	1	6	1.99	1	6: single-copy homozygote
15461128	1.26	1	4	2.04	1	4: single-copy homozygote
15501348	1.17	1	11	2.03	1	11: single-copy homozygote
15521034	1.26	1	5	2.08	1	5: single-copy homozygote
16030625	1.12	1	10	2.43	1	10: single-copy homozygote
16040632	0.67	1	3	1.99	1	3: single-copy homozygote
15480413	1.10	1	10	2.00	1	10: single-copy homozygote
15492608	1.38	1	3	2.08	1	3: single-copy homozygote
15501083	1.16	1	2	2.17	1	2: single-copy homozygote
16030009	1.03	1	15	1.92	1	15: single-copy homozygote
16030011	1.07	1	6	2.01	1	6: single-copy homozygote
15491447	0.95	1	9	2.00	1	9: single-copy homozygote
15500313	1.12	1	9	2.00	1	9: single-copy homozygote
15521137	1.24	1	5	1.94	1	5: single-copy homozygote
16030057	1.09	1	15	1.88	1	15: single-copy homozygote
15491449	1.15	1	12	1.98	1	12: single-copy homozygote
15491450	1.29	1	4	2.00	1	4: single-copy homozygote
15493103	0.84	1	15	3.95	2	15: two-copy homozygote
15491580	1.30	1	11	1.99	1	11: single-copy homozygote
15493057	1.24	1	7	1.94	1	7: single-copy homozygote

Transgenic Line	T_0		T_1			T_2
	Ratio (target/ref)	Estimated Copy Number	Homozygote No.	Ratio (target/ref)	Estimated Copy Number of T_0 Plant	Verification of T_1 Homozygote
15493093	1.15	1	7	1.77	1	7: single-copy homozygote
15501304	1.35	1	2	1.99	1	2: single-copy homozygote
15501312	1.37	1	14	1.87	1	14: single-copy homozygote
15510421	1.21	1	4	1.97	1	4: single-copy homozygote
16030002	1.10	1	11	2.35	1	11: single-copy homozygote
16030017	1.26	1	6	2.08	1	6: single-copy homozygote
16040368	1.04	1	5	2.30	1	5: single-copy homozygote
16040369	0.93	1	6	2.04	1	6: single-copy homozygote
15470744	1.18	1	13	2.00	1	13: single-copy homozygote
16030013	1.04	1	12	1.98	1	12: single-copy homozygote
16140976	1.35	1	5	1.89	1	5: single-copy homozygote
15520565	1.34	1	16	2.02	1	16: single-copy homozygote
16010210	1.28	1	3	4.33	2	3: two-copy homozygote
16030050	0.95	1	4	1.67	1	4: single-copy homozygote
15520065	1.10	1	4	2.04	1	4: single-copy homozygote
15510377	0.92	1	10	1.95	1	10: single-copy homozygote
16150175	1.22	1	6	2.01	1	6: single-copy homozygote
15491158	1.01	1	21	2.05	1	21: single-copy homozygote
15500855	1.12	1	12	2.05	1	12: single-copy homozygote
15451083	1.35	1	15	1.95	1	15: single-copy homozygote

Transgenic Line	T_0		T_1			T_2
	Ratio (target/ref)	Estimated Copy Number	Homozygote No.	Ratio (target/ref)	Estimated Copy Number of T_0 Plant	Verification of T_1 Homozygote
15410466	1.14	1	6	1.98	1	6: single-copy homozygote
15410495	1.10	1	2	1.98	1	2: single-copy homozygote
15462162	1.24	1	9	1.68	1	9: single-copy homozygote
15451687	1.30	1	7	1.92	1	7: single-copy homozygote
16010040	0.73	1	13	2.31	1	13: single-copy homozygote
15492573	1.09	1	12	1.91	1	12: single-copy homozygote
16010343	1.27	1	2	2.05	1	2: single-copy homozygote
16010364	1.38	1	4	2.07	1	4: single-copy homozygote
15510476	1.15	1	11	2.05	1	11: single-copy homozygote
16040486	1.22	1	12	2.15	1	12: single-copy homozygote
16010516	1.38	1	11	3.87	2	11: two-copy homozygote
16010539	1.35	1	4	1.97	1	4: single-copy homozygote
16040084	1.03	1	2	1.80	1	2: single-copy homozygote
15510471	1.02	1	2	2.21	1	2: single-copy homozygote
15430201	1.32	1	26	1.99	1	26: single-copy homozygote
15470153	0.99	1	11	2.02	1	11: single-copy homozygote
16140567	0.81	1	5	2.00	1	5: single-copy homozygote
16140692	1.26	1	16	1.98	1	16: single-copy homozygote
16150184	1.31	1	13	2.00	1	13: single-copy homozygote
16160257	1.16	1	14	2.01	1	14: single-copy homozygote

Transgenic Line	T_0		T_1			T_2
	Ratio (target/ref)	Estimated Copy Number	Homozygote No.	Ratio (target/ref)	Estimated Copy Number of T_0 Plant	Verification of T_1 Homozygote
16060073	1.19	1	11	1.99	1	11: single-copy homozygote
16170114	1.06	1	11	1.85	1	11: single-copy homozygote
16130751	1.24	1	8	2.05	1	8: single-copy homozygote
16180471	1.14	1	3	2.03	1	3: single-copy homozygote
16080391	1.21	1	19	2.04	1	19: single-copy homozygote
16020009	1.10	1	12	2.02	1	12: single-copy homozygote
16131050	1.16	1	20	1.98	1	20: single-copy homozygote
16160024	1.07	1	5	1.67	1	5: single-copy homozygote
16040521	1.08	1	14	2.02	1	14: single-copy homozygote
16030638	1.34	1	2	1.98	1	2: single-copy homozygote
16040500	1.03	1	6	2.08	1	6: single-copy homozygote
16050122	1.01	1	6	1.96	1	6: single-copy homozygote
16050499	1.16	1	19	1.99	1	19: single-copy homozygote
16040578	1.25	1	17	2.04	1	17: single-copy homozygote
16160094	1.39	1	13	1.99	1	13: single-copy homozygote
16150002	1.05	1	5	1.98	1	5: single-copy homozygote
15491097	1.11	1	2	2.04	1	2: single-copy homozygote
15290798	1.29	1	13	1.66	1	13: single-copy homozygote
15451660	0.78	1	9	4.04	2	9: two-copy homozygote
16030350	1.19	1	8	2.09	1	8: single-copy homozygote

Transgenic Line	T_0		T_1			T_2
	Ratio (target/ref)	Estimated Copy Number	Homozygote No.	Ratio (target/ref)	Estimated Copy Number of T_0 Plant	Verification of T_1 Homozygote
16030371	1.27	1	13	2.12	1	13: single-copy homozygote
16030538	1.05	1	14	2.05	1	14: single-copy homozygote
16040327	1.40	1	4	1.98	1	4: single-copy homozygote
16030434	0.91	1	18	1.94	1	18: single-copy homozygote
16030450	1.01	1	1	2.00	1	1: single-copy homozygote
16050028	0.61	1	7	3.96	2	7: two-copy homozygote
16050089	0.52	1	18	2.01	1	18: single-copy homozygote
16060340	1.21	1	3	2.08	1	3: single-copy homozygote
16040112	1.30	1	8	1.98	1	8: single-copy homozygote
15450657	1.21	1	7	2.03	1	7: single-copy homozygote
15450856	1.09	1	18	2.21	1	18: single-copy homozygote
15451183	1.17	1	3	1.96	1	3: single-copy homozygote
15451261	1.40	1	8	4.08	2	8: two-copy homozygote
15370559	0.87	1	10	1.62	1	10: single-copy homozygote
15490178	1.11	1	1	2.06	1	1: single-copy homozygote
15520753	1.32	1	4	1.92	1	4: single-copy homozygote
16060204	1.26	1	12	2.15	1	12: single-copy homozygote
16060211	1.35	1	2	1.98	1	2: single-copy homozygote
16140391	1.16	1	19	1.99	1	19: single-copy homozygote
16160030	1.36	1	14	2.06	1	14: single-copy homozygote

Transgenic Line	T_0		T_1			T_2
	Ratio (target/ref)	Estimated Copy Number	Homozygote No.	Ratio (target/ref)	Estimated Copy Number of T_0 Plant	Verification of T_1 Homozygote
16160031	1.26	1	6	2.00	1	6: single-copy homozygote
16160035	1.31	1	5	2.01	1	5: single-copy homozygote
16160152	0.84	1	5	2.01	1	5: single-copy homozygote
16160160	0.98	1	1	1.85	1	1: single-copy homozygote
16141280	1.20	1	17	1.97	1	17: single-copy homozygote
15491757	1.25	1	13	2.00	1	13: single-copy homozygote
15492922	1.04	1	9	2.04	1	9: single-copy homozygote
15480183	1.04	1	6	1.96	1	6: single-copy homozygote
15481071	0.91	1	1	1.99	1	1: single-copy homozygote
15471133	1.25	1	6	2.05	1	6: single-copy homozygote
16150177	1.19	1	8	3.84	2	8: two-copy homozygote
16140463	1.31	1	6	1.94	1	6: single-copy homozygote
16160432	0.97	1	3	2.00	1	3: single-copy homozygote
16170529	1.16	1	18	1.99	1	18: single-copy homozygote
16160711	0.97	1	5	1.74	1	5: single-copy homozygote
16160586	1.11	1	17	1.96	1	17: single-copy homozygote
15270383	1.21	1	4	1.98	1	4: single-copy homozygote
15300750	0.81	1	12	4.02	2	12: two-copy homozygote
15421055	0.80	1	22	4.04	2	22: two-copy homozygote
15440873	1.12	1	5	1.97	1	5: single-copy homozygote

Transgenic Line	T_0		T_1			T_2
	Ratio (target/ref)	Estimated Copy Number	Homozygote No.	Ratio (target/ref)	Estimated Copy Number of T_0 Plant	Verification of T_1 Homozygote
15520884	1.16	1	8	2.07	1	8: single-copy homozygote
16150091	1.10	1	1	2.01	1	1: single-copy homozygote
16160312	1.00	1	1	1.85	1	1: single-copy homozygote
16160333	1.18	1	5	2.15	1	5: single-copy homozygote
16170596	0.99	1	15	1.98	1	15: single-copy homozygote
16130096	1.31	1	13	2.00	1	13: single-copy homozygote
16080164	1.39	1	17	1.98	1	17: single-copy homozygote
16160499	1.20	1	19	2.05	1	19: single-copy homozygote
15360018	0.82	1	4	2.32	1	4: single-copy homozygote
15380275	0.68	1	1	2.18	1	1: single-copy homozygote
15390260	1.36	1	5	2.39	1	5: single-copy homozygote
15400048	1.27	1	2	2.02	1	2: single-copy homozygote
15400100	0.49	0	7	1.83	1	7: single-copy homozygote
16140244	1.30	1	9	2.07	1	9: single-copy homozygote
15451449	1.28	1	2	1.97	1	2: single-copy homozygote
15491613	0.99	1	1	1.97	1	1: single-copy homozygote
16030034	0.81	1	11	1.85	1	11: single-copy homozygote
15500288	1.00	1	14	2.00	1	14: single-copy homozygote
16010669	1.35	1	1	2.41	1	1: single-copy homozygote
16010743	1.26	1	12	1.82	1	12: single-copy homozygote

Transgenic Line	T_0		T_1			T_2
	Ratio (target/ref)	Estimated Copy Number	Homozygote No.	Ratio (target/ref)	Estimated Copy Number of T_0 Plant	Verification of T_1 Homozygote
16020017	1.10	1	14	2.08	1	14: single-copy homozygote
16040142	1.00	1	16	1.90	1	16: single-copy homozygote
16040148	0.98	1	3	1.95	1	3: single-copy homozygote
16040167	1.22	1	6	1.94	1	6: single-copy homozygote
16040185	1.24	1	4	1.77	1	4: single-copy homozygote
16040197	1.27	1	3	1.87	1	3: single-copy homozygote
16040218	1.14	1	14	2.05	1	14: single-copy homozygote
16050523	0.81	1	16	1.97	1	16: single-copy homozygote
16141027	1.15	1	8	1.86	1	8: single-copy homozygote
16180138	1.10	1	15	1.99	1	15: single-copy homozygote
16141437	1.20	1	15	1.68	1	15: single-copy homozygote
15050410	1.07	1	10	2.00	1	10: single-copy homozygote
16160223	1.18	1	17	1.95	1	17: single-copy homozygote
16160265	1.16	1	15	2.00	1	15: single-copy homozygote
16160288	1.13	1	6	2.01	1	6: single-copy homozygote
16130134	1.28	1	14	1.98	1	14: single-copy homozygote
16160092	1.32	1	2	4.85	⩾3	2: multi-copy
16131974	0.95	1	9	5.80	⩾3	9: multi-copy
16160765	1.15	1	1	1.85	1	1: single-copy homozygote
16160815	1.26	1	10	2.01	1	10: single-copy homozygote

Transgenic Line	T_0		T_1			T_2
	Ratio (target/ref)	Estimated Copy Number	Homozygote No.	Ratio (target/ref)	Estimated Copy Number of T_0 Plant	Verification of T_1 Homozygote
16090466	0.85	1	9	1.99	1	9: single-copy homozygote
16140586	1.38	1	6	2.25	1	6: single-copy homozygote
16150165	1.06	1	11	2.03	1	11: single-copy homozygote
15490974	1.17	1	8	2.07	1	8: single-copy homozygote
15492035	1.27	1	3	1.97	1	3: single-copy homozygote
15492040	1.06	1	12	2.04	1	12: single-copy homozygote
16140004	1.12	1	3	1.74	1	3: single-copy homozygote
16140183	1.19	1	7	2.00	1	7: single-copy homozygote
15520834	0.94	1	4	1.96	1	4: single-copy homozygote
16010823	1.04	1	6	3.92	2	6: two-copy homozygote
15491967	1.37	1	13	2.00	1	13: single-copy homozygote
16010486	1.17	1	11	1.99	1	11: single-copy homozygote
15460557	1.19	1	15	2.00	1	15: single-copy homozygote
15460883	1.04	1	11	1.84	1	11: single-copy homozygote
15481563	1.07	1	9	1.95	1	9: single-copy homozygote
15481650	1.11	1	2	2.05	1	2: single-copy homozygote
15481668	1.12	1	8	1.94	1	8: single-copy homozygote
15500798	1.11	1	1	2.53	1	1: single-copy homozygote
15510342	1.21	1	6	2.07	1	6: single-copy homozygote
16010658	0.97	1	1	2.18	1	1: single-copy homozygote

Transgenic Line	T_0		T_1			T_2
	Ratio (target/ref)	Estimated Copy Number	Homozygote No.	Ratio (target/ref)	Estimated Copy Number of T_0 Plant	Verification of T_1 Homozygote
16010661	1.15	1	3	2.25	1	3: single-copy homozygote
16030523	1.09	1	2	2.02	1	2: single-copy homozygote
16030539	1.09	1	7	1.89	1	7: single-copy homozygote
15470288	1.16	1	13	2.00	1	13: single-copy homozygote
15471144	1.19	1	4	2.12	1	4: single-copy homozygote
15481339	1.09	1	8	2.01	1	8: single-copy homozygote
15491601	1.01	1	1	2.00	1	1: single-copy homozygote
15460816	1.21	1	5	2.01	1	5: single-copy homozygote
15500791	1.16	1	3	2.01	1	3: single-copy homozygote
15461991	1.26	1	12	1.98	1	12: single-copy homozygote
15492008	1.22	1	9	2.11	1	9: single-copy homozygote
15460889	1.28	1	16	2.03	1	16: single-copy homozygote
15490920	1.34	1	1	1.86	1	1: single-copy homozygote
15491739	1.13	1	8	2.02	1	8: single-copy homozygote
16010245	1.24	1	5	1.95	1	5: single-copy homozygote
15491693	1.24	1	4	2.03	1	4: single-copy homozygote
15520436	1.27	1	10	1.99	1	10: single-copy homozygote
15490884	1.13	1	2	1.94	1	2: single-copy homozygote
15470537	1.24	1	10	1.95	1	10: single-copy homozygote
15470588	1.07	1	14	1.98	1	14: single-copy homozygote

Transgenic Line	T_0		T_1			T_2
	**Ratio (target/ref)**	**Estimated Copy Number**	**Homozygote No.**	**Ratio (target/ref)**	**Estimated Copy Number of T_0 Plant**	**Verification of T_1 Homozygote**
15481923	0.99	1	11	1.91	1	11: single-copy homozygote
16020132	1.18	1	1	2.03	1	1: single-copy homozygote
16020143	1.32	1	1	1.94	1	1: single-copy homozygote
15501305	1.10	1	9	2.01	1	9: single-copy homozygote
15520141	0.66	1	2	1.88	1	2: single-copy homozygote
15520304	0.67	1	8	3.70	2	8: two-copy homozygote
16010092	1.21	1	4	2.03	1	4: single-copy homozygote
15461322	1.19	1	14	1.94	1	14: single-copy homozygote
15461786	1.23	1	20	1.68	1	20: single-copy homozygote
15461022	1.20	1	13	1.95	1	13: single-copy homozygote
15480644	0.96	1	13	4.76	⩾3	13: multi-copy
15490271	1.13	1	4	2.01	1	4: single-copy homozygote
15500141	1.12	1	3	3.57	2	3: two-copy homozygote
15500299	1.02	1	12	1.87	1	12: single-copy homozygote
15490349	1.26	1	6	1.99	1	6: single-copy homozygote
15490401	1.17	1	1	1.97	1	1: single-copy homozygote
16010688	1.14	1	1	1.98	1	1: single-copy homozygote
16050011	1.12	1	20	2.02	1	20: single-copy homozygote
15481878	1.16	1	11	2.01	1	11: single-copy homozygote
15490197	1.06	1	11	2.13	1	11: single-copy homozygote

Transgenic Line	T_0		T_1			T_2
	**Ratio (target/ref)**	**Estimated Copy Number**	**Homozygote No.**	**Ratio (target/ref)**	**Estimated Copy Number of T_0 Plant**	**Verification of T_1 Homozygote**
15490364	0.85	1	8	2.06	1	8: single-copy homozygote
15450562	0.85	1	2	2.08	1	2: single-copy homozygote
15492714	1.15	1	4	2.06	1	4: single-copy homozygote
15500213	0.99	1	3	2.04	1	3: single-copy homozygote
15500079	1.20	1	16	1.96	1	16: single-copy homozygote
16010630	1.17	1	5	1.96	1	5: single-copy homozygote
15441370	1.28	1	5	2.04	1	5: single-copy homozygote
15470935	1.22	1	12	2.02	1	12: single-copy homozygote
15520931	1.04	1	11	1.89	1	11: single-copy homozygote
16030616	1.06	1	12	1.90	1	12: single-copy homozygote
15491689	1.22	1	1	2.00	1	1: single-copy homozygote
15491786	1.12	1	3	2.02	1	3: single-copy homozygote
15491823	1.24	1	7	1.97	1	7: single-copy homozygote
15491837	1.18	1	15	1.91	1	15: single-copy homozygote
16040458	1.01	1	8	1.75	1	8: single-copy homozygote
15450664	1.11	1	10	2.02	1	10: single-copy homozygote
15500472	1.31	1	6	1.93	1	6: single-copy homozygote
15500498	0.84	1	5	2.06	1	5: single-copy homozygote
15521038	1.15	1	4	2.13	1	4: single-copy homozygote
15500626	1.34	1	2	1.81	1	2: single-copy homozygote

Transgenic Line	T_0		T_1			T_2
	Ratio (target/ref)	Estimated Copy Number	Homozygote No.	Ratio (target/ref)	Estimated Copy Number of T_0 Plant	Verification of T_1 Homozygote
15482138	1.05	1	5	1.99	1	5: single-copy homozygote
16010807	1.12	1	1	1.98	1	1: single-copy homozygote
15461201	1.03	1	3	2.07	1	3: single-copy homozygote
15461261	1.33	1	9	2.11	1	9: single-copy homozygote
15481124	1.15	1	4	2.02	1	4: single-copy homozygote
16030654	1.27	1	8	1.92	1	8: single-copy homozygote
15451151	1.16	1	10	4.09	2	10: two-copy homozygote
15490269	1.30	1	2	1.87	1	2: single-copy homozygote
15492723	1.22	1	3	1.97	1	3: single-copy homozygote
15451303	1.26	1	13	1.98	1	13: single-copy homozygote
15491643	1.12	1	12	2.00	1	12: single-copy homozygote
15471187	1.14	1	1	2.12	1	1: single-copy homozygote
15490982	1.17	1	11	1.97	1	11: single-copy homozygote
15480865	1.34	1	16	1.90	1	16: single-copy homozygote
15520539	0.84	1	8	2.07	1	8: single-copy homozygote
16030164	1.08	1	1	2.03	1	1: single-copy homozygote
16030309	0.99	1	26	2.01	1	26: single-copy homozygote
16030643	0.94	1	15	1.99	1	15: single-copy homozygote
15470543	0.91	1	23	1.92	1	23: single-copy homozygote
15490070	1.17	1	15	1.91	1	15: single-copy homozygote

Transgenic Line	T_0		T_1			T_2
	Ratio (target/ref)	Estimated Copy Number	Homozygote No.	Ratio (target/ref)	Estimated Copy Number of T_0 Plant	Verification of T_1 Homozygote
15490301	1.16	1	13	2.07	1	13: single-copy homozygote
15490476	1.16	1	12	2.02	1	12: single-copy homozygote
15490557	0.84	1	11	1.93	1	11: single-copy homozygote
15490626	1.29	1	8	1.90	1	8: single-copy homozygote
15470538	1.07	1	2	2.07	1	2: single-copy homozygote
15490237	1.27	1	14	2.04	1	14: single-copy homozygote
15520912	1.23	1	4	2.01	1	4: single-copy homozygote
15520966	1.17	1	10	1.94	1	10: single-copy homozygote
15520967	1.39	1	4	1.96	1	4: single-copy homozygote
16010638	1.09	1	4	1.83	1	4: single-copy homozygote
15462054	1.08	1	9	1.92	1	9: single-copy homozygote
15462055	1.03	1	15	1.93	1	15: single-copy homozygote
15462229	1.11	1	12	2.00	1	12: single-copy homozygote
15492061	1.17	1	6	3.82	2	6: two-copy homozygote
15492215	1.14	1	6	1.97	1	6: single-copy homozygote
15492383	1.06	1	1	1.96	1	1: single-copy homozygote
15451125	1.29	1	7	2.03	1	7: single-copy homozygote
15451237	0.76	1	10	1.97	1	10: single-copy homozygote
15461443	1.18	1	7	1.96	1	7: single-copy homozygote
15490265	1.32	1	8	1.71	1	8: single-copy homozygote

Transgenic Line	T_0		T_1			T_2
	Ratio (target/ref)	Estimated Copy Number	Homozygote No.	Ratio (target/ref)	Estimated Copy Number of T_0 Plant	Verification of T_1 Homozygote
15461407	1.13	1	10	2.00	1	10: single-copy homozygote
15490858	1.31	1	3	2.06	1	3: single-copy homozygote
15491708	1.18	1	15	1.97	1	15: single-copy homozygote
16030146	1.19	1	19	1.99	1	19: single-copy homozygote
15470574	0.95	1	16	2.32	1	16: single-copy homozygote
15460720	1.12	1	10	2.05	1	10: single-copy homozygote
15460571	1.25	1	7	1.82	1	7: single-copy homozygote
15520271	1.16	1	3	1.97	1	3: single-copy homozygote
15430698	0.95	1	6	1.91	1	6: single-copy homozygote
15491440	1.04	1	13	1.96	1	13: single-copy homozygote
15491505	1.16	1	23	2.00	1	23: single-copy homozygote
16020174	0.88	1	7	4.35	2	7: two-copy homozygote
16020175	1.10	1	1	2.22	1	1: single-copy homozygote
16020242	1.10	1	11	1.98	1	11: single-copy homozygote
16030183	1.17	1	3	1.99	1	3: single-copy homozygote
16020163	1.13	1	1	1.83	1	1: single-copy homozygote
15511009	1.10	1	8	4.20	2	8: two-copy homozygote
15492260	1.07	1	9	4.11	2	9: two-copy homozygote
15500811	1.19	1	2	1.93	1	2: single-copy homozygote
15481155	1.11	1	1	2.03	1	1: single-copy homozygote

**Transgenic Line**	**T_0**		**T_1**			**T_2**
	**Ratio (target/ref)**	**Estimated Copy Number**	**Homozygote No.**	**Ratio (target/ref)**	**Estimated Copy Number of T_0 Plant**	**Verification of T_1 Homozygote**
15520029	0.82	1	12	1.92	1	12: single-copy homozygote
15510478	1.11	1	1	1.99	1	1: single-copy homozygote
15461813	1.04	1	1	2.13	1	1: single-copy homozygote
15492073	1.20	1	2	1.96	1	2: single-copy homozygote
15481541	1.14	1	1	2.11	1	1: single-copy homozygote
15470859	0.65	1	6	1.98	1	6: single-copy homozygote
15500992	1.19	1	8	2.10	1	8: single-copy homozygote
15480932	1.17	1	4	2.04	1	4: single-copy homozygote
16010021	1.16	1	3	2.05	1	3: single-copy homozygote
15451601	0.96	1	2	4.10	2	2: two-copy homozygote
15462092	0.90	1	4	1.96	1	4: single-copy homozygote
15491388	1.05	1	5	1.98	1	5: single-copy homozygote
15491406	1.04	1	7	1.97	1	7: single-copy homozygote
15500795	1.12	1	8	1.98	1	8: single-copy homozygote
15480778	1.13	1	3	2.24	1	3: single-copy homozygote
15491592	1.28	1	22	2.00	1	22: single-copy homozygote
15491293	1.14	1	8	4.00	2	8: two-copy homozygote
15491352	1.18	1	7	2.04	1	7: single-copy homozygote
15461869	1.17	1	19	1.96	1	19: single-copy homozygote
15511029	1.13	1	14	2.00	1	14: single-copy homozygote

Transgenic Line	T_0		T_1			T_2
	**Ratio (target/ref)**	**Estimated Copy Number**	**Homozygote No.**	**Ratio (target/ref)**	**Estimated Copy Number of T_0 Plant**	**Verification of T_1 Homozygote**
15390427	1.20	1	24	2.01	1	24: single-copy homozygote
15300728	1.03	1	1	1.97	1	1: single-copy homozygote
15300763	1.20	1	12	2.03	1	12: single-copy homozygote
15260352	1.22	1	1	2.00	1	1: single-copy homozygote
15290079	1.39	1	20	2.08	1	20: single-copy homozygote
15330785	1.17	1	8	2.03	1	8: single-copy homozygote
15290186	1.36	1	6	1.96	1	6: single-copy homozygote
16110983	1.38	1	4	2.35	1	4: single-copy homozygote
16131903	0.99	1	6	2.06	1	6: single-copy homozygote
16080290	1.36	1	12	2.11	1	12: single-copy homozygote
16100413	1.14	1	2	1.95	1	2: single-copy homozygote
16121095	1.18	1	4	1.99	1	4: single-copy homozygote
15111558	1.03	1	13	2.31	1	13: single-copy homozygote
15180006	1.17	1	2	1.99	1	2: single-copy homozygote
16140847	0.99	1	13	2.04	1	13: single-copy homozygote
16111334	1.24	1	5	1.88	1	5: single-copy homozygote
14371384	1.06	1	1	2.28	1	1: single-copy homozygote
14391435	1.10	1	15	1.97	1	15: single-copy homozygote
14310219	1.35	1	1	2.00	1	1: single-copy homozygote
14330052	1.27	1	8	1.96	1	8: single-copy homozygote

Transgenic Line	T_0		T_1			T_2
	**Ratio (target/ref)**	**Estimated Copy Number**	**Homozygote No.**	**Ratio (target/ref)**	**Estimated Copy Number of T_0 Plant**	**Verification of T_1 Homozygote**
16170023	0.97	1	12	2.04	1	12: single-copy homozygote
16170066	1.02	1	10	1.98	1	10: single-copy homozygote
16130438	1.26	1	8	2.02	1	8: single-copy homozygote
16200274	1.10	1	7	1.87	1	7: single-copy homozygote
15121284	0.49	0	2	2.10	1	2: single-copy homozygote
15120340	1.09	1	11	2.11	1	11: single-copy homozygote
15120343	1.11	1	6	3.64	2	6: two-copy homozygote
15400785	1.13	1	7	2.10	1	7: single-copy homozygote
16131089	1.26	1	9	2.09	1	9: single-copy homozygote
16150077	1.21	1	15	2.08	1	15: single-copy homozygote
16111469	1.37	1	10	1.98	1	10: single-copy homozygote
16111470	1.23	1	14	2.07	1	14: single-copy homozygote
16180548	0.91	1	13	2.00	1	13: single-copy homozygote
15441087	1.20	1	1	2.06	1	1: single-copy homozygote
15451075	1.01	1	10	2.08	1	10: single-copy homozygote
16111431	1.29	1	9	2.13	1	9: single-copy homozygote
16111444	1.30	1	4	2.20	1	4: single-copy homozygote
16111502	1.33	1	1	1.90	1	1: single-copy homozygote
16121181	1.15	1	4	2.40	1	4: single-copy homozygote
16121182	0.86	1	5	1.99	1	5: single-copy homozygote

Transgenic Line	T_0		T_1			T_2
	Ratio (target/ref)	Estimated Copy Number	Homozygote No.	Ratio (target/ref)	Estimated Copy Number of T_0 Plant	Verification of T_1 Homozygote
16121185	1.26	1	21	1.95	1	21: single-copy homozygote
16130513	1.01	1	9	2.03	1	9: single-copy homozygote
16160010	1.19	1	8	2.30	1	8: single-copy homozygote
16160056	1.33	1	14	2.12	1	14: single-copy homozygote
16160058	1.24	1	4	3.77	2	4: two-copy homozygote
16130011	0.60	1	3	1.94	1	3: single-copy homozygote
16130170	1.11	1	13	2.09	1	13: single-copy homozygote
16210018	1.25	1	7	1.91	1	7: single-copy homozygote
16210056	1.15	1	9	2.25	1	9: single-copy homozygote
16210057	1.37	1	16	2.16	1	16: single-copy homozygote
16210060	1.27	1	6	1.91	1	6: single-copy homozygote
16110667	1.24	1	17	2.05	1	17: single-copy homozygote
16110695	0.85	1	13	2.05	1	13: single-copy homozygote
16111351	1.37	1	10	2.25	1	10: single-copy homozygote
16121224	1.21	1	8	2.06	1	8: single-copy homozygote
14340365	1.21	1	10	2.13	1	10: single-copy homozygote
16140719	1.13	1	12	2.01	1	12: single-copy homozygote
16111459	1.36	1	2	2.08	1	2: single-copy homozygote
16120220	1.23	1	17	1.96	1	17: single-copy homozygote
16111108	1.24	1	6	1.88	1	6: single-copy homozygote

Transgenic Line	T_0		T_1			T_2
	Ratio (target/ref)	Estimated Copy Number	Homozygote No.	Ratio (target/ref)	Estimated Copy Number of T_0 Plant	Verification of T_1 Homozygote
16111109	1.06	1	2	1.83	1	2: single-copy homozygote
16111530	0.46	0	7	1.87	1	7: single-copy homozygote
16160406	1.30	1	13	1.87	1	13: single-copy homozygote
16131207	1.39	1	16	1.76	1	16: single-copy homozygote
14371185	0.94	1	12	1.97	1	12: single-copy homozygote
16120260	1.16	1	16	1.82	1	16: single-copy homozygote
16200074	1.06	1	3	2.14	1	3: single-copy homozygote
14381495	1.29	1	21	2.22	1	21: single-copy homozygote
14381497	1.30	1	9	2.02	1	9: single-copy homozygote
14340491	1.11	1	4	2.06	1	4: single-copy homozygote
16111093	1.40	1	15	2.04	1	15: single-copy homozygote
16111197	1.15	1	14	2.00	1	14: single-copy homozygote
15441782	1.19	1	10	1.95	1	10: single-copy homozygote
15100832	0.96	1	6	2.03	1	6: single-copy homozygote
15100846	1.13	1	6	2.08	1	6: single-copy homozygote
15170425	1.01	1	6	2.14	1	6: single-copy homozygote
15171242	1.18	1	2	2.30	1	2: single-copy homozygote
15180738	1.17	1	13	2.33	1	13: single-copy homozygote
16120451	1.28	1	4	2.04	1	4: single-copy homozygote
16100041	1.00	1	7	1.93	1	7: single-copy homozygote

Transgenic Line	T_0		T_1			T_2
	Ratio (target/ref)	Estimated Copy Number	Homozygote No.	Ratio (target/ref)	Estimated Copy Number of T_0 Plant	Verification of T_1 Homozygote
16100087	1.19	1	11	2.01	1	11: single-copy homozygote
16100089	1.03	1	9	1.99	1	9: single-copy homozygote
16100091	1.13	1	7	1.97	1	7: single-copy homozygote
16110223	1.10	1	10	1.91	1	10: single-copy homozygote
16110255	1.33	1	1	1.77	1	1: single-copy homozygote
16130399	1.33	1	6	2.11	1	6: single-copy homozygote
15160877	1.08	1	5	1.96	1	5: single-copy homozygote
15200052	1.18	1	9	1.85	1	9: single-copy homozygote
15211035	0.81	1	6	1.80	1	6: single-copy homozygote
15410021	1.20	1	14	2.00	1	14: single-copy homozygote
15340092	1.34	1	7	3.94	2	7: two-copy homozygote
15360071	1.30	1	16	1.99	1	16: single-copy homozygote
16140935	1.33	1	13	2.02	1	13: single-copy homozygote
16120356	1.10	1	1	1.99	1	1: single-copy homozygote
15481037	1.13	1	4	1.93	1	4: single-copy homozygote
16030239	1.16	1	13	2.03	1	13: single-copy homozygote
16030311	1.16	1	1	2.03	1	1: single-copy homozygote
15160020	1.02	1	14	2.24	1	14: single-copy homozygote
15160028	1.01	1	8	2.03	1	8: single-copy homozygote
15140965	1.05	1	8	2.29	1	8: single-copy homozygote

Transgenic Line	T_0		T_1			T_2
	Ratio (target/ref)	Estimated Copy Number	Homozygote No.	Ratio (target/ref)	Estimated Copy Number of T_0 Plant	Verification of T_1 Homozygote
15490018	1.18	1	7	2.03	1	7: single-copy homozygote
15170676	1.03	1	3	1.85	1	3: single-copy homozygote
15181309	1.16	1	11	2.09	1	11: single-copy homozygote
16131638	1.18	1	7	2.06	1	7: single-copy homozygote
16120427	1.37	1	1	2.05	1	1: single-copy homozygote
16240427	1.32	1	2	2.01	1	2: single-copy homozygote
16141276	1.26	1	2	1.97	1	2: single-copy homozygote
16210591	1.19	1	6	2.05	1	6: single-copy homozygote
16230455	0.91	1	2	2.19	1	2: single-copy homozygote
16240408	0.79	1	2	2.47	1	2: single-copy homozygote
16160199	0.86	1	4	1.96	1	4: single-copy homozygote
16160255	1.17	1	13	1.98	1	13: single-copy homozygote
16190764	1.11	1	13	2.02	1	13: single-copy homozygote
16230297	1.28	1	4	2.07	1	4: single-copy homozygote
16130340	1.11	1	7	2.02	1	7: single-copy homozygote
16100728	1.30	1	10	1.88	1	10: single-copy homozygote
16100729	1.29	1	16	1.94	1	16: single-copy homozygote
16100825	1.29	1	3	1.91	1	3: single-copy homozygote
16110251	1.30	1	3	2.05	1	3: single-copy homozygote
16100789	1.24	1	3	1.98	1	3: single-copy homozygote

Transgenic Line	T_0		T_1			T_2
	Ratio (target/ref)	Estimated Copy Number	Homozygote No.	Ratio (target/ref)	Estimated Copy Number of T_0 Plant	Verification of T_1 Homozygote
16110260	1.11	1	6	2.17	1	6: single-copy homozygote
14370707	1.03	1	6	2.06	1	6: single-copy homozygote
16131030	0.90	1	3	2.10	1	3: single-copy homozygote
16260052	0.9	1	3	1.88	1	3: single-copy homozygote
16250156	0.62	1	4	2.10	1	4: single-copy homozygote
15181067	0.79	1	10	2.08	1	10: single-copy homozygote
15160352	1.13	1	8	2.43	1	8: single-copy homozygote
15181066	1.14	1	6	1.99	1	6: single-copy homozygote
15190363	1.13	1	14	2.02	1	14: single-copy homozygote
16100809	1.18	1	10	2.04	1	10: single-copy homozygote
15511024	1.04	1	2	2.01	1	2: single-copy homozygote
15370534	1.31	1	8	1.85	1	8: single-copy homozygote
16131497	1.27	1	8	2.01	1	8: single-copy homozygote
16131531	1.19	1	3	1.95	1	3: single-copy homozygote
16111626	1.28	1	7	2.12	1	7: single-copy homozygote
15410443	1.13	1	7	1.94	1	7: single-copy homozygote
15410513	1.10	1	7	1.90	1	7: single-copy homozygote
15432042	1.22	1	4	1.99	1	4: single-copy homozygote
15432016	1.31	1	13	2.04	1	13: single-copy homozygote
16161149	0.92	1	7	2.15	1	7: single-copy homozygote

Transgenic Line	T_0		T_1			T_2
	Ratio (target/ref)	Estimated Copy Number	Homozygote No.	Ratio (target/ref)	Estimated Copy Number of T_0 Plant	Verification of T_1 Homozygote
16080310	0.78	1	3	3.91	2	3: two-copy homozygote
15480621	1.21	1	4	2.14	1	4: single-copy homozygote
15431683	1.32	1	11	2.01	1	11: single-copy homozygote
15400816	1.33	1	3	1.95	1	3: single-copy homozygote
15460020	0.73	1	6	4.28	2	6: two-copy homozygote
15230227	1.01	1	16	1.99	1	16: single-copy homozygote
15240077	1.12	1	10	2.00	1	10: single-copy homozygote
15170126	1.14	1	13	2.13	1	13: single-copy homozygote
15170156	1.12	1	5	1.89	1	5: single-copy homozygote
15190151	1.11	1	3	2.07	1	3: single-copy homozygote
15190183	1.32	1	7	1.93	1	7: single-copy homozygote
15190204	1.01	1	2	3.63	2	2: two-copy homozygote
15170091	1.01	1	15	1.96	1	15: single-copy homozygote
15170205	1.05	1	8	1.93	1	8: single-copy homozygote
15520099	0.94	1	8	2.03	1	8: single-copy homozygote
15520507	1.04	1	2	1.85	1	2: single-copy homozygote
16030636	1.10	1	1	1.92	1	1: single-copy homozygote
16030675	1.10	1	15	2.04	1	15: single-copy homozygote
16030680	1.09	1	9	1.97	1	9: single-copy homozygote
15490753	1.16	1	10	2.00	1	10: single-copy homozygote

Transgenic Line	T_0		T_1			T_2
	Ratio (target/ref)	Estimated Copy Number	Homozygote No.	Ratio (target/ref)	Estimated Copy Number of T_0 Plant	Verification of T_1 Homozygote
15510457	0.71	1	3	1.97	1	3: single-copy homozygote
15520341	0.76	1	3	2.13	1	3: single-copy homozygote
16010168	1.29	1	4	1.99	1	4: single-copy homozygote
16020368	1.39	1	7	1.98	1	7: single-copy homozygote
15490696	1.18	1	13	2.00	1	13: single-copy homozygote
15520389	0.73	1	7	2.09	1	7: single-copy homozygote
16030054	1.01	1	16	1.91	1	16: single-copy homozygote
16040400	1.22	1	8	2.00	1	8: single-copy homozygote
15410419	0.89	1	12	1.96	1	12: single-copy homozygote
15410420	1.17	1	17	2.03	1	17: single-copy homozygote
15430708	1.39	1	2	1.85	1	2: single-copy homozygote
15400197	1.16	1	7	2.02	1	7: single-copy homozygote
15452160	1.34	1	10	2.03	1	10: single-copy homozygote
15441604	1.37	1	14	1.97	1	14: single-copy homozygote
16100200	1.11	1	18	2.03	1	18: single-copy homozygote
16100234	1.36	1	14	2.00	1	14: single-copy homozygote
16100584	1.30	1	4	2.04	1	4: single-copy homozygote
16100617	1.29	1	16	1.95	1	16: single-copy homozygote
16100673	1.04	1	9	1.93	1	9: single-copy homozygote
16131988	1.38	1	20	2.02	1	20: single-copy homozygote

Transgenic Line	T_0		T_1			T_2
	Ratio (target/ref)	Estimated Copy Number	Homozygote No.	Ratio (target/ref)	Estimated Copy Number of T_0 Plant	Verification of T_1 Homozygote
14430342	1.22	1	5	1.97	1	5: single-copy homozygote
14401335	1.26	1	4	2.04	1	4: single-copy homozygote
16130786	0.80	1	6	1.91	1	6: single-copy homozygote
16130844	1.21	1	11	4.18	2	11: two-copy homozygote
16090392	0.95	1	4	1.98	1	4: single-copy homozygote
16090396	1.30	1	13	2.00	1	13: single-copy homozygote
16130707	1.39	1	11	2.15	1	11: single-copy homozygote
16131870	1.25	1	2	1.94	1	2: single-copy homozygote
16111056	1.28	1	4	1.98	1	4: single-copy homozygote
16111559	1.21	1	4	1.95	1	4: single-copy homozygote
16111563	1.31	1	10	1.89	1	10: single-copy homozygote
16131936	1.07	1	6	2.12	1	6: single-copy homozygote
16140987	0.94	1	13	1.82	1	13: single-copy homozygote
16111062	1.33	1	8	2.03	1	8: single-copy homozygote
16111068	1.13	1	11	2.26	1	11: single-copy homozygote
16120541	1.28	1	6	4.19	2	6: two-copy homozygote
15290336	1.37	1	8	1.88	1	8: single-copy homozygote
15350568	1.27	1	15	2.05	1	15: single-copy homozygote
15300410	1.19	1	2	1.84	1	2: single-copy homozygote
16120504	0.73	1	6	2.12	1	6: single-copy homozygote

Transgenic Line	T_0		T_1			T_2
	Ratio (target/ref)	Estimated Copy Number	Homozygote No.	Ratio (target/ref)	Estimated Copy Number of T_0 Plant	Verification of T_1 Homozygote
16131705	1.18	1	11	2.29	1	11: single-copy homozygote
15290976	1.30	1	1	2.01	1	1: single-copy homozygote
15350337	1.15	1	4	1.93	1	4: single-copy homozygote
15320163	1.33	1	4	2.02	1	4: single-copy homozygote
15260518	1.20	1	8	2.06	1	8: single-copy homozygote
15260520	1.23	1	1	2.00	1	1: single-copy homozygote
15290541	1.32	1	5	2.20	1	5: single-copy homozygote
15290401	1.12	1	17	2.04	1	17: single-copy homozygote
15290781	1.22	1	6	1.99	1	6: single-copy homozygote
15290751	1.34	1	3	2.00	1	3: single-copy homozygote
15300974	1.17	1	15	2.02	1	15: single-copy homozygote
15290696	1.22	1	7	1.88	1	7: single-copy homozygote
15290338	1.37	1	9	2.10	1	9: single-copy homozygote
15290316	1.40	1	1	2.13	1	1: single-copy homozygote
15290305	1.28	1	4	2.15	1	4: single-copy homozygote
15330772	1.33	1	2	2.06	1	2: single-copy homozygote
15190237	1.31	1	4	4.07	2	4: two-copy homozygote
16100632	1.33	1	7	1.91	1	7: single-copy homozygote
16111329	1.10	1	11	1.95	1	11: single-copy homozygote
15290983	1.16	1	7	2.21	1	7: single-copy homozygote

Transgenic Line	T_0		T_1			T_2
	Ratio (target/ref)	Estimated Copy Number	Homozygote No.	Ratio (target/ref)	Estimated Copy Number of T_0 Plant	Verification of T_1 Homozygote
15400391	1.13	1	6	2.08	1	6: single-copy homozygote
16140775	1.06	1	3	2.44	1	3: single-copy homozygote
15350655	1.23	1	3	2.14	1	3: single-copy homozygote
15360482	1.09	1	1	1.97	1	1: single-copy homozygote
15390629	1.35	1	10	2.07	1	10: single-copy homozygote
15301224	1.28	1	1	2.18	1	1: single-copy homozygote
15400600	1.18	1	11	1.88	1	11: single-copy homozygote
16131104	1.17	1	2	2.32	1	2: single-copy homozygote
16180570	1.20	1	3	2.11	1	3: single-copy homozygote
16120174	1.21	1	2	2.03	1	2: single-copy homozygote
16230621	1.25	1	8	2.24	1	8: single-copy homozygote
15360184	1.21	1	4	2.23	1	4: single-copy homozygote
16060135	1.28	1	6	1.98	1	6: single-copy homozygote
16060154	1.11	1	5	2.00	1	5: single-copy homozygote
15300320	1.21	1	1	4.28	2	1: two-copy homozygote
15330562	1.28	1	15	1.94	1	15: single-copy homozygote
15350594	1.30	1	2	1.90	1	2: single-copy homozygote
15290404	1.36	1	1	1.90	1	1: single-copy homozygote
15311188	1.38	1	12	2.12	1	12: single-copy homozygote
15290362	1.37	1	5	1.86	1	5: single-copy homozygote

Transgenic Line	T_0		T_1			T_2
	Ratio (target/ref)	Estimated Copy Number	Homozygote No.	Ratio (target/ref)	Estimated Copy Number of T_0 Plant	Verification of T_1 Homozygote
16210250	1.37	1	2	1.90	1	2: single-copy homozygote
16220096	1.3	1	9	2.04	1	9: single-copy homozygote
16250511	0.73	1	2	2.05	1	2: single-copy homozygote
16260261	0.86	1	11	1.98	1	11: single-copy homozygote
16270395	1.09	1	11	2.05	1	11: single-copy homozygote
15290607	1.35	1	1	2.04	1	1: single-copy homozygote
15300114	1.29	1	8	2.00	1	8: single-copy homozygote
15300093	1.39	1	7	2.01	1	7: single-copy homozygote
15331067	1.11	1	2	2.17	1	2: single-copy homozygote
15330388	1.30	1	8	2.00	1	8: single-copy homozygote
15350387	1.36	1	3	1.94	1	3: single-copy homozygote
15310404	0.95	1	3	2.07	1	3: single-copy homozygote
15310438	1.19	1	6	2.00	1	6: single-copy homozygote
15301112	1.29	1	2	2.03	1	2: single-copy homozygote
15350624	1.00	1	9	1.96	1	9: single-copy homozygote
15300580	0.97	1	1	1.85	1	1: single-copy homozygote
15310959	1.13	1	3	1.98	1	3: single-copy homozygote
15330805	1.01	1	9	2.26	1	9: single-copy homozygote
15330748	1.23	1	1	2.30	1	1: single-copy homozygote
15320257	1.14	1	9	1.96	1	9: single-copy homozygote

Transgenic Line	T_0		T_1			T_2
	Ratio (target/ref)	Estimated Copy Number	Homozygote No.	Ratio (target/ref)	Estimated Copy Number of T_0 Plant	Verification of T_1 Homozygote
15380403	0.68	1	2	2.03	1	2: single-copy homozygote
16131178	1.35	1	8	2.03	1	8: single-copy homozygote
16131194	1.18	1	15	1.98	1	15: single-copy homozygote
16131172	0.91	1	5	2.19	1	5: single-copy homozygote
16200782	1.37	1	8	1.88	1	8: single-copy homozygote
16120443	1.28	1	8	2.06	1	8: single-copy homozygote
16131834	1.17	1	5	2.06	1	5: single-copy homozygote
16121138	1.06	1	3	1.98	1	3: single-copy homozygote
16121139	1.02	1	8	2.08	1	8: single-copy homozygote
16121145	1.19	1	14	1.97	1	14: single-copy homozygote
16141253	1.32	1	9	2.02	1	9: single-copy homozygote
16120402	1.31	1	10	2.13	1	10: single-copy homozygote
16131762	1.28	1	6	2.25	1	6: single-copy homozygote
16180502	1.28	1	1	2.03	1	1: single-copy homozygote
16180504	0.92	1	14	1.98	1	14: single-copy homozygote
15390183	1.10	1	5	1.94	1	5: single-copy homozygote
15380616	0.46	0	8	2.01	1	8: single-copy homozygote
16040397	0.77	1	7	2.28	1	7: single-copy homozygote
16050537	1.36	1	6	2.08	1	6: single-copy homozygote
16060145	1.13	1	3	2.06	1	3: single-copy homozygote

Transgenic Line	T_0		T_1			T_2
	**Ratio (target/ref)**	**Estimated Copy Number**	**Homozygote No.**	**Ratio (target/ref)**	**Estimated Copy Number of T_0 Plant**	**Verification of T_1 Homozygote**
16060157	1.13	1	6	2.01	1	6: single-copy homozygote
15410595	1.19	1	3	1.99	1	3: single-copy homozygote
15461156	1.34	1	3	1.98	1	3: single-copy homozygote
15400328	1.30	1	22	2.09	1	22: single-copy homozygote
15441532	1.37	1	3	2.02	1	3: single-copy homozygote
15441556	1.18	1	3	2.19	1	3: single-copy homozygote
15520495	0.89	1	6	1.98	1	6: single-copy homozygote
15430141	1.33	1	3	2.01	1	3: single-copy homozygote
15481379	1.16	1	17	1.91	1	17: single-copy homozygote
15440117	1.31	1	5	2.29	1	5: single-copy homozygote
15440137	1.17	1	11	2.00	1	11: single-copy homozygote
15431089	1.13	1	21	1.91	1	21: single-copy homozygote
15470109	1.09	1	12	2.05	1	12: single-copy homozygote
16010268	1.23	1	3	2.17	1	3: single-copy homozygote
15360150	1.18	1	5	1.97	1	5: single-copy homozygote
15340116	1.15	1	4	2.18	1	4: single-copy homozygote
15330648	1.32	1	17	2.02	1	17: single-copy homozygote
15340090	1.34	1	5	2.27	1	5: single-copy homozygote
15340060	1.21	1	15	2.20	1	15: single-copy homozygote
16060190	1.10	1	11	2.15	1	11: single-copy homozygote

Transgenic Line	T_0		T_1			T_2
	**Ratio (target/ref)**	**Estimated Copy Number**	**Homozygote No.**	**Ratio (target/ref)**	**Estimated Copy Number of T_0 Plant**	**Verification of T_1 Homozygote**
16070073	1.32	1	7	2.27	1	7: single-copy homozygote
16110781	1.30	1	7	1.96	1	7: single-copy homozygote
16131099	1.19	1	9	1.92	1	9: single-copy homozygote
16140744	1.13	1	11	2.00	1	11: single-copy homozygote
16100476	1.21	1	3	1.91	1	3: single-copy homozygote
16131139	1.33	1	1	2.12	1	1: single-copy homozygote
16100627	1.25	1	7	2.28	1	7: single-copy homozygote
16100629	0.87	1	4	2.26	1	4: single-copy homozygote
16100653	1.31	1	7	2.37	1	7: single-copy homozygote
16111254	1.24	1	12	2.08	1	12: single-copy homozygote
16111255	1.12	1	9	2.16	1	9: single-copy homozygote
16120045	1.09	1	21	2.02	1	21: single-copy homozygote
16110474	1.03	1	4	1.95	1	4: single-copy homozygote
16121319	1.38	1	13	1.92	1	13: single-copy homozygote
16190251	1.24	1	17	2.12	1	17: single-copy homozygote
16200459	1.15	1	10	1.92	1	10: single-copy homozygote
16210198	1.07	1	11	1.88	1	11: single-copy homozygote
16130269	1.36	1	9	1.90	1	9: single-copy homozygote
16140080	1.13	1	2	2.18	1	2: single-copy homozygote
16100707	1.15	1	7	2.17	1	7: single-copy homozygote

Transgenic Line	T_0		T_1			T_2
	Ratio (target/ref)	Estimated Copy Number	Homozygote No.	Ratio (target/ref)	Estimated Copy Number of T_0 Plant	Verification of T_1 Homozygote
16180321	1.04	1	6	1.98	1	6: single-copy homozygote
16140134	1.34	1	2	2.19	1	2: single-copy homozygote
16160528	1.10	1	18	1.96	1	18: single-copy homozygote
16121358	1.18	1	4	2.15	1	4: single-copy homozygote
16100842	1.10	1	7	2.39	1	7: single-copy homozygote
16130810	0.75	1	5	2.04	1	5: single-copy homozygote
16140424	1.28	1	16	2.30	1	16: single-copy homozygote
16131864	1.13	1	4	1.92	1	4: single-copy homozygote
16131868	1.20	1	24	1.98	1	24: single-copy homozygote
16141228	1.35	1	12	2.32	1	12: single-copy homozygote
16160857	1.32	1	3	1.98	1	3: single-copy homozygote
16121155	1.04	1	13	2.01	1	13: single-copy homozygote
16160901	1.28	1	1	2.12	1	1: single-copy homozygote
16140277	1.36	1	3	1.99	1	3: single-copy homozygote
16140363	1.00	1	16	1.92	1	16: single-copy homozygote
16190017	1.27	1	18	2.03	1	18: single-copy homozygote
16141011	0.96	1	5	1.99	1	5: single-copy homozygote
16100768	1.35	1	4	2.04	1	4: single-copy homozygote
16121435	1.37	1	17	1.96	1	17: single-copy homozygote
16200389	1.29	1	10	2.03	1	10: single-copy homozygote

Transgenic Line	T_0		T_1			T_2
	Ratio (target/ref)	Estimated Copy Number	Homozygote No.	Ratio (target/ref)	Estimated Copy Number of T_0 Plant	Verification of T_1 Homozygote
16210185	1.10	1	1	2.09	1	1: single-copy homozygote
16190121	1.11	1	17	2.03	1	17: single-copy homozygote
16190496	1.36	1	1	2.01	1	1: single-copy homozygote
16190507	1.18	1	9	2.03	1	9: single-copy homozygote
16200158	0.93	1	13	2.02	1	13: single-copy homozygote
16210311	0.66	1	3	1.97	1	3: single-copy homozygote
16141076	1.02	1	4	2.03	1	4: single-copy homozygote
16110228	1.20	1	16	1.97	1	16: single-copy homozygote
16200270	1.10	1	12	1.98	1	12: single-copy homozygote
16210368	1.14	1	15	2.00	1	15: single-copy homozygote
16230618	0.93	1	1	1.84	1	1: single-copy homozygote
16260086	0.98	1	11	1.86	1	11: single-copy homozygote
16160235	1.12	1	15	1.95	1	15: single-copy homozygote
16160725	1.26	1	9	1.95	1	9: single-copy homozygote
16200469	1.02	1	11	1.95	1	11: single-copy homozygote
16200521	1.27	1	1	1.98	1	1: single-copy homozygote
16200531	1.06	1	10	2.08	1	10: single-copy homozygote
16200549	0.99	1	8	1.91	1	8: single-copy homozygote
16200544	1.26	1	2	2.01	1	2: single-copy homozygote
16200552	1.19	1	4	2.02	1	4: single-copy homozygote

**Transgenic Line**	**T_0**		**T_1**			**T_2**
	**Ratio (target/ref)**	**Estimated Copy Number**	**Homozygote No.**	**Ratio (target/ref)**	**Estimated Copy Number of T_0 Plant**	**Verification of T_1 Homozygote**
16210112	1.08	1	4	2.03	1	4: single-copy homozygote
16210113	1.1	1	5	1.92	1	5: single-copy homozygote
16210140	1.28	1	8	1.87	1	8: single-copy homozygote
16240176	1.31	1	1	1.93	1	1: single-copy homozygote
16170009	1.31	1	1	1.89	1	1: single-copy homozygote
16200290	0.99	1	1	1.97	1	1: single-copy homozygote
16200291	1.10	1	12	1.91	1	12: single-copy homozygote
15090254	1.05	1	12	1.97	1	12: single-copy homozygote
16200298	1.04	1	1	1.88	1	1: single-copy homozygote
15250362	1.38	1	8	2.24	1	8: single-copy homozygote
15270364	1.38	1	8	2.12	1	8: single-copy homozygote
16190821	1.33	1	11	1.84	1	11: single-copy homozygote
16200679	1.18	1	3	1.99	1	3: single-copy homozygote
16240548	1.34	1	3	1.96	1	3: single-copy homozygote
16230561	1.17	1	4	2.08	1	4: single-copy homozygote
16270176	1.16	1	7	2.13	1	7: single-copy homozygote
16270204	0.98	1	13	2.11	1	13: single-copy homozygote
16270205	0.94	1	12	1.96	1	12: single-copy homozygote
16270260	1.2	1	10	1.89	1	10: single-copy homozygote
16200935	1.19	1	2	1.99	1	2: single-copy homozygote

Transgenic Line	T_0		T_1			T_2
	Ratio (target/ref)	Estimated Copy Number	Homozygote No.	Ratio (target/ref)	Estimated Copy Number of T_0 Plant	Verification of T_1 Homozygote
16220373	1.28	1	13	2.03	1	13: single-copy homozygote
16210512	1.13	1	12	1.93	1	12: single-copy homozygote
16250490	1.27	1	8	2.50	1	8: single-copy homozygote
16220228	1.13	1	10	1.91	1	10: single-copy homozygote
16250234	0.84	1	3	1.92	1	3: single-copy homozygote
16270187	1.15	1	10	1.91	1	10: single-copy homozygote
16270295	1.03	1	2	1.83	1	2: single-copy homozygote
16270297	1.07	1	8	1.93	1	8: single-copy homozygote
16200375	1.10	1	16	2.00	1	16: single-copy homozygote
16240247	1.13	1	1	2.09	1	1: single-copy homozygote
14360524	1.16	1	10	2.15	1	10: single-copy homozygote
14370830	1.22	1	12	2.04	1	12: single-copy homozygote
16230356	0.99	1	2	1.86	1	2: single-copy homozygote
16230374	1.27	1	13	1.97	1	13: single-copy homozygote
16111202	1.28	1	1	1.92	1	1: single-copy homozygote
16080297	1.37	1	15	2.08	1	15: single-copy homozygote
16080318	1.07	1	5	2.02	1	5: single-copy homozygote
16131200	1.08	1	16	1.99	1	16: single-copy homozygote
16100565	1.18	1	15	1.98	1	15: single-copy homozygote
14320376	1.37	1	23	1.97	1	23: single-copy homozygote

Transgenic Line	T_0		T_1			T_2
	Ratio (target/ref)	Estimated Copy Number	Homozygote No.	Ratio (target/ref)	Estimated Copy Number of T_0 Plant	Verification of T_1 Homozygote
16080491	1.37	1	1	2.04	1	1: single-copy homozygote
16090128	1.33	1	15	1.81	1	15: single-copy homozygote
16100055	1.39	1	1	1.79	1	1: single-copy homozygote
14310131	1.27	1	3	2.06	1	3: single-copy homozygote
14320250	1.38	1	1	2.04	1	1: single-copy homozygote
16090021	0.61	1	16	1.97	1	16: single-copy homozygote
16090033	1.36	1	5	2.00	1	5: single-copy homozygote
16090042	1.29	1	15	1.83	1	15: single-copy homozygote
16100481	0.85	1	11	2.07	1	11: single-copy homozygote
16100491	1.19	1	9	1.98	1	9: single-copy homozygote
16100562	1.14	1	16	2.01	1	16: single-copy homozygote
16140648	1.06	1	13	2.07	1	13: single-copy homozygote
16090299	1.25	1	11	1.99	1	11: single-copy homozygote
16090591	1.26	1	3	2.07	1	3: single-copy homozygote
16111332	1.18	1	11	4.12	2	11: two-copy homozygote
15081742	1.23	1	4	2.04	1	4: single-copy homozygote
16141230	1.39	1	5	2.19	1	5: single-copy homozygote
16141243	1.28	1	10	2.09	1	10: single-copy homozygote
16120550	0.65	1	1	1.95	1	1: single-copy homozygote
16190725	1.30	1	1	2.02	1	1: single-copy homozygote

Transgenic Line	T_0		T_1			T_2
	Ratio (target/ref)	Estimated Copy Number	Homozygote No.	Ratio (target/ref)	Estimated Copy Number of T_0 Plant	Verification of T_1 Homozygote
15100420	1.13	1	10	2.15	1	10: single-copy homozygote
15111048	1.10	1	8	2.03	1	8: single-copy homozygote
15120050	1.30	1	9	2.02	1	9: single-copy homozygote
15120449	1.12	1	3	1.99	1	3: single-copy homozygote
15120463	1.14	1	18	2.01	1	18: single-copy homozygote
15250193	1.28	1	3	2.00	1	3: single-copy homozygote
15290460	1.32	1	1	3.92	2	1: two-copy homozygote
15290987	1.22	1	1	2.18	1	1: single-copy homozygote
15260496	1.12	1	3	1.95	1	3: single-copy homozygote
15220709	1.36	1	2	2.14	1	2: single-copy homozygote
16121617	1.25	1	5	1.95	1	5: single-copy homozygote
15300343	1.29	1	1	2.09	1	1: single-copy homozygote
15300396	1.40	1	12	2.05	1	12: single-copy homozygote
15290413	1.38	1	12	1.91	1	12: single-copy homozygote
15290267	0.72	1	11	2.06	1	11: single-copy homozygote
15280502	1.07	1	2	2.11	1	2: single-copy homozygote
15290251	1.26	1	3	2.13	1	3: single-copy homozygote
15350510	1.22	1	2	1.95	1	2: single-copy homozygote
15310732	1.20	1	2	1.92	1	2: single-copy homozygote
15260235	1.11	1	1	2.19	1	1: single-copy homozygote

Transgenic Line	T_0		T_1			T_2
	Ratio (target/ref)	Estimated Copy Number	Homozygote No.	Ratio (target/ref)	Estimated Copy Number of T_0 Plant	Verification of T_1 Homozygote
15320998	0.98	1	1	2.00	1	1: single-copy homozygote
15260319	1.19	1	3	2.02	1	3: single-copy homozygote
15250038	1.33	1	11	2.02	1	11: single-copy homozygote
16121020	1.29	1	3	2.33	1	3: single-copy homozygote
15321073	1.19	1	11	2.13	1	11: single-copy homozygote
15300120	1.22	1	7	2.09	1	7: single-copy homozygote
15290127	1.30	1	7	1.97	1	7: single-copy homozygote
15300028	1.29	1	6	1.98	1	6: single-copy homozygote
15440929	1.20	1	7	2.22	1	7: single-copy homozygote
15462193	1.19	1	7	2.28	1	7: single-copy homozygote
15462220	0.55	1	6	2.09	1	6: single-copy homozygote
15470665	1.14	1	1	2.32	1	1: single-copy homozygote
15492234	1.06	1	7	1.80	1	7: single-copy homozygote
15310948	1.32	1	2	2.10	1	2: single-copy homozygote
15310559	1.17	1	7	1.95	1	7: single-copy homozygote
15310805	1.22	1	12	2.12	1	12: single-copy homozygote
15310913	1.07	1	2	2.22	1	2: single-copy homozygote
15390095	0.93	1	11	2.07	1	11: single-copy homozygote
15360661	1.23	1	13	2.02	1	13: single-copy homozygote
15280291	1.37	1	2	2.23	1	2: single-copy homozygote

Transgenic Line	T_0		T_1			T_2
	Ratio (target/ref)	Estimated Copy Number	Homozygote No.	Ratio (target/ref)	Estimated Copy Number of T_0 Plant	Verification of T_1 Homozygote
15310518	0.74	1	9	2.10	1	9: single-copy homozygote
15310695	1.07	1	3	1.96	1	3: single-copy homozygote
15290035	1.19	1	2	2.03	1	2: single-copy homozygote
15380768	1.06	1	1	1.97	1	1: single-copy homozygote
15350744	1.09	1	1	2.39	1	1: single-copy homozygote
15300825	1.08	1	4	1.97	1	4: single-copy homozygote
15320975	0.96	1	1	2.56	1	1: two-copy homozygote
15311238	1.38	1	14	2.12	1	14: single-copy homozygote
15350447	0.56	1	2	3.96	2	2: two-copy homozygote
15350608	0.75	1	4	2.20	1	4: single-copy homozygote
16100810	1.05	1	1	2.37	1	1: single-copy homozygote
16100730	1.16	1	3	2.01	1	3: single-copy homozygote
16110049	1.19	1	1	1.89	1	1: single-copy homozygote
16110050	1.20	1	10	2.11	1	10: single-copy homozygote
16190925	1.31	1	1	1.95	1	1: single-copy homozygote
15310099	1.04	1	5	2.30	1	5: single-copy homozygote
15311117	1.25	1	2	2.30	1	2: single-copy homozygote
15330585	1.26	1	11	2.03	1	11: single-copy homozygote
16080004	1.28	1	14	1.96	1	14: single-copy homozygote
16080192	1.24	1	13	2.00	1	13: single-copy homozygote

Transgenic Line	T_0		T_1			T_2
	Ratio (target/ref)	Estimated Copy Number	Homozygote No.	Ratio (target/ref)	Estimated Copy Number of T_0 Plant	Verification of T_1 Homozygote
16160147	0.88	1	8	1.96	1	8: single-copy homozygote
16120723	1.32	1	7	2.30	1	7: single-copy homozygote
15370355	1.16	1	12	2.18	1	12: single-copy homozygote
15451703	1.16	1	14	2.36	1	14: single-copy homozygote
15380668	0.92	1	8	2.00	1	8: single-copy homozygote
15400040	1.28	1	17	2.16	1	17: single-copy homozygote
15400829	1.20	1	3	2.13	1	3: single-copy homozygote
15420348	1.40	1	1	2.03	1	1: single-copy homozygote
15420349	1.33	1	12	2.01	1	12: single-copy homozygote
15431649	1.05	1	8	2.03	1	8: single-copy homozygote
15400479	1.24	1	8	1.99	1	8: single-copy homozygote
15420381	1.31	1	7	1.84	1	7: single-copy homozygote
15410301	1.06	1	4	1.97	1	4: single-copy homozygote
15380659	1.24	1	2	1.83	1	2: single-copy homozygote
15410249	1.14	1	13	2.31	1	13: single-copy homozygote
15440164	1.34	1	1	1.92	1	1: single-copy homozygote
15380417	1.35	1	4	2.20	1	4: single-copy homozygote
15390636	1.34	1	10	2.15	1	10: single-copy homozygote
15390688	1.17	1	20	2.13	1	20: single-copy homozygote
15400357	1.17	1	12	2.14	1	12: single-copy homozygote

Transgenic Line	T_0		T_1			T_2
	**Ratio (target/ref)**	**Estimated Copy Number**	**Homozygote No.**	**Ratio (target/ref)**	**Estimated Copy Number of T_0 Plant**	**Verification of T_1 Homozygote**
16150224	1.33	1	10	2.23	1	10: single-copy homozygote
16150225	1.15	1	4	2.04	1	4: single-copy homozygote
16170257	1.13	1	1	2.09	1	1: single-copy homozygote
16120118	1.21	1	3	2.04	1	3: single-copy homozygote
16090501	1.34	1	15	2.00	1	15: single-copy homozygote
16130673	1.06	1	7	2.12	1	7: single-copy homozygote
16130651	1.08	1	4	2.00	1	4: single-copy homozygote
16140338	0.96	1	11	1.96	1	11: single-copy homozygote
16141425	1.02	1	8	2.03	1	8: single-copy homozygote
16090459	1.25	1	7	1.84	1	7: single-copy homozygote
16120696	1.35	1	3	2.00	1	3: single-copy homozygote
16120726	1.37	1	1	2.03	1	1: single-copy homozygote
16141095	1.17	1	5	2.00	1	5: single-copy homozygote
16110439	1.17	1	4	1.92	1	4: single-copy homozygote
16121298	1.39	1	7	2.17	1	7: single-copy homozygote
16170465	1.15	1	11	2.01	1	11: single-copy homozygote
16170424	0.99	1	9	2.04	1	9: single-copy homozygote
16200143	1.00	1	12	1.95	1	12: single-copy homozygote
16200176	1.05	1	4	2.28	1	4: single-copy homozygote
16200211	1.08	1	13	2.09	1	13: single-copy homozygote

Transgenic Line	T_0		T_1			T_2
	**Ratio (target/ref)**	**Estimated Copy Number**	**Homozygote No.**	**Ratio (target/ref)**	**Estimated Copy Number of T_0 Plant**	**Verification of T_1 Homozygote**
16120676	1.37	1	10	2.03	1	10: single-copy homozygote
16100352	1.22	1	6	1.94	1	6: single-copy homozygote
16100393	1.40	1	3	2.05	1	3: single-copy homozygote
14401016	1.02	1	15	1.93	1	15: single-copy homozygote
16190031	1.06	1	1	2.12	1	1: single-copy homozygote
16200200	1.06	1	10	1.97	1	10: single-copy homozygote
16121454	1.18	1	11	2.15	1	11: single-copy homozygote
16121540	1.15	1	5	2.45	1	5: single-copy homozygote
16160651	1.18	1	8	2.07	1	8: single-copy homozygote
16190262	1.38	1	8	2.49	1	8: single-copy homozygote
16210050	1.2	1	9	2.02	1	9: single-copy homozygote
16140262	1.26	1	10	2.06	1	10: single-copy homozygote
16141114	1.26	1	1	1.99	1	1: single-copy homozygote
16180163	1.10	1	6	2.07	1	6: single-copy homozygote
16130217	1.04	1	6	1.95	1	6: single-copy homozygote
16180275	1.16	1	5	2.47	1	5: single-copy homozygote
16160267	1.05	1	11	2.03	1	11: single-copy homozygote
16160269	1.10	1	4	1.94	1	4: single-copy homozygote
16160739	1.07	1	5	2.09	1	5: single-copy homozygote
16170360	0.97	1	6	2.28	1	6: single-copy homozygote

Transgenic Line	T_0		T_1			T_2
	Ratio (target/ref)	Estimated Copy Number	Homozygote No.	Ratio (target/ref)	Estimated Copy Number of T_0 Plant	Verification of T_1 Homozygote
15160925	1.22	1	10	2.04	1	10: single-copy homozygote
15240012	1.31	1	3	1.98	1	3: single-copy homozygote
16130662	1.11	1	7	3.92	2	7: two-copy homozygote
16160053	0.89	1	7	2.05	1	7: single-copy homozygote
16160119	1.14	1	4	2.02	1	4: single-copy homozygote
16121419	1.36	1	13	2.09	1	13: single-copy homozygote
16190235	0.95	1	4	1.99	1	4: single-copy homozygote
16190260	1.31	1	10	1.98	1	10: single-copy homozygote
16200526	0.62	1	12	2.03	1	12: single-copy homozygote
16200535	1.37	1	3	2.00	1	3: single-copy homozygote
16200537	1.09	1	6	2.09	1	6: single-copy homozygote
16100764	1.12	1	7	2.00	1	7: single-copy homozygote
16200491	1.31	1	6	2.08	1	6: single-copy homozygote
16200504	1.03	1	8	1.93	1	8: single-copy homozygote
16200566	1.07	1	3	1.99	1	3: single-copy homozygote
16200582	1.30	1	4	2.11	1	4: single-copy homozygote
16180197	1.24	1	3	2.01	1	3: single-copy homozygote
16200503	0.96	1	6	1.95	1	6: single-copy homozygote
16210163	1.28	1	3	2.02	1	3: single-copy homozygote
16140917	1.04	1	6	1.99	1	6: single-copy homozygote

Transgenic Line	T_0		T_1			T_2
	Ratio (target/ref)	Estimated Copy Number	Homozygote No.	Ratio (target/ref)	Estimated Copy Number of T_0 Plant	Verification of T_1 Homozygote
16140928	1.18	1	8	2.13	1	8: single-copy homozygote
16200261	1.02	1	12	1.95	1	12: single-copy homozygote
16150106	1.27	1	4	2.00	1	4: single-copy homozygote
16160417	0.81	1	3	2.23	1	3: single-copy homozygote
16220227	0.64	1	5	1.98	1	5: single-copy homozygote
14170029	0.95	1	7	2.22	1	7: single-copy homozygote
16200645	1.15	1	1	2.25	1	1: single-copy homozygote
16210523	1.08	1	6	2.03	1	6: single-copy homozygote
16220283	1.28	1	8	2.16	1	8: single-copy homozygote
16180316	1.13	1	7	1.96	1	7: single-copy homozygote
16200819	1.10	1	7	2.17	1	7: single-copy homozygote
16210026	1.18	1	5	1.80	1	5: single-copy homozygote
16210135	1.28	1	1	2.04	1	1: single-copy homozygote
16210136	1.36	1	8	1.93	1	8: single-copy homozygote
16210264	1.03	1	5	1.94	1	5: single-copy homozygote
16210083	1.37	1	15	1.95	1	15: single-copy homozygote
16180233	1.07	1	6	2.01	1	6: single-copy homozygote
16200473	0.86	1	7	2.02	1	7: single-copy homozygote
16200478	1.17	1	13	2.03	1	13: single-copy homozygote
16210085	1.40	1	11	2.17	1	11: single-copy homozygote

Transgenic Line	T_0		T_1			T_2
	Ratio (target/ref)	Estimated Copy Number	Homozygote No.	Ratio (target/ref)	Estimated Copy Number of T_0 Plant	Verification of T_1 Homozygote
16210261	1.39	1	1	1.97	1	1: single-copy homozygote
16240124	1.15	1	1	1.83	1	1: single-copy homozygote
14260059	1.14	1	7	2.09	1	7: single-copy homozygote
14290236	0.70	1	6	2.33	1	6: single-copy homozygote
15441154	1.30	1	2	2.20	1	2: single-copy homozygote
15441149	1.31	1	5	1.99	1	5: single-copy homozygote
15450728	1.25	1	7	5.71	$\geqslant 3$	7: multi-copy
15461291	1.31	1	4	2.02	1	4: single-copy homozygote
14330445	1.19	1	11	2.04	1	11: single-copy homozygote
16160714	1.10	1	3	2.10	1	3: single-copy homozygote
16230233	1.19	1	8	2.30	1	8: single-copy homozygote
16270533	0.78	1	14	2.15	1	14: single-copy homozygote
15450628	1.08	1	3	2.01	1	3: single-copy homozygote
15461455	1.20	1	11	2.08	1	11: single-copy homozygote
15461460	1.28	1	17	2.17	1	17: single-copy homozygote
15470278	1.02	1	12	2.05	1	12: single-copy homozygote
15480634	1.04	1	1	2.08	1	1: single-copy homozygote
15480114	1.26	1	2	2.03	1	2: single-copy homozygote
15480115	0.97	1	1	1.94	1	1: single-copy homozygote
15480305	1.21	1	8	1.98	1	8: single-copy homozygote

Transgenic Line	T_0		T_1			T_2
	**Ratio (target/ref)**	**Estimated Copy Number**	**Homozygote No.**	**Ratio (target/ref)**	**Estimated Copy Number of T_0 Plant**	**Verification of T_1 Homozygote**
15490082	1.28	1	2	1.87	1	2: single-copy homozygote
16050466	1.18	1	2	2.21	1	2: single-copy homozygote
15140507	0.99	1	7	2.11	1	7: single-copy homozygote
15120335	1.08	1	9	2.20	1	9: single-copy homozygote
16131458	1.24	1	7	1.97	1	7: single-copy homozygote
16210003	1.40	1	6	2.02	1	6: single-copy homozygote
15510844	1.40	1	11	1.99	1	11: single-copy homozygote
15440877	1.18	1	7	2.10	1	7: single-copy homozygote
15470692	1.24	1	12	2.07	1	12: single-copy homozygote
15492027	1.00	1	13	2.00	1	13: single-copy homozygote
15490309	1.36	1	10	1.94	1	10: single-copy homozygote
15451468	1.23	1	25	2.01	1	25: single-copy homozygote
15451483	1.36	1	8	1.88	1	8: single-copy homozygote
15481948	1.33	1	8	1.98	1	8: single-copy homozygote
15060269	1.07	1	12	2.28	1	12: single-copy homozygote
15100522	1.40	1	1	2.03	1	1: single-copy homozygote
14410350	1.16	1	15	1.97	1	15: single-copy homozygote
16140727	1.10	1	5	2.29	1	5: single-copy homozygote
16100773	1.07	1	11	1.95	1	11: single-copy homozygote
16110524	1.19	1	10	1.93	1	10: single-copy homozygote

Transgenic Line	T_0		T_1			T_2
	Ratio (target/ref)	Estimated Copy Number	Homozygote No.	Ratio (target/ref)	Estimated Copy Number of T_0 Plant	Verification of T_1 Homozygote
16121569	1.33	1	2	2.01	1	2: single-copy homozygote
16111137	1.24	1	6	2.20	1	6: single-copy homozygote
14320492	0.88	1	10	1.96	1	10: single-copy homozygote
16150035	1.34	1	14	2.00	1	14: single-copy homozygote
16100493	1.17	1	4	2.01	1	4: single-copy homozygote
16111073	0.79	1	1	2.36	1	1: single-copy homozygote
16111467	0.75	1	5	2.05	1	5: single-copy homozygote
14371182	1.37	1	7	4.01	2	7: two-copy homozygote
15451720	1.32	1	19	2.16	1	19: single-copy homozygote
15451940	1.19	1	9	1.96	1	9: single-copy homozygote
15452080	1.07	1	1	2.26	1	1: single-copy homozygote
15140545	1.32	1	3	3.88	2	3: two-copy homozygote
15160807	1.09	1	11	2.24	1	11: single-copy homozygote
15431827	1.35	1	6	1.87	1	6: single-copy homozygote
16180354	1.38	1	3	2.15	1	3: single-copy homozygote
14340273	1.05	1	6	2.08	1	6: single-copy homozygote
17090296	0.88	1	12	2.14	1	12: single-copy homozygote
16131344	1.02	1	5	2.00	1	5: single-copy homozygote
14340463	1.18	1	19	2.04	1	19: single-copy homozygote
16230088	1.33	1	13	4.00	2	13: two-copy homozygote

Transgenic Line	T_0		T_1			T_2
	Ratio (target/ref)	Estimated Copy Number	Homozygote No.	Ratio (target/ref)	Estimated Copy Number of T_0 Plant	Verification of T_1 Homozygote
16230130	1.21	1	2	2.05	1	2: single-copy homozygote
17100238	1.19	1	1	2.43	1	1: single-copy homozygote
17100265	1	1	1	2.15	1	1: single-copy homozygote
17100270	1.29	1	5	2.04	1	5: single-copy homozygote
17100384	1.28	1	2	2.35	1	2: single-copy homozygote
16210631	1.11	1	1	2.16	1	1: single-copy homozygote
16210636	1.39	1	5	2.25	1	5: single-copy homozygote
16230580	1.29	1	10	2.05	1	10: single-copy homozygote
16230595	1.09	1	8	2.23	1	8: single-copy homozygote
16190856	1.27	1	8	2.03	1	8: single-copy homozygote
16270491	1.15	1	11	1.93	1	11: single-copy homozygote
16210131	1.26	1	2	1.87	1	2: single-copy homozygote
16210189	1.22	1	4	2.28	1	4: single-copy homozygote
16240112	1.32	1	18	2.00	1	18: single-copy homozygote
16240172	1.35	1	7	1.89	1	7: single-copy homozygote
16210373	1.17	1	1	2.03	1	1: single-copy homozygote
16220190	1.35	1	4	2.10	1	4: single-copy homozygote
16220200	1.29	1	2	1.94	1	2: single-copy homozygote
16270141	0.7	1	2	1.87	1	2: single-copy homozygote
17010436	1.18	1	1	2.35	1	1: single-copy homozygote

Transgenic Line	T_0		T_1			T_2
	Ratio (target/ref)	Estimated Copy Number	Homozygote No.	Ratio (target/ref)	Estimated Copy Number of T_0 Plant	Verification of T_1 Homozygote
17010437	1.16	1	2	2.00	1	2: single-copy homozygote
17010446	1.29	1	14	1.96	1	14: single-copy homozygote
14401028	1.06	1	6	2.06	1	6: single-copy homozygote
14420761	1.03	1	1	2.23	1	1: single-copy homozygote
15461207	0.92	1	1	2.01	1	1: single-copy homozygote
15390514	1.40	1	6	1.94	1	6: single-copy homozygote
15400729	1.25	1	16	2.00	1	16: single-copy homozygote
16160870	1.27	1	6	1.87	1	6: single-copy homozygote
15450477	1.17	1	9	2.16	1	9: single-copy homozygote
15450499	0.87	1	7	2.23	1	7: single-copy homozygote
15450516	1.39	1	9	2.07	1	9: single-copy homozygote
15450517	1.15	1	8	1.87	1	8: single-copy homozygote
15510883	1.39	1	14	2.31	1	14: single-copy homozygote
16131042	1.30	1	6	2.02	1	6: single-copy homozygote
16260065	0.97	1	8	1.84	1	8: single-copy homozygote
16131395	1.24	1	10	1.98	1	10: single-copy homozygote
16160727	1.24	1	4	2.19	1	4: single-copy homozygote
16131303	1.24	1	10	1.97	1	10: single-copy homozygote
16190768	1.32	1	6	1.93	1	6: single-copy homozygote
17140215	1.13	1	15	1.98	1	15: single-copy homozygote

Transgenic Line	T_0		T_1			T_2
	Ratio (target/ref)	Estimated Copy Number	Homozygote No.	Ratio (target/ref)	Estimated Copy Number of T_0 Plant	Verification of T_1 Homozygote
17110078	1.07	1	5	1.95	1	5: single-copy homozygote
17120280	1.07	1	7	2.09	1	7: single-copy homozygote
16160845	1.09	1	14	2.02	1	14: single-copy homozygote
17070411	1.21	1	2	2.11	1	2: single-copy homozygote
17080075	1.13	1	10	2.04	1	10: single-copy homozygote
16230641	1.3	1	2	1.89	1	2: single-copy homozygote
16230642	1.07	1	15	2.03	1	15: single-copy homozygote
17150584	0.79	1	13	1.86	1	13: single-copy homozygote
16200797	1.15	1	2	1.85	1	2: single-copy homozygote
16210369	1.14	1	1	2.30	1	1: single-copy homozygote
16170231	1.08	1	15	2.34	1	15: single-copy homozygote
14430021	1.21	1	9	1.95	1	9: single-copy homozygote
15190004	1.08	1	4	2.10	1	4: single-copy homozygote
15510450	1.12	1	11	2.06	1	11: single-copy homozygote
16050118	0.98	1	14	2.42	1	14: single-copy homozygote
17030232	1.05	1	7	2.03	1	7: single-copy homozygote
15470900	1.08	1	8	1.99	1	8: single-copy homozygote
15492546	1.28	1	8	1.85	1	8: single-copy homozygote
16020259	1.02	1	19	2.08	1	19: single-copy homozygote
16030120	1.22	1	10	2.02	1	10: single-copy homozygote

Transgenic Line	T_0		T_1			T_2
	Ratio (target/ref)	Estimated Copy Number	Homozygote No.	Ratio (target/ref)	Estimated Copy Number of T_0 Plant	Verification of T_1 Homozygote
17150481	0.96	1	17	2.10	1	17: single-copy homozygote
16110404	1.40	1	13	2.22	1	13: single-copy homozygote
15491810	1.29	1	15	1.99	1	15: single-copy homozygote
17070031	0.85	1	2	2.04	1	2: single-copy homozygote
15110173	1.30	1	7	1.91	1	7: single-copy homozygote
15480677	1.34	1	3	1.84	1	3: single-copy homozygote
15520527	0.60	1	6	2.03	1	6: single-copy homozygote
15520550	0.92	1	7	1.92	1	7: single-copy homozygote
16030262	1.30	1	1	1.99	1	1: single-copy homozygote
15451861	1.11	1	5	1.96	1	5: single-copy homozygote
15491927	1.02	1	5	2.23	1	5: single-copy homozygote
15460560	1.38	1	1	2.02	1	1: single-copy homozygote
17150517	1.25	1	13	2.06	1	13: single-copy homozygote
16260082	1.1	1	4	1.99	1	4: single-copy homozygote
16280295	1.36	1	8	2.00	1	8: single-copy homozygote
16250127	0.86	1	10	2.01	1	10: single-copy homozygote
17150675	1.26	1	2	2.04	1	2: single-copy homozygote
17150682	1.04	1	8	1.91	1	8: single-copy homozygote
17150684	1.06	1	8	1.82	1	8: single-copy homozygote
17150704	1.16	1	4	2.05	1	4: single-copy homozygote

Transgenic Line	T_0		T_1			T_2
	**Ratio (target/ref)**	**Estimated Copy Number**	**Homozygote No.**	**Ratio (target/ref)**	**Estimated Copy Number of T_0 Plant**	**Verification of T_1 Homozygote**
15450621	1.09	1	2	1.85	1	2: single-copy homozygote
15190093	1.24	1	8	1.85	1	8: single-copy homozygote
15150545	1.05	1	17	1.88	1	17: single-copy homozygote
15450818	1.06	1	11	2.09	1	11: single-copy homozygote
15520186	0.78	1	2	3.92	2	2: two-copy homozygote
16030630	1.28	1	19	2.01	1	19: single-copy homozygote
16060256	1.14	1	9	1.88	1	9: single-copy homozygote
16100465	1.20	1	10	1.88	1	10: single-copy homozygote
16490130	1.13	1	7	2.03	1	7: single-copy homozygote
16490131	1.04	1	12	1.95	1	12: single-copy homozygote
16190723	1.13	1	23	1.97	1	23: single-copy homozygote
16230278	1.18	1	11	2.00	1	11: single-copy homozygote
15462100	1.18	1	5	2.03	1	5: single-copy homozygote
15462128	1.10	1	16	2.02	1	16: single-copy homozygote
15492249	1.40	1	9	2.04	1	9: single-copy homozygote
15310934	1.18	1	3	2.00	1	3: single-copy homozygote
15310961	1.03	1	1	1.91	1	1: single-copy homozygote
17160165	0.97	1	4	2.09	1	4: single-copy homozygote
16131540	0.88	1	6	1.96	1	6: single-copy homozygote
16131197	1.32	1	9	1.98	1	9: single-copy homozygote

Transgenic Line	T_0		T_1			T_2
	Ratio (target/ref)	Estimated Copy Number	Homozygote No.	Ratio (target/ref)	Estimated Copy Number of T_0 Plant	Verification of T_1 Homozygote
15290277	1.22	1	17	1.74	1	17: single-copy homozygote
15260517	0.83	1	13	2.02	1	13: single-copy homozygote
15260218	0.85	1	12	1.95	1	12: single-copy homozygote
15260262	1.36	1	1	1.95	1	1: single-copy homozygote
16130973	1.24	1	12	2.01	1	12: single-copy homozygote
17100203	1.32	1	1	2.10	1	1: single-copy homozygote
17100208	1.25	1	1	2.17	1	1: single-copy homozygote
17110122	1.22	1	4	2.15	1	4: single-copy homozygote
17110123	1.04	1	2	2.06	1	2: single-copy homozygote
16040450	1.14	1	5	2.00	1	5: single-copy homozygote
16060147	1.11	1	13	2.02	1	13: single-copy homozygote
16060158	1.25	1	4	2.00	1	4: single-copy homozygote
16060159	1.13	1	14	1.96	1	14: single-copy homozygote
15431641	1.18	1	7	2.01	1	7: single-copy homozygote
15451511	1.02	1	17	2.04	1	17: single-copy homozygote
15410087	1.26	1	14	1.84	1	14: single-copy homozygote
15410103	1.22	1	1	2.03	1	1: single-copy homozygote
15390021	1.20	1	11	2.03	1	11: single-copy homozygote
15390239	1.32	1	5	1.89	1	5: single-copy homozygote
15330847	1.14	1	22	1.97	1	22: single-copy homozygote

Transgenic Line	T_0		T_1			T_2
	Ratio (target/ref)	Estimated Copy Number	Homozygote No.	Ratio (target/ref)	Estimated Copy Number of T_0 Plant	Verification of T_1 Homozygote
16200681	0.75	1	5	2.41	1	5: single-copy homozygote
16200711	1.22	1	10	2.18	1	10: single-copy homozygote
15311320	1.13	1	2	2.13	1	2: single-copy homozygote
15440973	0.87	1	7	1.84	1	7: single-copy homozygote
16131826	1.33	1	13	1.82	1	13: single-copy homozygote
16120354	1.27	1	2	1.97	1	2: single-copy homozygote
16131944	1.36	1	9	1.73	1	9: single-copy homozygote
16190321	0.75	1	20	1.93	1	20: single-copy homozygote
15490333	1.11	1	3	2.04	1	3: single-copy homozygote
15490437	1.18	1	3	1.95	1	3: single-copy homozygote
16240377	1.34	1	9	2.05	1	9: single-copy homozygote
16260145	0.77	1	5	2.11	1	5: single-copy homozygote
15461996	1.24	1	6	2.00	1	6: single-copy homozygote
15462266	1.08	1	14	1.99	1	14: single-copy homozygote
17050192	1.24	1	1	2.27	1	1: single-copy homozygote
15420930	0.83	1	5	2.24	1	5: single-copy homozygote
15360388	1.05	1	3	2.13	1	3: single-copy homozygote
15430493	1.26	1	1	2.24	1	1: single-copy homozygote
15500944	1.07	1	10	2.00	1	10: single-copy homozygote
15500945	1.01	1	6	1.98	1	6: single-copy homozygote

Transgenic Line	T_0		T_1			T_2
	Ratio (target/ref)	Estimated Copy Number	Homozygote No.	Ratio (target/ref)	Estimated Copy Number of T_0 Plant	Verification of T_1 Homozygote
15520016	0.89	1	4	2.06	1	4: single-copy homozygote
16210515	1.16	1	7	2.12	1	7: single-copy homozygote
16250487	0.78	1	13	2.09	1	13: single-copy homozygote
16280128	1.04	1	5	1.87	1	5: single-copy homozygote
16260564	0.65	1	2	1.98	1	2: single-copy homozygote
16020266	1.23	1	2	2.04	1	2: single-copy homozygote
15400561	0.77	1	22	2.00	1	22: single-copy homozygote
17190152	1.07	1	1	1.99	1	1: single-copy homozygote
16010074	0.90	1	2	1.92	1	2: single-copy homozygote
16130749	1.16	1	17	1.94	1	17: single-copy homozygote
16130757	1.06	1	8	2.00	1	8: single-copy homozygote
16190844	1.15	1	17	2.00	1	17: single-copy homozygote
17100193	1.16	1	2	1.86	1	2: single-copy homozygote
17140268	1.06	1	8	1.96	1	8: single-copy homozygote
17140579	1.01	1	1	1.97	1	1: single-copy homozygote
17150674	1.34	1	1	4.34	2	1: two-copy homozygote
17090180	1.05	1	4	1.98	1	4: single-copy homozygote
16131781	1.18	1	6	2.11	1	6: single-copy homozygote
16131542	1.26	1	8	2.00	1	8: single-copy homozygote
16131812	1.37	1	8	3.91	2	8: two-copy homozygote

Transgenic Line	T_0		T_1			T_2
	Ratio (target/ref)	Estimated Copy Number	Homozygote No.	Ratio (target/ref)	Estimated Copy Number of T_0 Plant	Verification of T_1 Homozygote
16130584	1.21	1	8	2.02	1	8: single-copy homozygote
16230351	1.2	1	4	1.87	1	4: single-copy homozygote
16230352	0.91	1	5	1.78	1	5: single-copy homozygote
16230375	1.35	1	5	2.00	1	5: single-copy homozygote
17170041	1.09	1	1	1.91	1	1: single-copy homozygote
16161137	1.08	1	7	2.00	1	7: single-copy homozygote
16230140	1.21	1	2	2.02	1	2: single-copy homozygote
16160948	1.06	1	5	1.90	1	5: single-copy homozygote
16160949	1.02	1	10	2.05	1	10: single-copy homozygote
16161023	1.24	1	6	2.00	1	6: single-copy homozygote
16161038	1.06	1	6	1.84	1	6: single-copy homozygote
16230074	1.26	1	13	2.00	1	13: single-copy homozygote
16230081	1.2	1	1	1.93	1	1: single-copy homozygote
16210287	1.26	1	19	3.80	2	19: two-copy homozygote
16200830	1.19	1	6	1.98	1	6: single-copy homozygote
16200833	0.92	1	5	1.99	1	5: single-copy homozygote
16200025	1.11	1	1	2.29	1	1: single-copy homozygote
16200137	1.26	1	1	1.86	1	1: single-copy homozygote
16200179	1.22	1	8	1.99	1	8: single-copy homozygote
16200338	1.13	1	11	1.93	1	11: single-copy homozygote

Transgenic Line	T_0		T_1			T_2
	Ratio (target/ref)	Estimated Copy Number	Homozygote No.	Ratio (target/ref)	Estimated Copy Number of T_0 Plant	Verification of T_1 Homozygote
16200348	1.17	1	7	1.94	1	7: single-copy homozygote
15100378	1.10	1	16	1.93	1	16: single-copy homozygote
15370256	1.04	1	4	1.88	1	4: single-copy homozygote
16111246	1.31	1	2	2.02	1	2: single-copy homozygote
16120982	1.15	1	4	1.95	1	4: single-copy homozygote
15180153	0.76	1	1	1.99	1	1: single-copy homozygote
15400709	1.21	1	3	1.85	1	3: single-copy homozygote
16220285	1.19	1	3	1.90	1	3: single-copy homozygote
16230325	0.99	1	4	1.96	1	4: single-copy homozygote
17150141	1.3	1	2	2.03	1	2: single-copy homozygote
17180656	1.22	1	1	2.01	1	1: single-copy homozygote
16250281	1.39	1	3	2.16	1	3: single-copy homozygote
17050112	1.05	1	6	1.93	1	6: single-copy homozygote
17070459	1.15	1	6	2.23	1	6: single-copy homozygote
17090207	1.24	1	4	1.92	1	4: single-copy homozygote
17090276	1.18	1	1	2.26	1	1: single-copy homozygote
16280076	1.38	1	1	2.07	1	1: single-copy homozygote
17070193	1.12	1	2	2.16	1	2: single-copy homozygote
17140460	1.38	1	8	2.03	1	8: single-copy homozygote
16200960	1.05	1	10	2.00	1	10: single-copy homozygote

Transgenic Line	T_0		T_1			T_2
	**Ratio (target/ref)**	**Estimated Copy Number**	**Homozygote No.**	**Ratio (target/ref)**	**Estimated Copy Number of T_0 Plant**	**Verification of T_1 Homozygote**
16200980	1.11	1	2	1.99	1	2: single-copy homozygote
16260169	1.26	1	7	2.15	1	7: single-copy homozygote
16210595	1.27	1	12	2.19	1	12: single-copy homozygote
16220386	1.15	1	18	2.03	1	18: single-copy homozygote
16240428	1.07	1	4	1.93	1	4: single-copy homozygote
16250421	0.91	1	5	2.02	1	5: single-copy homozygote
16230229	0.96	1	11	2.00	1	11: single-copy homozygote
16270288	1.09	1	14	1.94	1	14: single-copy homozygote
17180279	1.12	1	9	2.02	1	9: single-copy homozygote
16080216	1.28	1	2	2.04	1	2: single-copy homozygote
16110635	1.28	1	11	2.20	1	11: single-copy homozygote
16110658	1.05	1	1	2.05	1	1: single-copy homozygote
16131015	1.39	1	16	2.14	1	16: single-copy homozygote
16120203	0.85	1	1	1.92	1	1: single-copy homozygote
15111365	1.28	1	9	1.96	1	9: single-copy homozygote
16200799	0.67	1	7	1.88	1	7: single-copy homozygote
16200800	1.05	1	7	1.74	1	7: single-copy homozygote
15030070	1.00	1	15	3.72	2	15: two-copy homozygote
15311264	1.01	1	12	1.93	1	12: single-copy homozygote
15230050	1.18	1	14	2.11	1	14: single-copy homozygote

Transgenic Line	T_0		T_1			T_2
	Ratio (target/ref)	Estimated Copy Number	Homozygote No.	Ratio (target/ref)	Estimated Copy Number of T_0 Plant	Verification of T_1 Homozygote
15370346	1.37	1	14	2.06	1	14: single-copy homozygote
15340411	1.20	1	4	2.04	1	4: single-copy homozygote
15310320	1.28	1	9	2.34	1	9: single-copy homozygote
15360422	1.15	1	14	1.77	1	14: single-copy homozygote
16121179	1.34	1	2	2.06	1	2: single-copy homozygote
16121200	1.23	1	2	2.04	1	2: single-copy homozygote
16160491	1.19	1	1	1.75	1	1: single-copy homozygote
16490163	0.94	1	3	2.13	1	3: single-copy homozygote
16490208	1.11	1	18	2.05	1	18: single-copy homozygote
16110037	1.33	1	15	1.93	1	15: single-copy homozygote
16160944	1.25	1	10	1.83	1	10: single-copy homozygote
16490145	1.01	1	1	1.93	1	1: single-copy homozygote
15490819	0.71	1	3	3.87	2	3: two-copy homozygote
16010742	0.48	0	2	1.89	1	2: single-copy homozygote
16020015	1.39	1	16	2.19	1	16: single-copy homozygote
16190387	1.31	1	14	2.04	1	14: single-copy homozygote
16470211	0.87	1	16	1.91	1	16: single-copy homozygote
16490167	0.91	1	18	1.91	1	18: single-copy homozygote
17010448	1.27	1	3	2.04	1	3: single-copy homozygote
16110433	1.33	1	17	1.95	1	17: single-copy homozygote

Transgenic Line	T_0		T_1			T_2
	Ratio (target/ref)	Estimated Copy Number	Homozygote No.	Ratio (target/ref)	Estimated Copy Number of T_0 Plant	Verification of T_1 Homozygote
16121340	1.14	1	5	2.04	1	5: single-copy homozygote
16500122	1.31	1	5	2.02	1	5: single-copy homozygote
17070470	1.01	1	1	2.60	1	1: single-copy homozygote
16500438	1.21	1	13	2.01	1	13: single-copy homozygote
17050163	1.01	1	12	1.99	1	12: single-copy homozygote
17150497	1.15	1	11	1.98	1	11: single-copy homozygote
17160211	1.16	1	1	1.92	1	1: single-copy homozygote
17010328	1.37	1	14	2.02	1	14: single-copy homozygote
17010418	1.37	1	1	1.92	1	1: single-copy homozygote
17030189	1.09	1	1	2.19	1	1: single-copy homozygote
16500131	0.67	1	11	2.06	1	11: single-copy homozygote
16500504	1.16	1	17	2.01	1	17: single-copy homozygote
16500505	1.29	1	11	2.08	1	11: single-copy homozygote
17020427	1.12	1	4	1.98	1	4: single-copy homozygote
17010089	1.26	1	19	1.89	1	19: single-copy homozygote
17010090	1.31	1	11	1.96	1	11: single-copy homozygote
17010492	0.89	1	7	2.04	1	7: single-copy homozygote
17070198	1.07	1	12	2.06	1	12: two-copy homozygote
17070199	1.28	1	6	2.02	1	6: single-copy homozygote
16120722	1.22	1	11	1.90	1	11: single-copy homozygote

Transgenic Line	T_0		T_1			T_2
	Ratio (target/ref)	Estimated Copy Number	Homozygote No.	Ratio (target/ref)	Estimated Copy Number of T_0 Plant	Verification of T_1 Homozygote
16120721	1.21	1	5	1.96	1	5: single-copy homozygote
16190134	1.34	1	9	1.95	1	9: single-copy homozygote
16220324	1.26	1	1	2.19	1	1: single-copy homozygote
16270084	1.01	1	11	1.97	1	11: single-copy homozygote
16140576	1.05	1	11	2.08	1	11: single-copy homozygote
16090621	1.28	1	6	2.32	1	6: single-copy homozygote
16120732	1.10	1	8	2.05	1	8: single-copy homozygote
17100020	0.96	1	7	2.03	1	7: single-copy homozygote
17100298	1.15	1	2	2.20	1	2: single-copy homozygote
17140548	1.13	1	9	1.88	1	9: single-copy homozygote
17180838	1.25	1	18	2.03	1	18: single-copy homozygote
17140735	1.1	1	1	2.06	1	1: single-copy homozygote
16170219	1.09	1	7	2.28	1	7: single-copy homozygote
16170223	1.29	1	2	1.88	1	2: single-copy homozygote
16220093	1.36	1	7	2.03	1	7: single-copy homozygote
16220226	1.32	1	1	1.93	1	1: single-copy homozygote
16220203	1.18	1	6	2.01	1	6: single-copy homozygote
16190269	0.88	1	1	1.89	1	1: single-copy homozygote
15500833	1.23	1	5	2.13	1	5: single-copy homozygote
15500834	1.06	1	18	2.11	1	18: single-copy homozygote

Transgenic Line	T_0		T_1			T_2
	Ratio (target/ref)	Estimated Copy Number	Homozygote No.	Ratio (target/ref)	Estimated Copy Number of T_0 Plant	Verification of T_1 Homozygote
16210455	1.36	1	2	2.24	1	2: single-copy homozygote
15491637	1.28	1	15	2.01	1	15: single-copy homozygote
15510469	1.39	1	17	1.92	1	17: single-copy homozygote
15481704	1.29	1	3	2.01	1	3: single-copy homozygote
16020213	1.19	1	1	1.78	1	1: single-copy homozygote
16020249	1.33	1	7	2.55	1	7: single-copy homozygote
16020098	1.33	1	5	1.92	1	5: single-copy homozygote
15470232	1.16	1	5	1.90	1	5: single-copy homozygote
15481129	1.07	1	20	1.87	1	20: single-copy homozygote
15480571	1.15	1	10	1.99	1	10: single-copy homozygote
15491486	1.31	1	3	2.09	1	3: single-copy homozygote
16020230	1.20	1	11	2.07	1	11: single-copy homozygote
17150094	0.95	1	1	2.32	1	1: single-copy homozygote
17030194	1.29	1	1	1.92	1	1: single-copy homozygote
16500240	1.35	1	15	1.99	1	15: single-copy homozygote
16500485	1.16	1	14	1.95	1	14: single-copy homozygote
16490095	1.15	1	23	1.96	1	23: single-copy homozygote
17030214	1.27	1	2	2.03	1	2: single-copy homozygote
17030238	1.36	1	4	2.16	1	4: single-copy homozygote
17080227	1.01	1	2	2.00	1	2: single-copy homozygote

Transgenic Line	T_0		T_1			T_2
	Ratio (target/ref)	Estimated Copy Number	Homozygote No.	Ratio (target/ref)	Estimated Copy Number of T_0 Plant	Verification of T_1 Homozygote
16490024	1.2	1	4	1.97	1	4: single-copy homozygote
17020141	1.15	1	3	2.13	1	3: single-copy homozygote
16500109	0.95	1	1	2.06	1	1: single-copy homozygote
16500251	0.88	1	7	1.99	1	7: single-copy homozygote
17060126	1.02	1	3	1.83	1	3: single-copy homozygote
16460225	0.89	1	16	2.01	1	16: single-copy homozygote
17050386	1.12	1	6	1.97	1	6: two-copy homozygote
17080147	1.36	1	1	1.81	1	1: single-copy homozygote
17010225	1.38	1	10	1.97	1	10: single-copy homozygote
17020190	1.04	1	4	2.20	1	4: single-copy homozygote
17020028	0.67	1	1	2.11	1	1: single-copy homozygote
17010011	0.95	1	1	1.92	1	1: single-copy homozygote
17030127	1	1	10	2.07	1	10: single-copy homozygote
17200288	1.17	1	7	2.08	1	7: single-copy homozygote
16490008	1.11	1	17	1.98	1	17: single-copy homozygote
17020377	0.93	1	3	2.08	1	3: single-copy homozygote
17030306	1.21	1	9	2.36	1	9: single-copy homozygote
16480096	0.73	1	9	2.04	1	9: single-copy homozygote
16480097	0.88	1	2	2.24	1	2: single-copy homozygote
16480106	0.8	1	12	2.19	1	12: single-copy homozygote

Transgenic Line	T_0		T_1			T_2
	Ratio (target/ref)	Estimated Copy Number	Homozygote No.	Ratio (target/ref)	Estimated Copy Number of T_0 Plant	Verification of T_1 Homozygote
16480136	0.92	1	17	1.94	1	17: single-copy homozygote
16490119	1.03	1	16	2.33	1	16: single-copy homozygote
17010441	1.29	1	18	2.05	1	18: single-copy homozygote
17010442	1.28	1	12	2.23	1	12: single-copy homozygote
16480076	0.67	1	3	1.97	1	3: single-copy homozygote
16490185	0.87	1	8	2.21	1	8: single-copy homozygote
17080331	1.1	1	3	2.31	1	3: single-copy homozygote
17090073	1.39	1	1	2.56	1	1: single-copy homozygote
17090058	1.1	1	4	2.03	1	4: single-copy homozygote
17140912	1.14	1	1	2.30	1	1: single-copy homozygote
17050487	1.14	1	1	2.48	1	1: single-copy homozygote
17100030	1.37	1	1	2.51	1	1: single-copy homozygote
17100200	1.15	1	2	1.90	1	2: single-copy homozygote
17150033	1.3	1	11	2.00	1	11: single-copy homozygote
17150034	1.3	1	12	2.08	1	12: single-copy homozygote
17160463	0.62	1	8	2.00	1	8: single-copy homozygote
17080010	1.01	1	4	2.03	1	4: single-copy homozygote
17030152	1.01	1	1	2.09	1	1: single-copy homozygote
17030154	1.25	1	3	2.35	1	3: single-copy homozygote
17040016	1.26	1	11	1.98	1	11: single-copy homozygote

Transgenic Line	T_0		T_1			T_2
	**Ratio (target/ref)**	**Estimated Copy Number**	**Homozygote No.**	**Ratio (target/ref)**	**Estimated Copy Number of T_0 Plant**	**Verification of T_1 Homozygote**
17050501	1.03	1	5	2.01	1	5: single-copy homozygote
17090148	1.38	1	1	2.06	1	1: single-copy homozygote
17070282	0.67	1	1	2.05	1	1: single-copy homozygote
17120288	0.99	1	1	2.15	1	1: single-copy homozygote
17140048	1.23	1	16	1.96	1	16: single-copy homozygote
17160143	0.98	1	5	2.02	1	5: single-copy homozygote
17160145	0.97	1	10	2.00	1	10: single-copy homozygote
17110048	0.99	1	5	1.98	1	5: single-copy homozygote
17180859	1.18	1	1	2.01	1	1: single-copy homozygote
17150185	0.88	1	3	1.96	1	3: single-copy homozygote
17150280	1.08	1	7	1.80	1	7: single-copy homozygote
17151138	0.86	1	3	2.08	1	3: single-copy homozygote
17180501	1.26	1	9	1.93	1	9: single-copy homozygote
17150076	1.26	1	2	2.12	1	2: single-copy homozygote
17180217	1.01	1	7	2.15	1	7: single-copy homozygote
17100412	1.06	1	7	1.82	1	7: single-copy homozygote
17080254	1.29	1	3	2.07	1	3: single-copy homozygote
17140788	1.03	1	2	2.25	1	2: single-copy homozygote
17140847	1.13	1	4	1.94	1	4: single-copy homozygote
17140943	1.22	1	7	2.10	1	7: single-copy homozygote

Transgenic Line	T_0		T_1			T_2
	Ratio (target/ref)	Estimated Copy Number	Homozygote No.	Ratio (target/ref)	Estimated Copy Number of T_0 Plant	Verification of T_1 Homozygote
17150377	1.24	1	7	2.02	1	7: single-copy homozygote
17150506	1.06	1	4	1.98	1	4: single-copy homozygote
17150256	0.91	1	5	2.23	1	5: single-copy homozygote
17170024	1.37	1	13	4.40	2	13: two-copy homozygote
17240091	1.12	1	15	2.19	1	15: single-copy homozygote
17150340	1.22	1	9	2.01	1	9: single-copy homozygote
17160082	0.58	1	5	1.94	1	5: single-copy homozygote
17180016	1.17	1	12	2.13	1	12: single-copy homozygote
16230335	1.11	1	8	2.04	1	8: single-copy homozygote
17290342	0.95	1	2	2.17	1	2: single-copy homozygote
17310967	1.21	1	3	1.88	1	3: single-copy homozygote
17310976	1.38	1	10	2.16	1	10: single-copy homozygote
17110120	1.14	1	14	2.01	1	14: single-copy homozygote
17260832	1.18	1	11	2.02	1	11: single-copy homozygote
17290703	1.09	1	4	2.03	1	4: single-copy homozygote
17300237	1.17	1	6	2.24	1	6: single-copy homozygote
17420725	0.97	1	5	1.88	1	5: single-copy homozygote
17290678	1.05	1	1	2.09	1	1: single-copy homozygote
17290891	0.95	1	3	1.96	1	3: single-copy homozygote
17381034	1.34	1	5	1.95	1	5: single-copy homozygote

Transgenic Line	T_0		T_1			T_2
	Ratio (target/ref)	Estimated Copy Number	Homozygote No.	Ratio (target/ref)	Estimated Copy Number of T_0 Plant	Verification of T_1 Homozygote
17320076	1.02	1	7	2.01	1	7: single-copy homozygote
17320077	1.00	1	1	2.10	1	1: single-copy homozygote
17340728	1.07	1	1	2.25	1	1: single-copy homozygote
17200027	1.36	1	8	1.96	1	8: single-copy homozygote
17310457	1.23	1	7	2.08	1	7: single-copy homozygote
17320097	1.18	1	4	1.99	1	4: single-copy homozygote
17320108	1.09	1	1	1.95	1	1: single-copy homozygote
17370705	0.88	1	2	1.96	1	2: single-copy homozygote
17310140	1.30	1	2	1.99	1	2: single-copy homozygote
17320378	1.27	1	11	1.98	1	11: single-copy homozygote
17360210	1.30	1	19	2.02	1	19: single-copy homozygote
17381037	1.20	1	2	1.99	1	2: single-copy homozygote
17420577	1.18	1	1	2.07	1	1: single-copy homozygote
17420711	0.99	1	2	2.00	1	2: single-copy homozygote
17120020	0.91	1	1	1.80	1	1: single-copy homozygote
17330476	0.95	1	13	1.98	1	13: single-copy homozygote
17330478	0.90	1	13	1.97	1	13: single-copy homozygote
17341572	1.18	1	16	1.98	1	16: single-copy homozygote
17391699	1.32	1	12	1.95	1	12: single-copy homozygote
17260657	1.24	1	9	1.98	1	9: single-copy homozygote

Transgenic Line	T_0		T_1			T_2
	Ratio (target/ref)	Estimated Copy Number	Homozygote No.	Ratio (target/ref)	Estimated Copy Number of T_0 Plant	Verification of T_1 Homozygote
17260668	1.07	1	10	2.13	1	10: single-copy homozygote
17320918	1.03	1	10	1.85	1	10: single-copy homozygote
17360337	1.16	1	1	1.99	1	1: single-copy homozygote
17080349	0.87	1	1	2.08	1	1: single-copy homozygote
17190211	1.27	1	10	2.05	1	10: single-copy homozygote
17370323	0.86	1	10	2.13	1	10: single-copy homozygote
17400365	1.32	1	3	1.86	1	3: single-copy homozygote
17340577	1.05	1	21	2.10	1	21: single-copy homozygote
17340815	1.06	1	20	2.01	1	20: single-copy homozygote
17360287	1.13	1	8	1.97	1	8: single-copy homozygote
17360321	1.11	1	3	1.83	1	3: single-copy homozygote
17350046	1.25	1	8	2.20	1	8: single-copy homozygote
17370371	0.69	1	17	4.12	2	17: two-copy homozygote
17370792	1.29	1	13	1.99	1	13: single-copy homozygote
17370813	1.10	1	7	2.05	1	7: single-copy homozygote
17350570	1.24	1	19	2.00	1	19: single-copy homozygote
17360133	1.32	1	22	2.13	1	22: single-copy homozygote
17370694	1.17	1	5	1.84	1	5: single-copy homozygote
17370706	1.05	1	1	2.05	1	1: single-copy homozygote
17380720	0.93	1	13	2.01	1	13: single-copy homozygote

Transgenic Line	T_0		T_1			T_2
	**Ratio (target/ref)**	**Estimated Copy Number**	**Homozygote No.**	**Ratio (target/ref)**	**Estimated Copy Number of T_0 Plant**	**Verification of T_1 Homozygote**
17380924	1.07	1	6	2.00	1	6: single-copy homozygote
17390522	1.08	1	12	1.99	1	12: single-copy homozygote
16470310	0.91	1	21	1.98	1	21: single-copy homozygote
17360304	1.12	1	11	1.94	1	11: single-copy homozygote
17400608	1.23	1	1	1.81	1	1: single-copy homozygote
17410880	1.26	1	4	2.07	1	4: single-copy homozygote
16460136	1.37	1	19	1.98	1	19: single-copy homozygote
17321017	1.06	1	10	2.05	1	10: single-copy homozygote
17360448	1.25	1	9	1.85	1	9: single-copy homozygote
17370869	1.25	1	10	2.23	1	10: single-copy homozygote
17250894	1.07	1	7	2.37	1	7: single-copy homozygote
17220744	1.29	1	20	2.01	1	20: single-copy homozygote
17190293	1.32	1	10	1.97	1	10: single-copy homozygote
17290041	0.92	1	7	2.48	1	7: single-copy homozygote
17290056	1.35	1	9	2.26	1	9: single-copy homozygote
17320575	0.97	1	7	2.07	1	7: single-copy homozygote
17050471	0.95	1	13	1.82	1	13: single-copy homozygote
17020304	0.59	1	7	1.99	1	7: single-copy homozygote
17421143	0.94	1	8	2.10	1	8: single-copy homozygote
17430859	1.20	1	7	2.01	1	7: single-copy homozygote

Transgenic Line	T_0		T_1			T_2
	Ratio (target/ref)	Estimated Copy Number	Homozygote No.	Ratio (target/ref)	Estimated Copy Number of T_0 Plant	Verification of T_1 Homozygote
17140918	1.30	1	4	2.08	1	4: single-copy homozygote
17160327	1.03	1	7	1.85	1	7: single-copy homozygote
17300349	0.81	1	13	1.74	1	13: single-copy homozygote
17350688	1.39	1	8	1.79	1	8: single-copy homozygote
17380841	1.02	1	6	1.87	1	6: single-copy homozygote
17420753	1.23	1	5	1.94	1	5: single-copy homozygote
17110257	1.07	1	6	1.88	1	6: single-copy homozygote
17160467	1.07	1	2	1.97	1	2: single-copy homozygote
17420480	1.22	1	1	1.99	1	1: single-copy homozygote
17360109	1.17	1	14	1.97	1	14: single-copy homozygote
17400381	1.11	1	10	1.81	1	10: single-copy homozygote
17401359	1.24	1	6	2.14	1	6: single-copy homozygote
17150357	1.11	1	2	1.88	1	2: single-copy homozygote
17250580	1.11	1	8	1.94	1	8: single-copy homozygote
17310030	1.13	1	12	1.80	1	12: single-copy homozygote
17350627	1.33	1	9	1.91	1	9: single-copy homozygote
17390165	1.26	1	3	2.31	1	3: single-copy homozygote
17331206	1.20	1	6	1.93	1	6: single-copy homozygote
17360295	1.09	1	4	1.91	1	4: single-copy homozygote
17400461	1.19	1	12	2.07	1	12: single-copy homozygote

Transgenic Line	T_0		T_1			T_2
	Ratio (target/ref)	Estimated Copy Number	Homozygote No.	Ratio (target/ref)	Estimated Copy Number of T_0 Plant	Verification of T_1 Homozygote
17240038	1.20	1	1	2.08	1	1: single-copy homozygote
17240615	0.67	1	5	2.01	1	5: single-copy homozygote
17371088	1.18	1	16	1.92	1	16: single-copy homozygote
17371197	1.07	1	2	1.99	1	2: single-copy homozygote
17400217	1.38	1	12	1.97	1	12: single-copy homozygote
17330577	1.33	1	16	2.33	1	16: single-copy homozygote
17391491	1.26	1	5	1.84	1	5: single-copy homozygote
17401101	1.30	1	5	2.06	1	5: single-copy homozygote
17370800	0.88	1	3	1.85	1	3: single-copy homozygote
17380421	1.33	1	7	1.90	1	7: single-copy homozygote
17390512	1.10	1	5	1.98	1	5: single-copy homozygote
17321119	0.98	1	2	3.73	2	2: single-copy homozygote
17370729	1.08	1	19	2.04	1	19: single-copy homozygote
17360924	1.08	1	14	1.93	1	14: single-copy homozygote
17290291	1.01	1	7	1.97	1	7: single-copy homozygote
17350476	0.66	1	3	2.02	1	3: single-copy homozygote
17321187	1.29	1	6	1.94	1	6: single-copy homozygote
17331467	1.15	1	7	2.06	1	7: single-copy homozygote
17350556	1.11	1	2	2.03	1	2: single-copy homozygote
17380528	1.35	1	1	2.10	1	1: single-copy homozygote

Transgenic Line	T_0		T_1			T_2
	Ratio (target/ref)	Estimated Copy Number	Homozygote No.	Ratio (target/ref)	Estimated Copy Number of T_0 Plant	Verification of T_1 Homozygote
17380547	1.02	1	4	1.85	1	4: single-copy homozygote
17300737	0.93	1	8	1.89	1	8: single-copy homozygote
17330212	1.10	1	10	1.99	1	10: single-copy homozygote
17341673	1.18	1	3	2.37	1	3: single-copy homozygote
17391402	1.38	1	12	2.14	1	12: single-copy homozygote
17410211	1.13	1	6	1.91	1	6: single-copy homozygote
17410267	1.08	1	7	1.96	1	7: single-copy homozygote
17391675	1.26	1	23	2.30	1	23: single-copy homozygote
17410225	1.16	1	10	1.95	1	10: single-copy homozygote
17340521	1.10	1	18	1.89	1	18: single-copy homozygote
17390421	1.38	1	15	2.18	1	15: single-copy homozygote
17390688	1.35	1	5	1.86	1	5: single-copy homozygote
17380057	1.31	1	10	1.97	1	10: single-copy homozygote
17400755	1.12	1	12	1.97	1	12: single-copy homozygote
17400796	1.01	1	11	2.02	1	11: single-copy homozygote
17400852	1.24	1	1	2.17	1	1: single-copy homozygote
17400860	1.25	1	22	1.89	1	22: single-copy homozygote
17380073	1.37	1	12	2.03	1	12: single-copy homozygote
17380074	1.19	1	1	2.35	1	1: single-copy homozygote
17400821	1.21	1	13	1.88	1	13: single-copy homozygote

Transgenic Line	T_0		T_1			T_2
	Ratio (target/ref)	Estimated Copy Number	Homozygote No.	Ratio (target/ref)	Estimated Copy Number of T_0 Plant	Verification of T_1 Homozygote
17380034	1.20	1	20	2.01	1	20: single-copy homozygote
17380078	1.04	1	3	1.95	1	3: single-copy homozygote
17380080	0.95	1	9	2.27	1	9: single-copy homozygote
17380152	1.23	1	5	2.25	1	5: single-copy homozygote
17380026	1.11	1	21	2.48	1	21: single-copy homozygote
17400869	1.23	1	16	1.93	1	16: single-copy homozygote
17400699	1.11	1	19	2.00	1	19: single-copy homozygote
17400920	1.17	1	7	2.01	1	7: single-copy homozygote
17391220	1.31	1	5	1.99	1	5: single-copy homozygote
17400787	1.29	1	15	1.97	1	15: single-copy homozygote
17430880	1.22	1	13	1.93	1	13: single-copy homozygote
15150366	0.98	1	16	2.46	1	16: single-copy homozygote
16170210	1.29	1	4	2.07	1	4: single-copy homozygote
17180051	1.17	1	3	2.05	1	3: single-copy homozygote
16030331	1.20	1	7	1.90	1	7: single-copy homozygote
16060060	1.19	1	16	1.92	1	16: single-copy homozygote
17371153	1.21	1	14	2.27	1	14: single-copy homozygote
17371154	1.16	1	13	2.47	1	13: single-copy homozygote
15470442	1.09	1	12	2.05	1	12: single-copy homozygote
16490294	0.51	1	3	1.93	1	3: single-copy homozygote

**Transgenic Line**	**T_0**		**T_1**			**T_2**
	**Ratio (target/ref)**	**Estimated Copy Number**	**Homozygote No.**	**Ratio (target/ref)**	**Estimated Copy Number of T_0 Plant**	**Verification of T_1 Homozygote**
17100229	1.14	1	11	2.09	1	11: single-copy homozygote
17320337	1.04	1	2	1.91	1	2: single-copy homozygote
16190071	1.38	1	4	1.93	1	4: single-copy homozygote
17150183	1.26	1	3	2.21	1	3: single-copy homozygote
17331028	1.13	1	10	2.15	1	10: single-copy homozygote
17391209	1.23	1	9	2.21	1	9: single-copy homozygote
17410806	1.09	1	14	2.30	1	14: single-copy homozygote
17430836	1.22	1	14	2.21	1	14: single-copy homozygote
14340886	1.02	1	15	1.99	1	15: single-copy homozygote
14280062	1.23	1	3	1.90	1	3: single-copy homozygote
15520678	1.37	1	12	1.87	1	12: single-copy homozygote
15430634	1.14	1	20	1.81	1	20: single-copy homozygote
17120047	0.98	1	4	2.00	1	4: single-copy homozygote
17470050	1.13	1	17	2.12	1	17: single-copy homozygote
17481008	1.35	1	4	2.49	1	4: single-copy homozygote
17490773	1.18	1	16	1.76	1	16: single-copy homozygote
17430786	1.11	1	2	1.98	1	2: single-copy homozygote
17440969	1.00	1	23	2.00	1	23: single-copy homozygote
17450217	1.12	1	4	2.01	1	4: single-copy homozygote
17450490	1.33	1	23	1.99	1	23: single-copy homozygote

Transgenic Line	T_0		T_1			T_2
	Ratio (target/ref)	Estimated Copy Number	Homozygote No.	Ratio (target/ref)	Estimated Copy Number of T_0 Plant	Verification of T_1 Homozygote
17450546	1.07	1	3	1.93	1	3: single-copy homozygote
17450584	1.28	1	8	2.05	1	8: single-copy homozygote
17451189	1.23	1	13	2.24	1	13: single-copy homozygote
17451346	1.12	1	2	2.26	1	2: single-copy homozygote
17451923	1.05	1	15	2.24	1	15: single-copy homozygote
17460009	0.87	1	10	2.00	1	10: single-copy homozygote
17460040	1.06	1	10	1.95	1	10: single-copy homozygote
17460120	1.01	1	13	2.05	1	13: single-copy homozygote
17460123	1.17	1	1	2.15	1	1: single-copy homozygote
17460134	1.31	1	13	2.17	1	13: single-copy homozygote
17460234	1.08	1	1	1.99	1	1: single-copy homozygote
17460256	0.70	1	15	2.02	1	15: single-copy homozygote
17460278	1.20	1	1	1.86	1	1: single-copy homozygote
17461059	1.16	1	1	2.09	1	1: single-copy homozygote
17461111	1.05	1	9	1.95	1	9: single-copy homozygote
17470240	1.31	1	7	2.03	1	7: single-copy homozygote
17470347	1.29	1	5	2.16	1	5: single-copy homozygote
17470405	1.18	1	3	2.06	1	3: single-copy homozygote
17470476	1.21	1	7	1.99	1	7: single-copy homozygote
17470511	1.18	1	16	1.91	1	16: single-copy homozygote

Transgenic Line	T_0		T_1			T_2
	**Ratio (target/ref)**	**Estimated Copy Number**	**Homozygote No.**	**Ratio (target/ref)**	**Estimated Copy Number of T_0 Plant**	**Verification of T_1 Homozygote**
17470721	1.18	1	8	1.95	1	8: single-copy homozygote
17471045	1.19	1	5	1.90	1	5: single-copy homozygote
17471110	1.01	1	10	1.74	1	10: single-copy homozygote
17471240	1.17	1	10	1.96	1	10: single-copy homozygote
17480307	1.27	1	11	2.00	1	11: single-copy homozygote
17480480	0.97	1	18	1.94	1	18: single-copy homozygote
17480527	0.99	1	3	2.11	1	3: single-copy homozygote
17480670	0.52	1	2	1.97	1	2: single-copy homozygote
17480672	1.23	1	12	1.99	1	12: single-copy homozygote
17480748	1.08	1	9	1.83	1	9: single-copy homozygote
17480765	1.15	1	14	2.05	1	14: single-copy homozygote
17480965	1.12	1	4	1.89	1	4: single-copy homozygote
17490081	1.24	1	24	1.97	1	24: single-copy homozygote
17490086	1.30	1	11	1.88	1	11: single-copy homozygote
17490105	0.68	1	17	1.88	1	17: single-copy homozygote
17490123	1.21	1	14	1.95	1	14: single-copy homozygote
17490217	1.36	1	5	1.88	1	5: single-copy homozygote
17490353	1.13	1	4	2.00	1	4: single-copy homozygote
17490404	1.38	1	18	2.03	1	18: single-copy homozygote
17490475	1.23	1	13	2.01	1	13: single-copy homozygote

Transgenic Line	T_0		T_1			T_2
	Ratio (target/ref)	Estimated Copy Number	Homozygote No.	Ratio (target/ref)	Estimated Copy Number of T_0 Plant	Verification of T_1 Homozygote
17490496	1.28	1	9	2.01	1	9: single-copy homozygote
17490513	1.00	1	11	2.01	1	11: single-copy homozygote
17490706	1.29	1	21	1.97	1	21: single-copy homozygote
17491055	1.35	1	3	2.54	1	3: single-copy homozygote
17491085	1.33	1	10	2.01	1	10: single-copy homozygote
17500025	1.39	1	22	1.99	1	22: single-copy homozygote
17500069	1.20	1	11	1.92	1	11: single-copy homozygote
17500125	1.11	1	5	1.94	1	5: single-copy homozygote
17500200	1.26	1	10	2.02	1	10: single-copy homozygote
17500615	1.19	1	10	2.38	1	10: single-copy homozygote
17500661	0.92	1	4	2.30	1	4: single-copy homozygote
17500729	1.30	1	3	2.20	1	3: single-copy homozygote
17500975	1.02	1	5	1.83	1	5: single-copy homozygote
17500989	1.15	1	10	1.98	1	10: single-copy homozygote
17501482	0.76	1	8	1.79	1	8: single-copy homozygote
18010007	1.24	1	19	1.90	1	19: single-copy homozygote
18010241	1.22	1	6	1.92	1	6: single-copy homozygote
18010347	1.18	1	7	2.08	1	7: single-copy homozygote
18010406	1.32	1	7	1.99	1	7: single-copy homozygote
18010410	0.99	1	15	1.91	1	15: single-copy homozygote

Transgenic Line	T_0		T_1			T_2
	**Ratio (target/ref)**	**Estimated Copy Number**	**Homozygote No.**	**Ratio (target/ref)**	**Estimated Copy Number of T_0 Plant**	**Verification of T_1 Homozygote**
18010481	1.17	1	3	1.94	1	3: single-copy homozygote
18010543	1.20	1	8	1.99	1	8: single-copy homozygote
18010633	0.88	1	1	1.85	1	1: single-copy homozygote
18010773	1.23	1	4	2.01	1	4: single-copy homozygote
18011205	1.27	1	1	2.12	1	1: single-copy homozygote
18020222	0.97	1	3	1.97	1	3: single-copy homozygote
18020670	1.31	1	9	1.81	1	9: single-copy homozygote
18020867	1.17	1	17	2.10	1	17: single-copy homozygote
18020891	1.18	1	4	2.02	1	4: single-copy homozygote
18021464	1.17	1	12	2.18	1	12: single-copy homozygote
18010249	1.17	1	10	2.22	1	10: single-copy homozygote
18020067	1.08	1	11	1.95	1	11: single-copy homozygote
18020196	1.25	1	5	1.99	1	5: single-copy homozygote
18020604	1.11	1	14	1.92	1	14: single-copy homozygote
18021495	0.60	1	8	1.89	1	8: single-copy homozygote
18030274	1.26	1	6	1.83	1	6: single-copy homozygote
18030512	1.37	1	5	1.97	1	5: single-copy homozygote
18030888	1.20	1	14	1.98	1	14: single-copy homozygote
18030889	1.33	1	1	1.97	1	1: single-copy homozygote
18030899	1.15	1	1	2.03	1	1: single-copy homozygote

Transgenic Line	T_0		T_1			T_2
	**Ratio (target/ref)**	**Estimated Copy Number**	**Homozygote No.**	**Ratio (target/ref)**	**Estimated Copy Number of T_0 Plant**	**Verification of T_1 Homozygote**
18040628	1.17	1	2	1.80	1	2: single-copy homozygote
18041265	1.08	1	17	2.09	1	17: single-copy homozygote
18041332	1.06	1	5	2.02	1	5: single-copy homozygote
18041422	0.95	1	11	1.92	1	11: single-copy homozygote
18041948	1.11	1	1	1.94	1	1: single-copy homozygote
18042665	0.97	1	20	1.87	1	20: single-copy homozygote
18050215	1.16	1	20	1.98	1	20: single-copy homozygote
18050321	1.23	1	8	3.53	2	8: two-copy homozygote
18050509	1.07	1	7	2.00	1	7: single-copy homozygote
18050873	1.25	1	24	2.04	1	24: single-copy homozygote
18050884	1.04	1	2	2.11	1	2: single-copy homozygote
18050899	1.37	1	2	1.93	1	2: single-copy homozygote
18050912	1.32	1	2	1.85	1	2: single-copy homozygote
18050941	1.35	1	4	1.96	1	4: single-copy homozygote
18051818	1.00	1	3	2.08	1	3: single-copy homozygote
18060333	1.12	1	6	1.88	1	6: single-copy homozygote
18060398	1.04	1	5	1.94	1	5: single-copy homozygote
18060419	1.35	1	9	1.80	1	9: single-copy homozygote
18060471	1.33	1	2	1.94	1	2: single-copy homozygote
18060559	1.25	1	2	1.86	1	2: single-copy homozygote

Transgenic Line	T_0		T_1			T_2
	Ratio (target/ref)	Estimated Copy Number	Homozygote No.	Ratio (target/ref)	Estimated Copy Number of T_0 Plant	Verification of T_1 Homozygote
18061013	1.22	1	8	1.82	1	8: single-copy homozygote
18061145	0.90	1	4	1.74	1	4: single-copy homozygote
18061437	1.17	1	4	2.26	1	4: single-copy homozygote
18070013	1.27	1	9	1.88	1	9: single-copy homozygote
18070471	0.96	1	2	1.98	1	2: single-copy homozygote
18070484	1.27	1	14	2.05	1	14: single-copy homozygote
18041874	1.22	1	9	2.45	1	9: single-copy homozygote
18050530	1.27	1	1	1.94	1	1: single-copy homozygote
18050542	1.12	1	4	2.02	1	4: single-copy homozygote
18061043	1.19	1	2	2.21	1	2: single-copy homozygote
18061171	0.86	1	3	2.11	1	3: single-copy homozygote
18080383	1.37	1	3	2.19	1	3: single-copy homozygote
18080416	1.28	1	3	2.23	1	3: single-copy homozygote
18080501	1.33	1	3	2.57	1	3: single-copy homozygote
18080585	1.07	1	1	2.30	1	1: single-copy homozygote
18080929	1.11	1	4	2.21	1	4: single-copy homozygote
18081416	1.12	1	3	2.17	1	3: single-copy homozygote
18090507	1.37	1	7	2.15	1	7: single-copy homozygote
18090640	1.03	1	12	2.06	1	12: single-copy homozygote
18090641	0.99	1	1	2.56	1	1: single-copy homozygote

Transgenic Line	T_0		T_1			T_2
	**Ratio (target/ref)**	**Estimated Copy Number**	**Homozygote No.**	**Ratio (target/ref)**	**Estimated Copy Number of T_0 Plant**	**Verification of T_1 Homozygote**
18110355	1.15	1	3	2.19	1	3: single-copy homozygote
18110392	1.25	1	7	2.52	1	7: single-copy homozygote
18121011	1.16	1	5	2.14	1	5: single-copy homozygote
18121059	1.03	1	1	2.13	1	1: single-copy homozygote
18130207	1.23	1	1	2.47	1	1: single-copy homozygote
18140008	1.29	1	3	2.05	1	3: single-copy homozygote
18090305	1.31	1	11	2.00	1	11: single-copy homozygote
18100448	1.37	1	6	2.12	1	6: single-copy homozygote
18100533	1.17	1	12	1.98	1	12: single-copy homozygote
18110130	1.17	1	10	1.96	1	10: single-copy homozygote
18130151	1.26	1	2	1.87	1	2: single-copy homozygote
18180208	1.12	1	5	1.89	1	5: single-copy homozygote
18180209	1.02	1	15	1.95	1	15: single-copy homozygote
18180507	1.00	1	2	1.72	1	2: single-copy homozygote
18180516	1.13	1	4	1.80	1	4: single-copy homozygote
18180635	1.09	1	5	2.00	1	5: single-copy homozygote
18180719	1.25	1	4	1.96	1	4: single-copy homozygote
18182389	0.97	1	1	2.00	1	1: single-copy homozygote
18190337	1.22	1	17	3.96	2	17: two-copy homozygote
17430788	1.34	1	3	2.01	1	3: single-copy homozygote

Transgenic Line	T_0		T_1			T_2
	**Ratio (target/ref)**	**Estimated Copy Number**	**Homozygote No.**	**Ratio (target/ref)**	**Estimated Copy Number of T_0 Plant**	**Verification of T_1 Homozygote**
17480508	1.17	1	4	1.99	1	4: single-copy homozygote
17490097	1.04	1	3	1.96	1	3: single-copy homozygote
17490793	1.37	1	10	1.90	1	10: single-copy homozygote
17500063	0.71	1	5	1.97	1	5: single-copy homozygote
18011178	0.97	1	6	2.11	1	6: single-copy homozygote
18011210	1.20	1	2	1.91	1	2: single-copy homozygote
17500659	1.29	1	3	2.09	1	3: single-copy homozygote
18020626	1.13	1	15	1.92	1	15: single-copy homozygote
18021487	0.56	1	14	1.97	1	14: single-copy homozygote
18041162	0.97	1	7	1.98	1	7: single-copy homozygote
18041318	1.37	1	4	2.26	1	4: single-copy homozygote
14320226	1.26	1	7	2.09	1	7: single-copy homozygote
14370681	1.05	1	10	2.47	1	10: single-copy homozygote
15401247	1.33	1	7	1.96	1	7: single-copy homozygote
15471363	1.23	1	15	1.99	1	15: single-copy homozygote
15490969	1.31	1	9	1.94	1	9: single-copy homozygote
16240468	1.29	1	1	2.01	1	1: single-copy homozygote
16270480	1.08	1	1	1.82	1	1: single-copy homozygote
16500265	1.34	1	8	1.86	1	8: single-copy homozygote
17010181	0.98	1	1	1.83	1	1: single-copy homozygote

Transgenic Line	T_0		T_1			T_2
	**Ratio (target/ref)**	**Estimated Copy Number**	**Homozygote No.**	**Ratio (target/ref)**	**Estimated Copy Number of T_0 Plant**	**Verification of T_1 Homozygote**
17010485	1.02	1	1	1.85	1	1: single-copy homozygote
17090047	0.70	1	1	2.13	1	1: single-copy homozygote
17090289	0.63	1	7	2.06	1	7: single-copy homozygote
17110112	1.10	1	1	3.73	2	1: two-copy homozygote
17140037	1.33	1	4	2.07	1	4: single-copy homozygote
17180144	0.87	1	5	1.64	1	5: single-copy homozygote
17190236	1.14	1	6	2.36	1	6: single-copy homozygote
17220798	1.24	1	8	2.05	1	8: single-copy homozygote
17290129	1.07	1	17	1.96	1	17: single-copy homozygote
17290290	1.26	1	3	2.05	1	3: single-copy homozygote
17290443	0.99	1	3	1.80	1	3: single-copy homozygote
17290852	1.26	1	1	2.07	1	1: single-copy homozygote
17310358	1.30	1	4	2.01	1	4: single-copy homozygote
17310506	1.28	1	9	1.99	1	9: single-copy homozygote
17310875	1.00	1	3	1.88	1	3: single-copy homozygote
17340835	1.08	1	7	2.04	1	7: single-copy homozygote
17340926	1.35	1	11	2.07	1	11: single-copy homozygote
17350792	1.25	1	1	2.02	1	1: single-copy homozygote
17360850	1.06	1	2	1.98	1	2: single-copy homozygote
17370784	0.71	1	2	1.88	1	2: single-copy homozygote

Transgenic Line	T_0		T_1			T_2
	Ratio (target/ref)	Estimated Copy Number	Homozygote No.	Ratio (target/ref)	Estimated Copy Number of T_0 Plant	Verification of T_1 Homozygote
17380252	1.09	1	5	1.99	1	5: single-copy homozygote
17400333	1.22	1	2	2.03	1	2: single-copy homozygote
17400429	1.29	1	17	2.05	1	17: single-copy homozygote
17400509	1.39	1	6	2.11	1	6: single-copy homozygote
17400527	1.29	1	7	2.02	1	7: single-copy homozygote
17401002	1.25	1	15	1.83	1	15: single-copy homozygote
17401023	1.14	1	4	2.00	1	4: single-copy homozygote
17421090	1.18	1	6	1.99	1	6: single-copy homozygote
17421134	1.17	1	8	2.21	1	8: single-copy homozygote
17430466	1.30	1	10	2.00	1	10: single-copy homozygote
16230559	0.76	1	1	1.98	1	1: single-copy homozygote
16250196	0.77	1	13	1.82	1	13: single-copy homozygote
17030095	1.08	1	8	2.47	1	8: single-copy homozygote
17100037	0.93	1	1	2.09	1	1: single-copy homozygote
17140511	1.11	1	15	2.00	1	15: single-copy homozygote
17150626	1.03	1	2	2.21	1	2: single-copy homozygote
17160339	0.85	1	3	1.93	1	3: single-copy homozygote
15300500	1.38	1	5	2.06	1	5: single-copy homozygote
15340546	1.18	1	12	1.94	1	12: single-copy homozygote
15360728	1.21	1	2	1.81	1	2: single-copy homozygote

Transgenic Line	T_0		T_1			T_2
	Ratio (target/ref)	Estimated Copy Number	Homozygote No.	Ratio (target/ref)	Estimated Copy Number of T_0 Plant	Verification of T_1 Homozygote
16100054	1.03	1	13	2.02	1	13: single-copy homozygote
16120366	0.81	1	6	2.14	1	6: single-copy homozygote
16141400	1.24	1	7	2.00	1	7: single-copy homozygote
16160178	1.11	1	7	1.95	1	7: single-copy homozygote
16170523	1.00	1	5	1.84	1	5: single-copy homozygote
16180541	1.22	1	2	2.30	1	2: single-copy homozygote
16250034	1.33	1	9	1.97	1	9: single-copy homozygote
16260278	0.76	1	11	1.99	1	11: single-copy homozygote
16340156	1.18	1	4	2.04	1	4: single-copy homozygote
17020324	0.95	1	4	2.03	1	4: single-copy homozygote
17030087	1.13	1	8	2.01	1	8: single-copy homozygote
17090038	1.27	1	5	2.01	1	5: single-copy homozygote
17200281	1.27	1	8	2.18	1	8: single-copy homozygote
17220175	1.10	1	7	2.00	1	7: single-copy homozygote
17220794	1.27	1	6	2.02	1	6: single-copy homozygote
17230491	1.03	1	6	1.95	1	6: single-copy homozygote
17251058	1.19	1	6	2.01	1	6: single-copy homozygote
17280267	1.04	1	1	3.83	2	1: two-copy homozygote
17290299	1.05	1	1	1.98	1	1: single-copy homozygote
17310254	1.25	1	11	1.98	1	11: single-copy homozygote

Transgenic Line	T_0		T_1			T_2
	**Ratio (target/ref)**	**Estimated Copy Number**	**Homozygote No.**	**Ratio (target/ref)**	**Estimated Copy Number of T_0 Plant**	**Verification of T_1 Homozygote**
17310274	1.07	1	4	2.12	1	4: single-copy homozygote
17320030	0.91	1	5	1.98	1	5: single-copy homozygote
17320580	1.04	1	2	1.91	1	2: single-copy homozygote
17321092	0.56	1	4	3.86	2	4: two-copy homozygote
17360198	1.30	1	4	2.13	1	4: single-copy homozygote
17371180	1.21	1	1	2.01	1	1: single-copy homozygote
17391261	1.20	1	11	2.04	1	11: single-copy homozygote
17391523	1.16	1	2	2.06	1	2: single-copy homozygote
17320449	1.02	1	10	1.98	1	10: single-copy homozygote
17430284	0.81	1	2	2.06	1	2: single-copy homozygote
17430595	1.20	1	3	2.29	1	3: single-copy homozygote
17430755	1.11	1	3	1.99	1	3: single-copy homozygote
17440273	1.24	1	1	2.17	1	1: single-copy homozygote
16140128	1.16	1	3	2.07	1	3: single-copy homozygote
15300670	1.31	1	13	1.92	1	13: single-copy homozygote
16100075	1.02	1	3	2.01	1	3: single-copy homozygote
16110103	1.35	1	5	2.03	1	5: single-copy homozygote

Notes: target, transgene *bar*; ref, endogenous gene *hmg*.
