# Supplementary material for: High-Throughput and Accurate Determination of Transgene Copy Number and Zygosity in Transgenic Maize: From DNA Extraction to Data Analysis
Source: Int J Mol Sci. 2021 Nov 19;22(22):12487. doi: 10.3390/ijms222212487 (PMC8619409; doi:10.3390/ijms222212487)
Supplement: Supplementary file 1 [file ijms-22-12487-s001.zip › Table S6.pdf]

Table S6. Primer pairs and probes of bar and hmg genes used in this study.

Type of Assay	Gene Name	Primer/Probe Name	Sequence (5'–3')	Amplicon Size (bp)	Reference
Sequence PCR	*hmg*	hmg-seq-F	ATCCCTGAGCGAGTCGGTAA	201	This paper
		hmg-seq--R	GTTTCCCTACCTCGCCCTTG		
TaqMan qPCR & Digital PCR	*bar*	bar-taq-F	ACAAGCACGGTCAACTTCC	60	[52]
		bar-taq-R	GAGGTCGTCCGTCCACTC		
		bar-taq-Probe	FAM-TACCGAGCCGCAGGAACC-BQ1		
	hmg	hmg-taq-F1	TTGGACTAGAAATCTCGTGCTGA	79	[53]
		hmg-taq-R1	GCTACATAGGGAGCCTTGTCCT		
		hmg-taq-F2	TGGACTAGAAATCTCGTGCTGA	76	This paper
		hmg-taq-R2	TACATAGGGAGCCTTGTCCT		
		hmg-taq-Probe	VIC-CAATCCACACAAACGCACGCGTA-BQ1		
Southern blot	*bar*	bar-southern-F	TTTCGGTGACGGGCAGGAC	484	This paper
		bar-southern-R	TGCACCATCGTCAACCACTA		
Vector construct	*hmg*	hmg-EcoR I-F	CGGAATTCGAAATCCCTGAGCGAGTCGG	229	This paper
		hmg-Hind III-R	CCCAAGCTTAGTAACAACGCAATTGA		
